# Heritable Genetic Effects Caused by a Single Generation of Captive Breeding

**DOI:** 10.1111/eva.70213

**Published:** 2026-03-11

**Authors:** Mark R. Christie, Xiaoshen Yin, Stephanie Bollmann, Michael S. Blouin

**Affiliations:** ^1^ Department of Biological Sciences Purdue University West Lafayette Indiana USA; ^2^ Department of Forestry and Natural Resources Purdue University West Lafayette Indiana USA; ^3^ MOE Key Laboratory of Marine Genetics and Breeding, College of Marine Life Sciences Ocean University of China Qingdao China; ^4^ Department of Integrative Biology Oregon State University Corvallis Oregon USA

**Keywords:** adaptive evolution, captive breeding, epigenetics, genetic adaptation, hatcheries, RNA‐seq, salmon, stocking

## Abstract

Understanding the genetic effects of captive breeding is critical for successful fisheries management and conservation efforts. Recent work has demonstrated that genetic adaptation to captivity, with resulting loss of fitness in the wild environment, can occur in as little as a single generation. To understand the genetic changes underlying such rapid adaptation, we performed crosses within and between first‐generation hatchery and natural‐origin steelhead ( *Oncorhynchus mykiss* ), raised their offspring in a common environment, and used RNA‐seq to examine global patterns of gene expression in those offspring. We found: (i) substantial transcriptomic evidence (hundreds of differentially expressed genes; DEGs) of genetic change induced by a single generation in the captive environment, where the majority of DEGs were upregulated in the offspring of first‐generation hatchery fish (75%), (ii) reciprocal crosses revealed that the differentially expressed genes could not be explained by maternal/paternal effects, chance differences in the background levels of gene expression among unrelated families, or other potential explanations, and (iii) the majority of DEGs are related to growth, development, behavior, immune response, and metabolism. These results demonstrate that a single generation of captive breeding results in the heritable activation of hundreds of genes with consequences for stocking or reintroduction efforts.

## 1 Introduction

Captive breeding and captive breeding programs are becoming increasingly necessary due to the accelerating pace of environmental change (Crates et al. 2023; Frankham 2008; IUCN Conservation Planning Specialist Group 2020; Seddon et al. 2007). Habitat loss, climate change, pollution, and novel infectious diseases are just some of the contemporary challenges faced by natural populations that may require the adoption of captive breeding to ensure their continued persistence (Butchart et al. 2010; Watson et al. 2019). On the one hand, successful captive breeding programs can result in increases in natural and captive population abundance (Ruggerone et al. 2010), facilitate public engagement (Bowler et al. 2012; Whitehouse et al. 2014), and provide a stopgap of last resort as an *ex situ* holdover while habitat is restored (Frankham et al. 2010). On the other hand, unintentional genetic effects of captive breeding can reduce the fitness of individuals when released back into nature. These negative effects can include loss of genetic diversity (e.g., genetic drift, inbreeding), relaxed natural selection on deleterious alleles, and genetic adaptation to captivity (Bryant and Reed 1999; Christie, Ford, and Blouin 2014; Fisch et al. 2015; Gering et al. 2019).

Although captive breeding programs often have plans to release captive‐born individuals back into nature (Frankham et al. 2010) many programs only release individuals on a small‐scale in a limited number of locations (Brichieri‐Colombi et al. 2019) or are waiting for habitat restoration and policy changes (e.g., decreased poaching; Wasser et al. 2015). By contrast, many captive‐born fish species are often stocked into natural water bodies to enhance recreational and commercial fisheries (Araki and Schmid 2010; Cowx et al. 2010). Pacific salmon reared in hatcheries are one example of this phenomenon with nearly 5.5 billion captive‐born salmon smolts intentionally released into tributaries of the Pacific Ocean each year (NPAFC 2023). The large scale of these hatchery programs, both in terms of the number of individuals and the number of breeding programs, suggests that rapid genetic adaptation to captivity may be more likely to occur (or more likely to simply be detected) in fishes than in other taxa (Willoughby and Christie 2019). Previous work on salmon and their relatives has demonstrated that they can respond to selection imposed by novel environments remarkably quickly (Fraser et al. 2011; Hendry et al. 2000; Pearse et al. 2009; Quinn et al. 2001; Willoughby et al. 2018), including captivity (Blouin et al. 2021; Christie, French, et al. 2014). Why salmon are so adaptable remains an open question; some non‐mutually exclusive possibilities include a whole genome duplication event that occurred approximately 100 million years ago (Allendorf et al. 1984; Berthelot et al. 2014), relatively large effective population sizes and high levels of standing genetic variation (Quinn 2018; Sparks et al. 2022), high fecundity (Quinn 2018), and a complex, yet flexible genomic architecture (Berthelot et al. 2014; Lien et al. 2016).

In Pacific salmon, genetic adaptation to captivity and loss of fitness in the wild has been observed after a single generation of captive breeding (Araki, Ardren, et al. 2007; Araki, Cooper, and Blouin 2007; Christie, Marine, French, and Blouin 2012; Farquharson et al. 2021; Milot et al. 2013). An early review of fitness in pedigreed salmon populations revealed that the lifetime reproductive success of first or early‐generation hatchery fish averages only half that of natural‐origin fish when both groups spawn in the wild, and at least some of that reduction in fitness is due to genetic adaptation to captivity (Christie, Ford, and Blouin 2014; O'Sullivan et al. 2020). Previous studies examining patterns of lifetime reproductive success in first‐generation hatchery and natural‐origin steelhead ( *Oncorhynchus mykiss* ) from the Hood River, Oregon (USA) found that first‐generation hatchery fish had a 15% reduction in lifetime reproductive success compared to natural‐origin fish when mating in the wild (Araki, Ardren, et al. 2007; Araki, Cooper, and Blouin 2007), but nearly twice the lifetime reproductive success of natural‐origin fish when mating in captivity (Christie, Marine, French, and Blouin 2012). Gene expression analyses (e.g., RNA‐seq) on fish from the same population identified genes related to metabolism, disease, and wound healing as possible targets of selection (Christie et al. 2016), the latter of which were also corroborated by genomic analyses of RNA‐seq data from Atlantic salmon ( *Salmo salar* ) (Harder and Christie 2022). These studies, in conjunction with additional experiments (e.g., Blouin et al. 2021), suggest that selection for enhanced growth rate, tolerance to novel pathogens, and coping with the direct and indirect effects of high rearing density (e.g., aggression) may play a role in facilitating genetic adaptation to captivity. Nevertheless, more studies with additional populations are required to understand both the traits that are under selection in the captive environment and the specific agents of selection that are driving genotypic and phenotypic responses.

**FIGURE 1:**
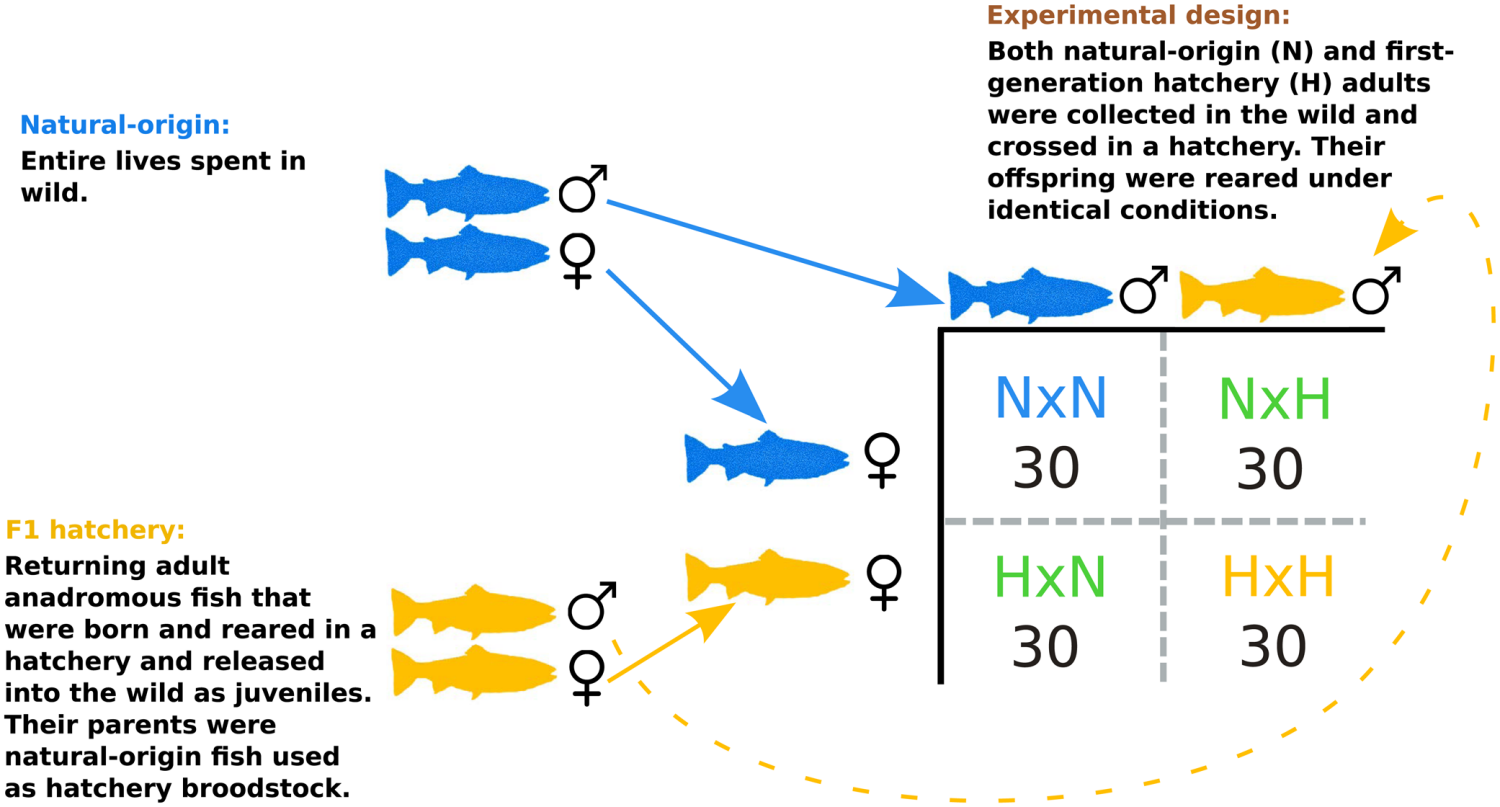
Illustration of the experimental design used in this study. Natural‐origin (N) and first‐generation hatchery (H) adults were collected from the Siletz River and crossed in a captive environment (a salmon hatchery). A total of 15 independent 2 × 2 matrices (a hatchery‐ and natural‐origin male crossed with a hatchery‐ and natural‐origin female) were created, and two offspring (one male, one female) were sequenced per full‐sibling family. All offspring were reared in a common environment. This design created 15 independent H × H families (two hatchery‐origin parents) that could be compared with 15 independent N × N families (two natural‐origin parents) and also created 15 H × N and 15 *N* × H reciprocal crosses which were used to make comparisons between families with equal amounts of captive ancestry and to investigate the influence of maternal and paternal effects.

To determine the number and identity of genes affected by the captive environment, we compared the transcriptomic responses between the offspring of first‐generation hatchery and natural‐origin steelhead reared in a common (hatchery) environment (Figure 1). Parents of known origin (hatchery versus natural) were collected as returning adults from the Siletz River, Oregon (USA), crosses were performed at a fish hatchery, and the eggs and offspring were reared under identical, controlled conditions until the swim‐up fry stage (yolk sac absorption). First‐generation hatchery fish had natural‐origin parents and only spent their first year in the hatchery before being released into the wild. A series of 2 × 2 crosses utilizing a hatchery and natural‐origin female crossed to a hatchery and natural‐origin male were implemented (15 2 × 2 matrices) to produce four types of families: two first‐generation hatchery parents (H × H), two natural‐origin parents (N × N), or one hatchery and one natural‐origin parent (H × N and N × H reciprocal crosses; Figure 1 and Table S1). The offspring were reared in identical environments after which we used RNA‐seq (*N* = 120 offspring) to ask the following questions: (1) are there more genes differentially expressed between the offspring of natural‐origin fish and the offspring of first‐generation hatchery fish than between the offspring of fish with identical amounts of hatchery and natural‐origin ancestry (the H × N and N × H reciprocal crosses reciprocal crosses)?, (2) is there any evidence of the differential expression being driven by maternal or paternal effects, which could represent carry‐over environmental effects (e.g., condition) and not necessarily a response to selection in captivity?, and (3) which genes are overrepresented in comparisons between first‐generation hatchery and natural‐origin offspring and what is their general function and likely contribution to phenotype? We find that hundreds of genes are differentially expressed between the offspring of first‐generation hatchery fish (H × H) and the offspring of natural‐origin fish (*N* × N), but not between the offspring of the H × N and N × H fish. Furthermore, these differences in gene expression cannot be explained by maternal/paternal effects, cross date, sampling noise, or false discovery.

## 2 Materials and Methods

### 2.1 Sample Information and Experimental Design

The hatchery‐origin and natural‐origin Siletz River adults used to create the crosses were collected as they returned to spawn (hatchery‐origin individuals were released into the Siletz as smolts and were identified by their fin clips; see Wilson 2008). Each adult was used in only two crosses (one main effects cross—e.g., H × H—and one reciprocal cross e.g., H × N, where the first H denotes the same fish), and never across matrices (see Tables S1 and S2 for complete family information; Figure 1 for crossing design). For all crosses we use H to denote a hatchery‐origin adult, N to denote a natural‐origin adult, where we first indicate the environment of the mother followed by the father (e.g., H × N represents an offspring with a hatchery‐origin mother and a natural‐origin father; see Figure 1). All offspring were reared until the swim‐up fry stage at which point the fry were frozen in liquid nitrogen and transferred to a −80°C freezer for storage. All fry were collected on the same day and at the same time of day (±1 h) to minimize changes in gene expression due to development or circadian rhythms (Reebs 2002). We sequenced two offspring (one male and one female) from every cell of all 15 2 × 2 matrices (Figure 1) resulting in a total of 120 fish sequenced (2 siblings per family × 4 cells per matrix × 15 matrices = 120 individuals). The parents of the crosses were collected as returning adults from the Siletz River, Oregon (USA). Crosses were performed at the Alsea Falls fish hatchery, and the eggs and offspring were reared under identical, controlled conditions at the Oregon Hatchery Research Center. Additional details regarding hatchery rearing practices can be found in Supporting Information.

### 2.2 Sequencing and Differential Expression

Total RNA was isolated from whole frozen fry using a modified trizol/chloroform protocol described previously (Christie et al. 2016; Fox et al. 2014). Briefly, RNA was extracted using Trizol Reagent (Invitrogen). Total RNA was treated for 10 min at 65°C with RNAsecure reagent (Ambion) and for 10 min at 37°C with RNase‐free TURBO DNase (Ambion). Total RNA was further purified using the RNeasy Mini Kit (Qiagen) according to the manufacturer's protocol. Concentration, integrity, and extent of contamination by ribosomal RNA were assessed using Qubit Fluorometer (Life Technologies) and Bioanalyzer 2100 (Agilent Technologies). Preparation of cDNA for sequencing was completed following the TruSeq RNA Sample Preparation Kit v2 protocol (Illumina). Each individual had a unique barcode, and barcodes were assigned evenly across sample types. All reads were aligned to *O. mykiss* genome USDA_OmykA_1.1 (GCF_013265735.2; Gao et al. 2021) and only reads that aligned to a single location were retained; additional details on sequence alignment can be found in Supporting Information.

We used two approaches to identify genes that were differentially expressed between the offspring of two hatchery‐origin parents (H × H) and two natural‐origin parents (N × N). First, we used DESEQ2 (Love et al. 2014) using the model: design ~cross_date + sex + ancestry where cross date and sex were fixed effects. Cross date represented the day that the parents of the F_1_ offspring were spawned (Table S1), and sex represented the genetic sex of the sequenced fry, where sex was identified via PCR of a sex‐specific marker *OmyY1* (Brunelli et al. 2008) (information for every individual are available in Table S2). Ancestry denoted whether the offspring was the product of an H × H or N × N mating (Figure 1) and was the primary focus of our study. We considered genes with a false discovery rate (FDR)‐adjusted *p*‐value < 0.05 to be differentially expressed. Because DESEQ2 cannot account for random effects, it cannot explicitly account for the multiple siblings sequenced within each family (Table S1). Thus, for both sets of samples, we also used LIMMA (Ritchie et al. 2015; Smyth 2005) to estimate the correlation in gene counts between siblings using the duplicateCorrelations function. The between‐sibling correlation was estimated as only 0.13, and this low value suggests that the effect of sibling is not substantially contributing to the differentially expressed genes identified by DESEQ2. All of the differentially expressed genes identified by LIMMA (*N = 336*) were also identified by DESEQ2 (*N = 385*). We focused on DESEQ2 output for subsequent analyses because the between‐sibling correlation was low and the design of this gene expression analysis was exploratory in nature. We used the same DESEQ2 model described above (changing the individuals in “ancestry”) to identify genes differentially expressed between the H × N and *N* × H offspring.

Given that a total of 385 DEGs were identified in the H × H versus N × N crosses and only five DEGs were identified in the H × N versus N × H crosses, we wanted to determine the probability of observing this pattern by chance. We addressed this possibility in two ways. First, we wanted to identify the effect size of hatchery or natural origin on the number of DEGs identified between H × H and N × N offspring. To do so, we took the entire set of offspring (including H × N and N × H offspring), and randomly sampled them without replacement and without respect to their identity and assigned them to two groups of 30 individuals. We constrained the number of H × H and N × N offspring allowed in each of the two groups, ranging from 1 to 30 in increments of 5. Thus, when the constraint was set to 1, each of the two reshuffled sample groups would contain either only 1 H × H offspring (the remainder consisting of H × N and N × H offspring) or 1 N × N offspring. When the constraint was set to 30, this simply represented our empirical data set. For each level of constraint, 1000 replicates were performed and the mean number of DEGs (FDR‐adjusted *p*‐value < 0.05) and 95% confidence intervals were plotted. For a second analysis, we next took the combined set of H × H and N × N offspring, randomly sampled them without replacement and without respect to their H × H and *N* × N identity and assigned them to two groups. Because there were 30 offspring in each group, each shuffled group had a sample size of 30. We again calculated the number of DEGs with an FDR *p*‐value < 0.05 using the methods described above. We repeated this procedure 1000 times and, to calculate the probability of our results occurring by chance, we divided the number of re‐shuffled datasets that identified as many or more DEGs as the empirical dataset by 1000. All calculations were performed with R 4.4.1 (Team, R. C 2024) and DESEQ2 where the testing of re‐shuffled RNA‐seq datasets were performed on a high‐performance computing cluster (https://www.rcac.purdue.edu/).

### 2.3 Alternative Explanations: Maternal/Paternal Effects, Relatedness, and Offspring Sex

**FIGURE 2:**
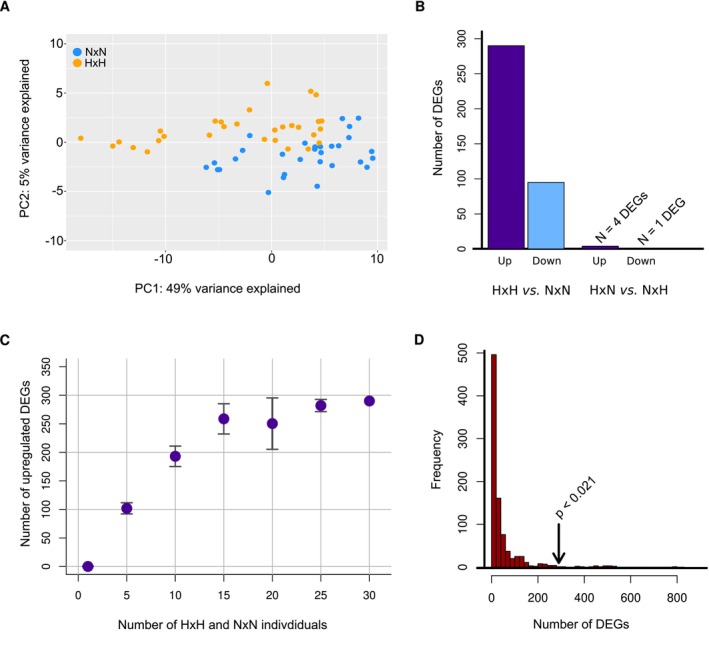
Examination of main effects. (A) Principal coordinates of the differentially expressed genes reveal clear differences between the offspring of the N × N crosses (blue points) and the offspring of the H × H crosses (orange points). (B) A total of 385 differentially expressed genes (DEGs) were identified between the H × H and N × N offspring, where 290 DEGS were upregulated in the H × H offspring (purple bar) and 95 genes were upregulated in the N × N offspring. A total of only 5 DEGs were identified between the H × N and N × H offspring (C) Randomization procedures split all offspring into two groups where one group contained exactly *N* H × H individuals and the other group contained exactly *N* N × N individuals. All individuals were sampled randomly, the remaining 30‐*N* sampled individuals were sampled with replacement from H × N versus N × H individuals (30 being the empirical sample size of each group), and the number of DEGs calculated. After 1000 randomizations, it is clear that the number of upregulated DEGs increases as a function of hatchery ancestry. Bars represent 95% confidence intervals (there are no bars for *N =* 30, which represents the empirical result, and bars for *N = 1* are too small to see). (D) A conservative randomization procedure using only H × H and *N* × N offspring revealed that the probability of observing 290 upregulated DEGs is less than 0.021.

**FIGURE 3:**
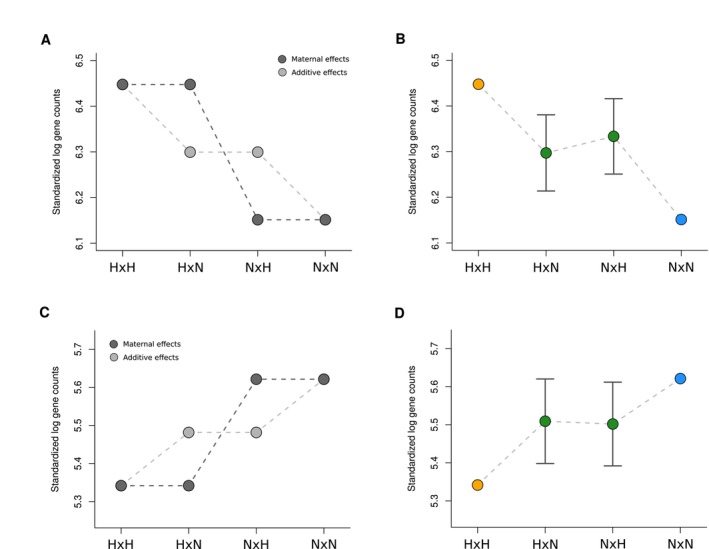
Examination of potential maternal and paternal effects. To test for these effects, we split the 385 main effects DEGs into those that were upregulated in hatchery H × H offspring (*n =* 290; panels A, B) and those that were upregulated in natural‐origin N × N offspring (*n =* 95; panels C, D). (A) Conceptual illustration of predicted effects: If maternal effects were driving the main effects, we would expect to see similar mean standardized gene counts between offspring that share a mother (H × H vs. H × N and N × H vs. N × N, where maternal environment is indicated first) (dark gray circles). Alternatively, if maternal effects are not driving the main effects, then the standardized gene counts should be additive (light gray circles). If paternal effects were driving the main effects, we would expect to see similar mean standardized gene counts between offspring that share a father (H × H vs. H × N and N × H vs. N × N) (Figure S1). (B) Empirical mean of the standardized log gene counts (±standard error) for the DEGs illustrating that the mean gene counts are remarkably additive and not driven by maternal effects. (C, D) Same as (A, B), but for the main effect DEGs that were upregulated in N × N offspring. Combined, these results illustrate that the main effects DEGs (i.e., those that differ between H × H and *N* × N offspring) are not driven by maternal or paternal effects and thus most likely to be driven by a response to selection in the hatchery environment or transgenerational epigenetic effects.

One possible explanation for our results is that the differences in gene expression between the H × H and N × N could be influenced by heritable maternal or paternal effects (Wolf and Wade 2009; Matos 2012). If the differences in gene expression were simply due to maternal effects (the mothers of the hatchery‐origin offspring and the natural‐origin offspring experienced different environments), then we would expect to see two patterns. First, the number of DEGs between H × H and N × N offspring should equal that between H × N and N × H offspring. This was clearly not the case (Figure 2B). Second, standardized gene counts should be similar between the offspring of the H × H fish and the offspring of the H × N fish because both groups of offspring shared the same hatchery mother (Figure 3A). Likewise, we would expect to see standardized gene counts that were similar between the offspring of *N* × N fish and N × H fish because both groups shared the same natural‐origin mother. A similar argument would apply for paternal effects.

To further determine whether the DEGs were affected by maternal or paternal effects we first isolated the standardized gene counts for all 120 individuals at the 385 DEGs (H × H vs. N × N) and separated the DEGs into those that were upregulated in the H × H offspring compared to the N × N offspring versus those that were upregulated in N × N offspring compared to the H × H offspring. For visualization purposes, we took the log of the standardized counts and then calculated the mean of the counts separately for each of the H × H, H × N, N × H, and H × N groups and plotted the means and standard errors. To illustrate the changes in expression for every DEG separately, we further standardized all gene counts to the mean value of the upregulated group by adding the difference between the observed H × H and the mean H × H gene count to every gene; this procedure was only implemented to assist with visualization where the higher variance in gene counts among all genes makes it more challenging to visualize patterns simultaneously.

We also calculated pairwise relatedness among all offspring samples. Relatedness can be higher among hatchery offspring compared to natural‐origin offspring (Christie, Marine, French, and Blouin 2012), and if relatedness were higher among the H × H offspring than the *N* × N offspring, this possibility could explain some of our results. To calculate relatedness, we first called SNPs from our RNA‐seq data set. Details regarding our SNP calling pipelines can be found in Supplementary Methods. A total of 11,846,925 SNPs were initially called from all 120 individuals during variant discovery. After variant discovery, SNPs were first filtered with the flag ‐‐max‐missing‐count to remove loci that were missing in more than 20% of individuals (*n =* 28 loci). We next removed individuals that had more than 20% of missing data. This resulted in the removal of one individual (S115), which had 29% missing data. The average missing data per individual was 3.3%. We next filtered out invariant sites and sites with a minor allele frequency less than 0.01, resulting in 626,075 SNPs. Using the SNPs called above, we calculated pairwise relatedness using VCFTOOLS (Danecek et al. 2011) and the ‐‐relatedness2 flag, which calculates a relatedness statistic based on the method of Manichaikul et al. (2010). The genetic sex of all offspring was identified via PCR of a sex‐specific marker *OmyY1* and was determined prior to RNA‐seq such that we could balance males and females (60 female, 60 male; Table S2). We also used DESEQ2 to explicitly test for differences between sexes.

### 2.4 Annotation and Gene Function

After identifying DEGs, the merged GTF file was used to create a bed file, and BEDTOOLS (Quinlan and Hall 2010) was used to isolate the sequences from the reference genome. We next used eggNOG 5.0 (Huerta‐Cepas et al. 2019) to identify both the gene names and their associated GO terms. To identify and visualize the GO terms we used REVIGO 1.8.1 (Supek et al. 2011) for all the GO terms associated with DEGs, using a list value of 0.7 (default), SimRel as the semantic similarity metric, removing obsolete GO terms, and selecting *Danio rerio* as a reference. After importing the results associated with Biological Processes into R, we identified terms to illustrate using k‐means clustering (Hartigan and Wong 1979) on PC1 and PC2 values. We chose to highlight 9 clusters by performing k‐means clustering using different values of clusters *k*, calculating the within sum of squares drawn according to the number of clusters, and using the location of the bend (knee) in the plot as an indicator of the appropriate number of clusters using the *fviz_nbclust()* in the package *factoextra* (Kassambara and Mundt 2020). For each cluster, the GO term found in the largest number of DEGs was used to name the cluster. We also isolated the three clusters with the largest within group sum of squares, and for each cluster individually, we split all GO terms into individual words, identified the word used in highest frequency (most frequent word in first cluster = “development”, most frequent word in second cluster = “development”, most frequent word in third cluster = “behavior”), and labeled the 6 highest frequency GO terms using that term (the third cluster only had 5). Importantly, the k‐means clustering approach was only used for visually illustrating and simplifying GO terms; the complete list of annotation and GO terms is available in Table 1 and Tables S3–S6, and Extra Table S1.

**TABLE 1:** GO terms associated with the natural‐ versus hatchery‐origin DEGs and reduced according to REVIGO.

Cluster	Count	Go term
#1B9E77	60	Anatomical structure development
#1B9E77	47	Cellular developmental process
#1B9E77	46	Cell differentiation
#1B9E77	40	Animal organ development
#1B9E77	35	Anatomical structure morphogenesis
#1B9E77	27	Tissue development
#1B9E77	19	Animal organ morphogenesis
#1B9E77	17	Anatomical structure formation involved in morphogenesis
#1B9E77	17	Epithelium development
#1B9E77	17	Muscle structure development
#D95F02	56	Multicellular organism development
#D95F02	49	System development
#D95F02	26	Nervous system development
#D95F02	16	Embryo development
#D95F02	14	Neuron development
#D95F02	12	Post‐embryonic development
#D95F02	11	Pattern specification process
#D95F02	11	Tube development
#D95F02	10	Skeletal system development
#D95F02	10	Tube morphogenesis
#7570B3	22	System process
#7570B3	18	Behavior
#7570B3	12	Muscle system process
#7570B3	11	Locomotory behavior
#7570B3	8	Cell activation
#7570B3	8	Circulatory system process
#7570B3	8	Blood circulation
#7570B3	8	Leukocyte activation
#7570B3	7	Muscle contraction
#7570B3	6	Adult locomotory behavior
#E7298A	40	Organelle organization
#E7298A	32	Cellular component biogenesis
#E7298A	16	Cytoskeleton organization
#E7298A	15	Protein‐containing complex organization
#E7298A	15	Membrane organization
#E7298A	14	Protein‐containing complex assembly
#E7298A	12	Mitochondrion organization
#E7298A	12	Actin cytoskeleton organization
#E7298A	11	Cell projection organization
#E7298A	11	Organelle assembly
#66A61E	45	Response to chemical
#66A61E	40	Cellular response to stimulus
#66A61E	37	Response to stress
#66A61E	31	Response to organic substance
#66A61E	29	Response to external stimulus
#66A61E	26	Signal transduction
#66A61E	22	Response to endogenous stimulus
#66A61E	20	Response to abiotic stimulus
#66A61E	19	Response to oxygen‐containing compound
#66A61E	18	Response to nitrogen compound
#E6AB02	49	Transport
#E6AB02	31	Cellular localization
#E6AB02	26	Macromolecule localization
#E6AB02	25	Organic substance transport
#E6AB02	23	Nitrogen compound transport
#E6AB02	21	Vesicle‐mediated transport
#E6AB02	19	Monoatomic ion transport
#E6AB02	17	Transmembrane transport
#E6AB02	16	Monoatomic ion transmembrane transport
#E6AB02	16	Intracellular transport
#A6761D	16	Negative regulation of response to stimulus
#A6761D	15	Regulation of intracellular signal transduction
#A6761D	14	Negative regulation of cell communication
#A6761D	14	Negative regulation of signaling
#A6761D	12	Regulation of response to stress
#A6761D	10	Positive regulation of response to stimulus
#A6761D	9	Positive regulation of cell communication
#A6761D	9	Positive regulation of signaling
#A6761D	8	Regulation of MAPK cascade
#A6761D	7	Regulation of defense response
#666666	30	Regulation of multicellular organismal development
#666666	14	Negative regulation of developmental process
#666666	11	Regulation of system process
#666666	11	Regulation of neuron differentiation
#666666	9	Regulation of developmental growth
#666666	8	Regulation of muscle system process
#666666	7	Regulation of striated muscle tissue development
#666666	7	Regulation of muscle organ development
#666666	7	Regulation of muscle tissue development
#666666	6	Regulation of cell morphogenesis
#B2DFEE	73	Cellular metabolic process
#B2DFEE	72	Organic substance metabolic process
#B2DFEE	69	Primary metabolic process
#B2DFEE	63	Nitrogen compound metabolic process
#B2DFEE	55	Organonitrogen compound metabolic process
#B2DFEE	51	Macromolecule metabolic process
#B2DFEE	39	Protein metabolic process
#B2DFEE	37	Biosynthetic process
#B2DFEE	37	Organic substance biosynthetic process
#B2DFEE	31	Cellular nitrogen compound metabolic process

*Note:* This table complements the terms highlighted in Figure 4 and lists the top 10 most abundant GO terms from each k.means cluster where each row indicates the HEX colors used to delineate each cluster, Count represents the number of distinct DEGs that contain a particular GO term, and the GO term description itself. Here, we show the top 10 most commonly occurring terms across DEGs per cluster; all GO terms associated with each DEG are available in Table S4 and the complete table for the GO terms per cluster is available in Table S5.

## 3 Results

### 3.1 Main Effects

We identified a total of 385 genes that were differentially expressed between H × H and N × N offspring with an FDR corrected *p*‐value of less than 0.05 (Figure 2A). The genomic position of all DEGs along with their *p*‐value, FDR corrected *p*‐values and log fold changes are given in Table S3. Out of all 385 DEGs, a total of 290 genes (75%) were upregulated in H × H offspring compared to N × N offspring whereas only 95 genes (25%) were upregulated in N × N offspring compared to H × H offspring (Figure 2B). The number of differentially expressed genes was highly dependent on the amount of captive vs. natural‐origin ancestry present in the samples being compared. Here we found a total of 385 genes differentially expressed between H × H and N × N fish, whereas there were only 5 genes differentially expressed between H × N and N × H fish (Figure 2B). We further found, via randomization tests, that as the proportion of H × H and N × N individuals within each of two samples increases, so too does the number of differentially expressed genes (Figure 2C). This result would not be expected if something other than hatchery ancestry were driving this effect. Importantly, experimental and rearing conditions, sample sizes, read depth, and statistical analyses were identical between these comparisons (Tables S1 and S2); the only variable that differed was the amount of hatchery or captive ancestry present in the offspring. Lastly, when simply randomizing H × H and *N* × N individuals between two samples, this conservative procedure revealed that the probability of observing 385 DEGs was < 0.048 and the probability for observing 290 upregulated and 95 downregulated genes was < 0.021 (Figure 2D) and 0.15, respectively.

### 3.2 Alternative Explanations

If the differences in gene expression were simply due to maternal effects, then we would expect to see two patterns. First, the number of DEGs between H × H and N × N crosses should equal that between H × N and N × H crosses. This was clearly not the case (Figure 2B). Second, standardized gene counts should be similar between the offspring of H × H fish and the offspring of H × N fish because both groups of offspring shared the same hatchery mother (Figure 3A). Likewise, we would also expect to see standardized gene counts that were similar between the offspring of N × N fish and N × H fish because both groups shared the same natural‐origin mother. However, among all crosses, we observed nearly additive effects, where the standardized gene counts for both H × N and N × H fish were intermediate between the gene counts for the H × H and N × N offspring (Figure 3B; Figures S1 and S2). We see a similar pattern for genes that were downregulated in H × H fish (Figure 3C,D) Likewise, if the differences in gene expression were simply due to paternal effects, then we expect to see similar gene counts between the H × H and N × H individuals, where both groups share a father (Figures S1 and S2), and between the N × N and H × N individuals, where both groups share again share a father. This was also not the case (Figure 2B,D). Combined, these results suggest that the differences in gene expression between the H × H and *N* × N offspring are not due to the different rearing environments experienced by their mothers or fathers. Lastly, we found that the relatedness among H × H offspring did not differ from that among N × N offspring (Figure S3) and that there were very few DEGs between male and female offspring (*n* = 4 genes).

### 3.3 Gene Function

**FIGURE 4:**
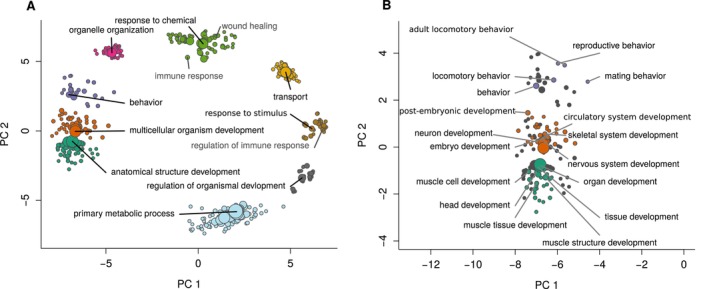
Illustration of gene ontology (GO) terms identified from the 385 genes differentially expressed between the H × H and *N* × N offspring. (A) After reducing and plotting the GO terms based on semantic similarity, we labeled each cluster with the GO term that was shared by the largest number of DEGs. Clusters relating to anatomical structure development, multicellular organismal development, behavior, organelle organization, response to chemicals, transport, response to stimulus, regulation of organismal development, and primary metabolic process were identified. Three terms that were shared with other RNA‐seq studies investigating the genetic effects of salmon hatcheries (“wound healing”, “immune response”, and “regulation of immune response” were also labeled. B) zooming in on the three clusters with the largest within group sum of squares, we used colored points to highlight the most commonly occurring GO terms and we label the 6 most frequent terms for each cluster (the third cluster only had 5). The top 10 most abundant GO terms from each k.means cluster can be found in Table 1 and the full list of GO terms can be found in Table S6. Combined, these results suggest that genes related to growth, development, behavior, immune responses and metabolism are responding to the novel hatchery environment. Table S1: For the 385 DEGs identified between the H × H and *N* × N offspring, 348 were successfully annotated with eggNOG. All eggNOG output and GO terms are reported for these 348 genes.

For the 385 DEGs identified between the H × H and *N* × N offspring, 348 were successfully annotated with eggNOG. A brief description of the annotated DEGs is available in Tables S3 and S4 (all eggNOG output and GO terms per gene are available in Extra Table S1). After reducing and clustering the GO terms based on semantic similarity, we found that these genes describe functions related to anatomical structure development, multicellular organismal development, behavior, organelle organization, response to chemicals, transport, response to stimulus, regulation of organismal development, and primary metabolic process (Figure 4a). Focusing on the three largest, co‐occurring clusters we find terms associated with muscle development, organ development, skeletal system development, circulatory system development, neuron development, locomotory development, and reproductive and mating behaviors among others (Figure 4b; Table 1). The number of occurrences of each GO term in the annotated DEG dataset along with the GO descriptions and relevant REVIGO output can be found in Table 1 and Table S6 (the full list of annotated DEGs, GO terms, and other annotation information are available in Extra Table S1). Combined, these results suggest that genes related to growth, development, behavior, immune responses and metabolism are responding to the novel hatchery environment.

## 4 Discussion

By examining the transcriptomic signatures of 120 individuals, we found that a single generation of captive breeding resulted in the differential expression of hundreds of genes between the offspring of two natural‐origin parents and two captive‐origin parents. The offspring were reared in a common environment, such that the primary difference between offspring was a single generation of captive ancestry in their parents. We further showed that these large differences in gene expression could not be explained by maternal effects, paternal effects, cross date, offspring sex, or relatedness. Likewise, there were no differences in sample sizes, read depth, or read quality among the different treatments. Importantly, the number of differentially expressed genes between the offspring from crosses with equal amounts of hatchery ancestry (H × N vs. N × H) yielded far fewer differentially expressed genes (5 vs. 385; Figure 2b) despite these offspring being treated identically to the offspring with two captive‐ or two natural‐origin parents (H × H vs. N × N). These results independently corroborate the results of Christie et al. (2016), which found similar differences in expression between H × H and *N* × N offspring using steelhead from the Hood River.

One interesting result of the current study is that substantially more DEGs were upregulated in H × H fish (75% vs. 25% upregulated for H × H vs. *N* × N offspring; *χ* ^2^ = 125.35; *p* < 0.0001). This result was not expected a priori, but suggests that responding to the novel, captive environment may include a generalized de‐repression of transcription (e.g., via heritable epigenetic effects) or a change in key transcription factors that affect multiple loci (e.g., Truby et al. 2024), in addition to adaptive change at multiple loci. In many experimental transcriptomics studies, there is an over‐representation of genes that are upregulated in the treatment group. For example, when diverse taxa are exposed to temperature stress there are often many more genes upregulated in the experimental treatment than the control group (e.g., Backenstose et al. 2026; Lee et al. 2025; Wen et al. 2019). This result suggests that hatchery exposure is an experience that may, at best, simply deviate from typical “control” (i.e., wild) conditions and, at worst, may cause a long‐term stress response that manifests across generations. Regardless of the mechanism, these results illustrate large, heritable transcriptomic responses to a single generation of captive breeding. Very few genes were differentially expressed between male and female offspring (*N* = 4). Because we found a nearly identical result in Hood River offspring (Christie et al. 2016), this result suggests that the expression of genes related to sex‐specific development does not happen until later in life (in both studies fry were sampled at the swim up stage, just after yolk absorption). A follow‐up study characterizing the expression profiles of sex‐determining genes throughout *O. mykiss* development seems warranted.

In this study, we found that a substantial number of DEGs have functions related to development, growth, metabolism, and behavior. This result is consistent with other studies (Reisenbichler et al. 2004; Christie et al. 2016; Blouin et al. 2021; Harder and Christie 2022) that suggest the captive environment is selecting on traits associated with growth rate, metabolism, immunity, and development. For example, steelhead hatcheries usually rear individuals on large quantities of protein‐rich food and release all individuals at 1 year of age to immediately migrate out to the ocean. In the natural‐environment, by contrast, steelhead can take anywhere from 1 to 3 years (typically 2 years) to begin their ocean‐migration (Quinn 2018). Because size‐at‐release from hatcheries is positively correlated with survival at sea (where the probability of survival is low), it is hypothesized that there is intense selection for any traits that promote accelerated growth in the hatchery environment (Blouin et al. 2021). We also downloaded the gene names and GO terms from five similar studies (Christie et al. 2016; Harder and Christie 2022; Howe et al. 2024; Leitwein et al. 2022; Le Luyer et al. 2017) to compare with the genes and GO terms identified in this study. Many studies reported only GO terms or gene names making direct comparisons challenging. Nevertheless, we identified five genes shared between this study and others: *FSTL1, PLPL9, SURF4, EGR2B*, and *ACTN3*. The specific function of each gene while generally known (*FSTL1—*adipose tissue development, growth; *PLPL9*—lipid metabolism and growth; *SURF4*—growth; *EGR2B*—nervous system development and behavior; *ACTN3*—known as the “speed gene” in humans—may suggest selection against fast‐twitch muscle in a hatchery environment) remain poorly characterized in salmonids, but further confirm our hypotheses for what traits may be under selection in the hatchery environment: development, growth, metabolism, and behavior. A total of 3007 out of our 4338 (69%) unique GO terms matched to other studies, however, the actual amount of overlap is difficult to quantify given the hierarchical nature of GO terms. A more thorough analysis of genes associated with hatchery domestication (e.g., a formal meta‐analysis) exploring this topic further may be fruitful.

By virtue of the experimental design and subsequent analyses, we were able to rule out many competing explanations for our results. For example, the majority of DEGs were expressed in an additive manner (Figure 3 and Figure S2) with very few genes showing evidence of being influenced by maternal or paternal effects. This result does not mean that maternal or paternal effects are not important in salmon per se (reviewed in Wolf and Wade 2009; Houde et al. 2015), but rather our results strongly suggest that maternal or paternal effects are not driving the differences in gene expression between the H × H and N × N offspring. We were also able to directly rule out parental relatedness (Figure S1), offspring relatedness (LIMMA), offspring sex, cross date (Figure S3), and technical artifacts (e.g., read depth, unbalanced sample sizes) as competing explanations. We can also indirectly rule out some additional explanations. For example, hybridization or introgression with summer‐run steelhead (or even different *Oncorhynchus* species) would be apparent both from differences in read depth (after alignment) and would create distant outliers in the PCA (Figure 1a). If such an issue occurred, we would also expect to see far more DEGs than reported in this study as well as higher pairwise *F*_*ST*_ between H × H and *N* × N fish across all SNPs (here *F*_*ST*_ = 0.0087). In fact, many confounding explanations would be likely to affect all four cross types equally. One caveat is that we unfortunately do not have any data on the run timing of each parent in the study. In the Siletz River, natural‐origin and first‐generation hatchery fish have overlapping run times (Figure S4), and both hatchery and natural‐origin fish were collected in March and April (Table S1) such that it is unlikely, but not impossible, that the two types of fish were sampled at different times. It is conceivable that one type of fish was held longer in captivity before spawning, on average, than the other and the time held before spawning could influence egg quality. However, that possibility would manifest as a maternal effect of fish type, which we ruled out as an explanation. Alternately, if there was a difference in run‐timing between that natural and hatchery origin fish, then that could conceivably cause their offspring to differ at multiple genes. In that case, the conclusion that the natural and hatchery‐origin fish differ genetically would still be true, but the timing of when selection occurred would differ (e.g., the adults collected for broodstock versus juveniles in the hatchery). Arguing against that hypothesis, a re‐examination of hatchery and natural origin steelhead from the Hood River (for which we did have data on parental run date) revealed no differences in gene expression owing to run‐timing despite nearly identical empirical results to those of this study (Figure S5). Furthermore, we isolated all SNPS within our VCF file for SNPs found within *GREB1L*, *ROCK1*, and *LRCC9*, genes known to influence run‐timing in *Oncorhynchus* species (Hess et al. 2016; Koch and Narum 2020; Barry et al. 2024). No SNPs were found in the latter two genes. A total of 47 SNPs were found in *GREB1L*. After plotting among‐individual genetic distances at these *GREB1L* SNPs using PCA, we found no differences among the four groups of fish (Figure S6).

Although the simplest explanation for expression differences between H × H and *N* × N offspring is additive genetic change in loci under selection, heritable epigenetic effects may also play a role (Le Luyer et al. 2017; Venney et al. 2021). If the differences in environments lead to trans‐generational epigenetic changes that subsequently change gene expression, then this represents a different mechanism than genetic adaptation (e.g., selection on cis or trans regulatory elements). Nevertheless, recent genetics studies have illustrated that trans‐generational epigenetic effects may be more rare than previously appreciated (Fitz‐James and Cavalli 2022). Intergenerational epigenetic effects are more common but are typically induced via simultaneous maternal exposure to the parent, it's F1 offspring, and the germline of the F1 offspring (Fitz‐James and Cavalli 2022). If such a mechanism were occurring in this system, then we would expect to see patterns of gene expression consistent with maternal effects, which we do not (Figure 3). From a broader perspective, the mechanism does matter because the rates at which captive ancestry can be purged from a natural population may depend, at least to some extent, on the mode of inheritance; future studies are needed both in salmonids and in the field of trans‐generational epigenetics in general (Bošković and Rando 2018; Anastasiadi et al. 2021; Lamka et al. 2022). Nevertheless, given the rapid genetic adaptation to hatcheries that occurs in steelhead (Christie, Marine, French, Waples, and Blouin 2012), it seems reasonable to conclude that at least some of the changes in gene expression observed here are a part of an adaptive response to the hatchery environment.

## Funding

This work was funded by the Bonneville Power Administration (project #2003‐054‐00) and the Oregon Department of Fish and Wildlife (Project: Collaborative Research, Education and Outreach at the Oregon Hatchery Research Center).

## Conflicts of Interest

The authors declare no conflicts of interest.

## Supporting information


**Figure S1:** Examination of potential paternal effects in the Siletz River offspring (see Figure 3 for maternal effects). To test for these effects, we split the 385 main effects DEGs into those that were upregulated in hatchery HxH offspring (*n* = 290; panels A, B) and those that were upregulated in natural‐origin NxN offspring (*n* = 95; panels C, D). (A) Conceptual illustration of predicted effects: if paternal effects were driving the main effects, we would expect to see similar mean normalized gene counts between offspring that share a father (HxH vs. NxH and HxN vs. NxN, where maternal environment is indicated first) (dark gray circles). Alternatively, if paternal effects are not driving the main effects, then the standardized gene counts should be additive (light gray circles). (B) Empirical mean of the standardized log gene counts (± standard error) for the DEGs illustrating that the mean gene counts are remarkably additive and not driven by paternal effects. (C, D) Same as (A, B), but for the main effect DEGs that were upregulated in NxN offspring.
**Figure S2:** Standardized log gene counts (standardized to the mean log H × H offspring gene count) for all DEGs to illustrate the effect for every gene, which again illustrates that most main effects DEGs are not driven by maternal or paternal effects. (A) DEGs that are upregulated in HxH offspring and (B) DEGs that are upregulated in NxN offspring.
**Figure S3:** All pairwise relatedness values between all offspring from the Hatchery × Hatchery (H × H) (A) and Natural‐origin × Natural‐origin (NxN) crosses (B). Using the SNPs described in the main text, we calculated pairwise relatedness using vcftools and the ‐‐relatedness2 flag, which calculates a relatedness statistic based on the method of Manichaikul et al. (2010). The purple vertical line illustrates the mean and the orange vertical line illustrates the median (overlapping in A). Notice that there were no differences in relatedness between the offspring of HxH and NxN fish.
**Figure S4:** Examination of the effect of parent run‐timing for Siletz River winter‐run steelhead. Over a 10‐year period (1997 to 2006) there were no substantial differences in the run‐timing of adults. The adults used in our study were collected in 2008. Figure reproduced with permission.
**Figure S5:** Although no data exist on the run‐timing for the Siletz River fish used in this study, we were able to re‐examine the effect of run‐timing for Hood River steelhead on the 723 genes differentially expressed between the NxN and HxH offspring. Examination of the effect of parent run‐timing (expressed as an ordinal date on the x‐axis) on standardized gene counts of the differentially expressed genes for the fathers and mothers of hatchery x hatchery (HxH) crosses (A, B) and the fathers and mothers of natural‐origin offspring (NxN) crosses (C, D) for the Hood River. There were no differences in mean and 95% confidence intervals of the standardized gene counts through time illustrating that the parental run timing had little to no effect on the expression of genes that were differentially expressed between HxH and NxN offspring. Although no such data exists for the Siletz River fish, these data suggest that parent run‐timing may also have a small effect on the DEGs identified between HxH and NxN Siletz River offspring.
**Figure S6:** Principal component analyses of among‐individual genetic distances for 47 SNPs found within the *GREB1L* gene isolated from the VCF file created from F1 offspring (see Methods). The *GREB1L* gene has been previously confirmed to play a large role in run‐timing of steelhead. Among the groups of parents, if there were differences in run‐timing that were driven by this gene, then we would expect to see differences in these SNPs. Instead, we see no substantial differences in the spread or location of individuals. We removed two individuals that had greater than 30% missing data (one individual had 71% missing data at this gene). Mean *F*_*ST*_ between NxN and HxH at all 47 SNPs was 0.0081, which is not higher than the genome‐wide mean of 0.0087.

**Table S1:** Crossing dates and number of specific crosses used in the experiment. Four complete matrices were created on March 17, March 24, April 3, and three complete matrices were created on April 8. The total number of crosses was equal to 60 (4 cross types per matrix × 15 full matrices) and we sequenced 2 offspring per cross (1 male and 1 female) resulting in a total sample size of 120 individuals. Full details for all offspring can be found in Table S2; see Figure 1 for an illustration of the crossing matrices.
**Table S2:** Sample information for all offspring sequenced in this study. Sample information includes the sample name (Name); cross type (Cross) where the mother is listed first and where N equals natural‐origin and H equals hatchery‐origin; Family is an alpha‐numeric code where the number represents the specific crossing matrix (see Figure 1) and the letter represents the cross type from that particular matrix (A = H × H, B = N × H, C = N × N, D = H × N); the date the cross was performed (Cross Date); the genetic sex of the sequenced offspring (Sex); the sequencing group (all sequenced on the same instrument as part of the same sequencing group); and the total number of reads mapped (Mapped) back to the reference genome.
**Table S3:** Summary of information for all 385 genes differentially expressed (DEG) between N × N and H × H offspring, sorted by log2 fold change (log FC). Here, the N × N offspring were used as the reference such that a negative log fold change means the gene was upregulated in the H × H offspring (downregulated in the N × N offspring) and a positive log fold change means the gene was down regulated in the HxH offspring (upregulated in the N × N offspring). Also included are the gene ID (prior to annotation), standard error associated with the log2 fold change (lfcSE), the test statistic for significance testing provided by DESeq2 (stat), the unadjusted *p*‐value (*p*‐value) and the FDR corrected *p*‐value (padj). All genes with *p*_adj_ ≤ 0.05 were retained.
**Table S4:** Gene names and annotations for DEGs that could be successfully annotated by EGGNOG. The full table, which includes all GO terms is available as an extra supplementary file (extra Table S1). Here we include the Query sequence, the *e*‐value, the preferred gene name (if available), and a description of the gene.
**Table S5:** Table of all the GO terms identified in the DEGs between the N × N and H × H offspring. The table is sorted by the count (number of occurrences) of each GO term within the annotated DEG dataset. Included is the GO ID, the corresponding name of the GO ID, the number of occurrences (Count) and the output from REVIGO including the Log Size, Frequency, Uniqueness, Dispensability, and PC coordinates used to create Figure 4. All GO terms associated with the DEGs are available in Extra Table S1.
**Table S6:** GO terms associated with the genes differentially expressed between natural‐origin offspring and hatchery‐origin offspring and reduced according to REVIGO. This table complements the terms highlighted in Figure 4 and lists all GO terms from each k.means cluster where each row indicates the HEX colors used to delineate each cluster, Count represents the number distinct DEGs that contain a particular GO term, and the GO term description itself.

## Data Availability

All sequence data are available at the NCBI Sequence Read Archive: Accession Numbers: PRJNA1201192 (https://www.ncbi.nlm.nih.gov/bioproject/PRJNA1201192). All code used in the project is hosted at GitHub https://github.com/ChristieLab/steelhead_siletz_RNAseq.

## References

[eva70213-bib-0001] Allendorf, F. W. , G. H. Thorgaard , and B. Turner . 1984. “Evolutionary Genetics of Fishes.” In Tetraploidy and the Evolution of Salmonid Fishes, 55–93. Springer US.

[eva70213-bib-0003] Anastasiadi, D. , C. J. Venney , L. Bernatchez , and M. Wellenreuther . 2021. “Epigenetic Inheritance and Reproductive Mode in Plants and Animals.” Trends in Ecology & Evolution 36, no. 12: 1124–1140. 10.1016/j.tree.2021.08.006. 34489118

[eva70213-bib-0004] Araki, H. , W. R. Ardren , E. Olsen , B. Cooper , and M. S. Blouin . 2007. “Reproductive Success of Captive‐Bred Steelhead Trout in the Wild: Evaluation of Three Hatchery Programs in the Hood River.” Conservation Biology 21, no. 1: 181–190. 10.1111/j.1523-1739.2006.00564.x. 17298524

[eva70213-bib-0005] Araki, H. , B. Cooper , and M. S. Blouin . 2007. “Genetic Effects of Captive Breeding Cause a Rapid, Cumulative Fitness Decline in the Wild.” Science 318, no. 5847: 100–103. 10.1126/science.1145621. 17916734

[eva70213-bib-0006] Araki, H. , and C. Schmid . 2010. “Is Hatchery Stocking a Help or Harm?: Evidence, Limitations and Future Directions in Ecological and Genetic Surveys.” Aquaculture 308: S2–S11. 10.1016/j.aquaculture.2010.05.036.

[eva70213-bib-0007] Backenstose, N. J. C. , A. M. Nalesnik , M. K. Bui , I. I. Ciubotariu , C. L. Searle , and M. R. Christie . 2026. “Delayed Transcriptional Response of *Daphnia pulex* to Thermal Stress.” G3 (Bethesda, Md.) 16, no. 3: p.jkaf311. 10.1093/g3journal/jkaf311 PMC12958815 41589072

[eva70213-bib-0008] Barry, P. D. , D. A. Tallmon , N. S. Howe , et al. 2024. “A Major Effect Locus Involved in Migration Timing Is Shared by Pink and Sockeye Salmon.” bioRxiv: The Preprint Server for Biology. 10.1101/2024.03.30.587279.

[eva70213-bib-0009] Berthelot, C. , F. Brunet , D. Chalopin , et al. 2014. “The Rainbow Trout Genome Provides Novel Insights Into Evolution After Whole‐Genome Duplication in Vertebrates.” Nature Communications 5: 10. 10.1038/ncomms4657. PMC4071752 24755649

[eva70213-bib-0010] Blouin, M. S. , M. C. Wrey , S. R. Bollmann , J. C. Skaar , R. G. Twibell , and C. Fuentes . 2021. “Offspring of First‐Generation Hatchery Steelhead Trout (*Oncorhynchus Mykiss*) Grow Faster in the Hatchery Than Offspring of Wild Fish, but Survive Worse in the Wild: Possible Mechanisms for Inadvertent Domestication and Fitness Loss in Hatchery Salmon.” PLoS One 16, no. 12: 12. 10.1371/journal.pone.0257407. PMC8675725 34914737

[eva70213-bib-0011] Bošković, A. , and O. J. Rando . 2018. “Transgenerational Epigenetic Inheritance.” Annual Review of Genetics 52, no. 1: 21–41. 10.1146/annurev-genet-120417-031404 30160987

[eva70213-bib-0012] Bowler, M. T. , H. M. Buchanan‐Smith , and A. Whiten . 2012. “Assessing Public Engagement With Science in a University Primate Research Centre in a National Zoo.” PLoS One 7, no. 4: e34505. 22496822 10.1371/journal.pone.0034505 PMC3319593

[eva70213-bib-0013] Brichieri‐Colombi, T. A. , N. A. Lloyd , J. M. McPherson , and A. Moehrenschlager . 2019. “Limited Contributions of Released Animals From Zoos to North American Conservation Translocations.” Conservation Biology 33, no. 1: 33–39. 10.1111/cobi.13160. 29923231 PMC7380022

[eva70213-bib-0014] Brunelli, J. P. , K. J. Wertzler , K. Sundin , and G. H. Thorgaard . 2008. “Y‐Specific Sequences and Polymorphisms in Rainbow Trout and Chinook Salmon.” Genome 51, no. 9: 739–748. 10.1139/g08-060. 18772952

[eva70213-bib-0015] Bryant, E. H. , and D. H. Reed . 1999. “Fitness Decline Under Relaxed Selection in Captive Populations.” Conservation Biology 13, no. 3: 665–669. 10.1046/j.1523-1739.1999.97518.x.

[eva70213-bib-0016] Butchart, S. H. M. , M. Walpole , B. Collen , et al. 2010. “Global Biodiversity: Indicators of Recent Declines.” Science 328, no. 5982: 1164–1168. 10.1126/science.1187512. 20430971

[eva70213-bib-0017] Christie, M. R. , M. J. Ford , and M. S. Blouin . 2014. “On the Reproductive Success of Early‐Generation Hatchery Fish in the Wild.” Evolutionary Applications 7, no. 8: 883–896. 10.1111/eva.12183. 25469167 PMC4211718

[eva70213-bib-0018] Christie, M. R. , R. A. French , M. L. Marine , and M. S. Blouin . 2014. “How Much Does Inbreeding Contribute to the Reduced Fitness of Hatchery‐Born Steelhead ( *Oncorhynchus mykiss* ) in the Wild?” Journal of Heredity 105, no. 1: 111–119. 10.1093/jhered/est076. 24187426

[eva70213-bib-0019] Christie, M. R. , M. L. Marine , S. E. Fox , R. A. French , and M. S. Blouin . 2016. “A Single Generation of Domestication Heritably Alters the Expression of Hundreds of Genes.” Nature Communications 7: 6. 10.1038/ncomms10676. PMC4757788 26883375

[eva70213-bib-0020] Christie, M. R. , M. L. Marine , R. A. French , and M. S. Blouin . 2012. “Genetic Adaptation to Captivity Can Occur in a Single Generation.” Proceedings of the National Academy of Sciences of the United States of America 109, no. 1: 238–242. 10.1073/pnas.1111073109. 22184236 PMC3252900

[eva70213-bib-0021] Christie, M. R. , M. L. Marine , R. A. French , R. S. Waples , and M. S. Blouin . 2012. “Effective Size of a Wild Salmonid Population Is Greatly Reduced by Hatchery Supplementation.” Heredity 109, no. 4: 254–260. 10.1038/hdy.2012.39. 22805657 PMC3464026

[eva70213-bib-0022] Cowx, I. G. , R. Arlinghaus , and S. J. Cooke . 2010. “Harmonizing Recreational Fisheries and Conservation Objectives for Aquatic Biodiversity in Inland Waters.” Journal of Fish Biology 76, no. 9: 2194–2215. 10.1111/j.1095-8649.2010.02686.x. 20557659

[eva70213-bib-0023] Crates, R. , D. Stojanovic , and R. Heinsohn . 2023. “The Phenotypic Costs of Captivity.” Biological Reviews 98, no. 2: 434–449. 10.1111/brv.12913. 36341701

[eva70213-bib-0024] Danecek, P. , A. Auton , G. Abecasis , et al. 2011. “The Variant Call Format and VCFtools.” Bioinformatics 27, no. 15: 2156–2158. 10.1093/bioinformatics/btr330. 21653522 PMC3137218

[eva70213-bib-0025] Farquharson, K. A. , C. J. Hogg , and C. E. Grueber . 2021. “Offspring Survival Changes Over Generations of Captive Breeding.” Nature Communications 12, no. 1: 3045. 10.1038/s41467-021-22631-0. PMC8144597 34031378

[eva70213-bib-0026] Fisch, K. M. , C. C. Kozfkay , J. A. Ivy , O. A. Ryder , and R. S. Waples . 2015. “Fish Hatchery Genetic Management Techniques: Integrating Theory With Implementation.” North American Journal of Aquaculture 77, no. 3: 343–357. 10.1080/15222055.2014.999846.

[eva70213-bib-0027] Fitz‐James, M. H. , and G. Cavalli . 2022. “Molecular Mechanisms of Transgenerational Epigenetic Inheritance.” Nature Reviews Genetics 23, no. 6: 325–341. 10.1038/s41576-021-00438-5. PMC7619059 34983971

[eva70213-bib-0028] Fox, S. E. , M. R. Christie , M. Marine , H. D. Priest , T. C. Mockler , and M. S. Blouin . 2014. “Sequencing and Characterization of the Anadromous Steelhead ( *Oncorhynchus mykiss* ) Transcriptome.” Marine Genomics 15: 13–15. 10.1016/j.margen.2013.12.001. 24440488

[eva70213-bib-0029] Frankham, R. 2008. “Genetic Adaptation to Captivity in Species Conservation Programs.” Molecular Ecology 17, no. 1: 325–333. 10.1111/j.1365-294X.2007.03399.x. 18173504

[eva70213-bib-0030] Frankham, R. , J. D. Ballou , and D. A. Briscoe . 2010. Introduction to Conservation Genetics. Cambridge university press.

[eva70213-bib-0031] Fraser, D. J. , L. K. Weir , L. Bernatchez , M. M. Hansen , and E. B. Taylor . 2011. “Extent and Scale of Local Adaptation in Salmonid Fishes: Review and Meta‐Analysis.” Heredity 106, no. 3: 404–420. 10.1038/hdy.2010.167. 21224881 PMC3131967

[eva70213-bib-0032] Gao, G. , S. Magadan , G. C. Waldbieser , et al. 2021. “A Long Reads‐Based De‐Novo Assembly of the Genome of the Arlee Homozygous Line Reveals Chromosomal Rearrangements in Rainbow Trout.” G3: Genes, Genomes, Genetics 11, no. 4: jkab052. 10.1093/g3journal/jkab052. 33616628 PMC8763230

[eva70213-bib-0033] Gering, E. , D. Incorvaia , R. Henriksen , D. Wright , and T. Getty . 2019. “Maladaptation in Feral and Domesticated Animals.” Evolutionary Applications 12, no. 7: 1274–1286. 10.1111/eva.12784. 31417614 PMC6691326

[eva70213-bib-0034] Harder, A. M. , and M. R. Christie . 2022. “Genomic Signatures of Adaptation to Novel Environments: Hatchery and Life History‐Associated Loci in Landlocked and Anadromous Atlantic Salmon ( *Salmo salar* ).” Canadian Journal of Fisheries and Aquatic Sciences 79, no. 5: 761–770.

[eva70213-bib-0035] Hartigan, J. A. , and M. A. Wong . 1979. “Algorithm AS 136: A K‐Means Clustering Algorithm.” Applied Statistics 28: 100–108. 10.2307/2346830.

[eva70213-bib-0036] Hendry, A. P. , J. K. Wenburg , P. Bentzen , E. C. Volk , and T. P. Quinn . 2000. “Rapid Evolution of Reproductive Isolation in the Wild: Evidence From Introduced Salmon.” Science 290, no. 5491: 516–518. 10.1126/science.290.5491.516. 11039932

[eva70213-bib-0037] Hess, J. E. , J. S. Zendt , A. R. Matala , and S. R. Narum . 2016. “Genetic Basis of Adult Migration Timing in Anadromous Steelhead Discovered Through Multivariate Association Testing.” Proceedings of the Royal Society B: Biological Sciences 283, no. 1830: 20153064. 10.1098/rspb.2015.3064 PMC4874702 27170720

[eva70213-bib-0038] Houde, A. L. , C. A. Black , C. C. Wilson , T. E. Pitcher , and B. D. Neff . 2015. “Genetic and Maternal Effects on Juvenile Survival and Fitness‐Related Traits in Three Populations of Atlantic Salmon.” Canadian Journal of Fisheries and Aquatic Sciences 72, no. 5: 751–758.

[eva70213-bib-0039] Howe, N. S. , M. C. Hale , C. D. Waters , S. M. Schaal , K. R. Shedd , and W. A. Larson . 2024. “Genomic Evidence for Domestication Selection in Three Hatchery Populations of Chinook Salmon, *Oncorhynchus Tshawytscha* .” Evolutionary Applications 17, no. 2: e13656. 38357359 10.1111/eva.13656 PMC10866082

[eva70213-bib-0040] Huerta‐Cepas, J. , D. Szklarczyk , D. Heller , et al. 2019. “eggNOG 5.0: A Hierarchical, Functionally and Phylogenetically Annotated Orthology Resource Based on 5090 Organisms and 2502 Viruses.” Nucleic Acids Research 47, no. D1: D309–D314. 10.1093/nar/gky1085. 30418610 PMC6324079

[eva70213-bib-0041] IUCN Conservation Planning Specialist Group . 2020. IUCN Red List Captive Breeding Recommendations.

[eva70213-bib-0042] Kassambara, A. , and F. Mundt . 2020. “_factoextra: Extract and Visualize the Results of Multivariate Data Analyses_. R Package Version 1.0.7.”

[eva70213-bib-0043] Koch, I. J. , and S. R. Narum . 2020. “Validation and Association of Candidate Markers for Adult Migration Timing and Fitness in Chinook Salmon.” Evolutionary Applications 13, no. 9: 2316–2332. 33005226 10.1111/eva.13026 PMC7513726

[eva70213-bib-0044] Lamka, G. F. , A. M. Harder , M. Sundaram , et al. 2022. “Epigenetics in Ecology, Evolution, and Conservation.” Frontiers in Ecology and Evolution 10: 871791. 10.3389/fevo.2022.871791.

[eva70213-bib-0045] Le Luyer, J. , M. Laporte , T. D. Beacham , et al. 2017. “Parallel Epigenetic Modifications Induced by Hatchery Rearing in a Pacific Salmon.” Proceedings of the National Academy of Sciences of the United States of America 114, no. 49: 12964–12969. 10.1073/pnas.1711229114. 29162695 PMC5724268

[eva70213-bib-0046] Lee, A. , B. N. Daniels , W. Hemstrom , et al. 2025. “Genetic Adaptation Despite High Gene Flow in a Range‐Expanding Population.” Molecular Ecology 34, no. 15: e17511. 39215560 10.1111/mec.17511 PMC11868467

[eva70213-bib-0047] Leitwein, M. , K. Wellband , H. Cayuela , et al. 2022. “Strong Parallel Differential Gene Expression Induced by Hatchery Rearing Weakly Associated With Methylation Signals in Adult Coho Salmon (*O. Kisutch*).” Genome Biology and Evolution 14, no. 4: evac036. 35276004 10.1093/gbe/evac036 PMC8995047

[eva70213-bib-0048] Lien, S. , B. F. Koop , S. R. Sandve , et al. 2016. “The Atlantic Salmon Genome Provides Insights Into Rediploidization.” Nature 533, no. 7602: 200. 10.1038/nature17164. 27088604 PMC8127823

[eva70213-bib-0049] Love, M. I. , W. Huber , and S. Anders . 2014. “Moderated Estimation of Fold Change and Dispersion for RNA‐Seq Data With DESeq2.” Genome Biology 15, no. 12: 38. 10.1186/s13059-014-0550-8. PMC4302049 25516281

[eva70213-bib-0050] Manichaikul, A. , J. C. Mychaleckyj , S. S. Rich , K. Daly , M. Sale , and W. M. Chen . 2010. “Robust Relationship Inference in Genome‐Wide Association Studies.” Bioinformatics 26, no. 22: 2867–2873. 10.1093/bioinformatics/btq559. 20926424 PMC3025716

[eva70213-bib-0051] Matos, M. 2012. “Maternal Effects Can Inflate Rate of Adaptation to Captivity.” Proceedings of the National Academy of Sciences 109, no. 36: E2380. 10.1073/pnas.1202193109 PMC3437868 22930821

[eva70213-bib-0052] Milot, E. , C. Perrier , L. Papillon , J. J. Dodson , and L. Bernatchez . 2013. “Reduced Fitness of Atlantic Salmon Released in the Wild After One Generation of Captive Breeding.” Evolutionary Applications 6, no. 3: 472–485. 10.1111/eva.12028. 23745139 PMC3673475

[eva70213-bib-0053] NPAFC . 2023. “NPAFC Statistics: Description of Pacific Salmonid Catch and Hatchery Release Data Files (Updated 24 July 2023) North Pacific Anadromous Fish Commission.” https://www.npafc.org/statistics/.

[eva70213-bib-0054] O'Sullivan, R. J. , T. Aykanat , S. E. Johnston , et al. 2020. “Captive‐Bred Atlantic Salmon Released Into the Wild Have Fewer Offspring Than Wild‐Bred Fish and Decrease Population Productivity.” Proceedings of the Royal Society B: Biological Sciences 287, no. 1937: 20201671. 10.1098/rspb.2020.1671. PMC7661298 33081620

[eva70213-bib-0056] Pearse, D. E. , S. A. Hayes , M. H. Bond , et al. 2009. “Over the Falls? Rapid Evolution of Ecotypic Differentiation in Steelhead/Rainbow Trout ( *Oncorhynchus mykiss* ).” Journal of Heredity 100, no. 5: 515–525. 10.1093/jhered/esp040. 19561050

[eva70213-bib-0057] Quinlan, A. R. , and I. M. Hall . 2010. “BEDTools: A Flexible Suite of Utilities for Comparing Genomic Features.” Bioinformatics 26, no. 6: 841–842. 10.1093/bioinformatics/btq033. 20110278 PMC2832824

[eva70213-bib-0058] Quinn, T. P. 2018. The Behavior and Ecology of Pacific Salmon and Trout. University of Washington Press.

[eva70213-bib-0059] Quinn, T. P. , M. T. Kinnison , and M. J. Unwin . 2001. “Evolution of Chinook Salmon (*Oncorhynchus Tshawytscha*) Populations in New Zealand: Pattern, Rate, and Process.” Genetica 112: 493–513. 11838785

[eva70213-bib-0060] Reebs, S. G. 2002. “Plasticity of Diel and Circadian Activity Rhythms in Fishes.” Reviews in Fish Biology and Fisheries 12, no. 4: 349–371. 10.1023/a:1025371804611.

[eva70213-bib-0061] Reisenbichler, R. , S. Rubin , L. Wetzel , and S. Phelps . 2004. “Natural Selection After Release From a Hatchery Leads to Domestication in Steelhead, *Oncorhynchus mykiss* .” Stock Enhancement and Sea Ranching: Developments, Pitfalls and Opportunities, edited by K. M. Leber , S. Kitada , H. L. Blankenship , and T. Svåsand , 371–384. Wiley‐Blackwell.

[eva70213-bib-0062] Ritchie, M. E. , B. Phipson , D. Wu , et al. 2015. “Limma Powers Differential Expression Analyses for RNA‐Sequencing and Microarray Studies.” Nucleic Acids Research 43, no. 7: 13. 10.1093/nar/gkv007. 25605792 PMC4402510

[eva70213-bib-0063] Ruggerone, G. T. , R. M. Peterman , B. Dorner , and K. W. Myers . 2010. “Magnitude and Trends in Abundance of Hatchery and Wild Pink Salmon, Chum Salmon, and Sockeye Salmon in the North Pacific Ocean.” Marine and Coastal Fisheries 2, no. 1: 306–328. 10.1577/c09-054.1.

[eva70213-bib-0064] Seddon, P. J. , D. P. Armstrong , and R. F. Maloney . 2007. “Developing the Science of Reintroduction Biology.” Conservation Biology 21, no. 2: 303–312. 10.1111/j.1523-1739.2006.00627.x. 17391180

[eva70213-bib-0065] Smyth, G. K. 2005. “Limma: Linear Models for Microarray Data.” In Bioinformatics and Computational Biology Solution Using R and Bioconductor, edited by R. Gentalman , V. J. Carey , W. Huber , R. A. Irizarry , and S. Dudoit , 397–420. Springer.

[eva70213-bib-0066] Sparks, M. M. , J. C. Kraft , K. M. S. Blackstone , G. G. McNickle , and M. R. Christie . 2022. “Large Genetic Divergence Underpins Cryptic Local Adaptation Across Ecological and Evolutionary Gradients.” Proceedings of the Royal Society B: Biological Sciences 289, no. 1984: 20221472. 10.1098/rspb.2022.1472. PMC9533007 36196546

[eva70213-bib-0067] Supek, F. , M. Bošnjak , N. Škunca , and T. Šmuc . 2011. “REVIGO Summarizes and Visualizes Long Lists of Gene Ontology Terms.” PLoS One 6, no. 7: e21800. 10.1371/journal.pone.0021800. 21789182 PMC3138752

[eva70213-bib-0068] Team, R. C . 2024. R: A Language and Environment for Statistical Computing. R Foundation for Statistical Computing. https://www.R‐project.org/.

[eva70213-bib-0069] Truby, N. L. , R. K. Kim , G. M. Silva , et al. 2024. “A Zinc Finger Transcription Factor Enables Social Behaviors While Controlling Transposable Elements and Immune Response in Prefrontal Cortex.” Translational Psychiatry 14, no. 1: 1–12. 38272911 10.1038/s41398-024-02775-5 PMC10810849

[eva70213-bib-0070] Venney, C. J. , K. W. Wellband , and D. D. Heath . 2021. “Rearing Environment Affects the Genetic Architecture and Plasticity of DNA Methylation in Chinook Salmon.” Heredity 126: 38–49. 32699390 10.1038/s41437-020-0346-4 PMC7852867

[eva70213-bib-0071] Wasser, S. K. , L. Brown , C. Mailand , et al. 2015. “Genetic Assignment of Large Seizures of Elephant Ivory Reveals Africa's Major Poaching Hotspots.” Science 349, no. 6243: 84–87. 10.1126/science.aaa2457. 26089357 PMC5535781

[eva70213-bib-0072] Watson, R. , I. Baste , A. Larigauderie , et al. 2019. Summary for Policymakers of the Global Assessment Report on Biodiversity and Ecosystem Services of the Intergovernmental Science‐Policy Platform on Biodiversity and Ecosystem Services, 22–47. IPBES Secretariat.

[eva70213-bib-0073] Wen, J. , F. Jiang , Y. Weng , et al. 2019. “Identification of Heat‐Tolerance QTLs and High‐Temperature Stress‐Responsive Genes Through Conventional QTL Mapping, QTL‐Seq and RNA‐Seq in Tomato.” BMC Plant Biology 19: 398. 31510927 10.1186/s12870-019-2008-3 PMC6739936

[eva70213-bib-0074] Whitehouse, J. , B. M. Waller , M. Chanvin , et al. 2014. “Evaluation of Public Engagement Activities to Promote Science in a Zoo Environment.” PLoS One 9, no. 11: e113395. 25415193 10.1371/journal.pone.0113395 PMC4240600

[eva70213-bib-0075] Willoughby, J. R. , and M. R. Christie . 2019. “Long‐Term Demographic and Genetic Effects of Releasing Captive‐Born Individuals Into the Wild.” Conservation Biology 33, no. 2: 377–388. 10.1111/cobi.13217. 30168872

[eva70213-bib-0076] Willoughby, J. R. , A. M. Harder , J. A. Tennessen , K. T. Scribner , and M. R. Christie . 2018. “Rapid Genetic Adaptation to a Novel Environment Despite a Genome‐Wide Reduction in Genetic Diversity.” Molecular Ecology 27, no. 20: 4041–4051. 10.1111/mec.14726. 29802799

[eva70213-bib-0077] Wilson, D. 2008. “Siletz Basin Steelhead Trapping and Management Activities. Oregon Department of Fish and Wildlife.” https://library.state.or.us/repository/2008/200805091147105/index.pdf.

[eva70213-bib-0078] Wolf, J. B. , and M. J. Wade . 2009. “What Are Maternal Effects (And What Are They Not)?” Philosophical Transactions of the Royal Society, B: Biological Sciences 364, no. 1520: 1107–1115. 10.1098/rstb.2008.0238. PMC2666680 19324615

