# Supplementary material for: Heritable Genetic Effects Caused by a Single Generation of Captive Breeding
Source: Evol Appl. 2026 Mar 11;19(3):e70213. doi: 10.1111/eva.70213 (PMC13093589; doi:10.1111/eva.70213)
Supplement: Supplementary file 1 — Figure S1: Examination of potential paternal effects in the Siletz River offspring (see Figure 3 for maternal effects). To test for these effects, we split the 385 main effects DEGs into those that were upregulated in hatchery HxH offspring (n = 290; panels A, B) and those that were upregulated in natural‐origin NxN offspring (n = 95; panels C, D). (A) Conceptual illustration of predicted effects: if paternal effects were driving the main effects, we would expect to see similar mean normalized gene counts between offspring that share a father (HxH vs. NxH and HxN vs. NxN, where maternal environment is indicated first) (dark gray circles). Alternatively, if paternal effects are not driving the main effects, then the standardized gene counts should be additive (light gray circles). (B) Empirical mean of the standardized log gene counts (± standard error) for the DEGs illustrating that the mean gene counts are remarkably additive and not driven by paternal effects. (C, D) Same as (A, B), but for the main effect DEGs that were upregulated in NxN offspring. Figure S2: Standardized log gene counts (standardized to the mean log H × H offspring gene count) for all DEGs to illustrate the effect for every gene, which again illustrates that most main effects DEGs are not driven by maternal or paternal effects. (A) DEGs that are upregulated in HxH offspring and (B) DEGs that are upregulated in NxN offspring. Figure S3: All pairwise relatedness values between all offspring from the Hatchery × Hatchery (H × H) (A) and Natural‐origin × Natural‐origin (NxN) crosses (B). Using the SNPs described in the main text, we calculated pairwise relatedness using vcftools and the ‐‐relatedness2 flag, which calculates a relatedness statistic based on the method of Manichaikul et al. (2010). The purple vertical line illustrates the mean and the orange vertical line illustrates the median (overlapping in A). Notice that there were no differences in relatedness between the offspring of HxH and Nx [file EVA-19-e70213-s002.docx]

**Supplementary Materials for: Heritable genetic effects caused by a single generation of captive breeding**

## **Supplementary Materials and Methods**

### *Sample information and experimental design*

Adults were collected and crossed in 2008. All fish were winter-run Siletz River steelhead, caught at the Siletz Falls Trap at river mile 64.5. The Oregon Department of Fish and Wildlife maintains a broodstock program in which only wild-origin fish are caught and brought to the Alsea falls hatchery for creation of the F1 first-generation hatchery fish for subsequent release into the Siletz. All hatchery-origin fish that are caught in the trap are removed or recycled downstream with the exception of fish that were included in this study. The total number of fish caught in the trap ranged from 314 to 1,422 between 1994 and 2007, with roughly equal numbers of first-generation hatchery and wild-origin individuals captured each year (Wilson, 2008). Unfortunately, we do not have the run dates for each of the parents that were collected for us in 2008, so we cannot test whether run date correlates with any pattern of gene expression in this dataset. However, winter-run hatchery and natural-origin steelhead in the Siletz River have very similar distributions of run timing (Wilson, 2008), and our request at the time was to collect equal numbers of each type of fish each week for the purpose of making 2x2 matrices; we were able to sequence two offspring from each cross type from all fifteen 2x2 matrices (Fig. 1, Table S2).

### *Sequencing alignment:*

Reads were first processed with Trimmomatic Version 0.30 (Bolger, Lohse, & Usadel, 2014) using the parameter flags PE -phred33 -leading 20 -trailing 20 -slidingwindow -5:20 minlen -50. We next aligned our reads to the rainbow trout reference genome to *O. mykiss* genome USDA_OmykA_1.1 (GCF_013265735.2; Gao et al., 2021) using HISAT2.1.0 (Kim, Langmead, & Salzberg, 2015) with the following parameters: --downstream-transcriptome-assembly --time --threads 20. We used samtools (Li et al., 2009) to sort and create BAM files from our SAM files and then used STRINGTIE 2.0.3 (Pertea et al., 2015) to create individual GTF files using the BAM files and the best available rainbow trout GTF file guide assembly (available here: <https://www.ncbi.nlm.nih.gov/genome/196?genome_assembly_id=319782>). We next used the default STRINGTIE parameters to merge all transcripts from our samples with the reference annotation. All samples (single and paired end) were used in the merge list to create a single merged GTF file. Lastly, we used FEATURECOUNTS in SUBREAD 1.6.1 (Liao, Smyth, & Shi, 2014) to create a transcript count matrix with our merged GTF file and sorted BAM files. For paired-end reads we excluded chimeric fragments (-C flag) and required that both reads be successfully mapped (-B flag). We employed the default FEATURECOUNTS settings, which does not count single reads that aligned to multiple locations in the reference or read pairs that overlap multiple features.

### *SNP Calling:*

With trimmed RNA-seq reads, we called SNPs with the variant discovery pipeline provided by the Genome Analysis Toolkit (GATK 3.8) (McKenna et al., 2010). We first called SNPs following the joint genotyping workflow for all n=120 samples simultaneously (Brouard, Schenkel, Marete, & Bissonnette, 2019). We started by mapping RNA-seq reads to the steelhead (*Oncorhynchus mykiss*) genome following the STAR 2-pass alignment steps. We set --outFilterMultimapNmax to 1, --outSJfilterReads to Unique, --alignEndsType to EndToEnd, --chimMainSegmentMultNmax to 1, --limitGenomeGenerateRAM to 250000000000, --sjdbOverhang to 99 or 149, --runThreadN to 10 and --limitSjdbInsertNsj to 25000000. All other parameters were set to default values (McKenna et al., 2010). By adding read groups, sorting, marking duplicates and creating indices, we obtained BAM files from SAM files generated by the STAR 2-pass alignment steps. After generating BAM files, we applied the GATK tool, SplitNCigarReads, to BAM files, to split reads into exon segments and cut sequences extending to intronic regions. Next, we called SNPs using the GATK tool, HaplotypeCaller, in which we set --genotyping_mode to DISCOVERY, --emitRefConfidence to GVCF, --variant_index_type to LINEAR, --variant_index_parameter to 128000, -pairHMM to VECTOR_LOGLESS_CACHING, -ploidy to 2 and -maxAltAlleles to 100. Lastly, we performed joint genotyping using the GATK tool, GenotypeGVCFs, with --max_alternate_alleles set to 100 and removed all indels. To validate SNPs called from the GATK joint genotyping workflow with RNA-seq reads, we additionally called SNPs following the RNA-seq variant calling pipeline. The differences between the joint genotyping workflow and the RNA-seq variant calling pipeline begin with the application of HaplotypeCaller. In comparison to the joint genotyping workflow, we supplied one sample to HaplotypeCaller at a time when applying the RNA-seq variant calling pipeline, in which we picked the option -dontUseSoftClippedBases and set -stand_call_conf to 20.0 and -maxAltAlleles to 100. Finally, we filtered variants using the GATK tool, VariantFiltration, by applying filters “FS > 30.0” and “QD < 2.0”, and removed indels. We used the SNPs that were identified in both the GATK joint genotyping workflow and the RNA-seq pipeline in all subsequent analyses.

### References

Araki, H., Ardren, W. R., Olsen, E., Cooper, B., & Blouin, M. S. (2007). Reproductive success of captive-bred steelhead trout in the wild: Evaluation of three hatchery programs in the Hood River. *Conservation Biology, 21*(1), 181-190. doi:10.1111/j.1523-1739.2006.00564.x

Araki, H., Cooper, B., & Blouin, M. S. (2007). Genetic effects of captive breeding cause a rapid, cumulative fitness decline in the wild. *Science, 318*(5847), 100-103. doi:10.1126/science.1145621

Berthelot, C., Brunet, F., Chalopin, D., Juanchich, A., Bernard, M., Noel, B., . . . Guiguen, Y. (2014). The rainbow trout genome provides novel insights into evolution after whole-genome duplication in vertebrates. *Nature Communications, 5*, 10. doi:10.1038/ncomms4657

Bolger, A. M., Lohse, M., & Usadel, B. (2014). Trimmomatic: a flexible trimmer for Illumina sequence data. *Bioinformatics, 30*(15), 2114-2120.

Brouard, J.-S., Schenkel, F., Marete, A., & Bissonnette, N. (2019). The GATK joint genotyping workflow is appropriate for calling variants in RNA-seq experiments. *Journal of animal science and biotechnology, 10*(1), 1-6.

Christie, M. R., Ford, M. J., & Blouin, M. S. (2014). On the reproductive success of early-generation hatchery fish in the wild. *Evolutionary Applications, 7*(8), 883-896. doi:10.1111/eva.12183

Christie, M. R., Marine, M. L., Fox, S. E., French, R. A., & Blouin, M. S. (2016). A single generation of domestication heritably alters the expression of hundreds of genes. *Nature Communications, 7*, 6. doi:10.1038/ncomms10676

Fox, S. E., Christie, M. R., Marine, M., Priest, H. D., Mockler, T. C., & Blouin, M. S. (2014). Sequencing and characterization of the anadromous steelhead (Oncorhynchus mykiss) transcriptome. *Marine Genomics, 15*, 13-15. doi:10.1016/j.margen.2013.12.001

Good, T. P., Waples, R. S., & Adams, P. B. (2005). Updated status of federally listed ESUs of West Coast salmon and steelhead.

Kim, D., Langmead, B., & Salzberg, S. L. (2015). HISAT: a fast spliced aligner with low memory requirements. *Nature methods, 12*(4), 357-360.

Li, H., Handsaker, B., Wysoker, A., Fennell, T., Ruan, J., Homer, N., . . . Durbin, R. (2009). The sequence alignment/map format and SAMtools. *Bioinformatics, 25*(16), 2078-2079.

Liao, Y., Smyth, G. K., & Shi, W. (2014). featureCounts: an efficient general purpose program for assigning sequence reads to genomic features. *Bioinformatics, 30*(7), 923-930.

Manichaikul, A., Mychaleckyj, J. C., Rich, S. S., Daly, K., Sale, M., & Chen, W. M. (2010). Robust relationship inference in genome-wide association studies. *Bioinformatics, 26*(22), 2867-2873. doi:10.1093/bioinformatics/btq559

McKenna, A., Hanna, M., Banks, E., Sivachenko, A., Cibulskis, K., Kernytsky, A., . . . Daly, M. (2010). The Genome Analysis Toolkit: a MapReduce framework for analyzing next-generation DNA sequencing data. *Genome research, 20*(9), 1297-1303.

Pearse, D. E., Barson, N. J., Nome, T., Gao, G., Campbell, M. A., Abadía-Cardoso, A., . . . Naish, K. A. (2019). Sex-dependent dominance maintains migration supergene in rainbow trout. *Nature ecology & evolution, 3*(12), 1731-1742.

Pertea, M., Pertea, G. M., Antonescu, C. M., Chang, T.-C., Mendell, J. T., & Salzberg, S. L. (2015). StringTie enables improved reconstruction of a transcriptome from RNA-seq reads. *Nature biotechnology, 33*(3), 290-295.

Reebs, S. G. (2002). Plasticity of diel and circadian activity rhythms in fishes. *Reviews in Fish Biology and Fisheries, 12*(4), 349-371. doi:10.1023/a:1025371804611

Wilson, D. (2008). *Siletz Basin Steelhead Trapping and Management Activities*: Oregon Department of Fish and Wildlife. Wilson, D. (2008) Siletz basin steelhead trapping and management activities. Available at <https://library.state.or.us/repository/2008/200805091147105/index.pdf>


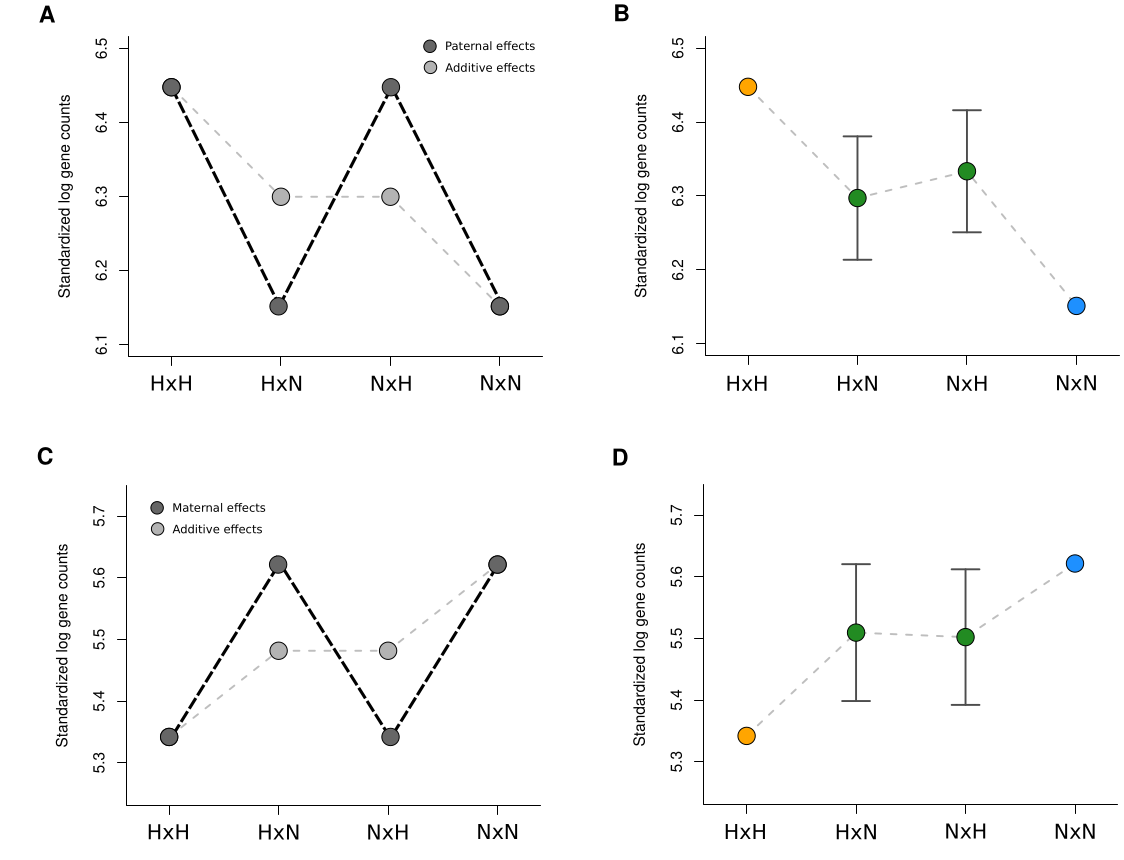
**Figure S1:** Examination of potential paternal effects in the Siletz River offspring (see Fig. 3 for maternal effects). To test for these effects, we split the 385 main effects DEGs into those that were upregulated in hatchery HxH offspring (n=290; panels A-B) and those that were upregulated in natural-origin NxN offspring (n=95; panels C-D). A.) Conceptual illustration of predicted effects: if paternal effects were driving the main effects, we would expect to see similar mean normalized gene counts between offspring that share a father (HxH vs. NxH and HxN vs. NxN, where maternal environment is indicated first) (dark gray circles). Alternatively, if paternal effects are not driving the main effects, then the standardized gene counts should be additive (light gray circles). B.) Empirical mean of the standardized log gene counts (± standard error) for the DEGs illustrating that the mean gene counts are remarkably additive and not driven by paternal effects. C-D.) Same as A-B, but for the main effect DEGs that were upregulated in NxN offspring.

**
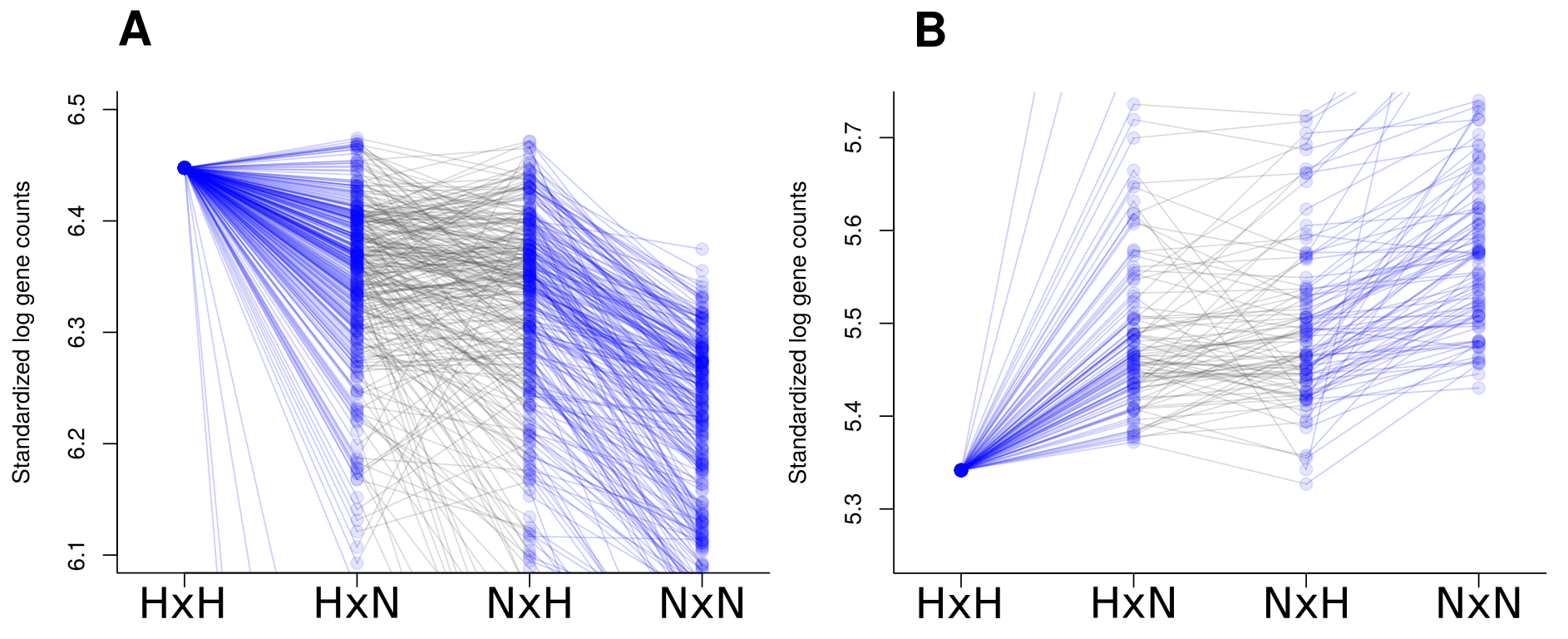
**

**Figure S2:** Standardized log gene counts (standardized to the mean log H$\times$H offspring gene count) for all DEGs to illustrate the effect for every gene, which again illustrates that most main effects DEGs are not driven by maternal or paternal effects. **A.)** DEGs that are upregulated in HxH offspring and **B.)** DEGs that are upregulated in NxN offspring


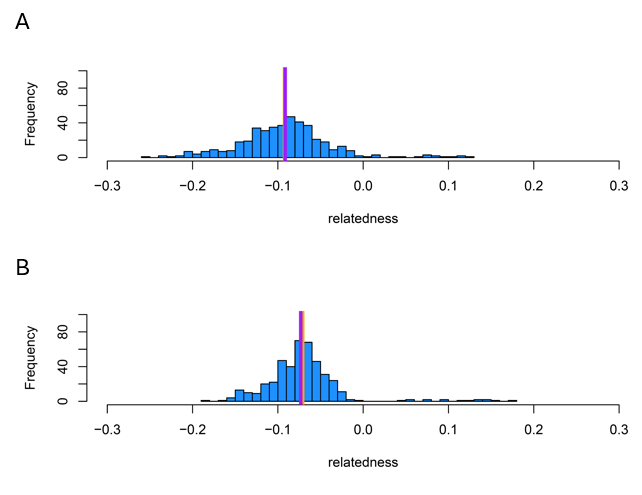


**Figure S3:** All pairwise relatedness values between all offspring from the Hatchery x Hatchery (HxH) (A) and Natural-origin x Natural-origin (NxN) crosses (B). Using the SNPs described in the main text, we calculated pairwise relatedness using vcftools and the --relatedness2 flag, which calculates a relatedness statistic based on the method of Manichaikul et al. (2010). The purple vertical line illustrates the mean and the orange vertical line illustrates the median (overlapping in A). Notice that there were no differences in relatedness between the offspring of HxH and NxN fish.

##

**
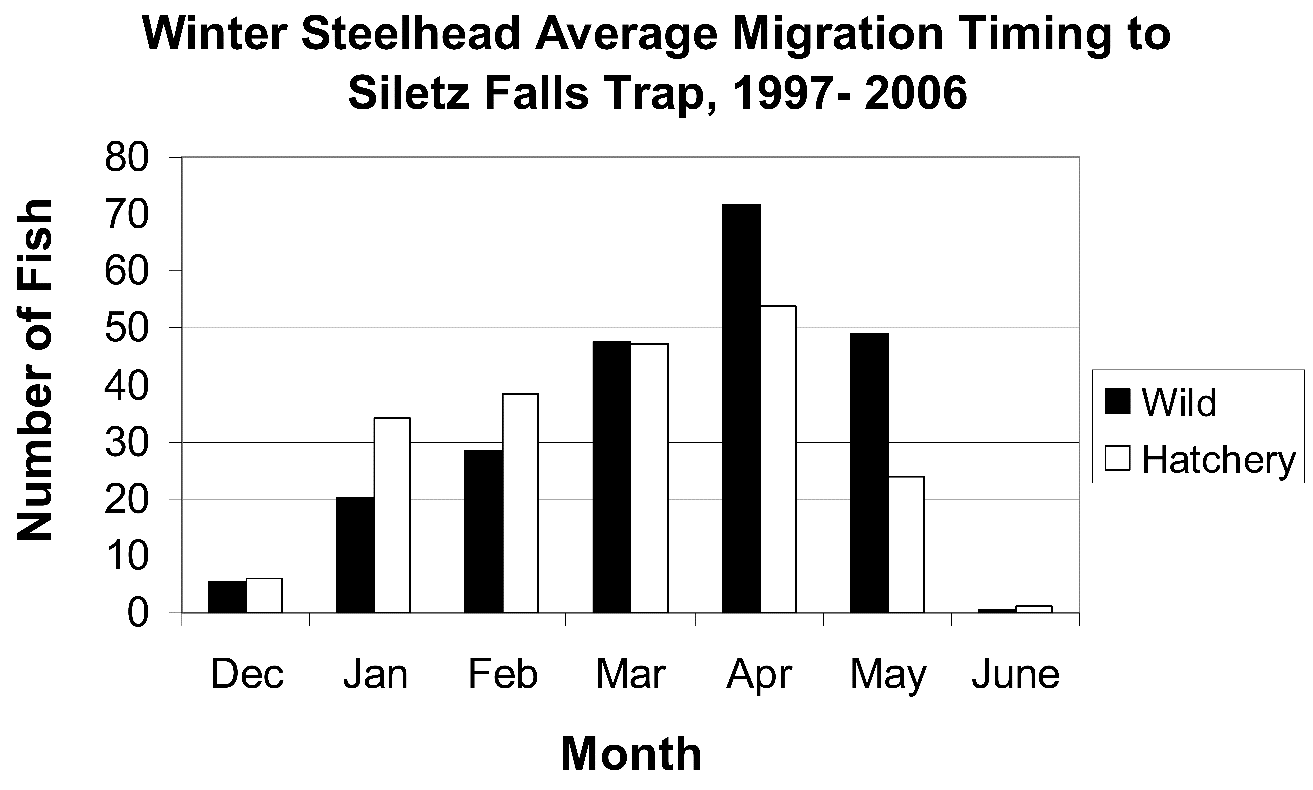
**

**Figure S4:** Examination of the effect of parent run-timing for Siletz River winter-run steelhead. Over a ten-year period (1997 to 2006) there were no substantial differences in the run-timing of adults. The adults used in our study were collected in 2008. Figure reproduced with permission.





NxN fathers

NxN fathers

NxN mothers

HxH fathers

HxH fathers

**Figure S5:** Although no data exist on the run-timing for the Siletz River fish used in this study, we were able to re-examine the effect of run-timing for Hood River steelhead on the 723 genes differentially expressed between the NxN and HxH offspring. Examination of the effect of parent run-timing (expressed as an ordinal date on the x-axis) on standardized gene counts of the differentially expressed genes for the fathers and mothers of hatchery x hatchery (HxH) crosses (A,B) and the fathers and mothers of natural-origin offspring (NxN) crosses (C,D) for the Hood River. There were no differences in mean and 95% confidence intervals of the standardized gene counts through time illustrating that the parental run timing had little to no effect on the expression of genes that were differentially expressed between HxH and NxN offspring. Although no such data exists for the Siletz River fish, these data suggest that parent run-timing may also have a small effect on the DEGs identified between HxH and NxN Siletz River offspring.


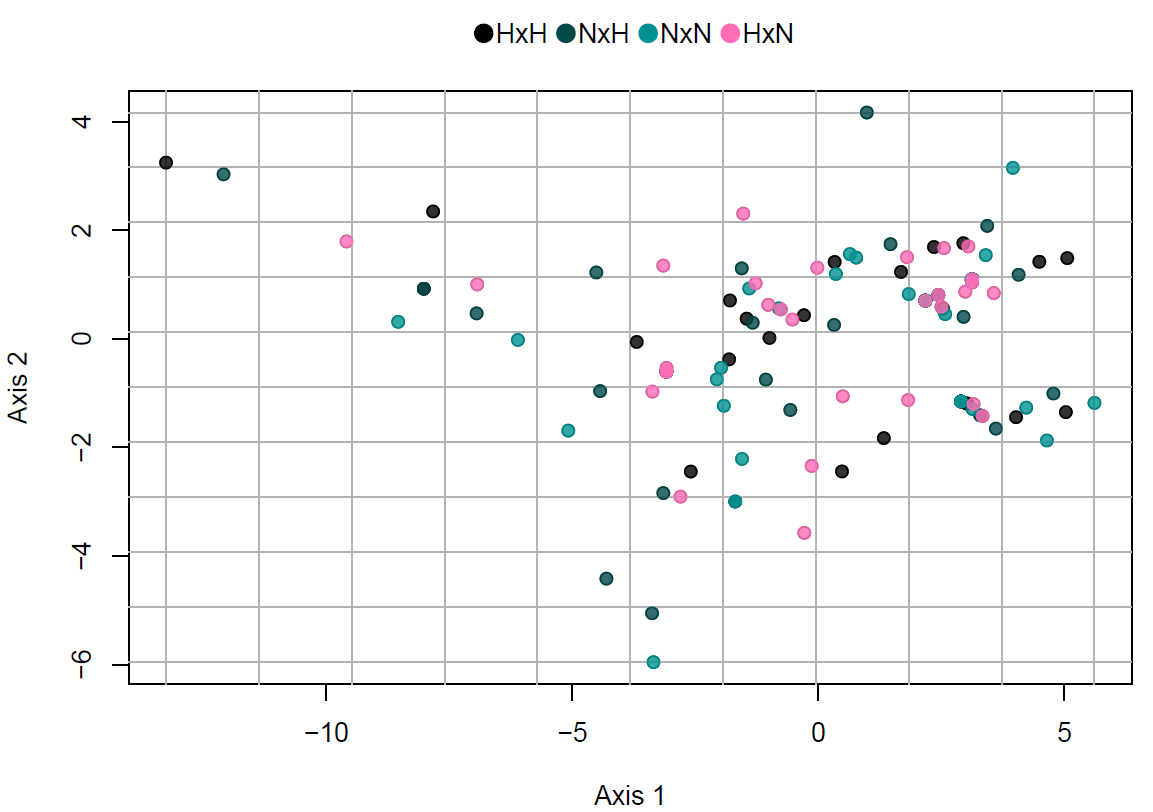


**Figure S6:** Principal component analyses of among-individual genetic distances for 47 SNPs found within the *GREB1L* gene isolated from the VCF file created from F1 offspring (see Methods). The *GREB1L* gene has been previously confirmed to play a large role in run-timing of steelhead. Among the groups of parents, if there were differences in run-timing that were driven by this gene, then we would expect to see differences in these SNPs. Instead, we see no substantial differences in the spread or location of individuals. We removed two individuals that had greater than 30% missing data (one individual had 71% missing data at this gene). Mean *F_ST_* between NxN and HxH at all 47 SNPs was 0.0081, which is not higher than the genome-wide mean of 0.0087.


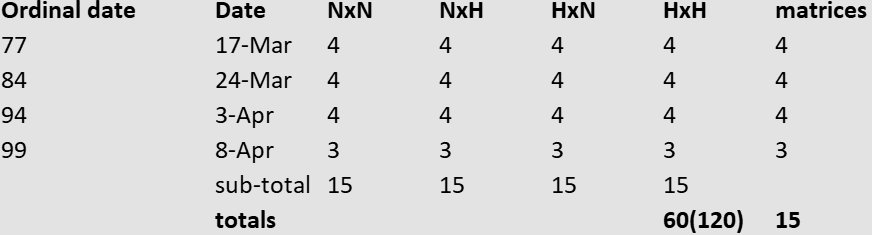


Table S1: Crossing dates and number of specific crosses used in the experiment. Four complete matrices were created on March 17, March 24, April 3, and three complete matrices were created on April 8. The total number of crosses was equal to 60 (4 cross types per matrix x 15 full matrices) and we sequenced 2 offspring per cross (1 male and 1 female) resulting in a total sample size of 120 individuals. Full details for all offspring can be found in Table S2; see Figure 1 for an illustration of the crossing matrices.

**Table S2:** Sample information for all offspring sequenced in this study. Sample information includes the sample name (Name); cross type (Cross) where the mother is listed first and where N equals natural-origin and H equals hatchery-origin; Family is an alpha-numeric code where the number represents the specific crossing matrix (see Fig. 1) and the letter represents the cross type from that particular matrix (A=HxH, B= NxH, C=NxN, D= HxN); the date the cross was performed (Cross Date); the genetic sex of the sequenced offspring (Sex); the sequencing group (all sequenced on the same instrument as part of the same sequencing group); and the total number of reads mapped (Mapped) back to the reference genome.

| **Name** | **Cross** | **Family** | **Cross Date** | **Sex** | **Sequencing Group** | **Mapped** |
| --- | --- | --- | --- | --- | --- | --- |
| s001 | HxH | 1A | 3/17/2008 | male | Group 1 (paired end 150bp) | 17979116 |
| s002 | HxH | 10A | 4/3/2008 | female | Group 1 (paired end 150bp) | 24697921 |
| s003 | HxH | 1A | 3/17/2008 | female | Group 1 (paired end 150bp) | 16332641 |
| s004 | HxH | 10A | 4/3/2008 | male | Group 1 (paired end 150bp) | 11338893 |
| s005 | NxH | 10B | 4/3/2008 | male | Group 1 (paired end 150bp) | 19329066 |
| s006 | NxH | 1B | 3/17/2008 | male | Group 1 (paired end 150bp) | 15978871 |
| s007 | NxH | 10B | 4/3/2008 | female | Group 1 (paired end 150bp) | 10083751 |
| s008 | NxH | 1B | 3/17/2008 | female | Group 1 (paired end 150bp) | 16593328 |
| s009 | NxN | 10C | 4/3/2008 | male | Group 1 (paired end 150bp) | 6916116 |
| s010 | NxN | 1C | 3/17/2008 | female | Group 1 (paired end 150bp) | 24962955 |
| s011 | NxN | 10C | 4/3/2008 | female | Group 1 (paired end 150bp) | 11800691 |
| s012 | NxN | 1C | 3/17/2008 | male | Group 1 (paired end 150bp) | 18673683 |
| s013 | HxN | 10D | 4/3/2008 | female | Group 1 (paired end 150bp) | 69574323 |
| s014 | HxN | 1D | 3/17/2008 | female | Group 1 (paired end 150bp) | 15618393 |
| s015 | HxN | 10D | 4/3/2008 | male | Group 1 (paired end 150bp) | 9115339 |
| s016 | HxN | 1D | 3/17/2008 | male | Group 1 (paired end 150bp) | 15881561 |
| s017 | HxH | 11A | 4/3/2008 | male | Group 1 (paired end 150bp) | 15654011 |
| s018 | HxH | 2A | 3/17/2008 | female | Group 1 (paired end 150bp) | 13923388 |
| s019 | HxH | 2A | 3/17/2008 | male | Group 1 (paired end 150bp) | 18608815 |
| s020 | HxH | 11A | 4/3/2008 | female | Group 1 (paired end 150bp) | 8484694 |
| s021 | NxH | 2B | 3/17/2008 | female | Group 1 (paired end 150bp) | 13960479 |
| s022 | NxH | 11B | 4/3/2008 | male | Group 1 (paired end 150bp) | 10521234 |
| s023 | NxH | 2B | 3/17/2008 | male | Group 1 (paired end 150bp) | 14204987 |
| s024 | NxH | 11B | 4/3/2008 | female | Group 1 (paired end 150bp) | 8304875 |
| s025 | NxN | 2C | 3/17/2008 | male | Group 1 (paired end 150bp) | 19445376 |
| s026 | NxN | 11C | 4/3/2008 | female | Group 1 (paired end 150bp) | 31713954 |
| s027 | NxN | 2C | 3/17/2008 | female | Group 1 (paired end 150bp) | 18077242 |
| s028 | NxN | 11C | 4/3/2008 | male | Group 1 (paired end 150bp) | 16777905 |
| s029 | HxN | 2D | 3/17/2008 | male | Group 1 (paired end 150bp) | 20490606 |
| s030 | HxN | 11D | 4/3/2008 | male | Group 1 (paired end 150bp) | 18664062 |
| s031 | HxN | 2D | 3/17/2008 | female | Group 1 (paired end 150bp) | 18701904 |
| s032 | HxN | 11D | 4/3/2008 | female | Group 1 (paired end 150bp) | 15343987 |
| s033 | HxH | 3A | 3/17/2008 | male | Group 1 (paired end 150bp) | 13185371 |
| s034 | HxH | 12A | 4/3/2008 | female | Group 1 (paired end 150bp) | 11628332 |
| s035 | HxH | 3A | 3/17/2008 | female | Group 1 (paired end 150bp) | 15431398 |
| s036 | HxH | 12A | 4/3/2008 | male | Group 1 (paired end 150bp) | 10072361 |
| s037 | NxH | 3B | 3/17/2008 | male | Group 1 (paired end 150bp) | 18219710 |
| s038 | NxH | 12B | 4/3/2008 | male | Group 1 (paired end 150bp) | 11440774 |
| s039 | NxH | 3B | 3/17/2008 | female | Group 1 (paired end 150bp) | 16119555 |
| s040 | NxH | 12B | 4/3/2008 | female | Group 1 (paired end 150bp) | 37701309 |
| s041 | NxN | 3C | 3/17/2008 | female | Group 1 (paired end 150bp) | 13808664 |
| s042 | NxN | 12C | 4/3/2008 | male | Group 1 (paired end 150bp) | 16705715 |
| s043 | NxN | 3C | 3/17/2008 | male | Group 1 (paired end 150bp) | 15735107 |
| s044 | NxN | 12C | 4/3/2008 | female | Group 1 (paired end 150bp) | 14122704 |
| s045 | HxN | 3D | 3/17/2008 | female | Group 1 (paired end 150bp) | 16004185 |
| s046 | HxN | 12D | 4/3/2008 | male | Group 1 (paired end 150bp) | 12046278 |
| s047 | HxN | 3D | 3/17/2008 | male | Group 1 (paired end 150bp) | 15406252 |
| s048 | HxN | 12D | 4/3/2008 | female | Group 1 (paired end 150bp) | 14685951 |
| s049 | HxH | 13A | 4/8/2008 | female | Group 1 (paired end 150bp) | 24590141 |
| s050 | HxH | 4A | 3/17/2008 | female | Group 1 (paired end 150bp) | 17519337 |
| s051 | HxH | 13A | 4/8/2008 | male | Group 1 (paired end 150bp) | 17316196 |
| s052 | HxH | 4A | 3/17/2008 | male | Group 1 (paired end 150bp) | 14219886 |
| s053 | NxH | 4B | 3/17/2008 | female | Group 1 (paired end 150bp) | 19124654 |
| s054 | NxH | 13B | 4/8/2008 | female | Group 1 (paired end 150bp) | 23379438 |
| s055 | NxH | 4B | 3/17/2008 | male | Group 1 (paired end 150bp) | 11065048 |
| s056 | NxH | 13B | 4/8/2008 | male | Group 1 (paired end 150bp) | 14066903 |
| s057 | NxN | 4C | 3/17/2008 | female | Group 1 (paired end 150bp) | 17703613 |
| s058 | NxN | 13C | 4/8/2008 | female | Group 1 (paired end 150bp) | 13672921 |
| s059 | NxN | 4C | 3/17/2008 | male | Group 1 (paired end 150bp) | 16953547 |
| s060 | NxN | 13C | 4/8/2008 | male | Group 1 (paired end 150bp) | 15827077 |
| s061 | HxN | 4D | 3/17/2008 | female | Group 1 (paired end 150bp) | 21717087 |
| s062 | HxN | 13D | 4/8/2008 | female | Group 1 (paired end 150bp) | 31036406 |
| s063 | HxN | 4D | 3/17/2008 | male | Group 1 (paired end 150bp) | 19577322 |
| s064 | HxN | 13D | 4/8/2008 | male | Group 1 (paired end 150bp) | 19174637 |
| s065 | HxH | 5A | 3/24/2008 | male | Group 1 (paired end 150bp) | 19247097 |
| s066 | HxH | 14A | 4/8/2008 | female | Group 1 (paired end 150bp) | 14923167 |
| s067 | HxH | 14A | 4/8/2008 | male | Group 1 (paired end 150bp) | 12657045 |
| s068 | HxH | 5A | 3/24/2008 | female | Group 1 (paired end 150bp) | 14050795 |
| s069 | NxH | 14B | 4/8/2008 | female | Group 1 (paired end 150bp) | 11119156 |
| s070 | NxH | 5B | 3/24/2008 | female | Group 1 (paired end 150bp) | 17318375 |
| s071 | NxH | 14B | 4/8/2008 | male | Group 1 (paired end 150bp) | 13216228 |
| s072 | NxH | 5B | 3/24/2008 | male | Group 1 (paired end 150bp) | 16043404 |
| s073 | NxN | 14C | 4/8/2008 | female | Group 1 (paired end 150bp) | 37605203 |
| s074 | NxN | 5C | 3/24/2008 | male | Group 1 (paired end 150bp) | 21182615 |
| s075 | NxN | 14C | 4/8/2008 | male | Group 1 (paired end 150bp) | 14347569 |
| s076 | NxN | 5C | 3/24/2008 | female | Group 1 (paired end 150bp) | 18058223 |
| s077 | HxN | 14D | 4/8/2008 | female | Group 1 (paired end 150bp) | 14874063 |
| s078 | HxN | 5D | 3/24/2008 | female | Group 1 (paired end 150bp) | 17004425 |
| s079 | HxN | 14D | 4/8/2008 | male | Group 1 (paired end 150bp) | 9960253 |
| s080 | HxN | 5D | 3/24/2008 | male | Group 1 (paired end 150bp) | 16543862 |
| s081 | HxH | 15A | 4/8/2008 | female | Group 1 (paired end 150bp) | 9294996 |
| s082 | HxH | 6A | 3/24/2008 | male | Group 1 (paired end 150bp) | 19848742 |
| s083 | HxH | 15A | 4/8/2008 | male | Group 1 (paired end 150bp) | 11858469 |
| s084 | HxH | 6A | 3/24/2008 | female | Group 1 (paired end 150bp) | 14649109 |
| s085 | NxH | 15B | 4/8/2008 | female | Group 1 (paired end 150bp) | 32675445 |
| s086 | NxH | 6B | 3/24/2008 | male | Group 1 (paired end 150bp) | 15709417 |
| s087 | NxH | 15B | 4/8/2008 | male | Group 1 (paired end 150bp) | 11623504 |
| s088 | NxH | 6B | 3/24/2008 | female | Group 1 (paired end 150bp) | 21332784 |
| s089 | NxN | 15C | 4/8/2008 | female | Group 1 (paired end 150bp) | 25375111 |
| s090 | NxN | 6C | 3/24/2008 | male | Group 1 (paired end 150bp) | 13661348 |
| s091 | NxN | 15C | 4/8/2008 | male | Group 1 (paired end 150bp) | 10184718 |
| s092 | NxN | 6C | 3/24/2008 | female | Group 1 (paired end 150bp) | 16502658 |
| s093 | HxN | 15D | 4/8/2008 | female | Group 1 (paired end 150bp) | 11356129 |
| s094 | HxN | 6D | 3/24/2008 | female | Group 1 (paired end 150bp) | 14833327 |
| s095 | HxN | 15D | 4/8/2008 | male | Group 1 (paired end 150bp) | 15507364 |
| s096 | HxN | 6D | 3/24/2008 | male | Group 1 (paired end 150bp) | 12675259 |
| s097 | HxH | 7A | 3/24/2008 | female | Group 1 (paired end 150bp) | 12898953 |
| s098 | HxH | 7A | 3/24/2008 | male | Group 1 (paired end 150bp) | 20361523 |
| s099 | NxH | 7B | 3/24/2008 | female | Group 1 (paired end 150bp) | 16738023 |
| s100 | NxH | 7B | 3/24/2008 | male | Group 1 (paired end 150bp) | 17520516 |
| s101 | NxN | 7C | 3/24/2008 | male | Group 1 (paired end 150bp) | 15608412 |
| s102 | NxN | 7C | 3/24/2008 | female | Group 1 (paired end 150bp) | 19026741 |
| s103 | HxN | 7D | 3/24/2008 | male | Group 1 (paired end 150bp) | 17960983 |
| s104 | HxN | 7D | 3/24/2008 | female | Group 1 (paired end 150bp) | 17301114 |
| s105 | HxH | 8A | 3/24/2008 | female | Group 1 (paired end 150bp) | 12469855 |
| s106 | HxH | 8A | 3/24/2008 | male | Group 1 (paired end 150bp) | 19585387 |
| s107 | NxH | 8B | 3/24/2008 | female | Group 1 (paired end 150bp) | 18865082 |
| s108 | NxH | 8B | 3/24/2008 | male | Group 1 (paired end 150bp) | 17041061 |
| s109 | NxN | 8C | 3/24/2008 | male | Group 1 (paired end 150bp) | 14958138 |
| s110 | NxN | 8C | 3/24/2008 | female | Group 1 (paired end 150bp) | 16405969 |
| s111 | HxN | 8D | 3/24/2008 | male | Group 1 (paired end 150bp) | 17174353 |
| s112 | HxN | 8D | 3/24/2008 | female | Group 1 (paired end 150bp) | 17563125 |
| s113 | HxH | 9A | 4/3/2008 | female | Group 1 (paired end 150bp) | 15006169 |
| s114 | HxH | 9A | 4/3/2008 | male | Group 1 (paired end 150bp) | 17518721 |
| s115 | NxH | 9B | 4/3/2008 | male | Group 1 (paired end 150bp) | 18379825 |
| s116 | NxH | 9B | 4/3/2008 | female | Group 1 (paired end 150bp) | 16039794 |
| s117 | NxN | 9C | 4/3/2008 | male | Group 1 (paired end 150bp) | 13873335 |
| s118 | NxN | 9C | 4/3/2008 | female | Group 1 (paired end 150bp) | 16188382 |
| s119 | HxN | 9D | 4/3/2008 | female | Group 1 (paired end 150bp) | 16394844 |
| s120 | HxN | 9D | 4/3/2008 | male | Group 1 (paired end 150bp) | 16438665 |

**Table S3:** Summary of information for all 385 genes differentially expressed (DEG) between NxN and HxH offspring, sorted by log2 fold change (log FC). Here, the NxN offspring were used as the reference such that a negative log fold change means the gene was upregulated in the HxH offspring (downregulated in the NxN offspring) and a positive log fold change means the gene was down regulated in the HxH offspring (upregulated in the NxN offspring). Also included are the gene ID (prior to annotation), standard error associated with the log2 fold change (lfcSE), the test statistic for significance testing provided by DESeq2 (stat), the unadjusted p-value (p-value) and the FDR corrected p-value (padj). All genes with padj <= 0.05 were retained.

| \| **DEG** \| **Gene ID** \| **logFC** \| **lfcSE** \| **stat** \| **p-value** \| **padj** \| \| --- \| --- \| --- \| --- \| --- \| --- \| --- \| \| 1 \| NA\|MSTRG.94764.1\|NA \| -4.857 \| 0.818 \| -5.937 \| 2.900E-09 \| 0.00008 \| \| 2 \| NA\|MSTRG.96214.1\|NA \| -1.965 \| 0.578 \| -3.403 \| 6.663E-04 \| 0.04958 \| \| 3 \| NA\|MSTRG.54258.1\|NA \| -1.397 \| 0.379 \| -3.689 \| 2.255E-04 \| 0.03136 \| \| 4 \| NA\|MSTRG.3792.1\|NA \| -1.341 \| 0.375 \| -3.576 \| 3.483E-04 \| 0.03746 \| \| 5 \| LOC110521313\|rna-XM_021598796.2\|NA \| -1.254 \| 0.363 \| -3.454 \| 5.515E-04 \| 0.04463 \| \| 6 \| NA\|MSTRG.69572.1\|NA \| -1.143 \| 0.289 \| -3.952 \| 7.750E-05 \| 0.01972 \| \| 7 \| NA\|MSTRG.95423.1\|NA \| -1.095 \| 0.294 \| -3.723 \| 1.969E-04 \| 0.02962 \| \| 8 \| NA\|MSTRG.42473.1\|NA \| -1.082 \| 0.275 \| -3.930 \| 8.480E-05 \| 0.02012 \| \| 9 \| NA\|MSTRG.24795.1\|NA \| -1.080 \| 0.293 \| -3.686 \| 2.280E-04 \| 0.03156 \| \| 10 \| NA\|MSTRG.19808.1\|NA \| -0.981 \| 0.284 \| -3.458 \| 5.434E-04 \| 0.04434 \| \| 11 \| fbxo32\|rna-NM_001193326.1\|NA \| -0.975 \| 0.283 \| -3.439 \| 5.832E-04 \| 0.04574 \| \| 12 \| LOC110490416\|rna-XM_021563799.2\|NA \| -0.935 \| 0.264 \| -3.546 \| 3.913E-04 \| 0.03849 \| \| 13 \| si:zfos-911d5.4\|rna-XM_036973948.1\|NA \| -0.909 \| 0.266 \| -3.421 \| 6.231E-04 \| 0.04793 \| \| 14 \| LOC110529281\|rna-XM_036986815.1\|NA \| -0.905 \| 0.184 \| -4.913 \| 8.960E-07 \| 0.00211 \| \| 15 \| NA\|MSTRG.74358.1\|NA \| -0.773 \| 0.214 \| -3.617 \| 2.981E-04 \| 0.03568 \| \| 16 \| NA\|MSTRG.92805.1\|NA \| -0.760 \| 0.149 \| -5.091 \| 3.560E-07 \| 0.00126 \| \| 17 \| NA\|MSTRG.55792.1\|NA \| -0.754 \| 0.191 \| -3.944 \| 8.000E-05 \| 0.01984 \| \| 18 \| LOC110517154\|rna-XM_036942177.1\|NA \| -0.722 \| 0.187 \| -3.860 \| 1.135E-04 \| 0.02257 \| \| 19 \| NA\|MSTRG.18190.1\|NA \| -0.708 \| 0.180 \| -3.922 \| 8.780E-05 \| 0.02020 \| \| 20 \| LOC110499466\|rna-XM_021576604.2\|NA \| -0.704 \| 0.150 \| -4.705 \| 2.540E-06 \| 0.00422 \| \| 21 \| NA\|MSTRG.4670.1\|NA \| -0.700 \| 0.153 \| -4.585 \| 4.550E-06 \| 0.00523 \| \| 22 \| NA\|MSTRG.152.1\|NA \| -0.679 \| 0.164 \| -4.128 \| 3.650E-05 \| 0.01388 \| \| 23 \| si:ch211-227n13.3\|rna-XM_021590149.2\|NA \| -0.674 \| 0.177 \| -3.805 \| 1.417E-04 \| 0.02497 \| \| 24 \| retreg1\|rna-XM_021612611.2\|NA \| -0.664 \| 0.181 \| -3.666 \| 2.468E-04 \| 0.03236 \| \| 25 \| LOC110496718\|rna-XR_005037537.1\|NA \| -0.660 \| 0.160 \| -4.137 \| 3.510E-05 \| 0.01388 \| \| 26 \| NA\|MSTRG.74345.1\|NA \| -0.639 \| 0.153 \| -4.164 \| 3.130E-05 \| 0.01330 \| \| 27 \| LOC110519057\|rna-XM_036987651.1\|NA \| -0.637 \| 0.183 \| -3.484 \| 4.939E-04 \| 0.04331 \| \| 28 \| NA\|MSTRG.91781.1\|NA \| -0.635 \| 0.173 \| -3.661 \| 2.510E-04 \| 0.03236 \| \| 29 \| mef2b\|rna-XM_021586891.2\|NA \| -0.634 \| 0.162 \| -3.922 \| 8.770E-05 \| 0.02020 \| \| 30 \| LOC110498668\|rna-XM_036954372.1\|NA \| -0.632 \| 0.177 \| -3.562 \| 3.684E-04 \| 0.03769 \| \| 31 \| NA\|MSTRG.48102.1\|NA \| -0.629 \| 0.176 \| -3.580 \| 3.436E-04 \| 0.03746 \| \| 32 \| LOC110485631\|rna-XM_036940236.1\|NA \| -0.621 \| 0.157 \| -3.946 \| 7.940E-05 \| 0.01984 \| \| 33 \| cand2\|rna-XM_036950927.1\|NA \| -0.620 \| 0.137 \| -4.538 \| 5.690E-06 \| 0.00595 \| \| 34 \| NA\|MSTRG.35500.1\|NA \| -0.605 \| 0.126 \| -4.800 \| 1.590E-06 \| 0.00320 \| \| 35 \| NA\|MSTRG.76934.1\|NA \| -0.604 \| 0.153 \| -3.958 \| 7.550E-05 \| 0.01939 \| \| 36 \| LOC110485670\|rna-XM_036949328.1\|NA \| -0.603 \| 0.168 \| -3.593 \| 3.275E-04 \| 0.03684 \| \| 37 \| NA\|MSTRG.65842.1\|NA \| -0.594 \| 0.135 \| -4.420 \| 9.870E-06 \| 0.00876 \| \| 38 \| NA\|MSTRG.58264.1\|NA \| -0.584 \| 0.127 \| -4.581 \| 4.630E-06 \| 0.00523 \| \| 39 \| NA\|MSTRG.69585.1\|NA \| -0.581 \| 0.150 \| -3.867 \| 1.101E-04 \| 0.02233 \| \| 40 \| NA\|MSTRG.17823.1\|NA \| -0.581 \| 0.161 \| -3.614 \| 3.014E-04 \| 0.03572 \| \| 41 \| LOC110486651\|rna-XM_021558411.2\|NA \| -0.580 \| 0.137 \| -4.243 \| 2.200E-05 \| 0.01069 \| \| 42 \| LOC110504779\|rna-XM_021583591.2\|NA \| -0.565 \| 0.137 \| -4.131 \| 3.600E-05 \| 0.01388 \| \| 43 \| NA\|MSTRG.52017.1\|NA \| -0.560 \| 0.144 \| -3.887 \| 1.016E-04 \| 0.02142 \| \| 44 \| NA\|MSTRG.20844.1\|NA \| -0.556 \| 0.141 \| -3.934 \| 8.360E-05 \| 0.02009 \| \| 45 \| LOC110506480\|rna-XM_021586119.2\|NA \| -0.551 \| 0.137 \| -4.030 \| 5.590E-05 \| 0.01692 \| \| 46 \| NA\|MSTRG.12853.1\|NA \| -0.551 \| 0.160 \| -3.440 \| 5.811E-04 \| 0.04574 \| \| 47 \| NA\|MSTRG.94600.1\|NA \| -0.545 \| 0.136 \| -4.003 \| 6.270E-05 \| 0.01748 \| \| 48 \| kbtbd12\|rna-XM_036950176.1\|NA \| -0.535 \| 0.157 \| -3.401 \| 6.721E-04 \| 0.04958 \| \| 49 \| NA\|MSTRG.50932.1\|NA \| -0.534 \| 0.115 \| -4.644 \| 3.420E-06 \| 0.00483 \| \| 50 \| si:ch73-265d7.2\|rna-XM_021578351.2\|NA \| -0.533 \| 0.132 \| -4.028 \| 5.630E-05 \| 0.01692 \| \| 51 \| LOC110527239\|rna-XM_036951334.1\|NA \| -0.528 \| 0.153 \| -3.449 \| 5.616E-04 \| 0.04497 \| \| 52 \| LOC110532039\|rna-XM_021615632.2\|NA \| -0.527 \| 0.132 \| -3.992 \| 6.550E-05 \| 0.01748 \| \| 53 \| LOC118966735\|gene-LOC118966735\|NA \| -0.525 \| 0.153 \| -3.419 \| 6.293E-04 \| 0.04814 \| \| 54 \| NA\|MSTRG.80115.1\|NA \| -0.523 \| 0.125 \| -4.182 \| 2.890E-05 \| 0.01294 \| \| 55 \| LOC110488553\|rna-XM_036942683.1\|NA \| -0.521 \| 0.126 \| -4.126 \| 3.690E-05 \| 0.01388 \| \| 56 \| NA\|MSTRG.61885.1\|NA \| -0.521 \| 0.147 \| -3.549 \| 3.861E-04 \| 0.03840 \| \| 57 \| NA\|MSTRG.58890.1\|NA \| -0.516 \| 0.144 \| -3.587 \| 3.343E-04 \| 0.03701 \| \| 58 \| eif4ebp3\|rna-XM_021617567.2\|NA \| -0.511 \| 0.139 \| -3.669 \| 2.439E-04 \| 0.03236 \| \| 59 \| LOC110504630\|rna-XM_036969936.1\|NA \| -0.506 \| 0.119 \| -4.268 \| 1.980E-05 \| 0.01055 \| \| 60 \| NA\|MSTRG.50060.1\|NA \| -0.506 \| 0.116 \| -4.364 \| 1.280E-05 \| 0.00914 \| \| 61 \| NA\|MSTRG.78558.1\|NA \| -0.500 \| 0.129 \| -3.893 \| 9.890E-05 \| 0.02115 \| \| 62 \| NA\|MSTRG.15072.1\|NA \| -0.499 \| 0.137 \| -3.652 \| 2.599E-04 \| 0.03296 \| \| 63 \| LOC110501849\|rna-XR_005038833.1\|NA \| -0.499 \| 0.114 \| -4.378 \| 1.200E-05 \| 0.00914 \| \| 64 \| NA\|MSTRG.22536.1\|NA \| -0.493 \| 0.099 \| -4.988 \| 6.100E-07 \| 0.00174 \| \| 65 \| NA\|MSTRG.53391.1\|NA \| -0.492 \| 0.121 \| -4.061 \| 4.880E-05 \| 0.01551 \| \| 66 \| LOC110509422\|rna-XM_036968227.1\|NA \| -0.488 \| 0.121 \| -4.016 \| 5.920E-05 \| 0.01723 \| \| 67 \| LOC110529842\|rna-XM_021612434.2\|NA \| -0.487 \| 0.124 \| -3.918 \| 8.950E-05 \| 0.02020 \| \| 68 \| LOC110519808\|rna-XM_036988560.1\|NA \| -0.482 \| 0.139 \| -3.462 \| 5.358E-04 \| 0.04413 \| \| 69 \| NA\|MSTRG.33267.1\|NA \| -0.481 \| 0.089 \| -5.386 \| 7.190E-08 \| 0.00068 \| \| 70 \| LOC110516984\|rna-XR_005042471.1\|NA \| -0.478 \| 0.131 \| -3.638 \| 2.750E-04 \| 0.03376 \| \| 71 \| tfr2\|rna-XM_036958415.1\|NA \| -0.475 \| 0.129 \| -3.671 \| 2.415E-04 \| 0.03236 \| \| 72 \| NA\|MSTRG.20386.1\|NA \| -0.475 \| 0.124 \| -3.826 \| 1.302E-04 \| 0.02359 \| \| 73 \| cisd1\|rna-XM_021576629.2\|NA \| -0.473 \| 0.124 \| -3.822 \| 1.322E-04 \| 0.02378 \| \| 74 \| LOC110532739\|rna-XM_036988808.1\|NA \| -0.469 \| 0.091 \| -5.150 \| 2.600E-07 \| 0.00122 \| \| 75 \| NA\|MSTRG.94628.1\|NA \| -0.466 \| 0.124 \| -3.766 \| 1.660E-04 \| 0.02726 \| \| 76 \| fgl2a\|rna-XM_036945275.1\|NA \| -0.465 \| 0.115 \| -4.025 \| 5.690E-05 \| 0.01692 \| \| 77 \| NA\|MSTRG.4000.1\|NA \| -0.461 \| 0.106 \| -4.344 \| 1.400E-05 \| 0.00914 \| \| 78 \| LOC110507519\|rna-XM_021587561.2\|NA \| -0.460 \| 0.128 \| -3.593 \| 3.270E-04 \| 0.03684 \| \| 79 \| NA\|MSTRG.56851.1\|NA \| -0.459 \| 0.087 \| -5.247 \| 1.550E-07 \| 0.00109 \| \| 80 \| fbxo30a\|rna-XM_021573763.2\|NA \| -0.459 \| 0.127 \| -3.614 \| 3.013E-04 \| 0.03572 \| \| 81 \| LOC110487250\|rna-XM_036949086.1\|NA \| -0.458 \| 0.131 \| -3.485 \| 4.919E-04 \| 0.04326 \| \| 82 \| NA\|MSTRG.61535.1\|NA \| -0.455 \| 0.105 \| -4.336 \| 1.450E-05 \| 0.00914 \| \| 83 \| NA\|MSTRG.73710.1\|NA \| -0.455 \| 0.107 \| -4.254 \| 2.100E-05 \| 0.01055 \| \| 84 \| LOC110500796\|rna-XM_036958315.1\|NA \| -0.455 \| 0.126 \| -3.606 \| 3.104E-04 \| 0.03608 \| \| 85 \| NA\|MSTRG.47396.1\|NA \| -0.452 \| 0.109 \| -4.134 \| 3.560E-05 \| 0.01388 \| \| 86 \| LOC110520289\|rna-XM_021597480.2\|NA \| -0.452 \| 0.129 \| -3.508 \| 4.517E-04 \| 0.04127 \| \| 87 \| NA\|MSTRG.69277.1\|NA \| -0.450 \| 0.122 \| -3.698 \| 2.172E-04 \| 0.03109 \| \| 88 \| NA\|MSTRG.36112.1\|NA \| -0.449 \| 0.122 \| -3.673 \| 2.401E-04 \| 0.03236 \| \| 89 \| LOC110502873\|rna-XM_021581221.2\|NA \| -0.445 \| 0.121 \| -3.673 \| 2.393E-04 \| 0.03236 \| \| 90 \| NA\|MSTRG.26805.1\|NA \| -0.443 \| 0.124 \| -3.564 \| 3.654E-04 \| 0.03769 \| \| 91 \| NA\|MSTRG.63329.1\|NA \| -0.434 \| 0.122 \| -3.572 \| 3.543E-04 \| 0.03746 \| \| 92 \| klhl40a\|rna-XM_021614116.2\|NA \| -0.430 \| 0.112 \| -3.833 \| 1.264E-04 \| 0.02359 \| \| 93 \| NA\|MSTRG.68287.1\|NA \| -0.430 \| 0.100 \| -4.306 \| 1.660E-05 \| 0.00937 \| \| 94 \| LOC110506984\|rna-XM_036963714.1\|NA \| -0.430 \| 0.112 \| -3.845 \| 1.204E-04 \| 0.02344 \| \| 95 \| polr1f\|rna-XM_021559827.2\|NA \| -0.429 \| 0.110 \| -3.908 \| 9.290E-05 \| 0.02024 \| \| 96 \| NA\|MSTRG.4226.1\|NA \| -0.428 \| 0.097 \| -4.395 \| 1.110E-05 \| 0.00901 \| \| 97 \| hsc70\|rna-XM_021624823.2\|NA \| -0.424 \| 0.083 \| -5.104 \| 3.330E-07 \| 0.00126 \| \| 98 \| actr3b\|rna-XM_021562501.2\|NA \| -0.422 \| 0.117 \| -3.617 \| 2.982E-04 \| 0.03568 \| \| 99 \| NA\|MSTRG.80239.1\|NA \| -0.421 \| 0.120 \| -3.511 \| 4.467E-04 \| 0.04103 \| \| 100 \| LOC110520080\|rna-XM_036974364.1\|NA \| -0.419 \| 0.121 \| -3.464 \| 5.328E-04 \| 0.04413 \| \| 101 \| sesn1\|rna-XM_021600702.2\|NA \| -0.414 \| 0.113 \| -3.651 \| 2.615E-04 \| 0.03296 \| \| 102 \| NA\|MSTRG.16443.1\|NA \| -0.405 \| 0.105 \| -3.870 \| 1.089E-04 \| 0.02233 \| \| 103 \| NA\|MSTRG.27949.1\|NA \| -0.403 \| 0.112 \| -3.602 \| 3.152E-04 \| 0.03618 \| \| 104 \| LOC110528303\|rna-XM_021610257.2\|NA \| -0.403 \| 0.117 \| -3.453 \| 5.535E-04 \| 0.04463 \| \| 105 \| tph2\|rna-XM_021576444.2\|NA \| -0.401 \| 0.102 \| -3.919 \| 8.880E-05 \| 0.02020 \| \| 106 \| LOC110487244\|rna-XM_036949088.1\|NA \| -0.398 \| 0.086 \| -4.628 \| 3.690E-06 \| 0.00497 \| \| 107 \| LOC110509620\|rna-XM_021590618.2\|NA \| -0.397 \| 0.089 \| -4.483 \| 7.350E-06 \| 0.00741 \| \| 108 \| LOC110495432\|rna-XM_036982002.1\|NA \| -0.397 \| 0.087 \| -4.545 \| 5.490E-06 \| 0.00595 \| \| 109 \| NA\|MSTRG.76679.1\|NA \| -0.395 \| 0.113 \| -3.495 \| 4.748E-04 \| 0.04283 \| \| 110 \| NA\|MSTRG.29745.1\|NA \| -0.394 \| 0.109 \| -3.627 \| 2.868E-04 \| 0.03463 \| \| 111 \| si:dkey-65j6.2\|rna-XM_036947156.1\|NA \| -0.394 \| 0.080 \| -4.951 \| 7.390E-07 \| 0.00190 \| \| 112 \| LOC110504479\|rna-XM_036970135.1\|NA \| -0.389 \| 0.106 \| -3.651 \| 2.611E-04 \| 0.03296 \| \| 113 \| NA\|MSTRG.71911.1\|NA \| -0.387 \| 0.101 \| -3.837 \| 1.248E-04 \| 0.02359 \| \| 114 \| NA\|MSTRG.73317.1\|NA \| -0.386 \| 0.089 \| -4.350 \| 1.360E-05 \| 0.00914 \| \| 115 \| NA\|MSTRG.40449.1\|NA \| -0.383 \| 0.113 \| -3.399 \| 6.762E-04 \| 0.04958 \| \| 116 \| NA\|MSTRG.89775.1\|NA \| -0.381 \| 0.107 \| -3.563 \| 3.673E-04 \| 0.03769 \| \| 117 \| NA\|MSTRG.82396.1\|NA \| -0.380 \| 0.097 \| -3.921 \| 8.810E-05 \| 0.02020 \| \| 118 \| LOC110526368\|rna-XM_036980723.1\|NA \| -0.378 \| 0.102 \| -3.692 \| 2.225E-04 \| 0.03109 \| \| 119 \| NA\|MSTRG.93824.1\|NA \| -0.378 \| 0.101 \| -3.748 \| 1.784E-04 \| 0.02830 \| \| 120 \| LOC110508796\|rna-XM_021589624.2\|NA \| -0.377 \| 0.106 \| -3.551 \| 3.834E-04 \| 0.03839 \| \| 121 \| LOC110502883\|rna-XM_036960634.1\|NA \| -0.374 \| 0.105 \| -3.578 \| 3.459E-04 \| 0.03746 \| \| 122 \| NA\|MSTRG.71457.1\|NA \| -0.373 \| 0.093 \| -3.992 \| 6.560E-05 \| 0.01748 \| \| 123 \| atp2a1\|rna-XM_036937978.1\|NA \| -0.372 \| 0.103 \| -3.610 \| 3.060E-04 \| 0.03600 \| \| 124 \| LOC110520331\|rna-XM_021597577.2\|NA \| -0.372 \| 0.085 \| -4.366 \| 1.270E-05 \| 0.00914 \| \| 125 \| syt5b\|rna-XM_036946541.1\|NA \| -0.369 \| 0.087 \| -4.222 \| 2.430E-05 \| 0.01122 \| \| 126 \| igdcc4\|rna-XM_036980687.1\|NA \| -0.367 \| 0.096 \| -3.840 \| 1.229E-04 \| 0.02359 \| \| 127 \| cacng6b\|rna-XM_036940887.1\|NA \| -0.367 \| 0.092 \| -3.988 \| 6.670E-05 \| 0.01761 \| \| 128 \| LOC110494170\|rna-XM_036950251.1\|NA \| -0.362 \| 0.098 \| -3.697 \| 2.178E-04 \| 0.03109 \| \| 129 \| NA\|MSTRG.34577.1\|NA \| -0.360 \| 0.104 \| -3.453 \| 5.548E-04 \| 0.04463 \| \| 130 \| NA\|MSTRG.15624.1\|NA \| -0.359 \| 0.078 \| -4.602 \| 4.180E-06 \| 0.00523 \| \| 131 \| NA\|MSTRG.67897.1\|NA \| -0.354 \| 0.094 \| -3.752 \| 1.757E-04 \| 0.02802 \| \| 132 \| LOC110523889\|rna-XM_036977600.1\|NA \| -0.350 \| 0.081 \| -4.321 \| 1.550E-05 \| 0.00914 \| \| 133 \| NA\|MSTRG.25072.1\|NA \| -0.349 \| 0.084 \| -4.167 \| 3.080E-05 \| 0.01330 \| \| 134 \| LOC110537651\|rna-XM_021623857.2\|NA \| -0.348 \| 0.093 \| -3.758 \| 1.710E-04 \| 0.02759 \| \| 135 \| NA\|MSTRG.7011.1\|NA \| -0.348 \| 0.090 \| -3.848 \| 1.192E-04 \| 0.02341 \| \| 136 \| NA\|MSTRG.76362.1\|NA \| -0.346 \| 0.086 \| -4.022 \| 5.770E-05 \| 0.01697 \| \| 137 \| LOC110526049\|rna-XM_021606649.2\|NA \| -0.345 \| 0.094 \| -3.657 \| 2.557E-04 \| 0.03281 \| \| 138 \| NA\|MSTRG.76947.1\|NA \| -0.344 \| 0.096 \| -3.575 \| 3.498E-04 \| 0.03746 \| \| 139 \| LOC110536498\|rna-XM_036936539.1\|NA \| -0.341 \| 0.080 \| -4.251 \| 2.130E-05 \| 0.01055 \| \| 140 \| NA\|MSTRG.64107.1\|NA \| -0.338 \| 0.099 \| -3.401 \| 6.718E-04 \| 0.04958 \| \| 141 \| LOC110496189\|rna-XM_021571936.2\|NA \| -0.335 \| 0.094 \| -3.554 \| 3.788E-04 \| 0.03825 \| \| 142 \| NA\|MSTRG.51902.1\|NA \| -0.334 \| 0.097 \| -3.448 \| 5.652E-04 \| 0.04507 \| \| 143 \| NA\|MSTRG.55724.1\|NA \| -0.334 \| 0.083 \| -4.010 \| 6.080E-05 \| 0.01748 \| \| 144 \| LOC110492188\|gene-LOC110492188\|NA \| -0.328 \| 0.088 \| -3.710 \| 2.073E-04 \| 0.03057 \| \| 145 \| LOC110520799\|rna-XM_036939723.1\|NA \| -0.328 \| 0.090 \| -3.634 \| 2.794E-04 \| 0.03414 \| \| 146 \| NA\|MSTRG.46448.1\|NA \| -0.327 \| 0.094 \| -3.486 \| 4.907E-04 \| 0.04326 \| \| 147 \| NA\|MSTRG.63539.1\|NA \| -0.326 \| 0.093 \| -3.525 \| 4.243E-04 \| 0.04021 \| \| 148 \| NA\|MSTRG.72190.1\|NA \| -0.326 \| 0.096 \| -3.401 \| 6.706E-04 \| 0.04958 \| \| 149 \| NA\|MSTRG.48336.1\|NA \| -0.324 \| 0.088 \| -3.668 \| 2.446E-04 \| 0.03236 \| \| 150 \| NA\|MSTRG.66382.1\|NA \| -0.321 \| 0.090 \| -3.552 \| 3.823E-04 \| 0.03839 \| \| 151 \| NA\|MSTRG.55210.1\|NA \| -0.321 \| 0.093 \| -3.464 \| 5.320E-04 \| 0.04413 \| \| 152 \| NA\|MSTRG.87339.1\|NA \| -0.320 \| 0.093 \| -3.462 \| 5.361E-04 \| 0.04413 \| \| 153 \| NA\|MSTRG.36083.1\|NA \| -0.320 \| 0.086 \| -3.729 \| 1.925E-04 \| 0.02957 \| \| 154 \| NA\|MSTRG.28258.1\|NA \| -0.320 \| 0.092 \| -3.487 \| 4.887E-04 \| 0.04325 \| \| 155 \| NA\|MSTRG.8836.1\|NA \| -0.319 \| 0.084 \| -3.780 \| 1.568E-04 \| 0.02619 \| \| 156 \| NA\|MSTRG.12111.1\|NA \| -0.319 \| 0.078 \| -4.088 \| 4.350E-05 \| 0.01497 \| \| 157 \| NA\|MSTRG.76748.1\|NA \| -0.318 \| 0.092 \| -3.437 \| 5.887E-04 \| 0.04604 \| \| 158 \| LOC110520452\|rna-XM_021597779.2\|NA \| -0.316 \| 0.089 \| -3.547 \| 3.894E-04 \| 0.03844 \| \| 159 \| NA\|MSTRG.1050.1\|NA \| -0.313 \| 0.082 \| -3.826 \| 1.304E-04 \| 0.02359 \| \| 160 \| NA\|MSTRG.84188.1\|NA \| -0.312 \| 0.081 \| -3.865 \| 1.112E-04 \| 0.02233 \| \| 161 \| NA\|MSTRG.31725.1\|NA \| -0.306 \| 0.088 \| -3.467 \| 5.262E-04 \| 0.04413 \| \| 162 \| LOC110509134\|rna-XM_021590107.2\|NA \| -0.304 \| 0.087 \| -3.487 \| 4.885E-04 \| 0.04325 \| \| 163 \| NA\|MSTRG.62036.1\|NA \| -0.303 \| 0.087 \| -3.483 \| 4.965E-04 \| 0.04339 \| \| 164 \| NA\|MSTRG.31107.1\|NA \| -0.303 \| 0.085 \| -3.542 \| 3.975E-04 \| 0.03883 \| \| 165 \| NA\|MSTRG.28342.1\|NA \| -0.300 \| 0.078 \| -3.817 \| 1.349E-04 \| 0.02410 \| \| 166 \| micu3b\|rna-XM_036969588.1\|NA \| -0.299 \| 0.076 \| -3.924 \| 8.700E-05 \| 0.02020 \| \| 167 \| LOC110530527\|rna-XM_021613688.2\|NA \| -0.296 \| 0.085 \| -3.482 \| 4.979E-04 \| 0.04339 \| \| 168 \| NA\|MSTRG.78250.1\|NA \| -0.294 \| 0.063 \| -4.660 \| 3.160E-06 \| 0.00470 \| \| 169 \| LOC110492071\|rna-XM_021566068.2\|NA \| -0.294 \| 0.082 \| -3.563 \| 3.660E-04 \| 0.03769 \| \| 170 \| NA\|MSTRG.80524.1\|NA \| -0.293 \| 0.080 \| -3.641 \| 2.714E-04 \| 0.03370 \| \| 171 \| NA\|MSTRG.62976.1\|NA \| -0.291 \| 0.061 \| -4.774 \| 1.810E-06 \| 0.00320 \| \| 172 \| LOC110527890\|rna-XM_021609481.2\|NA \| -0.288 \| 0.084 \| -3.429 \| 6.064E-04 \| 0.04677 \| \| 173 \| trip10a\|rna-XM_036941095.1\|NA \| -0.287 \| 0.080 \| -3.591 \| 3.296E-04 \| 0.03688 \| \| 174 \| tango6\|rna-XM_021569556.2\|NA \| -0.286 \| 0.077 \| -3.732 \| 1.899E-04 \| 0.02957 \| \| 175 \| LOC110534123\|rna-XM_021618802.2\|NA \| -0.285 \| 0.081 \| -3.531 \| 4.138E-04 \| 0.03960 \| \| 176 \| hspbap1\|rna-XM_036959210.1\|NA \| -0.285 \| 0.082 \| -3.460 \| 5.394E-04 \| 0.04427 \| \| 177 \| NA\|MSTRG.10335.1\|NA \| -0.285 \| 0.082 \| -3.487 \| 4.881E-04 \| 0.04325 \| \| 178 \| NA\|MSTRG.9030.1\|NA \| -0.284 \| 0.072 \| -3.942 \| 8.090E-05 \| 0.01984 \| \| 179 \| klhl31\|rna-XM_021576492.2\|NA \| -0.284 \| 0.074 \| -3.829 \| 1.287E-04 \| 0.02359 \| \| 180 \| LOC110529039\|rna-XM_036984162.1\|NA \| -0.284 \| 0.077 \| -3.682 \| 2.317E-04 \| 0.03190 \| \| 181 \| LOC110503125\|rna-XM_036960572.1\|NA \| -0.283 \| 0.080 \| -3.555 \| 3.774E-04 \| 0.03825 \| \| 182 \| tsc1a\|rna-XM_036980376.1\|NA \| -0.282 \| 0.079 \| -3.575 \| 3.500E-04 \| 0.03746 \| \| 183 \| NA\|MSTRG.19396.1\|NA \| -0.282 \| 0.068 \| -4.158 \| 3.200E-05 \| 0.01330 \| \| 184 \| si:ch211-251b21.1\|rna-XM_021565655.2\|NA \| -0.280 \| 0.072 \| -3.908 \| 9.320E-05 \| 0.02024 \| \| 185 \| LOC110504897\|rna-XM_021583765.2\|NA \| -0.280 \| 0.075 \| -3.724 \| 1.965E-04 \| 0.02962 \| \| 186 \| NA\|MSTRG.62838.1\|NA \| -0.279 \| 0.079 \| -3.549 \| 3.867E-04 \| 0.03840 \| \| 187 \| kcnab2a\|rna-XM_021569439.2\|NA \| -0.278 \| 0.068 \| -4.091 \| 4.300E-05 \| 0.01497 \| \| 188 \| NA\|MSTRG.83976.1\|NA \| -0.277 \| 0.071 \| -3.877 \| 1.058E-04 \| 0.02212 \| \| 189 \| NA\|MSTRG.52609.1\|NA \| -0.277 \| 0.080 \| -3.464 \| 5.323E-04 \| 0.04413 \| \| 190 \| NA\|MSTRG.40857.1\|NA \| -0.274 \| 0.069 \| -3.969 \| 7.220E-05 \| 0.01888 \| \| 191 \| NA\|MSTRG.38152.1\|NA \| -0.273 \| 0.074 \| -3.705 \| 2.116E-04 \| 0.03080 \| \| 192 \| pno1\|rna-XM_021564644.2\|NA \| -0.273 \| 0.076 \| -3.572 \| 3.542E-04 \| 0.03746 \| \| 193 \| NA\|MSTRG.19236.1\|NA \| -0.272 \| 0.061 \| -4.419 \| 9.930E-06 \| 0.00876 \| \| 194 \| igsf9b\|rna-XM_021605895.2\|NA \| -0.271 \| 0.073 \| -3.707 \| 2.099E-04 \| 0.03071 \| \| 195 \| shrprbck1r\|rna-XM_021562478.2\|NA \| -0.270 \| 0.076 \| -3.533 \| 4.102E-04 \| 0.03954 \| \| 196 \| LOC110527932\|rna-XM_036983423.1\|NA \| -0.268 \| 0.071 \| -3.767 \| 1.651E-04 \| 0.02725 \| \| 197 \| NA\|MSTRG.73149.1\|NA \| -0.267 \| 0.079 \| -3.400 \| 6.728E-04 \| 0.04958 \| \| 198 \| LOC110535649\|rna-XM_021620765.2\|NA \| -0.267 \| 0.072 \| -3.694 \| 2.204E-04 \| 0.03109 \| \| 199 \| LOC110506650\|rna-XM_036963621.1\|NA \| -0.266 \| 0.072 \| -3.700 \| 2.158E-04 \| 0.03109 \| \| 200 \| LOC110529642\|rna-XM_036985063.1\|NA \| -0.266 \| 0.071 \| -3.763 \| 1.682E-04 \| 0.02744 \| \| 201 \| NA\|MSTRG.93178.1\|NA \| -0.266 \| 0.073 \| -3.647 \| 2.653E-04 \| 0.03328 \| \| 202 \| NA\|MSTRG.91092.1\|NA \| -0.265 \| 0.076 \| -3.488 \| 4.866E-04 \| 0.04325 \| \| 203 \| tut7\|rna-XM_036936544.1\|NA \| -0.260 \| 0.075 \| -3.479 \| 5.039E-04 \| 0.04364 \| \| 204 \| gmpr\|rna-XM_036953270.1\|NA \| -0.260 \| 0.068 \| -3.829 \| 1.287E-04 \| 0.02359 \| \| 205 \| NA\|MSTRG.84174.1\|NA \| -0.258 \| 0.069 \| -3.739 \| 1.849E-04 \| 0.02900 \| \| 206 \| NA\|MSTRG.15321.1\|NA \| -0.257 \| 0.065 \| -3.964 \| 7.380E-05 \| 0.01912 \| \| 207 \| NA\|MSTRG.7196.1\|NA \| -0.256 \| 0.071 \| -3.627 \| 2.870E-04 \| 0.03463 \| \| 208 \| NA\|MSTRG.34955.1\|NA \| -0.256 \| 0.073 \| -3.498 \| 4.694E-04 \| 0.04261 \| \| 209 \| NA\|MSTRG.61212.1\|NA \| -0.255 \| 0.064 \| -3.999 \| 6.350E-05 \| 0.01748 \| \| 210 \| NA\|MSTRG.89182.1\|NA \| -0.254 \| 0.063 \| -4.034 \| 5.480E-05 \| 0.01683 \| \| 211 \| wdr92\|rna-XM_021564643.2\|NA \| -0.253 \| 0.059 \| -4.322 \| 1.550E-05 \| 0.00914 \| \| 212 \| LOC110527711\|rna-XR_005052577.1\|NA \| -0.252 \| 0.059 \| -4.255 \| 2.090E-05 \| 0.01055 \| \| 213 \| NA\|MSTRG.61010.1\|NA \| -0.252 \| 0.073 \| -3.449 \| 5.622E-04 \| 0.04497 \| \| 214 \| six1a\|rna-XM_021584956.2\|NA \| -0.251 \| 0.065 \| -3.842 \| 1.219E-04 \| 0.02357 \| \| 215 \| NA\|MSTRG.43042.1\|NA \| -0.251 \| 0.070 \| -3.603 \| 3.150E-04 \| 0.03618 \| \| 216 \| NA\|MSTRG.61136.1\|NA \| -0.248 \| 0.073 \| -3.411 \| 6.470E-04 \| 0.04911 \| \| 217 \| LOC100136600\|rna-XM_021608668.2\|NA \| -0.248 \| 0.058 \| -4.240 \| 2.230E-05 \| 0.01069 \| \| 218 \| crls1\|rna-XM_021574179.2\|NA \| -0.247 \| 0.070 \| -3.519 \| 4.330E-04 \| 0.04021 \| \| 219 \| NA\|MSTRG.27943.1\|NA \| -0.246 \| 0.070 \| -3.533 \| 4.108E-04 \| 0.03954 \| \| 220 \| NA\|MSTRG.9041.1\|NA \| -0.246 \| 0.049 \| -4.986 \| 6.150E-07 \| 0.00174 \| \| 221 \| LOC110524251\|rna-XM_021603727.2\|NA \| -0.246 \| 0.065 \| -3.784 \| 1.545E-04 \| 0.02597 \| \| 222 \| LOC110493916\|rna-XM_036949907.1\|NA \| -0.245 \| 0.071 \| -3.463 \| 5.343E-04 \| 0.04413 \| \| 223 \| NA\|MSTRG.37805.1\|NA \| -0.245 \| 0.062 \| -3.940 \| 8.150E-05 \| 0.01984 \| \| 224 \| NA\|MSTRG.41241.1\|NA \| -0.244 \| 0.070 \| -3.468 \| 5.246E-04 \| 0.04413 \| \| 225 \| NA\|MSTRG.9978.1\|NA \| -0.243 \| 0.069 \| -3.525 \| 4.236E-04 \| 0.04021 \| \| 226 \| LOC110522226\|rna-XM_021600440.2\|NA \| -0.243 \| 0.069 \| -3.523 \| 4.267E-04 \| 0.04021 \| \| 227 \| LOC110537712\|rna-XM_036937680.1\|NA \| -0.243 \| 0.070 \| -3.490 \| 4.836E-04 \| 0.04325 \| \| 228 \| LOC110507447\|rna-XM_021587447.2\|NA \| -0.242 \| 0.071 \| -3.414 \| 6.412E-04 \| 0.04892 \| \| 229 \| NA\|MSTRG.16444.1\|NA \| -0.241 \| 0.070 \| -3.436 \| 5.904E-04 \| 0.04604 \| \| 230 \| LOC110538370\|rna-XM_021625145.2\|NA \| -0.241 \| 0.064 \| -3.760 \| 1.698E-04 \| 0.02756 \| \| 231 \| NA\|MSTRG.17018.1\|NA \| -0.240 \| 0.070 \| -3.432 \| 5.986E-04 \| 0.04656 \| \| 232 \| NA\|MSTRG.81331.1\|NA \| -0.240 \| 0.054 \| -4.407 \| 1.050E-05 \| 0.00898 \| \| 233 \| NA\|MSTRG.13680.1\|NA \| -0.239 \| 0.058 \| -4.123 \| 3.730E-05 \| 0.01388 \| \| 234 \| dnm1l\|rna-XM_036959424.1\|NA \| -0.238 \| 0.067 \| -3.532 \| 4.117E-04 \| 0.03954 \| \| 235 \| NA\|MSTRG.79386.1\|NA \| -0.237 \| 0.051 \| -4.664 \| 3.110E-06 \| 0.00470 \| \| 236 \| LOC110537324\|rna-XM_036937308.1\|NA \| -0.236 \| 0.063 \| -3.723 \| 1.972E-04 \| 0.02962 \| \| 237 \| spg11\|rna-XM_021565607.2\|NA \| -0.235 \| 0.064 \| -3.670 \| 2.426E-04 \| 0.03236 \| \| 238 \| NA\|MSTRG.31609.1\|NA \| -0.234 \| 0.056 \| -4.155 \| 3.260E-05 \| 0.01334 \| \| 239 \| NA\|MSTRG.43967.1\|NA \| -0.234 \| 0.064 \| -3.640 \| 2.722E-04 \| 0.03370 \| \| 240 \| LOC110528302\|rna-XM_021610256.2\|NA \| -0.233 \| 0.067 \| -3.471 \| 5.186E-04 \| 0.04413 \| \| 241 \| NA\|MSTRG.35202.1\|NA \| -0.232 \| 0.067 \| -3.479 \| 5.030E-04 \| 0.04364 \| \| 242 \| NA\|MSTRG.30826.1\|NA \| -0.232 \| 0.067 \| -3.475 \| 5.113E-04 \| 0.04385 \| \| 243 \| NA\|MSTRG.6333.1\|NA \| -0.232 \| 0.045 \| -5.187 \| 2.140E-07 \| 0.00121 \| \| 244 \| NA\|MSTRG.84017.1\|NA \| -0.231 \| 0.060 \| -3.833 \| 1.266E-04 \| 0.02359 \| \| 245 \| LOC110526035\|rna-XM_036980225.1\|NA \| -0.230 \| 0.052 \| -4.444 \| 8.830E-06 \| 0.00845 \| \| 246 \| LOC110492109\|rna-XM_021566132.2\|NA \| -0.229 \| 0.063 \| -3.662 \| 2.505E-04 \| 0.03236 \| \| 247 \| LOC110500075\|rna-XM_021577224.2\|NA \| -0.226 \| 0.065 \| -3.510 \| 4.477E-04 \| 0.04103 \| \| 248 \| NA\|MSTRG.72801.1\|NA \| -0.225 \| 0.066 \| -3.429 \| 6.055E-04 \| 0.04677 \| \| 249 \| LOC110499211\|rna-XM_021576199.2\|NA \| -0.225 \| 0.057 \| -3.915 \| 9.050E-05 \| 0.02024 \| \| 250 \| NA\|MSTRG.40202.1\|NA \| -0.223 \| 0.062 \| -3.580 \| 3.439E-04 \| 0.03746 \| \| 251 \| NA\|MSTRG.31189.1\|NA \| -0.223 \| 0.058 \| -3.864 \| 1.115E-04 \| 0.02233 \| \| 252 \| stau2\|rna-XM_036934670.1\|NA \| -0.221 \| 0.062 \| -3.554 \| 3.793E-04 \| 0.03825 \| \| 253 \| tecpr2\|rna-XM_021574003.2\|NA \| -0.221 \| 0.061 \| -3.652 \| 2.604E-04 \| 0.03296 \| \| 254 \| LOC110505429\|rna-XM_021584633.2\|NA \| -0.220 \| 0.057 \| -3.894 \| 9.860E-05 \| 0.02115 \| \| 255 \| LOC110486126\|rna-XM_021557496.2\|NA \| -0.219 \| 0.062 \| -3.519 \| 4.330E-04 \| 0.04021 \| \| 256 \| LOC110492373\|rna-XM_036947351.1\|NA \| -0.216 \| 0.045 \| -4.835 \| 1.330E-06 \| 0.00289 \| \| 257 \| NA\|MSTRG.23611.1\|NA \| -0.215 \| 0.063 \| -3.440 \| 5.821E-04 \| 0.04574 \| \| 258 \| NA\|MSTRG.66064.1\|NA \| -0.214 \| 0.060 \| -3.575 \| 3.504E-04 \| 0.03746 \| \| 259 \| NA\|MSTRG.43512.1\|NA \| -0.213 \| 0.050 \| -4.257 \| 2.070E-05 \| 0.01055 \| \| 260 \| LOC110521263\|rna-XM_021598719.2\|NA \| -0.210 \| 0.055 \| -3.794 \| 1.480E-04 \| 0.02548 \| \| 261 \| ddhd1b\|rna-XM_021612316.2\|NA \| -0.210 \| 0.060 \| -3.466 \| 5.291E-04 \| 0.04413 \| \| 262 \| NA\|MSTRG.56511.1\|NA \| -0.206 \| 0.059 \| -3.515 \| 4.390E-04 \| 0.04063 \| \| 263 \| NA\|MSTRG.6535.1\|NA \| -0.205 \| 0.055 \| -3.756 \| 1.727E-04 \| 0.02771 \| \| 264 \| LOC110491308\|rna-XM_021564664.2\|NA \| -0.201 \| 0.055 \| -3.632 \| 2.817E-04 \| 0.03428 \| \| 265 \| NA\|MSTRG.88725.1\|NA \| -0.197 \| 0.051 \| -3.831 \| 1.278E-04 \| 0.02359 \| \| 266 \| washc4\|rna-XM_036968633.1\|NA \| -0.195 \| 0.049 \| -4.007 \| 6.160E-05 \| 0.01748 \| \| 267 \| LOC110507499\|rna-XM_036965703.1\|NA \| -0.194 \| 0.056 \| -3.491 \| 4.812E-04 \| 0.04325 \| \| 268 \| NA\|MSTRG.43542.1\|NA \| -0.192 \| 0.057 \| -3.402 \| 6.701E-04 \| 0.04958 \| \| 269 \| NA\|MSTRG.56297.1\|NA \| -0.191 \| 0.047 \| -4.061 \| 4.890E-05 \| 0.01551 \| \| 270 \| LOC110526374\|rna-XM_021607292.2\|NA \| -0.190 \| 0.053 \| -3.583 \| 3.401E-04 \| 0.03736 \| \| 271 \| LOC110499341\|rna-XM_036956203.1\|NA \| -0.189 \| 0.051 \| -3.709 \| 2.079E-04 \| 0.03057 \| \| 272 \| arhgef25a\|rna-XM_036946877.1\|NA \| -0.189 \| 0.048 \| -3.933 \| 8.390E-05 \| 0.02009 \| \| 273 \| LOC110494274\|rna-XM_021569217.2\|NA \| -0.189 \| 0.052 \| -3.613 \| 3.024E-04 \| 0.03572 \| \| 274 \| LOC110537452\|rna-XM_021623510.2\|NA \| -0.187 \| 0.054 \| -3.474 \| 5.126E-04 \| 0.04385 \| \| 275 \| wu:fb55g09\|rna-XM_021610803.2\|NA \| -0.187 \| 0.051 \| -3.664 \| 2.482E-04 \| 0.03236 \| \| 276 \| NA\|MSTRG.25790.1\|NA \| -0.186 \| 0.052 \| -3.565 \| 3.645E-04 \| 0.03769 \| \| 277 \| LOC110528493\|rna-XM_036984071.1\|NA \| -0.184 \| 0.048 \| -3.795 \| 1.475E-04 \| 0.02548 \| \| 278 \| NA\|MSTRG.10221.1\|NA \| -0.183 \| 0.049 \| -3.728 \| 1.927E-04 \| 0.02957 \| \| 279 \| NA\|MSTRG.21076.1\|NA \| -0.182 \| 0.046 \| -3.995 \| 6.480E-05 \| 0.01748 \| \| 280 \| heatr3\|rna-XM_021607688.2\|NA \| -0.181 \| 0.053 \| -3.399 \| 6.755E-04 \| 0.04958 \| \| 281 \| LOC110528073\|rna-XM_021609869.2\|NA \| -0.175 \| 0.038 \| -4.589 \| 4.450E-06 \| 0.00523 \| \| 282 \| LOC110530683\|rna-XM_036986341.1\|NA \| -0.170 \| 0.042 \| -4.075 \| 4.600E-05 \| 0.01527 \| \| 283 \| NA\|MSTRG.17107.1\|NA \| -0.166 \| 0.040 \| -4.164 \| 3.130E-05 \| 0.01330 \| \| 284 \| NA\|MSTRG.32153.1\|NA \| -0.164 \| 0.043 \| -3.801 \| 1.442E-04 \| 0.02514 \| \| 285 \| LOC110524847\|rna-XM_021604811.2\|NA \| -0.163 \| 0.044 \| -3.719 \| 2.002E-04 \| 0.02975 \| \| 286 \| NA\|MSTRG.73598.1\|NA \| -0.162 \| 0.040 \| -4.055 \| 5.020E-05 \| 0.01567 \| \| 287 \| NA\|MSTRG.88177.1\|NA \| -0.151 \| 0.042 \| -3.563 \| 3.671E-04 \| 0.03769 \| \| 288 \| NA\|MSTRG.52384.1\|NA \| -0.149 \| 0.043 \| -3.468 \| 5.249E-04 \| 0.04413 \| \| 289 \| LOC110522333\|rna-XM_021600657.2\|NA \| -0.133 \| 0.036 \| -3.729 \| 1.922E-04 \| 0.02957 \| \| 290 \| LOC110538221\|rna-XM_021624891.2\|NA \| -0.103 \| 0.028 \| -3.693 \| 2.216E-04 \| 0.03109 \| \| 291 \| LOC110528591\|rna-XM_036984208.1\|NA \| 0.127 \| 0.037 \| 3.466 \| 5.286E-04 \| 0.04413 \| \| 292 \| NA\|MSTRG.80989.1\|NA \| 0.155 \| 0.043 \| 3.596 \| 3.228E-04 \| 0.03675 \| \| 293 \| LOC110498114\|rna-XM_021574706.2\|NA \| 0.165 \| 0.046 \| 3.599 \| 3.195E-04 \| 0.03652 \| \| 294 \| LOC110536259\|rna-XM_021621942.2\|NA \| 0.165 \| 0.048 \| 3.444 \| 5.739E-04 \| 0.04564 \| \| 295 \| NA\|MSTRG.54321.1\|NA \| 0.170 \| 0.044 \| 3.872 \| 1.079E-04 \| 0.02233 \| \| 296 \| specc1la\|rna-XM_021621264.2\|NA \| 0.174 \| 0.048 \| 3.638 \| 2.750E-04 \| 0.03376 \| \| 297 \| LOC110492121\|rna-XM_021566155.2\|NA \| 0.178 \| 0.050 \| 3.536 \| 4.059E-04 \| 0.03938 \| \| 298 \| NA\|MSTRG.55348.1\|NA \| 0.189 \| 0.055 \| 3.459 \| 5.419E-04 \| 0.04434 \| \| 299 \| ttyh2\|rna-XM_021576244.2\|NA \| 0.195 \| 0.051 \| 3.814 \| 1.368E-04 \| 0.02430 \| \| 300 \| NA\|MSTRG.86690.1\|NA \| 0.196 \| 0.056 \| 3.522 \| 4.281E-04 \| 0.04021 \| \| 301 \| NA\|MSTRG.92561.1\|NA \| 0.198 \| 0.055 \| 3.590 \| 3.305E-04 \| 0.03688 \| \| 302 \| NA\|MSTRG.33787.1\|NA \| 0.199 \| 0.056 \| 3.540 \| 4.000E-04 \| 0.03894 \| \| 303 \| mgat1a\|rna-XM_021594697.2\|NA \| 0.206 \| 0.055 \| 3.772 \| 1.621E-04 \| 0.02691 \| \| 304 \| NA\|MSTRG.27332.1\|NA \| 0.208 \| 0.055 \| 3.792 \| 1.493E-04 \| 0.02555 \| \| 305 \| uggt2\|rna-XM_036974551.1\|NA \| 0.210 \| 0.049 \| 4.334 \| 1.470E-05 \| 0.00914 \| \| 306 \| epb41l3b\|rna-XM_036966494.1\|NA \| 0.220 \| 0.062 \| 3.519 \| 4.326E-04 \| 0.04021 \| \| 307 \| NA\|MSTRG.50669.1\|NA \| 0.229 \| 0.067 \| 3.404 \| 6.641E-04 \| 0.04958 \| \| 308 \| NA\|MSTRG.5850.1\|NA \| 0.232 \| 0.068 \| 3.406 \| 6.581E-04 \| 0.04958 \| \| 309 \| mpped2\|rna-XM_021575859.2\|NA \| 0.238 \| 0.060 \| 3.992 \| 6.540E-05 \| 0.01748 \| \| 310 \| NA\|MSTRG.27931.1\|NA \| 0.239 \| 0.068 \| 3.512 \| 4.450E-04 \| 0.04103 \| \| 311 \| NA\|MSTRG.37499.1\|NA \| 0.241 \| 0.070 \| 3.429 \| 6.057E-04 \| 0.04677 \| \| 312 \| NA\|MSTRG.11072.1\|NA \| 0.243 \| 0.057 \| 4.279 \| 1.880E-05 \| 0.01038 \| \| 313 \| LOC110494367\|rna-XM_036950426.1\|NA \| 0.244 \| 0.060 \| 4.062 \| 4.870E-05 \| 0.01551 \| \| 314 \| bgnb\|rna-XM_036947570.1\|NA \| 0.250 \| 0.070 \| 3.557 \| 3.757E-04 \| 0.03825 \| \| 315 \| cspg4\|rna-XM_021564725.2\|NA \| 0.253 \| 0.061 \| 4.123 \| 3.740E-05 \| 0.01388 \| \| 316 \| LOC110502849\|rna-XM_021581183.2\|NA \| 0.255 \| 0.057 \| 4.440 \| 8.980E-06 \| 0.00845 \| \| 317 \| rpn2\|rna-XM_021609415.2\|NA \| 0.260 \| 0.075 \| 3.470 \| 5.205E-04 \| 0.04413 \| \| 318 \| NA\|MSTRG.18794.1\|NA \| 0.267 \| 0.078 \| 3.441 \| 5.796E-04 \| 0.04574 \| \| 319 \| ttc33\|rna-XM_021590585.2\|NA \| 0.269 \| 0.079 \| 3.400 \| 6.746E-04 \| 0.04958 \| \| 320 \| LOC110531865\|rna-XM_021615335.2\|NA \| 0.275 \| 0.072 \| 3.835 \| 1.255E-04 \| 0.02359 \| \| 321 \| hyou1\|rna-XM_036989906.1\|NA \| 0.278 \| 0.076 \| 3.643 \| 2.694E-04 \| 0.03366 \| \| 322 \| tjp2a\|rna-XM_036967845.1\|NA \| 0.280 \| 0.069 \| 4.053 \| 5.050E-05 \| 0.01567 \| \| 323 \| si:ch211-113g11.6\|rna-XM_021559185.2\|NA \| 0.283 \| 0.083 \| 3.403 \| 6.664E-04 \| 0.04958 \| \| 324 \| NA\|MSTRG.46082.1\|NA \| 0.290 \| 0.076 \| 3.804 \| 1.424E-04 \| 0.02497 \| \| 325 \| LOC110497901\|rna-XM_021574355.2\|NA \| 0.291 \| 0.079 \| 3.695 \| 2.195E-04 \| 0.03109 \| \| 326 \| NA\|MSTRG.87482.1\|NA \| 0.299 \| 0.081 \| 3.692 \| 2.223E-04 \| 0.03109 \| \| 327 \| LOC110502707\|rna-XM_021580955.2\|NA \| 0.301 \| 0.084 \| 3.572 \| 3.536E-04 \| 0.03746 \| \| 328 \| LOC110508914\|rna-XM_021589823.2\|NA \| 0.302 \| 0.063 \| 4.773 \| 1.820E-06 \| 0.00320 \| \| 329 \| bgna\|rna-XM_021566437.2\|NA \| 0.316 \| 0.090 \| 3.504 \| 4.578E-04 \| 0.04169 \| \| 330 \| LOC110485921\|rna-XM_021557154.2\|NA \| 0.319 \| 0.092 \| 3.475 \| 5.101E-04 \| 0.04385 \| \| 331 \| LOC110500180\|rna-XM_021577383.2\|NA \| 0.320 \| 0.087 \| 3.663 \| 2.496E-04 \| 0.03236 \| \| 332 \| NA\|MSTRG.17706.1\|NA \| 0.328 \| 0.084 \| 3.913 \| 9.130E-05 \| 0.02024 \| \| 333 \| LOC110508975\|rna-XM_021589911.2\|NA \| 0.335 \| 0.095 \| 3.522 \| 4.291E-04 \| 0.04021 \| \| 334 \| NA\|MSTRG.9752.1\|NA \| 0.335 \| 0.095 \| 3.548 \| 3.876E-04 \| 0.03840 \| \| 335 \| nomo\|rna-XM_036949408.1\|NA \| 0.337 \| 0.078 \| 4.326 \| 1.520E-05 \| 0.00914 \| \| 336 \| LOC110507602\|rna-XM_021587735.2\|NA \| 0.338 \| 0.082 \| 4.118 \| 3.820E-05 \| 0.01394 \| \| 337 \| nfatc1\|rna-XM_021571742.2\|NA \| 0.340 \| 0.079 \| 4.310 \| 1.630E-05 \| 0.00937 \| \| 338 \| vill\|rna-XM_036945629.1\|NA \| 0.341 \| 0.091 \| 3.744 \| 1.808E-04 \| 0.02852 \| \| 339 \| NA\|MSTRG.40307.1\|NA \| 0.344 \| 0.079 \| 4.336 \| 1.450E-05 \| 0.00914 \| \| 340 \| NA\|MSTRG.49521.1\|NA \| 0.347 \| 0.083 \| 4.160 \| 3.190E-05 \| 0.01330 \| \| 341 \| NA\|MSTRG.52969.1\|NA \| 0.352 \| 0.099 \| 3.564 \| 3.652E-04 \| 0.03769 \| \| 342 \| ercc6l\|rna-XM_021618270.2\|NA \| 0.356 \| 0.099 \| 3.586 \| 3.360E-04 \| 0.03706 \| \| 343 \| NA\|MSTRG.91619.1\|NA \| 0.371 \| 0.095 \| 3.887 \| 1.017E-04 \| 0.02142 \| \| 344 \| ahsg2\|rna-XM_021589944.2\|NA \| 0.372 \| 0.091 \| 4.083 \| 4.440E-05 \| 0.01511 \| \| 345 \| stc1\|rna-XM_021621470.2\|NA \| 0.376 \| 0.096 \| 3.909 \| 9.290E-05 \| 0.02024 \| \| 346 \| NA\|MSTRG.63700.1\|NA \| 0.380 \| 0.100 \| 3.785 \| 1.535E-04 \| 0.02595 \| \| 347 \| LOC110530835\|rna-XM_036986605.1\|NA \| 0.381 \| 0.093 \| 4.116 \| 3.850E-05 \| 0.01394 \| \| 348 \| LOC110531808\|rna-XM_021615238.2\|NA \| 0.383 \| 0.112 \| 3.409 \| 6.510E-04 \| 0.04928 \| \| 349 \| timm21\|rna-XM_021620460.2\|NA \| 0.389 \| 0.114 \| 3.412 \| 6.442E-04 \| 0.04902 \| \| 350 \| racgap1\|rna-XM_021566127.2\|NA \| 0.392 \| 0.111 \| 3.524 \| 4.252E-04 \| 0.04021 \| \| 351 \| NA\|MSTRG.21638.1\|NA \| 0.392 \| 0.110 \| 3.564 \| 3.658E-04 \| 0.03769 \| \| 352 \| NA\|MSTRG.11592.1\|NA \| 0.393 \| 0.102 \| 3.865 \| 1.113E-04 \| 0.02233 \| \| 353 \| NA\|MSTRG.47880.1\|NA \| 0.397 \| 0.116 \| 3.421 \| 6.250E-04 \| 0.04795 \| \| 354 \| NA\|MSTRG.29009.1\|NA \| 0.403 \| 0.117 \| 3.454 \| 5.520E-04 \| 0.04463 \| \| 355 \| NA\|MSTRG.54657.1\|NA \| 0.405 \| 0.111 \| 3.667 \| 2.454E-04 \| 0.03236 \| \| 356 \| NA\|MSTRG.86790.1\|NA \| 0.408 \| 0.108 \| 3.785 \| 1.534E-04 \| 0.02595 \| \| 357 \| LOC110492346\|rna-XM_021566584.2\|NA \| 0.412 \| 0.094 \| 4.393 \| 1.120E-05 \| 0.00901 \| \| 358 \| LOC110498032\|rna-XM_036954954.1\|NA \| 0.414 \| 0.113 \| 3.669 \| 2.433E-04 \| 0.03236 \| \| 359 \| NA\|MSTRG.20943.1\|NA \| 0.443 \| 0.108 \| 4.103 \| 4.080E-05 \| 0.01459 \| \| 360 \| LOC110490880\|rna-XM_036945666.1\|NA \| 0.447 \| 0.124 \| 3.608 \| 3.091E-04 \| 0.03608 \| \| 361 \| NA\|MSTRG.27958.1\|NA \| 0.450 \| 0.106 \| 4.266 \| 1.990E-05 \| 0.01055 \| \| 362 \| LOC110511819\|rna-XM_021592563.2\|NA \| 0.460 \| 0.125 \| 3.663 \| 2.497E-04 \| 0.03236 \| \| 363 \| NA\|MSTRG.85398.1\|NA \| 0.486 \| 0.136 \| 3.569 \| 3.584E-04 \| 0.03769 \| \| 364 \| NA\|MSTRG.87442.1\|NA \| 0.508 \| 0.142 \| 3.593 \| 3.275E-04 \| 0.03684 \| \| 365 \| knstrn\|rna-XM_021600599.2\|NA \| 0.513 \| 0.138 \| 3.726 \| 1.946E-04 \| 0.02962 \| \| 366 \| LOC110532114\|rna-XR_005053249.1\|NA \| 0.519 \| 0.130 \| 3.996 \| 6.430E-05 \| 0.01748 \| \| 367 \| LOC110529064\|rna-XM_036984353.1\|NA \| 0.521 \| 0.151 \| 3.454 \| 5.528E-04 \| 0.04463 \| \| 368 \| LOC110521605\|gene-LOC110521605\|NA \| 0.523 \| 0.146 \| 3.589 \| 3.325E-04 \| 0.03696 \| \| 369 \| NA\|MSTRG.70354.1\|NA \| 0.553 \| 0.153 \| 3.606 \| 3.106E-04 \| 0.03608 \| \| 370 \| NA\|MSTRG.80186.1\|NA \| 0.558 \| 0.132 \| 4.227 \| 2.370E-05 \| 0.01115 \| \| 371 \| NA\|MSTRG.8372.1\|NA \| 0.571 \| 0.161 \| 3.543 \| 3.957E-04 \| 0.03879 \| \| 372 \| zgc:110540\|rna-XM_021617560.2\|NA \| 0.578 \| 0.133 \| 4.343 \| 1.400E-05 \| 0.00914 \| \| 373 \| LOC110486057\|rna-XM_036947133.1\|NA \| 0.616 \| 0.160 \| 3.847 \| 1.194E-04 \| 0.02341 \| \| 374 \| NA\|MSTRG.70383.1\|NA \| 0.618 \| 0.177 \| 3.496 \| 4.721E-04 \| 0.04272 \| \| 375 \| LOC118940171\|rna-XM_036948453.1\|NA \| 0.627 \| 0.180 \| 3.475 \| 5.112E-04 \| 0.04385 \| \| 376 \| LOC110493778\|rna-XM_036949768.1\|NA \| 0.663 \| 0.152 \| 4.355 \| 1.330E-05 \| 0.00914 \| \| 377 \| NA\|MSTRG.33509.1\|NA \| 0.693 \| 0.165 \| 4.203 \| 2.640E-05 \| 0.01202 \| \| 378 \| LOC110493648\|rna-XM_021568038.2\|NA \| 0.695 \| 0.171 \| 4.061 \| 4.880E-05 \| 0.01551 \| \| 379 \| mettl21e\|rna-XM_036972377.1\|NA \| 0.716 \| 0.182 \| 3.942 \| 8.080E-05 \| 0.01984 \| \| 380 \| NA\|MSTRG.63640.1\|NA \| 0.850 \| 0.208 \| 4.097 \| 4.190E-05 \| 0.01479 \| \| 381 \| LOC110491669\|rna-XM_021565291.2\|NA \| 0.912 \| 0.265 \| 3.442 \| 5.778E-04 \| 0.04574 \| \| 382 \| LOC110505631\|rna-XM_021584972.2\|NA \| 1.324 \| 0.367 \| 3.603 \| 3.141E-04 \| 0.03618 \| \| 383 \| LOC110498431\|rna-XM_036933693.1\|NA \| 1.351 \| 0.363 \| 3.719 \| 1.997E-04 \| 0.02975 \| \| 384 \| LOC110528486\|rna-XM_021610589.2\|NA \| 1.778 \| 0.320 \| 5.554 \| 2.790E-08 \| 0.00039 \| \| 385 \| NA\|MSTRG.63348.1\|NA \| 1.804 \| 0.442 \| 4.080 \| 4.500E-05 \| 0.01511 \| |  |  |  |  |  |
| --- | --- | --- | --- | --- | --- | --- | --- | --- | --- | --- | --- | --- | --- | --- | --- | --- | --- | --- | --- | --- | --- | --- | --- | --- | --- | --- | --- | --- | --- | --- | --- | --- | --- | --- | --- | --- | --- | --- | --- | --- | --- | --- | --- | --- | --- | --- | --- | --- | --- | --- | --- | --- | --- | --- | --- | --- | --- | --- | --- | --- | --- | --- | --- | --- | --- | --- | --- | --- | --- | --- | --- | --- | --- | --- | --- | --- | --- | --- | --- | --- | --- | --- | --- | --- | --- | --- | --- | --- | --- | --- | --- | --- | --- | --- | --- | --- | --- | --- | --- | --- | --- | --- | --- | --- | --- | --- | --- | --- | --- | --- | --- | --- | --- | --- | --- | --- | --- | --- | --- | --- | --- | --- | --- | --- | --- | --- | --- | --- | --- | --- | --- | --- | --- | --- | --- | --- | --- | --- | --- | --- | --- | --- | --- | --- | --- | --- | --- | --- | --- | --- | --- | --- | --- | --- | --- | --- | --- | --- | --- | --- | --- | --- | --- | --- | --- | --- | --- | --- | --- | --- | --- | --- | --- | --- | --- | --- | --- | --- | --- | --- | --- | --- | --- | --- | --- | --- | --- | --- | --- | --- | --- | --- | --- | --- | --- | --- | --- | --- | --- | --- | --- | --- | --- | --- | --- | --- | --- | --- | --- | --- | --- | --- | --- | --- | --- | --- | --- | --- | --- | --- | --- | --- | --- | --- | --- | --- | --- | --- | --- | --- | --- | --- | --- | --- | --- | --- | --- | --- | --- | --- | --- | --- | --- | --- | --- | --- | --- | --- | --- | --- | --- | --- | --- | --- | --- | --- | --- | --- | --- | --- | --- | --- | --- | --- | --- | --- | --- | --- | --- | --- | --- | --- | --- | --- | --- | --- | --- | --- | --- | --- | --- | --- | --- | --- | --- | --- | --- | --- | --- | --- | --- | --- | --- | --- | --- | --- | --- | --- | --- | --- | --- | --- | --- | --- | --- | --- | --- | --- | --- | --- | --- | --- | --- | --- | --- | --- | --- | --- | --- | --- | --- | --- | --- | --- | --- | --- | --- | --- | --- | --- | --- | --- | --- | --- | --- | --- | --- | --- | --- | --- | --- | --- | --- | --- | --- | --- | --- | --- | --- | --- | --- | --- | --- | --- | --- | --- | --- | --- | --- | --- | --- | --- | --- | --- | --- | --- | --- | --- | --- | --- | --- | --- | --- | --- | --- | --- | --- | --- | --- | --- | --- | --- | --- | --- | --- | --- | --- | --- | --- | --- | --- | --- | --- | --- | --- | --- | --- | --- | --- | --- | --- | --- | --- | --- | --- | --- | --- | --- | --- | --- | --- | --- | --- | --- | --- | --- | --- | --- | --- | --- | --- | --- | --- | --- | --- | --- | --- | --- | --- | --- | --- | --- | --- | --- | --- | --- | --- | --- | --- | --- | --- | --- | --- | --- | --- | --- | --- | --- | --- | --- | --- | --- | --- | --- | --- | --- | --- | --- | --- | --- | --- | --- | --- | --- | --- | --- | --- | --- | --- | --- | --- | --- | --- | --- | --- | --- | --- | --- | --- | --- | --- | --- | --- | --- | --- | --- | --- | --- | --- | --- | --- | --- | --- | --- | --- | --- | --- | --- | --- | --- | --- | --- | --- | --- | --- | --- | --- | --- | --- | --- | --- | --- | --- | --- | --- | --- | --- | --- | --- | --- | --- | --- | --- | --- | --- | --- | --- | --- | --- | --- | --- | --- | --- | --- | --- | --- | --- | --- | --- | --- | --- | --- | --- | --- | --- | --- | --- | --- | --- | --- | --- | --- | --- | --- | --- | --- | --- | --- | --- | --- | --- | --- | --- | --- | --- | --- | --- | --- | --- | --- | --- | --- | --- | --- | --- | --- | --- | --- | --- | --- | --- | --- | --- | --- | --- | --- | --- | --- | --- | --- | --- | --- | --- | --- | --- | --- | --- | --- | --- | --- | --- | --- | --- | --- | --- | --- | --- | --- | --- | --- | --- | --- | --- | --- | --- | --- | --- | --- | --- | --- | --- | --- | --- | --- | --- | --- | --- | --- | --- | --- | --- | --- | --- | --- | --- | --- | --- | --- | --- | --- | --- | --- | --- | --- | --- | --- | --- | --- | --- | --- | --- | --- | --- | --- | --- | --- | --- | --- | --- | --- | --- | --- | --- | --- | --- | --- | --- | --- | --- | --- | --- | --- | --- | --- | --- | --- | --- | --- | --- | --- | --- | --- | --- | --- | --- | --- | --- | --- | --- | --- | --- | --- | --- | --- | --- | --- | --- | --- | --- | --- | --- | --- | --- | --- | --- | --- | --- | --- | --- | --- | --- | --- | --- | --- | --- | --- | --- | --- | --- | --- | --- | --- | --- | --- | --- | --- | --- | --- | --- | --- | --- | --- | --- | --- | --- | --- | --- | --- | --- | --- | --- | --- | --- | --- | --- | --- | --- | --- | --- | --- | --- | --- | --- | --- | --- | --- | --- | --- | --- | --- | --- | --- | --- | --- | --- | --- | --- | --- | --- | --- | --- | --- | --- | --- | --- | --- | --- | --- | --- | --- | --- | --- | --- | --- | --- | --- | --- | --- | --- | --- | --- | --- | --- | --- | --- | --- | --- | --- | --- | --- | --- | --- | --- | --- | --- | --- | --- | --- | --- | --- | --- | --- | --- | --- | --- | --- | --- | --- | --- | --- | --- | --- | --- | --- | --- | --- | --- | --- | --- | --- | --- | --- | --- | --- | --- | --- | --- | --- | --- | --- | --- | --- | --- | --- | --- | --- | --- | --- | --- | --- | --- | --- | --- | --- | --- | --- | --- | --- | --- | --- | --- | --- | --- | --- | --- | --- | --- | --- | --- | --- | --- | --- | --- | --- | --- | --- | --- | --- | --- | --- | --- | --- | --- | --- | --- | --- | --- | --- | --- | --- | --- | --- | --- | --- | --- | --- | --- | --- | --- | --- | --- | --- | --- | --- | --- | --- | --- | --- | --- | --- | --- | --- | --- | --- | --- | --- | --- | --- | --- | --- | --- | --- | --- | --- | --- | --- | --- | --- | --- | --- | --- | --- | --- | --- | --- | --- | --- | --- | --- | --- | --- | --- | --- | --- | --- | --- | --- | --- | --- | --- | --- | --- | --- | --- | --- | --- | --- | --- | --- | --- | --- | --- | --- | --- | --- | --- | --- | --- | --- | --- | --- | --- | --- | --- | --- | --- | --- | --- | --- | --- | --- | --- | --- | --- | --- | --- | --- | --- | --- | --- | --- | --- | --- | --- | --- | --- | --- | --- | --- | --- | --- | --- | --- | --- | --- | --- | --- | --- | --- | --- | --- | --- | --- | --- | --- | --- | --- | --- | --- | --- | --- | --- | --- | --- | --- | --- | --- | --- | --- | --- | --- | --- | --- | --- | --- | --- | --- | --- | --- | --- | --- | --- | --- | --- | --- | --- | --- | --- | --- | --- | --- | --- | --- | --- | --- | --- | --- | --- | --- | --- | --- | --- | --- | --- | --- | --- | --- | --- | --- | --- | --- | --- | --- | --- | --- | --- | --- | --- | --- | --- | --- | --- | --- | --- | --- | --- | --- | --- | --- | --- | --- | --- | --- | --- | --- | --- | --- | --- | --- | --- | --- | --- | --- | --- | --- | --- | --- | --- | --- | --- | --- | --- | --- | --- | --- | --- | --- | --- | --- | --- | --- | --- | --- | --- | --- | --- | --- | --- | --- | --- | --- | --- | --- | --- | --- | --- | --- | --- | --- | --- | --- | --- | --- | --- | --- | --- | --- | --- | --- | --- | --- | --- | --- | --- | --- | --- | --- | --- | --- | --- | --- | --- | --- | --- | --- | --- | --- | --- | --- | --- | --- | --- | --- | --- | --- | --- | --- | --- | --- | --- | --- | --- | --- | --- | --- | --- | --- | --- | --- | --- | --- | --- | --- | --- | --- | --- | --- | --- | --- | --- | --- | --- | --- | --- | --- | --- | --- | --- | --- | --- | --- | --- | --- | --- | --- | --- | --- | --- | --- | --- | --- | --- | --- | --- | --- | --- | --- | --- | --- | --- | --- | --- | --- | --- | --- | --- | --- | --- | --- | --- | --- | --- | --- | --- | --- | --- | --- | --- | --- | --- | --- | --- | --- | --- | --- | --- | --- | --- | --- | --- | --- | --- | --- | --- | --- | --- | --- | --- | --- | --- | --- | --- | --- | --- | --- | --- | --- | --- | --- | --- | --- | --- | --- | --- | --- | --- | --- | --- | --- | --- | --- | --- | --- | --- | --- | --- | --- | --- | --- | --- | --- | --- | --- | --- | --- | --- | --- | --- | --- | --- | --- | --- | --- | --- | --- | --- | --- | --- | --- | --- | --- | --- | --- | --- | --- | --- | --- | --- | --- | --- | --- | --- | --- | --- | --- | --- | --- | --- | --- | --- | --- | --- | --- | --- | --- | --- | --- | --- | --- | --- | --- | --- | --- | --- | --- | --- | --- | --- | --- | --- | --- | --- | --- | --- | --- | --- | --- | --- | --- | --- | --- | --- | --- | --- | --- | --- | --- | --- | --- | --- | --- | --- | --- | --- | --- | --- | --- | --- | --- | --- | --- | --- | --- | --- | --- | --- | --- | --- | --- | --- | --- | --- | --- | --- | --- | --- | --- | --- | --- | --- | --- | --- | --- | --- | --- | --- | --- | --- | --- | --- | --- | --- | --- | --- | --- | --- | --- | --- | --- | --- | --- | --- | --- | --- | --- | --- | --- | --- | --- | --- | --- | --- | --- | --- | --- | --- | --- | --- | --- | --- | --- | --- | --- | --- | --- | --- | --- | --- | --- | --- | --- | --- | --- | --- | --- | --- | --- | --- | --- | --- | --- | --- | --- | --- | --- | --- | --- | --- | --- | --- | --- | --- | --- | --- | --- | --- | --- | --- | --- | --- | --- | --- | --- | --- | --- | --- | --- | --- | --- | --- | --- | --- | --- | --- | --- | --- | --- | --- | --- | --- | --- | --- | --- | --- | --- | --- | --- | --- | --- | --- | --- | --- | --- | --- | --- | --- | --- | --- | --- | --- | --- | --- | --- | --- | --- | --- | --- | --- | --- | --- | --- | --- | --- | --- | --- | --- | --- | --- | --- | --- | --- | --- | --- | --- | --- | --- | --- | --- | --- | --- | --- | --- | --- | --- | --- | --- | --- | --- | --- | --- | --- | --- | --- | --- | --- | --- | --- | --- | --- | --- | --- | --- | --- | --- | --- | --- | --- | --- | --- | --- | --- | --- | --- | --- | --- | --- | --- | --- | --- | --- | --- | --- | --- | --- | --- | --- | --- | --- | --- | --- | --- | --- | --- | --- | --- | --- | --- | --- | --- | --- | --- | --- | --- | --- | --- | --- | --- | --- | --- | --- | --- | --- | --- | --- | --- | --- | --- | --- | --- | --- | --- | --- | --- | --- | --- | --- | --- | --- | --- | --- | --- | --- | --- | --- | --- | --- | --- | --- | --- | --- | --- | --- | --- | --- | --- | --- | --- | --- | --- | --- | --- | --- | --- | --- | --- | --- | --- | --- | --- | --- | --- | --- | --- | --- | --- | --- | --- | --- | --- | --- | --- | --- | --- | --- | --- | --- | --- | --- | --- | --- | --- | --- | --- | --- | --- | --- | --- | --- | --- | --- | --- | --- | --- | --- | --- | --- | --- | --- | --- | --- | --- | --- | --- | --- | --- | --- | --- | --- | --- | --- | --- | --- | --- | --- | --- | --- | --- | --- | --- | --- | --- | --- | --- | --- | --- | --- | --- | --- | --- | --- | --- | --- | --- | --- | --- | --- | --- | --- | --- | --- | --- | --- | --- | --- | --- | --- | --- | --- | --- | --- | --- | --- | --- | --- | --- | --- | --- | --- | --- | --- | --- | --- | --- | --- | --- | --- | --- | --- | --- | --- | --- | --- | --- | --- | --- | --- | --- | --- | --- | --- | --- | --- | --- | --- | --- | --- | --- | --- | --- | --- | --- | --- | --- | --- | --- | --- | --- | --- | --- | --- | --- | --- | --- | --- | --- | --- | --- | --- | --- | --- | --- | --- | --- | --- | --- | --- | --- | --- | --- | --- | --- | --- | --- | --- | --- | --- | --- | --- | --- | --- | --- | --- | --- | --- | --- | --- | --- | --- | --- | --- | --- | --- | --- | --- | --- | --- | --- | --- | --- | --- | --- | --- | --- | --- | --- | --- | --- | --- | --- | --- | --- | --- | --- | --- | --- | --- | --- | --- | --- | --- | --- | --- | --- | --- | --- | --- | --- | --- | --- | --- | --- | --- | --- | --- | --- | --- | --- | --- | --- | --- | --- | --- | --- | --- | --- | --- | --- | --- | --- | --- | --- | --- | --- | --- | --- | --- | --- | --- | --- | --- | --- | --- | --- | --- | --- | --- | --- | --- | --- | --- | --- | --- | --- | --- | --- | --- | --- | --- | --- | --- | --- | --- | --- | --- | --- | --- | --- | --- | --- | --- | --- | --- | --- | --- | --- | --- | --- | --- | --- | --- | --- | --- | --- | --- | --- | --- | --- | --- | --- | --- | --- | --- | --- | --- | --- | --- | --- | --- | --- | --- | --- | --- | --- | --- | --- | --- | --- | --- | --- | --- | --- | --- | --- | --- | --- | --- | --- | --- | --- | --- | --- | --- | --- | --- | --- | --- | --- | --- | --- | --- | --- | --- | --- | --- | --- | --- | --- | --- | --- | --- | --- | --- | --- | --- | --- | --- | --- | --- | --- | --- | --- | --- | --- | --- | --- | --- | --- | --- | --- | --- | --- | --- | --- | --- | --- | --- | --- | --- | --- | --- | --- | --- | --- | --- | --- | --- | --- | --- | --- | --- | --- | --- | --- | --- | --- | --- | --- | --- | --- | --- | --- | --- | --- | --- | --- | --- | --- | --- | --- | --- | --- | --- | --- | --- | --- | --- | --- | --- | --- | --- | --- | --- | --- | --- | --- | --- | --- | --- | --- | --- | --- | --- | --- | --- | --- | --- | --- | --- | --- | --- | --- | --- | --- | --- | --- | --- | --- | --- | --- | --- | --- | --- | --- | --- | --- | --- | --- | --- | --- | --- | --- | --- | --- | --- | --- | --- | --- | --- | --- | --- | --- | --- | --- | --- | --- | --- | --- | --- | --- | --- | --- | --- | --- | --- | --- | --- | --- | --- | --- | --- | --- | --- | --- | --- | --- | --- | --- | --- | --- | --- | --- | --- | --- | --- | --- | --- | --- | --- | --- | --- | --- | --- | --- | --- | --- | --- | --- | --- | --- | --- | --- | --- | --- | --- | --- | --- | --- | --- | --- | --- | --- | --- | --- | --- | --- | --- | --- | --- | --- | --- | --- | --- | --- | --- | --- | --- | --- | --- | --- | --- | --- | --- | --- | --- | --- | --- | --- | --- | --- | --- | --- | --- | --- | --- | --- | --- | --- | --- | --- | --- | --- | --- | --- | --- | --- | --- | --- | --- | --- | --- | --- | --- | --- | --- | --- | --- | --- | --- | --- | --- | --- | --- | --- | --- | --- | --- | --- | --- | --- | --- | --- | --- | --- | --- | --- | --- | --- | --- | --- | --- | --- | --- | --- | --- | --- | --- | --- | --- | --- | --- | --- | --- | --- | --- | --- | --- | --- | --- | --- | --- | --- | --- | --- | --- | --- | --- | --- | --- | --- | --- | --- | --- | --- | --- | --- | --- | --- | --- | --- | --- | --- | --- | --- | --- | --- | --- | --- | --- | --- | --- | --- | --- | --- | --- | --- | --- | --- | --- | --- | --- | --- | --- | --- | --- | --- | --- | --- | --- | --- | --- | --- | --- | --- | --- | --- | --- | --- | --- | --- | --- | --- | --- | --- | --- | --- | --- | --- | --- | --- | --- | --- | --- | --- | --- | --- | --- | --- | --- | --- | --- | --- | --- | --- | --- | --- | --- | --- | --- | --- | --- | --- | --- | --- | --- | --- | --- | --- | --- | --- | --- | --- | --- | --- | --- | --- | --- | --- | --- | --- | --- | --- | --- | --- | --- | --- | --- | --- | --- | --- | --- | --- | --- | --- | --- | --- | --- | --- | --- | --- | --- | --- | --- | --- | --- | --- | --- | --- | --- | --- | --- | --- | --- | --- | --- | --- | --- | --- | --- | --- | --- | --- | --- | --- | --- | --- | --- | --- | --- | --- | --- | --- | --- | --- | --- | --- | --- | --- | --- | --- | --- | --- | --- | --- | --- | --- | --- | --- | --- | --- | --- | --- | --- | --- | --- | --- | --- | --- | --- | --- | --- | --- | --- | --- | --- | --- | --- | --- | --- | --- | --- | --- | --- | --- | --- | --- | --- | --- | --- | --- | --- | --- | --- | --- | --- | --- | --- | --- | --- | --- | --- | --- | --- | --- | --- | --- | --- | --- | --- | --- | --- | --- | --- | --- | --- | --- | --- | --- | --- | --- | --- | --- | --- | --- | --- | --- | --- | --- | --- | --- | --- | --- | --- | --- | --- | --- | --- | --- | --- | --- | --- | --- | --- | --- | --- | --- | --- | --- | --- | --- | --- | --- | --- | --- | --- | --- | --- | --- | --- | --- | --- | --- | --- | --- | --- | --- | --- | --- | --- | --- | --- | --- | --- | --- | --- | --- | --- | --- | --- | --- | --- | --- | --- | --- | --- | --- | --- | --- | --- | --- | --- | --- | --- | --- | --- | --- | --- | --- | --- | --- | --- | --- | --- | --- | --- | --- | --- | --- | --- | --- | --- | --- | --- | --- | --- | --- | --- | --- | --- | --- | --- | --- | --- | --- | --- | --- | --- | --- | --- | --- | --- | --- | --- | --- | --- | --- | --- | --- | --- | --- | --- | --- | --- | --- | --- | --- | --- | --- | --- | --- | --- | --- | --- | --- | --- | --- | --- | --- | --- | --- | --- | --- | --- | --- | --- | --- | --- | --- | --- | --- | --- | --- | --- | --- | --- | --- | --- | --- |
|  |  |  |  |  |  |

| Table S4: Gene names and annotations for DEGs that could be successfully annotated by EGGNOG. The full table, which includes all GO terms is available as an extra supplementary file (extra supplementary Table 1). Here we include the Query sequence, the e-value, the preferred gene name (if available), and a description of the gene. |  |  |  |  |  |
| --- | --- | --- | --- | --- | --- |
|  |  |  |  |  |  |
| \| **Query** \| **e-value** \| **Preferred name** \| **Description** \| \| --- \| --- \| --- \| --- \| \| NC_048565.1:4688695-4703383_19 \| 8.92E-158 \| KLHL30 \| Kelch-like 30 (Drosophila) \| \| NC_048565.1:14192467-14216596_30 \| 0.00E+00 \| SAMD9L \| Sterile alpha motif domain-containing protein \| \| NC_048565.1:20950107-20986095_5 \| 0.00E+00 \| - \| K02A2.6-like \| \| NC_048565.1:21271127-21303122_42 \| 0.00E+00 \| CILP2 \| Cartilage intermediate layer protein \| \| NC_048565.1:24596021-24602486_8 \| 6.00E-21 \| CIAPIN1 \| Component of the cytosolic iron-sulfur (Fe-S) protein assembly (CIA) machinery. Required for the maturation of extramitochondrial Fe-S proteins. Part of an electron transfer chain functioning in an early step of cytosolic Fe-S biogenesis. Electrons are transferred to the Fe-S cluster from NADPH via the FAD- and FMN-containing protein NDOR1. Has anti-apoptotic effects in the cell. Involved in negative control of cell death upon cytokine withdrawal. Promotes development of hematopoietic cells \| \| NC_048565.1:31980285-31989249_6 \| 4.50E-30 \| - \| Belongs to the isocitrate and isopropylmalate dehydrogenases family \| \| NC_048565.1:37212420-37248824_25 \| 1.12E-80 \| - \| retrotransposable element Tf2 155 kDa protein type 1-like \| \| NC_048565.1:52904644-52925634_4 \| 5.37E-76 \| - \| Transposase \| \| NC_048565.1:85358222-85624802_174 \| 0.00E+00 \| - \| K02A2.6-like \| \| NC_048565.1:90616647-90639542_12 \| 2.89E-54 \| - \| Myosin heavy chain, fast skeletal muscle-like \| \| NC_048565.1:90645978-90657019_16 \| 4.62E-56 \| - \| Myosin heavy chain, fast skeletal muscle-like \| \| NC_048565.1:90749275-90811956_33 \| 8.92E-57 \| - \| Myosin heavy chain, fast skeletal muscle-like \| \| NC_048566.1:5480975-5573038_50 \| 1.68E-27 \| PTP4A2 \| Protein tyrosine phosphatase type IVA \| \| NC_048566.1:11227497-11243302_17 \| 2.18E-26 \| FBXO32 \| F-box protein 32 \| \| NC_048566.1:17455727-17484284_24 \| 0.00E+00 \| ash1l \| Histone-lysine N-methyltransferase ASH1L \| \| NC_048566.1:18255918-18265786_7 \| 6.20E-35 \| CLK3 \| protein serine/threonine/tyrosine kinase activity \| \| NC_048566.1:19484328-19499142_19 \| 6.24E-72 \| CACNG6 \| Calcium channel, voltage-dependent, gamma subunit \| \| NC_048566.1:23246552-23251865_6 \| 5.04E-106 \| - \| Heat shock cognate \| \| NC_048566.1:32550907-32568101_6 \| 9.78E-79 \| - \| K02A2.6-like \| \| NC_048566.1:38963868-38966234_2 \| 9.00E-30 \| PPP1R8 \| protein serine/threonine phosphatase inhibitor activity \| \| NC_048566.1:51374969-51407238_17 \| 7.65E-190 \| - \| Transposase \| \| NC_048566.1:52903191-53021028_118 \| 0.00E+00 \| CSPG4 \| Chondroitin sulfate proteoglycan \| \| NC_048566.1:54852812-54944780_34 \| 4.96E-81 \| - \| Transposase \| \| NC_048566.1:56835034-56838853_3 \| 3.15E-17 \| C11orf58 \| Chromosome 11 open reading frame 58 \| \| NC_048566.1:67181100-67214386_54 \| 2.23E-172 \| - \| Transposase \| \| NC_048566.1:68950483-68977783_5 \| 2.48E-125 \| - \| Transposase \| \| NC_048566.1:73056128-73067792_12 \| 7.22E-14 \| TPM3 \| Tropomyosin \| \| NC_048566.1:88363311-88389528_6 \| 0.00E+00 \| GTF2IRD2B \| general transcription factor II-I repeat domain-containing protein \| \| NC_048566.1:89740189-89748145_8 \| 2.93E-35 \| - \| Transposase \| \| NC_048566.1:94150431-94173124_30 \| 6.95E-62 \| - \| Transposase \| \| NC_048567.1:7084606-7097131_2 \| 1.31E-274 \| ZNF687 \| Zinc finger protein \| \| NC_048567.1:10077928-10103975_16 \| 9.43E-12 \| RBFOX1 \| regulation of RNA splicing \| \| NC_048567.1:10180438-10226398_3 \| 4.32E-164 \| - \| Transposase \| \| NC_048567.1:11365423-11368231_3 \| 9.44E-13 \| GABARAPL1 \| GABA(A) receptor-associated protein like 1 \| \| NC_048567.1:23144162-23192782_47 \| 6.87E-82 \| - \| Transposase \| \| NC_048567.1:26533644-26536641_1 \| 4.48E-163 \| MGAT1 \| Mannosyl (alpha-1,3-)-glycoprotein beta-1,2-N-acetylglucosaminyltransferase a \| \| NC_048567.1:26653418-26693715_18 \| 5.08E-79 \| - \| Transposase \| \| NC_048567.1:27705985-27723794_5 \| 3.63E-225 \| - \| Transposase \| \| NC_048567.1:31562384-31631107_107 \| 5.45E-69 \| - \| Transposase \| \| NC_048567.1:35119498-35153277_31 \| 3.27E-159 \| - \| Transposase \| \| NC_048567.1:48645110-48667199_17 \| 0.00E+00 \| xirp2 \| Xin actin-binding \| \| NC_048567.1:51065174-51110434_64 \| 9.03E-114 \| - \| Transposase \| \| NC_048567.1:55375819-55382311_7 \| 2.47E-27 \| - \| RAB5B, member RAS oncogene family \| \| NC_048567.1:58766427-58774628_6 \| 1.70E-35 \| DHDH \| Dihydrodiol dehydrogenase (dimeric) \| \| NC_048567.1:67545597-67569008_22 \| 0.00E+00 \| - \| K02A2.6-like \| \| NC_048567.1:67660494-67881418_169 \| 1.20E-220 \| - \| Transposase \| \| NC_048567.1:83482571-83539355_68 \| 2.28E-109 \| TTLL4 \| Tubulin tyrosine ligase-like family, member 4 \| \| NC_048568.1:17935989-17938575_2 \| 0.00E+00 \| EXOC8 \| exocyst complex component \| \| NC_048568.1:18523565-18545606_2 \| 1.94E-49 \| TXLNB \| Taxilin beta a \| \| NC_048568.1:26686142-26756609_86 \| 1.75E-207 \| - \| Transposase \| \| NC_048569.1:28439062-28464098_29 \| 8.18E-114 \| GHR \| growth hormone \| \| NC_048569.1:35710205-35740468_2 \| 1.97E-75 \| - \| Transposase \| \| NC_048569.1:40902371-40915591_4 \| 2.50E-119 \| - \| Transposase \| \| NC_048569.1:42985474-42996257_7 \| 3.03E-96 \| - \| Transposase \| \| NC_048569.1:60297947-60306650_6 \| 1.08E-32 \| LPL \| The primary function of this lipase is the hydrolysis of triglycerides of circulating chylomicrons and very low density lipoproteins (VLDL). Binding to heparin sulfate proteogylcans at the cell surface is vital to the function. The apolipoprotein, APOC2, acts as a coactivator of LPL activity in the presence of lipids on the luminal surface of vascular endothelium \| \| NC_048569.1:61786447-61793654_1 \| 2.58E-48 \| MAU2 \| chromatid cohesion factor \| \| NC_048569.1:73557017-73574822_28 \| 1.67E-35 \| FBXL5 \| A Receptor for Ubiquitination Targets \| \| NC_048569.1:75219393-75230045_11 \| 3.77E-33 \| SERBP1 \| SERPINE1 mRNA binding protein 1a \| \| NC_048569.1:91572504-91663725_101 \| 5.94E-221 \| - \| Transposase \| \| NC_048569.1:95894849-95913833_9 \| 2.87E-25 \| ATG4B \| Cysteine protease required for the cytoplasm to vacuole transport (Cvt) and autophagy \| \| NC_048570.1:6750290-6771496_15 \| 9.98E-56 \| - \| Myosin heavy chain, fast skeletal muscle-like \| \| NC_048570.1:21031947-21035596_5 \| 4.52E-59 \| TOR2A \| Torsin family 2, member A \| \| NC_048570.1:26404186-26455420_3 \| 0.00E+00 \| IGSF9 \| Protein turtle homolog A-like \| \| NC_048570.1:29706053-29733234_25 \| 3.77E-62 \| CLIP1 \| linker protein \| \| NC_048570.1:33099066-33103756_4 \| 1.41E-13 \| - \| acute-phase response \| \| NC_048570.1:43939847-43942775_1 \| 5.80E-32 \| PRODH \| Proline dehydrogenase \| \| NC_048570.1:45655985-45743145_19 \| 3.70E-178 \| - \| Transposase \| \| NC_048570.1:46542604-46568019_8 \| 3.90E-37 \| - \| Transposase \| \| NC_048570.1:49897425-49928011_16 \| 8.79E-33 \| - \| Ribonuclease H protein \| \| NC_048570.1:58110966-58136498_44 \| 5.03E-198 \| - \| Transposase \| \| NC_048570.1:63953033-64013404_77 \| 1.66E-86 \| - \| K02A2.6-like \| \| NC_048570.1:65774787-65792682_24 \| 2.42E-32 \| - \| proton channel activity \| \| NC_048570.1:66085119-66098457_22 \| 3.11E-62 \| BTBD10 \| BTB (POZ) domain containing \| \| NC_048570.1:69222119-69229937_1 \| 2.32E-30 \| RHCG \| Belongs to the ammonium transporter (TC 2.A.49) family. Rh subfamily \| \| NC_048570.1:74345334-74378084_57 \| 1.78E-57 \| - \| Transposase \| \| NC_048570.1:75459659-75468648_14 \| 4.76E-44 \| HEATR3 \| HEAT repeat containing 3 \| \| NC_048570.1:90675451-90699430_28 \| 1.30E-23 \| CARS \| cysteinyl-tRNA synthetase \| \| NC_048571.1:1326442-1333881_12 \| 4.71E-13 \| SYAP1 \| Synapse associated protein 1 \| \| NC_048571.1:12213138-12256123_38 \| 4.92E-127 \| - \| Transposase \| \| NC_048571.1:17468347-17473938_6 \| 1.35E-50 \| ATP5B \| proton-transporting ATP synthase activity, rotational mechanism \| \| NC_048571.1:33325094-33362244_10 \| 1.14E-131 \| OSER1 \| Oxidative stress responsive serine-rich 1 \| \| NC_048571.1:44577147-44586619_12 \| 4.76E-72 \| RPN2 \| Ribophorin II \| \| NC_048571.1:47302690-47337234_13 \| 6.92E-135 \| GRM6 \| glutamate receptor \| \| NC_048571.1:48861825-48874458_24 \| 2.95E-58 \| UQCRC1 \| Ubiquinol-cytochrome c reductase core protein I \| \| NC_048571.1:58470112-58500215_38 \| 3.13E-43 \| CAPZB \| Capping protein (actin filament) muscle Z-line, beta \| \| NC_048571.1:63464383-63472404_9 \| 1.88E-24 \| - \| and coiled-coil \| \| NC_048571.1:67102102-67112373_1 \| 4.88E-55 \| P4HTM \| Prolyl 4-hydroxylase, transmembrane (endoplasmic reticulum) \| \| NC_048571.1:67122191-67132051_3 \| 1.89E-222 \| KBTBD12 \| Kelch repeat and BTB \| \| NC_048571.1:67855575-67864418_7 \| 7.56E-73 \| CAV3 \| May act as a scaffolding protein within caveolar membranes. Interacts directly with G-protein alpha subunits and can functionally regulate their activity \| \| NC_048571.1:74917716-74923212_4 \| 9.05E-85 \| GIMAP8 \| GTP binding \| \| NC_048571.1:75589846-75640265_13 \| 5.26E-121 \| - \| Transposase \| \| NC_048571.1:79389098-79421631_16 \| 2.28E-172 \| - \| Reverse transcriptase (RNA-dependent DNA polymerase) \| \| NC_048571.1:81606542-81652941_14 \| 6.12E-49 \| SLC6A6 \| neurotransmitter:sodium symporter activity \| \| NC_048571.1:87341189-87342665_1 \| 3.40E-127 \| CCER1 \| Wu fb55g09 \| \| NC_048571.1:87476350-87554717_35 \| 2.36E-107 \| - \| Transposase \| \| NC_048571.1:88981718-89115525_112 \| 2.67E-227 \| - \| Transposase \| \| NC_048572.1:22532673-22562329_18 \| 3.90E-73 \| - \| Transposase \| \| NC_048572.1:23585720-23615761_44 \| 7.67E-50 \| ADCK3 \| aarF domain containing kinase 3 \| \| NC_048572.1:31381107-31393074_2 \| 8.26E-141 \| DDHD1 \| DDHD domain containing 1b \| \| NC_048572.1:34374736-34394314_15 \| 3.68E-87 \| SLC25A29 \| Solute carrier family 25 (mitochondrial carnitine acylcarnitine carrier), member 29 \| \| NC_048572.1:35329638-35340474_1 \| 1.73E-182 \| - \| Transposase \| \| NC_048572.1:45509081-45546118_27 \| 6.04e-309 \| - \| K02A2.6-like \| \| NC_048572.1:51498295-51514538_2 \| 4.32E-86 \| - \| Transposase \| \| NC_048572.1:51741978-51751734_12 \| 1.11E-30 \| - \| Reverse transcriptase (RNA-dependent DNA polymerase) \| \| NC_048572.1:51795064-51805210_10 \| 1.44E-99 \| - \| Y L amino acid transporter 2-like \| \| NC_048572.1:52019649-52023960_4 \| 3.62E-27 \| SEC22B \| SEC22 vesicle trafficking protein homolog B (S. cerevisiae) \| \| NC_048572.1:57339585-57361813_4 \| 1.25E-62 \| - \| Transposase \| \| NC_048572.1:60300329-60321481_26 \| 1.22E-111 \| - \| Reverse transcriptase (RNA-dependent DNA polymerase) \| \| NC_048572.1:73692300-73696215_6 \| 6.29E-174 \| MYOC \| Myocilin \| \| NC_048572.1:74549831-74624237_17 \| 0.00E+00 \| - \| K02A2.6-like \| \| NC_048572.1:77894988-77919569_9 \| 1.20E-221 \| - \| K02A2.6-like \| \| NC_048572.1:85364733-85412816_14 \| 9.85E-196 \| - \| Reverse transcriptase (RNA-dependent DNA polymerase) \| \| NC_048572.1:85511382-85525390_6 \| 1.31E-97 \| - \| Kelch-like 40a (Drosophila) \| \| NC_048572.1:89967407-89974406_9 \| 1.20E-44 \| ACADM \| acyl-Coenzyme A dehydrogenase, C-4 to C-12 straight chain \| \| NC_048573.1:19017541-19030578_25 \| 9.69E-19 \| FXR2 \| negative regulation of translation \| \| NC_048573.1:25244197-25262210_1 \| 8.83E-40 \| CPZ \| Carboxypeptidase \| \| NC_048573.1:25707950-25742721_30 \| 0.00E+00 \| - \| K02A2.6-like \| \| NC_048573.1:27633636-27647745_6 \| 1.63E-121 \| - \| Transposase \| \| NC_048573.1:27915616-28099295_33 \| 0.00E+00 \| - \| K02A2.6-like \| \| NC_048573.1:39640879-39675631_48 \| 4.05E-215 \| - \| Transposase \| \| NC_048573.1:40844047-40866839_32 \| 1.24E-38 \| - \| Transposase \| \| NC_048573.1:42021496-42027071_6 \| 1.99E-127 \| - \| Grass carp reovirus (GCRV)-induced gene 2o \| \| NC_048573.1:42871497-42897897_20 \| 3.19E-190 \| - \| Transposase \| \| NC_048573.1:52452691-52473256_7 \| 1.58E-26 \| CSDE1 \| Cold shock domain-containing protein E1 \| \| NC_048573.1:68151271-68179048_10 \| 1.16E-28 \| - \| Transposase \| \| NC_048574.1:5729926-5860268_4 \| 0.00E+00 \| - \| Belongs to the TRAFAC class TrmE-Era-EngA-EngB-Septin- like GTPase superfamily. Septin GTPase family \| \| NC_048574.1:9275315-9282255_11 \| 1.63E-37 \| - \| Zgc 110540 \| \| NC_048574.1:13513408-13569059_80 \| 1.12E-229 \| SH3PXD2B \| SH3 and PX \| \| NC_048574.1:21542000-21564106_19 \| 3.79E-41 \| - \| Transposase \| \| NC_048574.1:33257903-33318427_39 \| 8.04E-69 \| - \| Transposase \| \| NC_048574.1:36104781-36117760_2 \| 0.00E+00 \| ERCC6L \| Excision repair cross-complementing rodent repair deficiency, complementation group 6-like \| \| NC_048574.1:39981706-40090045_12 \| 4.77E-134 \| MYO18A \| Belongs to the TRAFAC class myosin-kinesin ATPase superfamily. Myosin family \| \| NC_048574.1:45003077-45011000_15 \| 1.59E-19 \| - \| U2 snRNP-associated SURP \| \| NC_048574.1:47388839-47403411_27 \| 1.24E-92 \| - \| Transposase \| \| NC_048574.1:48354636-48393373_18 \| 6.49E-225 \| - \| Transposase \| \| NC_048574.1:61517745-61529376_10 \| 3.95E-40 \| - \| Transposase \| \| NC_048574.1:61807156-61821865_12 \| 0.00E+00 \| - \| K02A2.6-like \| \| NC_048574.1:69209181-69221379_5 \| 9.65E-67 \| - \| Transposase \| \| NC_048575.1:3683874-3959282_143 \| 6.21E-215 \| - \| Reverse transcriptase (RNA-dependent DNA polymerase) \| \| NC_048575.1:7705907-7719204_13 \| 2.28E-19 \| - \| Reverse transcriptase (RNA-dependent DNA polymerase) \| \| NC_048575.1:20130518-20133776_2 \| 4.22E-27 \| TIMM21 \| Translocase of inner mitochondrial membrane 21 homolog (yeast) \| \| NC_048575.1:23635854-23661667_43 \| 8.25E-169 \| - \| Transposase \| \| NC_048575.1:36947255-37051787_36 \| 0.00E+00 \| - \| K02A2.6-like \| \| NC_048575.1:51492045-51523279_33 \| 3.14E-299 \| SPECC1L \| Sperm antigen with calponin homology and coiled-coil domains \| \| NC_048575.1:55895074-55905515_3 \| 1.47E-53 \| STC1 \| Stanniocalcin 1 \| \| NC_048575.1:66500422-66510614_6 \| 1.32E-68 \| - \| Transposase \| \| NC_048575.1:75399995-75403338_1 \| 1.28E-87 \| XBP1 \| X-box binding protein 1 \| \| NC_048575.1:77700776-77725548_21 \| 6.05E-23 \| ERAP1 \| Endoplasmic reticulum aminopeptidase \| \| NC_048575.1:81558951-81571599_17 \| 2.93E-46 \| FST \| Follistatin \| \| NC_048575.1:83341427-83361014_7 \| 5.04E-73 \| EEF2 \| Elongation factor \| \| NC_048575.1:83634384-83698037_9 \| 6.62E-106 \| - \| Reverse transcriptase (RNA-dependent DNA polymerase) \| \| NC_048576.1:7912754-7972543_19 \| 5.62E-229 \| - \| Transposase \| \| NC_048576.1:19088756-19100725_10 \| 3.69E-59 \| AGPAT9 \| Glycerol-3-phosphate acyltransferase \| \| NC_048576.1:27834303-27850140_22 \| 6.47E-208 \| - \| Transposase \| \| NC_048576.1:31905481-31916227_12 \| 4.92E-48 \| - \| Transposase \| \| NC_048576.1:44220981-44225623_5 \| 6.62E-28 \| CKM \| Creatine kinase \| \| NC_048576.1:49167549-49208229_37 \| 0.00E+00 \| - \| K02A2.6-like \| \| NC_048576.1:49208284-49209887_2 \| 6.70E-112 \| - \| Transposase \| \| NC_048576.1:61724768-61753382_38 \| 1.45E-38 \| ATP2A1 \| ATPase, Ca transporting, cardiac muscle, fast twitch 1 \| \| NC_048576.1:63298745-63333492_13 \| 2.27E-273 \| - \| Reverse transcriptase (RNA-dependent DNA polymerase) \| \| NC_048576.1:69659317-69683445_27 \| 2.25E-203 \| - \| Transposase \| \| NC_048576.1:70034403-70046187_23 \| 1.18E-32 \| - \| DDE superfamily endonuclease \| \| NC_048576.1:71411522-71420923_5 \| 3.36E-32 \| arf-1.2 \| GTP binding \| \| NC_048576.1:75631055-75637902_5 \| 1.78E-241 \| TOB2 \| Transducer of ERBB2, 2 \| \| NC_048576.1:77109774-77121214_12 \| 9.01E-36 \| GCDH \| glutaryl-Coenzyme A dehydrogenase \| \| NC_048577.1:3533508-3554061_8 \| 4.69E-43 \| - \| RNA recognition motif \| \| NC_048577.1:12596679-12604679_5 \| 1.95E-65 \| ATG4D \| Cysteine protease required for the cytoplasm to vacuole transport (Cvt) and autophagy \| \| NC_048577.1:13665401-13672204_5 \| 1.07E-56 \| ASNA1 \| ATPase required for the post-translational delivery of tail-anchored (TA) proteins to the endoplasmic reticulum. Recognizes and selectively binds the transmembrane domain of TA proteins in the cytosol. This complex then targets to the endoplasmic reticulum by membrane-bound receptors, where the tail- anchored protein is released for insertion. This process is regulated by ATP binding and hydrolysis. ATP binding drives the homodimer towards the closed dimer state, facilitating recognition of newly synthesized TA membrane proteins. ATP hydrolysis is required for insertion. Subsequently, the homodimer reverts towards the open dimer state, lowering its affinity for the membrane-bound receptor, and returning it to the cytosol to initiate a new round of targeting \| \| NC_048577.1:20230786-20244551_25 \| 1.55E-25 \| - \| Transposase \| \| NC_048577.1:21072151-21099165_38 \| 1.04E-197 \| - \| Transposase \| \| NC_048577.1:31592188-31609185_14 \| 2.53E-222 \| - \| Transposase \| \| NC_048577.1:36412876-36443066_17 \| 1.28E-43 \| - \| DDE superfamily endonuclease \| \| NC_048577.1:41065494-41087292_24 \| 4.81E-28 \| - \| Transposase \| \| NC_048577.1:44021754-44026664_7 \| 1.15E-119 \| EPOR \| Erythropoietin receptor \| \| NC_048577.1:47460375-47465152_3 \| 4.26E-27 \| PCTP \| Phosphatidylcholine transfer \| \| NC_048577.1:48705713-48724776_7 \| 2.95E-221 \| - \| Transposase \| \| NC_048577.1:51405257-51412420_4 \| 2.15E-179 \| - \| Transposase \| \| NC_048578.1:3242450-3275774_22 \| 0.00E+00 \| - \| K02A2.6-like \| \| NC_048578.1:6039164-6125852_25 \| 0.00E+00 \| - \| K02A2.6-like \| \| NC_048578.1:17395671-17399118_2 \| 4.59E-32 \| ASB5 \| Ankyrin repeat and SOCS \| \| NC_048578.1:27502546-27535883_2 \| 2.63E-62 \| SLC16A2 \| Solute carrier family 16 (monocarboxylic acid transporters), member 2 \| \| NC_048579.1:1020193-1040975_31 \| 2.14E-149 \| KLHL40 \| Kelch-like \| \| NC_048579.1:1130239-1193950_38 \| 0.00E+00 \| - \| K02A2.6-like \| \| NC_048579.1:4154252-4167691_8 \| 9.94E-59 \| - \| Ribonuclease H protein \| \| NC_048579.1:16206792-16219123_18 \| 7.22E-90 \| - \| Transposase \| \| NC_048579.1:17207282-17226440_10 \| 1.26E-91 \| - \| Transposase \| \| NC_048579.1:28246478-28316514_25 \| 7.02E-34 \| MYOM1 \| Immunoglobulin like \| \| NC_048579.1:31237594-31287177_33 \| 3.51E-43 \| - \| Transposase \| \| NC_048579.1:43641342-43692459_37 \| 1.44E-206 \| - \| Transposase \| \| NC_048579.1:48768295-48775451_6 \| 7.16E-22 \| DENND6B \| DENN (AEX-3) domain \| \| NC_048579.1:57738534-57739096_1 \| 1.59E-36 \| - \| - \| \| NC_048579.1:57959580-57965064_7 \| 5.29E-96 \| - \| Bcl-2-like protein \| \| NC_048579.1:69083191-69087692_6 \| 1.07E-12 \| UBE2H \| ubiquitin-conjugating enzyme \| \| NC_048579.1:72421569-72426957_1 \| 2.27E-107 \| FGL2 \| Fibrinogen-like 2 \| \| NC_048580.1:8170589-8191496_14 \| 1.11E-55 \| - \| Ribonuclease H protein \| \| NC_048580.1:7238568-7253802_14 \| 1.89E-36 \| WDR92 \| Protein phosphatase 3 (formerly 2B), regulatory s1ubunit B, alpha isoform, b \| \| NC_048580.1:12274801-12279770_3 \| 3.21E-290 \| - \| PRY \| \| NC_048580.1:13686240-13697502_6 \| 4.20E-73 \| PANK1 \| Pantothenate kinase \| \| NC_048580.1:20679050-20681235_4 \| 1.60E-22 \| C16orf93 \| Chromosome 16 open reading frame 93 \| \| NC_048580.1:23053857-23070702_1 \| 9.57E-44 \| - \| Reverse transcriptase (RNA-dependent DNA polymerase) \| \| NC_048580.1:27645464-27661310_15 \| 1.32E-59 \| ATP2A2 \| positive regulation of endoplasmic reticulum calcium ion concentration \| \| NC_048580.1:32435645-32462806_14 \| 6.23E-48 \| - \| Transposase \| \| NC_048580.1:34055773-34064383_4 \| 1.06E-26 \| - \| ionotropic glutamate receptor activity \| \| NC_048580.1:40527927-40614259_106 \| 2.46E-82 \| - \| Transposase \| \| NC_048580.1:42335687-42364396_31 \| 7.60E-38 \| - \| Ribonuclease H protein \| \| NC_048580.1:43578536-43599139_32 \| 1.09E-119 \| - \| Transposase \| \| NC_048580.1:45562469-45569742_3 \| 1.21E-16 \| RACGAP1 \| Rac GTPase-activating protein \| \| NC_048580.1:45617125-45627801_10 \| 3.50E-96 \| - \| Transposase \| \| NC_048580.1:45885240-45909662_22 \| 2.54E-52 \| - \| Ribonuclease H protein \| \| NC_048580.1:49066331-49076265_5 \| 2.11E-29 \| - \| Transposase \| \| NC_048580.1:49745046-49778434_22 \| 8.80E-226 \| - \| Si dkey-65j6.2 \| \| NC_048580.1:52649499-52687625_18 \| 4.48E-124 \| - \| Transposase \| \| NC_048580.1:52880607-52888252_9 \| 4.58E-29 \| BGN \| May be involved in collagen fiber assembly \| \| NC_048580.1:55755130-55769029_8 \| 0.00E+00 \| - \| K02A2.6-like \| \| NC_048580.1:56240389-56284222_67 \| 7.39E-188 \| ADCY6 \| Belongs to the adenylyl cyclase class-4 guanylyl cyclase family \| \| NC_048580.1:56424187-56438268_6 \| 4.83E-47 \| NACA \| Nascent polypeptide-associated complex \| \| NC_048580.1:64391607-64393468_2 \| 1.78E-26 \| KBTBD12 \| Kelch repeat and BTB \| \| NC_048580.1:66320422-66345643_16 \| 6.21E-107 \| - \| Transposase \| \| NC_048581.1:49757-55991_3 \| 2.92E-26 \| MFAP4 \| Microfibrillar-associated protein 4 \| \| NC_048581.1:15850674-15871731_4 \| 1.79E-111 \| SLC25A28 \| Solute carrier family 25 (mitochondrial iron transporter), member 28 \| \| NC_048581.1:15947058-15974559_33 \| 4.08E-87 \| - \| Transposase \| \| NC_048581.1:21052932-21056639_2 \| 3.68E-153 \| - \| basic region leucin zipper \| \| NC_048581.1:22482938-22507147_32 \| 1.23E-118 \| - \| Transposase \| \| NC_048581.1:28859182-28886611_9 \| 1.52E-56 \| - \| Ribonuclease H protein \| \| NC_048581.1:34290017-34317682_22 \| 0.00E+00 \| GTF2IRD2B \| general transcription factor II-I repeat domain-containing protein \| \| NC_048581.1:42779461-42794918_3 \| 1.36E-42 \| - \| Transposase \| \| NC_048581.1:54717983-54725611_9 \| 6.15E-225 \| KBTBD12 \| Kelch repeat and BTB \| \| NC_048581.1:56747847-56786071_31 \| 1.16E-184 \| - \| Transposase \| \| NC_048581.1:61014725-61018743_1 \| 4.99E-19 \| BRK1 \| BRICK1, SCAR WAVE actin-nucleating complex subunit \| \| NC_048581.1:64043155-64052863_8 \| 1.05E-94 \| VWA1 \| Von Willebrand factor A \| \| NC_048581.1:66566779-66691051_165 \| 7.78E-148 \| - \| Transposase \| \| NC_048581.1:68895720-68928184_28 \| 2.90E-119 \| - \| Reverse transcriptase (RNA-dependent DNA polymerase) \| \| NC_048581.1:73226757-73251162_14 \| 1.99E-43 \| - \| Reverse transcriptase (RNA-dependent DNA polymerase) \| \| NC_048581.1:74877193-74921632_10 \| 1.72E-83 \| NFIC \| Recognizes and binds the palindromic sequence 5'- TTGGCNNNNNGCCAA-3' present in viral and cellular promoters and in the origin of replication of adenovirus type 2. These proteins are individually capable of activating transcription and replication \| \| NC_048581.1:82818900-82849983_2 \| 3.65E-49 \| IGFN1 \| Immunoglobulin-like and fibronectin type III \| \| NC_048581.1:88877784-88897712_13 \| 9.01E-96 \| CAND2 \| Cullin-associated and neddylation-dissociated 2 \| \| NC_048581.1:93531622-93561122_30 \| 2.32E-26 \| - \| Transposase \| \| NC_048582.1:6118562-6145394_24 \| 1.24E-210 \| - \| Reverse transcriptase (RNA-dependent DNA polymerase) \| \| NC_048582.1:12225385-12229990_3 \| 2.20E-50 \| IQCB1 \| IQ motif containing B1 \| \| NC_048582.1:20251273-20255220_7 \| 1.49E-231 \| KLHL41 \| Kelch-like \| \| NC_048582.1:23764573-23779948_14 \| 5.36E-89 \| MARCH7 \| Membrane-associated ring finger (C3HC4) 7 \| \| NC_048582.1:34645116-34704821_40 \| 6.47E-189 \| - \| Transposase \| \| NC_048582.1:37975556-37987864_8 \| 4.74E-80 \| - \| Kelch-like \| \| NC_048582.1:39084396-39114484_14 \| 1.42E-50 \| - \| Transposase \| \| NC_048582.1:40280292-40369178_7 \| 2.06E-202 \| - \| Transposase \| \| NC_048582.1:48837352-48851235_19 \| 3.69E-70 \| - \| Ribonuclease H protein \| \| NC_048582.1:54022282-54037508_17 \| 1.46E-31 \| GMPR \| Catalyzes the irreversible NADPH-dependent deamination of GMP to IMP. It functions in the conversion of nucleobase, nucleoside and nucleotide derivatives of G to A nucleotides, and in maintaining the intracellular balance of A and G nucleotides \| \| NC_048583.1:17645109-17682190_28 \| 4.07E-40 \| - \| Transposase \| \| NC_048583.1:19218449-19221372_7 \| 3.28E-66 \| CCDC86 \| Coiled-coil domain containing 86 \| \| NC_048583.1:26228684-26237366_3 \| 2.33E-197 \| - \| Transposase \| \| NC_048583.1:26864882-26872914_3 \| 0.00E+00 \| FBXO30 \| F-box protein 30a \| \| NC_048583.1:33975022-34009037_26 \| 7.79E-211 \| - \| Transposase \| \| NC_048583.1:35340536-35346492_2 \| 3.07E-19 \| - \| Ribonuclease H protein \| \| NC_048583.1:39293829-39297981_2 \| 4.62E-51 \| CRLS1 \| Cardiolipin synthase \| \| NC_048583.1:42399916-42414821_16 \| 1.99E-81 \| - \| Transposase \| \| NC_048583.1:47514824-47519991_2 \| 5.43E-146 \| PAX1 \| Paired box \| \| NC_048583.1:53259730-53342223_27 \| 4.94E-40 \| - \| Transposase \| \| NC_048584.1:2931410-3043226_123 \| 0.00E+00 \| - \| K02A2.6-like \| \| NC_048584.1:12693022-12728120_20 \| 1.05E-218 \| - \| Transposase \| \| NC_048584.1:23509632-23518631_7 \| 2.66E-24 \| - \| Transposase \| \| NC_048584.1:24286893-24404097_88 \| 0.00E+00 \| - \| K02A2.6-like \| \| NC_048584.1:32747591-32760073_13 \| 8.51E-111 \| SLC25A28 \| Belongs to the mitochondrial carrier (TC 2.A.29) family \| \| NC_048584.1:34569055-34574055_2 \| 2.52E-256 \| KLHL31 \| Kelch-like 31 (Drosophila) \| \| NC_048584.1:35272289-35311244_22 \| 3.66E-219 \| - \| Transposase \| \| NC_048585.1:20109070-20114322_5 \| 2.97E-79 \| - \| Transposase \| \| NC_048585.1:27890465-27902897_15 \| 5.28E-41 \| - \| Transposase \| \| NC_048585.1:41888787-41919352_16 \| 8.50E-39 \| ACTN3 \| Ca2+ insensitive EF hand \| \| NC_048585.1:53268513-53328874_65 \| 1.01E-156 \| EHBP1L1 \| EH domain binding protein 1-like 1 \| \| NC_048585.1:60333055-60343162_7 \| 3.75E-28 \| TMEM179B \| Transmembrane protein 179B \| \| NC_048585.1:61909911-61999755_114 \| 6.87E-44 \| ENDOD1 \| Endonuclease domain containing 1 \| \| NC_048585.1:63363590-63372541_5 \| 4.47E-30 \| - \| C-type natriuretic peptide 2-like \| \| NC_048585.1:63893194-64065840_136 \| 1.11E-210 \| - \| Transposase \| \| NC_048586.1:15041843-15065948_17 \| 1.28E-19 \| SLC25A12 \| Solute carrier family 25 \| \| NC_048586.1:27186245-27204132_24 \| 1.91E-211 \| - \| Transposase \| \| NC_048586.1:36405741-36434947_37 \| 2.29E-191 \| - \| Transposase \| \| NC_048586.1:36435000-36443179_1 \| 5.83E-62 \| DIRC2 \| Disrupted in renal carcinoma \| \| NC_048586.1:38491565-38519642_29 \| 3.68E-70 \| - \| Reverse transcriptase (RNA-dependent DNA polymerase) \| \| NC_048586.1:39575885-39680209_130 \| 1.24E-176 \| - \| Transposase \| \| NC_048587.1:2313758-2316818_2 \| 1.37E-199 \| EGR2 \| Early growth response \| \| NC_048587.1:9161392-9174448_7 \| 1.65E-105 \| - \| Transposase \| \| NC_048587.1:8850743-8858627_15 \| 3.14E-54 \| - \| Myosin heavy chain, fast skeletal muscle-like \| \| NC_048587.1:38437233-38439848_2 \| 5.72E-20 \| GSTA4 \| Glutathione S-transferase \| \| NC_048587.1:46448825-46459591_4 \| 3.87E-54 \| KDELR2 \| KDEL (Lys-Asp-Glu-Leu) endoplasmic reticulum protein retention receptor \| \| NC_048587.1:46608174-46631675_24 \| 7.29E-198 \| - \| Transposase \| \| NC_048587.1:47159772-47174198_25 \| 2.78E-38 \| KCTD17 \| Potassium channel tetramerization domain containing 17 \| \| NC_048587.1:47471182-47501401_22 \| 7.49E-99 \| - \| Reverse transcriptase (RNA-dependent DNA polymerase) \| \| NC_048587.1:62491785-62570675_27 \| 3.11E-55 \| - \| Ribonuclease H protein \| \| NC_048588.1:7373245-7404005_10 \| 2.79E-71 \| USP2 \| Ubiquitin carboxyl-terminal hydrolase 2-like isoform X1 \| \| NC_048588.1:13475209-13549653_81 \| 2.31E-182 \| - \| Transposase \| \| NC_048588.1:13666297-13680079_17 \| 3.12E-112 \| - \| Transposase \| \| NC_048589.1:4666657-4711974_27 \| 9.86E-53 \| - \| Transposase \| \| NC_048589.1:11554491-11605442_30 \| 2.43E-79 \| - \| Transposase \| \| NC_048589.1:16120039-16130534_5 \| 7.95E-157 \| - \| Transposase \| \| NC_048589.1:22498171-22500337_2 \| 1.13E-139 \| SIX1 \| Sine oculis homeobox homolog \| \| NC_048589.1:22704448-22726005_1 \| 0.00E+00 \| - \| Sacsin-like \| \| NC_048589.1:29342451-29405608_53 \| 3.02E-181 \| ANKRD9 \| ankyrin repeat domain-containing protein \| \| NC_048590.1:3419580-3477104_34 \| 4.38E-44 \| - \| Transposase \| \| NC_048590.1:6099213-6129024_2 \| 2.92E-119 \| WHAMM \| WAS protein homolog associated with actin, golgi membranes and microtubules \| \| NC_048590.1:14523758-14566303_14 \| 8.14E-213 \| - \| Transposase \| \| NC_048590.1:25659579-25675669_28 \| 4.79E-30 \| AMPD1 \| Adenosine/AMP deaminase \| \| NC_048590.1:27510706-27520929_7 \| 1.79E-32 \| USP10 \| Ubiquitin carboxyl-terminal hydrolase 10 \| \| NC_048590.1:32363834-32402341_11 \| 5.34E-48 \| - \| Reverse transcriptase (RNA-dependent DNA polymerase) \| \| NC_048590.1:34604116-34636624_44 \| 1.42E-216 \| - \| Transposase \| \| NC_048590.1:50343981-50349667_1 \| 2.32E-13 \| THAP2 \| THAP domain containing \| \| NC_048591.1:7531581-7567845_42 \| 2.73E-264 \| - \| Reverse transcriptase (RNA-dependent DNA polymerase) \| \| NC_048591.1:9731766-9762748_4 \| 1.13E-162 \| - \| Transposase \| \| NC_048591.1:10062379-10120083_4 \| 1.47E-80 \| - \| K02A2.6-like \| \| NC_048591.1:10236369-10250644_17 \| 7.71E-21 \| POLDIP2 \| Polymerase (DNA-directed), delta interacting protein 2 \| \| NC_048592.1:3309888-3315319_5 \| 1.88E-80 \| - \| ubiquitin ligase \| \| NC_048592.1:3797381-3814921_4 \| 3.16E-71 \| - \| Transposase \| \| NC_048592.1:8261156-8273529_5 \| 9.86E-22 \| TLK2 \| Serine threonine-protein kinase tousled-like \| \| NC_048592.1:8429767-8480564_49 \| 2.03E-100 \| - \| Transposase \| \| NC_048592.1:26656615-26675690_16 \| 2.40E-71 \| - \| Transposase \| \| NC_048592.1:30360185-30366670_7 \| 1.04E-24 \| CNN2 \| Thin filament-associated protein that is implicated in the regulation and modulation of smooth muscle contraction. It is capable of binding to actin, calmodulin, troponin C and tropomyosin. The interaction of calponin with actin inhibits the actomyosin Mg-ATPase activity \| \| NC_048592.1:31952199-31954935_1 \| 5.73E-92 \| CPN2 \| Carboxypeptidase N \| \| NC_048592.1:32700038-32704296_6 \| 8.48E-18 \| AHSG \| Belongs to the fetuin family \| \| NC_048592.1:39384479-39484807_36 \| 7.79E-211 \| - \| Transposase \| \| NC_048592.1:42349143-42356473_5 \| 2.70E-53 \| - \| Transposase \| \| NC_048593.1:9383513-9437443_56 \| 6.86E-186 \| - \| Reverse transcriptase (RNA-dependent DNA polymerase) \| \| NC_048593.1:11459925-11464673_2 \| 8.11E-21 \| ACADS \| acyl-Coenzyme A dehydrogenase, C-2 to C-3 short chain \| \| NC_048593.1:19563521-19599052_36 \| 5.64E-43 \| TJP2 \| Tight junction protein \| \| NC_048593.1:20970332-20977128_2 \| 1.44E-49 \| surf4 \| Surfeit locus protein \| \| NC_048593.1:23647257-23654994_9 \| 6.53E-15 \| - \| - \| \| NC_048593.1:31820484-31908551_128 \| 2.95E-215 \| RYR1 \| ryanodine receptor \| \| NC_048593.1:33605361-33651127_2 \| 2.39E-213 \| - \| Transposase \| \| NC_048593.1:35804622-35814315_5 \| 0.00E+00 \| - \| Belongs to the TRAFAC class TrmE-Era-EngA-EngB-Septin- like GTPase superfamily. Septin GTPase family \| \| NC_048593.1:36401541-36416091_8 \| 1.91E-290 \| - \| DNA polymerase type B, organellar and viral \| \| NC_050570.1:6076538-6113689_8 \| 1.66E-44 \| CLPX \| Caseinolytic mitochondrial matrix peptidase chaperone subunit \| \| NC_050570.1:11201683-11226241_18 \| 4.13E-77 \| - \| Transposase \| \| NC_050570.1:13956515-13981811_36 \| 1.93E-89 \| - \| Transposase \| \| NC_050570.1:25412313-25425239_11 \| 2.02E-20 \| - \| E3 ubiquitin-protein ligase RNF170-like \| \| NC_050570.1:27685355-27694136_6 \| 1.84E-35 \| PALLD \| Palladin, cytoskeletal associated protein \| \| NC_050570.1:44432259-44471478_17 \| 5.19E-42 \| ATP2A1 \| ATPase, Ca transporting, cardiac muscle, fast twitch 1 \| \| NC_050571.1:7663346-7683873_22 \| 1.98E-38 \| - \| Ribonuclease H protein \| \| NC_050571.1:14229946-14242895_11 \| 1.29E-61 \| - \| Ribonuclease H protein \| \| NC_050571.1:17971133-18010807_41 \| 2.43E-143 \| - \| Transposase \| \| NC_050571.1:21947801-21953127_6 \| 5.92E-134 \| - \| Transposase \| \| NC_050571.1:27175517-27191102_13 \| 6.22E-37 \| FBXW11 \| F-box and WD-40 domain protein \| \| NC_050571.1:31214900-31229343_17 \| 1.52E-26 \| - \| DDE superfamily endonuclease \| \| NC_050572.1:5452348-5468487_16 \| 5.29E-54 \| FUCA1 \| Alpha-L-fucosidase \| \| NC_050572.1:10670248-10675743_4 \| 2.45E-38 \| - \| Reverse transcriptase (RNA-dependent DNA polymerase) \| \| NC_050572.1:28036021-28083105_10 \| 9.73E-172 \| KCNG2 \| Potassium voltage-gated channel, subfamily G, member \| \| NC_050572.1:39276229-39291270_16 \| 1.30E-37 \| - \| Ribonuclease H protein \| \| NW_023493663.1:877777-937949_51 \| 1.65E-70 \| - \| Reverse transcriptase (RNA-dependent DNA polymerase) \| \| NW_023493665.1:1120371-1147001_9 \| 1.03E-91 \| - \| Transposase \| \| NW_023493714.1:215639-256995_18 \| 2.25E-35 \| - \| Nuclease-related domain \| \| NW_023493816.1:56914-97092_23 \| 9.49E-212 \| - \| Transposase \| \| NW_023494063.1:35914-36705_2 \| 2.54E-100 \| - \| Haptoglobin \| |  |  |  |  |  |
|  |  |  |  |  |  |

**Table S5:** Table of all the GO terms identified in the DEGs between the NxN and HxH offspring. The table is sorted by the count (number of occurrences) of each GO term within the annotated DEG dataset. Included is the GO ID, the corresponding name of the GO ID, the number of occurrences (Count) and the output from REVIGO including the Log Size, Frequency, Uniqueness, Dispensability, and PC coordinates used to create figure 4. All GO terms associated with the DEGs are available in Extra Supplementary Table 1.

| **GO ID** | **Name** | **Count** | **LogSize** | **Frequency** | **Unique** | **Disp.** | **PC_0** | **PC_1** |
| --- | --- | --- | --- | --- | --- | --- | --- | --- |
| GO:0008150 | biological process | 129 | 4.315 | 100.000 | 1.000 | 0.000 | -2.706 | -7.493 |
| GO:0009987 | cellular process | 117 | 4.194 | 75.678 | 1.000 | 0.000 | -5.013 | -6.663 |
| GO:0065007 | biological regulation | 93 | 4.044 | 53.560 | 1.000 | 0.000 | -1.599 | -0.663 |
| GO:0050789 | regulation of biological process | 87 | 4.030 | 51.909 | 0.927 | 0.423 | 0.925 | 1.341 |
| GO:0050794 | regulation of cellular process | 82 | 4.005 | 48.978 | 0.922 | 0.169 | 1.114 | 1.220 |
| GO:0008152 | metabolic process | 75 | 3.866 | 35.589 | 1.000 | 0.000 | -2.402 | -7.636 |
| GO:0044237 | cellular metabolic process | 73 | 3.751 | 27.282 | 0.924 | 0.309 | 2.032 | -5.776 |
| GO:0071704 | organic substance metabolic process | 72 | 3.770 | 28.497 | 0.933 | 0.521 | 2.073 | -5.878 |
| GO:0044238 | primary metabolic process | 69 | 3.744 | 26.841 | 0.934 | 0.509 | 2.080 | -5.814 |
| GO:0032501 | multicellular organismal process | 68 | 3.734 | 26.230 | 1.000 | 0.000 | -6.028 | -5.871 |
| GO:0050896 | response to stimulus | 66 | 3.786 | 29.597 | 1.000 | 0.000 | -2.518 | -6.010 |
| GO:0006807 | nitrogen compound metabolic process | 63 | 3.695 | 23.997 | 0.935 | 0.488 | 2.145 | -5.780 |
| GO:0032502 | developmental process | 63 | 3.721 | 25.470 | 1.000 | 0.000 | -5.665 | -6.171 |
| GO:0071840 | cellular component organization or biogenesis | 63 | 3.657 | 21.977 | 0.986 | 0.030 | -1.434 | 0.523 |
| GO:0016043 | cellular component organization | 62 | 3.637 | 21.018 | 0.946 | 0.519 | -4.640 | 5.646 |
| GO:0048518 | positive regulation of biological process | 61 | 3.436 | 13.210 | 0.937 | 0.230 | 1.133 | 2.250 |
| GO:0048856 | anatomical structure development | 60 | 3.703 | 24.443 | 0.905 | 0.678 | -6.822 | -0.743 |
| GO:0051179 | localization | 60 | 3.565 | 17.768 | 1.000 | 0.000 | -2.941 | -6.310 |
| GO:0019222 | regulation of metabolic process | 57 | 3.631 | 20.713 | 0.933 | 0.276 | 0.742 | 1.576 |
| GO:0007275 | multicellular organism development | 56 | 3.622 | 20.263 | 0.881 | 0.462 | -6.659 | -0.034 |
| GO:1901564 | organonitrogen compound metabolic process | 55 | 3.533 | 16.537 | 0.914 | 0.451 | 1.413 | -6.251 |
| GO:0043170 | macromolecule metabolic process | 51 | 3.616 | 20.025 | 0.921 | 0.527 | 1.721 | -6.165 |
| GO:0048522 | positive regulation of cellular process | 51 | 3.384 | 11.718 | 0.874 | 0.560 | 4.044 | -1.240 |
| GO:0006810 | transport | 49 | 3.504 | 15.472 | 0.934 | 0.626 | 4.744 | 4.227 |
| GO:0051234 | establishment of localization | 49 | 3.526 | 16.242 | 0.937 | 0.771 | null | null |
| GO:0048519 | negative regulation of biological process | 49 | 3.405 | 12.309 | 0.938 | 0.225 | 0.891 | 2.121 |
| GO:0048731 | system development | 49 | 3.554 | 17.337 | 0.873 | 0.636 | -6.662 | 0.141 |
| GO:0060255 | regulation of macromolecule metabolic process | 49 | 3.605 | 19.517 | 0.886 | 0.539 | 2.420 | 0.964 |
| GO:0065008 | regulation of biological quality | 49 | 3.161 | 7.019 | 0.947 | 0.172 | 0.028 | 1.159 |
| GO:0048869 | cellular developmental process | 47 | 3.444 | 13.466 | 0.903 | 0.566 | -6.843 | -0.850 |
| GO:0030154 | cell differentiation | 46 | 3.444 | 13.447 | 0.885 | 0.665 | -7.068 | -0.773 |
| GO:0031323 | regulation of cellular metabolic process | 46 | 3.608 | 19.623 | 0.881 | 0.765 | null | null |
| GO:0042221 | response to chemical | 45 | 3.194 | 7.562 | 0.952 | 0.303 | 0.268 | 6.268 |
| GO:0048523 | negative regulation of cellular process | 44 | 3.381 | 11.650 | 0.882 | 0.713 | null | null |
| GO:0080090 | regulation of primary metabolic process | 44 | 3.587 | 18.693 | 0.887 | 0.754 | null | null |
| GO:0051239 | regulation of multicellular organismal process | 43 | 3.005 | 4.897 | 0.945 | 0.000 | -0.432 | 1.392 |
| GO:0051171 | regulation of nitrogen compound metabolic process | 43 | 3.578 | 18.320 | 0.887 | 0.749 | null | null |
| GO:0006996 | organelle organization | 40 | 3.365 | 11.224 | 0.949 | 0.631 | -4.675 | 5.627 |
| GO:0048513 | animal organ development | 40 | 3.410 | 12.459 | 0.910 | 0.579 | -6.776 | -0.800 |
| GO:0051716 | cellular response to stimulus | 40 | 3.713 | 25.029 | 0.931 | 0.432 | 0.156 | 6.309 |
| GO:0019538 | protein metabolic process | 39 | 3.402 | 12.212 | 0.905 | 0.588 | 1.185 | -6.411 |
| GO:0006950 | response to stress | 37 | 3.180 | 7.329 | 0.952 | 0.330 | 0.275 | 6.368 |
| GO:0009058 | biosynthetic process | 37 | 3.451 | 13.670 | 0.941 | 0.405 | 2.257 | -5.617 |
| GO:0050793 | regulation of developmental process | 37 | 2.997 | 4.805 | 0.945 | 0.104 | -0.905 | 1.635 |
| GO:1901576 | organic substance biosynthetic process | 37 | 3.443 | 13.442 | 0.905 | 0.682 | 1.586 | -6.061 |
| GO:0009653 | anatomical structure morphogenesis | 35 | 3.387 | 11.805 | 0.911 | 0.570 | -6.862 | -0.784 |
| GO:0044249 | cellular biosynthetic process | 33 | 3.428 | 12.982 | 0.898 | 0.849 | null | null |
| GO:0048468 | cell development | 32 | 3.302 | 9.703 | 0.888 | 0.800 | null | null |
| GO:0044085 | cellular component biogenesis | 32 | 3.260 | 8.806 | 0.952 | 0.593 | -4.717 | 5.705 |
| GO:0007154 | cell communication | 31 | 3.667 | 22.481 | 0.986 | 0.045 | -4.809 | -6.852 |
| GO:0010033 | response to organic substance | 31 | 2.981 | 4.631 | 0.921 | 0.000 | -0.442 | 6.470 |
| GO:0023051 | regulation of signaling | 31 | 3.212 | 7.896 | 0.941 | 0.194 | -0.372 | 1.929 |
| GO:0023052 | signaling | 31 | 3.660 | 22.127 | 0.932 | 0.284 | 0.959 | 1.693 |
| GO:0034641 | cellular nitrogen compound metabolic process | 31 | 3.402 | 12.207 | 0.908 | 0.556 | 1.728 | -6.266 |
| GO:0009893 | positive regulation of metabolic process | 31 | 3.093 | 5.997 | 0.860 | 0.709 | null | null |
| GO:0051246 | regulation of protein metabolic process | 31 | 2.772 | 2.858 | 0.909 | 0.096 | 2.774 | 0.669 |
| GO:0051641 | cellular localization | 31 | 3.226 | 8.152 | 0.933 | 0.650 | 4.717 | 4.112 |
| GO:0022607 | cellular component assembly | 31 | 3.209 | 7.828 | 0.946 | 0.741 | null | null |
| GO:0009892 | negative regulation of metabolic process | 30 | 3.012 | 4.980 | 0.871 | 0.381 | 3.561 | 0.197 |
| GO:0051128 | regulation of cellular component organization | 30 | 2.989 | 4.713 | 0.940 | 0.104 | -0.496 | 2.360 |
| GO:2000026 | regulation of multicellular organismal development | 30 | 2.712 | 2.490 | 0.861 | 0.094 | 5.629 | -3.387 |
| GO:0009605 | response to external stimulus | 29 | 2.974 | 4.553 | 0.955 | 0.278 | 0.337 | 6.464 |
| GO:0070887 | cellular response to chemical stimulus | 29 | 3.022 | 5.086 | 0.909 | 0.789 | null | null |
| GO:0010604 | positive regulation of macromolecule metabolic process | 29 | 3.071 | 5.701 | 0.850 | 0.879 | null | null |
| GO:0000003 | reproduction | 28 | 2.563 | 1.768 | 1.000 | 0.000 | -5.898 | -6.083 |
| GO:0022414 | reproductive process | 28 | 2.560 | 1.754 | 0.954 | 0.000 | -6.912 | -4.783 |
| GO:0036211 | protein modification process | 28 | 3.009 | 4.941 | 0.906 | 0.000 | 0.905 | -6.537 |
| GO:0043412 | macromolecule modification | 28 | 3.073 | 5.730 | 0.926 | 0.438 | 1.240 | -6.628 |
| GO:0044281 | small molecule metabolic process | 28 | 3.090 | 5.953 | 0.948 | 0.323 | 2.430 | -5.315 |
| GO:0048583 | regulation of response to stimulus | 28 | 3.280 | 9.228 | 0.940 | 0.204 | 0.656 | 2.331 |
| GO:0010468 | regulation of gene expression | 28 | 3.562 | 17.652 | 0.879 | 0.741 | null | null |
| GO:1901360 | organic cyclic compound metabolic process | 28 | 3.375 | 11.471 | 0.928 | 0.453 | 1.683 | -5.993 |
| GO:0045595 | regulation of cell differentiation | 28 | 2.739 | 2.650 | 0.887 | 0.789 | null | null |
| GO:0009888 | tissue development | 27 | 3.209 | 7.828 | 0.916 | 0.514 | -6.920 | -0.860 |
| GO:0010646 | regulation of cell communication | 27 | 3.210 | 7.852 | 0.936 | 0.194 | 0.351 | 1.898 |
| GO:0046483 | heterocycle metabolic process | 27 | 3.341 | 10.608 | 0.920 | 0.376 | 2.308 | -6.134 |
| GO:0007165 | signal transduction | 26 | 3.633 | 20.820 | 0.857 | 0.580 | 0.307 | 5.188 |
| GO:0007399 | nervous system development | 26 | 3.259 | 8.792 | 0.880 | 0.531 | -6.692 | 0.269 |
| GO:0009056 | catabolic process | 26 | 3.149 | 6.825 | 0.947 | 0.334 | 2.492 | -5.460 |
| GO:0033036 | macromolecule localization | 26 | 3.170 | 7.159 | 0.944 | 0.631 | 4.833 | 4.073 |
| GO:0051173 | positive regulation of nitrogen compound metabolic process | 26 | 3.034 | 5.236 | 0.851 | 0.910 | null | null |
| GO:0006725 | cellular aromatic compound metabolic process | 25 | 3.347 | 10.768 | 0.920 | 0.422 | 2.288 | -6.190 |
| GO:0071310 | cellular response to organic substance | 25 | 2.851 | 3.430 | 0.905 | 0.741 | null | null |
| GO:0032504 | multicellular organism reproduction | 25 | 2.322 | 1.012 | 0.908 | 0.918 | null | null |
| GO:0048609 | multicellular organismal reproductive process | 25 | 2.307 | 0.978 | 0.905 | 0.914 | null | null |
| GO:0031324 | negative regulation of cellular metabolic process | 25 | 3.001 | 4.849 | 0.855 | 0.610 | 3.484 | 0.233 |
| GO:0010605 | negative regulation of macromolecule metabolic process | 25 | 2.998 | 4.815 | 0.859 | 0.941 | null | null |
| GO:0044248 | cellular catabolic process | 25 | 2.880 | 3.667 | 0.910 | 0.211 | 2.620 | -5.993 |
| GO:0044703 | multi-organism reproductive process | 25 | 0.477 | 0.010 | 0.969 | 0.597 | -6.878 | -4.797 |
| GO:0031325 | positive regulation of cellular metabolic process | 25 | 3.055 | 5.493 | 0.846 | 0.917 | null | null |
| GO:0071702 | organic substance transport | 25 | 3.169 | 7.145 | 0.939 | 0.676 | 4.754 | 4.277 |
| GO:0051240 | positive regulation of multicellular organismal process | 25 | 2.600 | 1.923 | 0.854 | 0.751 | null | null |
| GO:0006139 | nucleobase-containing compound metabolic process | 24 | 3.320 | 10.105 | 0.890 | 0.755 | null | null |
| GO:0032879 | regulation of localization | 24 | 2.748 | 2.708 | 0.948 | 0.095 | -5.209 | -5.398 |
| GO:1901575 | organic substance catabolic process | 24 | 3.083 | 5.861 | 0.912 | 0.831 | null | null |
| GO:0051094 | positive regulation of developmental process | 24 | 2.512 | 1.569 | 0.858 | 0.736 | null | null |
| GO:0002376 | immune system process | 23 | 3.020 | 5.072 | 1.000 | 0.000 | -6.261 | -5.762 |
| GO:0051172 | negative regulation of nitrogen compound metabolic process | 23 | 2.910 | 3.929 | 0.862 | 0.910 | null | null |
| GO:0009889 | regulation of biosynthetic process | 23 | 3.573 | 18.097 | 0.887 | 0.747 | null | null |
| GO:0071705 | nitrogen compound transport | 23 | 3.033 | 5.227 | 0.942 | 0.632 | 4.701 | 4.207 |
| GO:0003008 | system process | 22 | 3.026 | 5.135 | 0.932 | 0.268 | -6.739 | 2.441 |
| GO:0006793 | phosphorus metabolic process | 22 | 3.281 | 9.242 | 0.921 | 0.408 | 2.385 | -6.168 |
| GO:0009719 | response to endogenous stimulus | 22 | 2.754 | 2.742 | 0.958 | 0.257 | 0.364 | 6.649 |
| GO:0003006 | developmental process involved in reproduction | 22 | 2.316 | 0.998 | 0.900 | 0.916 | null | null |
| GO:0019953 | sexual reproduction | 22 | 2.502 | 1.536 | 0.954 | 0.964 | null | null |
| GO:0008104 | protein localization | 22 | 3.050 | 5.430 | 0.923 | 0.895 | null | null |
| GO:0031326 | regulation of cellular biosynthetic process | 22 | 3.570 | 17.981 | 0.880 | 0.812 | null | null |
| GO:0065009 | regulation of molecular function | 22 | 1.771 | 0.281 | 0.962 | 0.065 | -1.173 | -0.404 |
| GO:1901566 | organonitrogen compound biosynthetic process | 22 | 3.044 | 5.353 | 0.905 | 0.696 | 1.163 | -6.252 |
| GO:0016192 | vesicle-mediated transport | 21 | 2.987 | 4.699 | 0.933 | 0.000 | 4.900 | 4.211 |
| GO:0006796 | phosphate-containing compound metabolic process | 21 | 3.277 | 9.155 | 0.902 | 0.754 | null | null |
| GO:0042592 | homeostatic process | 21 | 2.869 | 3.580 | 1.000 | 0.000 | -3.200 | -7.480 |
| GO:0022008 | neurogenesis | 21 | 3.072 | 5.711 | 0.858 | 0.730 | null | null |
| GO:0009966 | regulation of signal transduction | 21 | 3.147 | 6.796 | 0.899 | 0.774 | null | null |
| GO:0009628 | response to abiotic stimulus | 20 | 2.591 | 1.884 | 0.959 | 0.243 | 0.304 | 6.935 |
| GO:0044271 | cellular nitrogen compound biosynthetic process | 20 | 2.998 | 4.815 | 0.896 | 0.359 | 1.568 | -6.169 |
| GO:0070727 | cellular macromolecule localization | 20 | 3.051 | 5.445 | 0.925 | 0.779 | null | null |
| GO:0048699 | generation of neurons | 20 | 3.022 | 5.091 | 0.857 | 0.872 | null | null |
| GO:0048878 | chemical homeostasis | 20 | 2.664 | 2.228 | 0.975 | 0.000 | -3.777 | -4.775 |
| GO:0050790 | regulation of catalytic activity | 20 | 1.663 | 0.218 | 0.923 | 0.063 | -3.488 | -6.687 |
| GO:0051241 | negative regulation of multicellular organismal process | 20 | 2.415 | 1.255 | 0.866 | 0.711 | null | null |
| GO:0006811 | monoatomic ion transport | 19 | 3.012 | 4.975 | 0.942 | 0.521 | 4.782 | 4.123 |
| GO:0009887 | animal organ morphogenesis | 19 | 2.932 | 4.142 | 0.898 | 0.000 | -6.490 | -0.858 |
| GO:0007276 | gamete generation | 19 | 2.303 | 0.969 | 0.904 | 0.982 | null | null |
| GO:0051049 | regulation of transport | 19 | 2.640 | 2.112 | 0.908 | 0.092 | 0.327 | -0.687 |
| GO:0010556 | regulation of macromolecule biosynthetic process | 19 | 3.566 | 17.845 | 0.879 | 0.814 | null | null |
| GO:1901700 | response to oxygen-containing compound | 19 | 2.662 | 2.219 | 0.928 | 0.694 | -0.528 | 6.492 |
| GO:0007610 | behavior | 18 | 2.401 | 1.216 | 0.942 | 0.000 | -7.005 | 2.612 |
| GO:0010243 | response to organonitrogen compound | 18 | 2.438 | 1.322 | 0.921 | 0.761 | null | null |
| GO:0040011 | locomotion | 18 | 2.425 | 1.284 | 1.000 | 0.000 | -3.858 | -6.887 |
| GO:0051649 | establishment of localization in cell | 18 | 3.048 | 5.406 | 0.924 | 0.790 | null | null |
| GO:0051247 | positive regulation of protein metabolic process | 18 | 2.408 | 1.235 | 0.857 | 0.349 | 4.015 | -0.485 |
| GO:0019219 | regulation of nucleobase-containing compound metabolic process | 18 | 3.511 | 15.695 | 0.881 | 0.740 | null | null |
| GO:1901698 | response to nitrogen compound | 18 | 2.504 | 1.540 | 0.930 | 0.659 | -0.595 | 6.500 |
| GO:0045597 | positive regulation of cell differentiation | 18 | 2.342 | 1.061 | 0.848 | 0.701 | null | null |
| GO:0009894 | regulation of catabolic process | 17 | 2.547 | 1.700 | 0.917 | 0.327 | 3.117 | 0.960 |
| GO:0018130 | heterocycle biosynthetic process | 17 | 2.760 | 2.785 | 0.906 | 0.551 | 2.308 | -5.999 |
| GO:0019438 | aromatic compound biosynthetic process | 17 | 2.759 | 2.776 | 0.906 | 0.537 | 2.227 | -6.035 |
| GO:0022412 | cellular process involved in reproduction in multicellular organism | 17 | 2.193 | 0.751 | 0.900 | 0.955 | null | null |
| GO:0010629 | negative regulation of gene expression | 17 | 2.477 | 1.448 | 0.872 | 0.785 | null | null |
| GO:0035556 | intracellular signal transduction | 17 | 3.120 | 6.375 | 0.878 | 0.382 | 0.100 | 5.628 |
| GO:0048646 | anatomical structure formation involved in morphogenesis | 17 | 2.928 | 4.098 | 0.907 | 0.591 | -6.359 | -0.884 |
| GO:0055085 | transmembrane transport | 17 | 3.168 | 7.135 | 0.930 | 0.676 | 4.810 | 4.225 |
| GO:0051252 | regulation of RNA metabolic process | 17 | 3.491 | 15.007 | 0.880 | 0.707 | null | null |
| GO:0060429 | epithelium development | 17 | 3.006 | 4.902 | 0.909 | 0.343 | -6.901 | -1.170 |
| GO:0061061 | muscle structure development | 17 | 2.606 | 1.952 | 0.929 | 0.299 | -6.558 | -1.290 |
| GO:1901362 | organic cyclic compound biosynthetic process | 17 | 2.823 | 3.216 | 0.912 | 0.531 | 1.473 | -5.935 |
| GO:0009059 | macromolecule biosynthetic process | 17 | 3.293 | 9.514 | 0.891 | 0.788 | null | null |
| GO:0060284 | regulation of cell development | 17 | 2.498 | 1.521 | 0.886 | 0.810 | null | null |
| GO:0006629 | lipid metabolic process | 16 | 2.972 | 4.539 | 0.934 | 0.315 | 1.605 | -6.696 |
| GO:0007010 | cytoskeleton organization | 16 | 3.037 | 5.265 | 0.946 | 0.592 | -4.513 | 5.792 |
| GO:0009410 | response to xenobiotic stimulus | 16 | 1.978 | 0.455 | 0.938 | 0.564 | -0.849 | 6.606 |
| GO:0009790 | embryo development | 16 | 3.128 | 6.506 | 0.889 | 0.492 | -6.613 | 0.209 |
| GO:0009725 | response to hormone | 16 | 2.486 | 1.477 | 0.919 | 0.772 | null | null |
| GO:0009890 | negative regulation of biosynthetic process | 16 | 2.942 | 4.234 | 0.861 | 0.921 | null | null |
| GO:0031327 | negative regulation of cellular biosynthetic process | 16 | 2.939 | 4.205 | 0.856 | 0.920 | null | null |
| GO:0032989 | cellular anatomical entity morphogenesis | 16 | 2.810 | 3.124 | 0.910 | 0.567 | -6.275 | -0.933 |
| GO:0034220 | monoatomic ion transmembrane transport | 16 | 2.972 | 4.539 | 0.926 | 0.513 | 4.872 | 4.155 |
| GO:0034654 | nucleobase-containing compound biosynthetic process | 16 | 2.708 | 2.470 | 0.890 | 0.548 | 1.489 | -6.111 |
| GO:0019220 | regulation of phosphate metabolic process | 16 | 2.238 | 0.833 | 0.901 | 0.974 | null | null |
| GO:0043067 | regulation of programmed cell death | 16 | 2.885 | 3.711 | 0.942 | 0.115 | -1.087 | 2.404 |
| GO:0046907 | intracellular transport | 16 | 2.951 | 4.326 | 0.923 | 0.509 | 4.623 | 4.272 |
| GO:0048585 | negative regulation of response to stimulus | 16 | 2.810 | 3.124 | 0.880 | 0.568 | 6.160 | 0.165 |
| GO:0050801 | monoatomic ion homeostasis | 16 | 2.480 | 1.458 | 0.974 | 0.799 | null | null |
| GO:0051174 | regulation of phosphorus metabolic process | 16 | 2.238 | 0.833 | 0.919 | 0.299 | 2.971 | 0.937 |
| GO:0031399 | regulation of protein modification process | 16 | 2.307 | 0.978 | 0.899 | 0.766 | null | null |
| GO:2001141 | regulation of RNA biosynthetic process | 16 | 3.457 | 13.873 | 0.880 | 0.816 | null | null |
| GO:0006508 | proteolysis | 15 | 3.058 | 5.527 | 0.910 | 0.625 | 1.008 | -6.505 |
| GO:0019637 | organophosphate metabolic process | 15 | 2.863 | 3.531 | 0.909 | 0.284 | 1.950 | -6.482 |
| GO:0019752 | carboxylic acid metabolic process | 15 | 2.740 | 2.659 | 0.895 | 0.272 | 2.090 | -6.499 |
| GO:0006082 | organic acid metabolic process | 15 | 2.778 | 2.902 | 0.899 | 0.740 | null | null |
| GO:0043436 | oxoacid metabolic process | 15 | 2.746 | 2.693 | 0.895 | 0.949 | null | null |
| GO:0007292 | female gamete generation | 15 | 1.903 | 0.383 | 0.912 | 0.825 | null | null |
| GO:0031329 | regulation of cellular catabolic process | 15 | 2.386 | 1.172 | 0.903 | 0.312 | 3.055 | 0.800 |
| GO:0032940 | secretion by cell | 15 | 2.326 | 1.022 | 0.937 | 0.413 | 5.013 | 3.959 |
| GO:0033554 | cellular response to stress | 15 | 2.873 | 3.614 | 0.933 | 0.268 | 0.593 | 6.564 |
| GO:0042325 | regulation of phosphorylation | 15 | 2.164 | 0.702 | 0.903 | 0.293 | 3.257 | 1.095 |
| GO:0043933 | protein-containing complex organization | 15 | 3.066 | 5.634 | 0.953 | 0.533 | -4.788 | 5.666 |
| GO:0044255 | cellular lipid metabolic process | 15 | 2.860 | 3.507 | 0.911 | 0.303 | 2.148 | -6.422 |
| GO:0046903 | secretion | 15 | 2.362 | 1.109 | 0.951 | 0.417 | 4.639 | 4.416 |
| GO:0051050 | positive regulation of transport | 15 | 2.017 | 0.499 | 0.881 | 0.767 | null | null |
| GO:0051130 | positive regulation of cellular component organization | 15 | 2.521 | 1.603 | 0.863 | 0.412 | 3.149 | -1.962 |
| GO:0033043 | regulation of organelle organization | 15 | 2.759 | 2.776 | 0.901 | 0.754 | null | null |
| GO:0006355 | regulation of DNA-templated transcription | 15 | 3.457 | 13.859 | 0.880 | 0.806 | null | null |
| GO:0061024 | membrane organization | 15 | 2.677 | 2.296 | 0.958 | 0.020 | -4.937 | 5.622 |
| GO:1901135 | carbohydrate derivative metabolic process | 15 | 2.897 | 3.817 | 0.938 | 0.286 | 1.413 | -5.590 |
| GO:1902531 | regulation of intracellular signal transduction | 15 | 2.852 | 3.444 | 0.905 | 0.633 | 6.368 | 0.633 |
| GO:0071495 | cellular response to endogenous stimulus | 14 | 2.710 | 2.480 | 0.944 | 0.884 | null | null |
| GO:0010628 | positive regulation of gene expression | 14 | 2.340 | 1.056 | 0.869 | 0.604 | 4.031 | -0.545 |
| GO:0010648 | negative regulation of cell communication | 14 | 2.727 | 2.577 | 0.883 | 0.667 | 5.779 | 0.484 |
| GO:0007281 | germ cell development | 14 | 2.090 | 0.591 | 0.865 | 0.932 | null | null |
| GO:0023057 | negative regulation of signaling | 14 | 2.727 | 2.577 | 0.887 | 0.666 | 5.836 | 0.418 |
| GO:0006812 | monoatomic cation transport | 14 | 2.916 | 3.987 | 0.938 | 0.903 | null | null |
| GO:0042127 | regulation of cell population proliferation | 14 | 2.577 | 1.826 | 0.946 | 0.090 | -4.957 | -5.320 |
| GO:1901565 | organonitrogen compound catabolic process | 14 | 2.934 | 4.156 | 0.901 | 0.771 | null | null |
| GO:0048666 | neuron development | 14 | 2.919 | 4.016 | 0.857 | 0.333 | -6.919 | 0.236 |
| GO:0030182 | neuron differentiation | 14 | 3.003 | 4.878 | 0.857 | 0.906 | null | null |
| GO:0055080 | monoatomic cation homeostasis | 14 | 2.450 | 1.361 | 0.972 | 0.873 | null | null |
| GO:0098771 | inorganic ion homeostasis | 14 | 2.394 | 1.196 | 0.974 | 0.860 | null | null |
| GO:0044093 | positive regulation of molecular function | 14 | 1.477 | 0.140 | 0.926 | 0.923 | null | null |
| GO:0051093 | negative regulation of developmental process | 14 | 2.286 | 0.930 | 0.869 | 0.690 | 5.898 | -2.515 |
| GO:0065003 | protein-containing complex assembly | 14 | 2.812 | 3.139 | 0.949 | 0.341 | -4.594 | 5.462 |
| GO:0009968 | negative regulation of signal transduction | 14 | 2.713 | 2.500 | 0.869 | 0.710 | null | null |
| GO:0002682 | regulation of immune system process | 13 | 2.734 | 2.621 | 0.949 | 0.095 | -5.655 | -5.086 |
| GO:0015833 | peptide transport | 13 | 1.531 | 0.160 | 0.954 | 0.460 | 4.710 | 4.755 |
| GO:0048477 | oogenesis | 13 | 1.839 | 0.329 | 0.871 | 0.812 | null | null |
| GO:0030029 | actin filament-based process | 13 | 2.761 | 2.790 | 0.990 | 0.020 | -5.250 | -6.543 |
| GO:0010558 | negative regulation of macromolecule biosynthetic process | 13 | 2.937 | 4.185 | 0.856 | 0.955 | null | null |
| GO:0098655 | monoatomic cation transmembrane transport | 13 | 2.881 | 3.681 | 0.927 | 0.892 | null | null |
| GO:0001932 | regulation of protein phosphorylation | 13 | 2.130 | 0.649 | 0.887 | 0.950 | null | null |
| GO:0042886 | amide transport | 13 | 1.833 | 0.325 | 0.953 | 0.359 | 4.689 | 4.705 |
| GO:0042981 | regulation of apoptotic process | 13 | 2.878 | 3.652 | 0.935 | 0.114 | -0.186 | 0.209 |
| GO:0044087 | regulation of cellular component biogenesis | 13 | 2.544 | 1.691 | 0.947 | 0.090 | -2.168 | -4.227 |
| GO:0045184 | establishment of protein localization | 13 | 2.909 | 3.924 | 0.924 | 0.833 | null | null |
| GO:0019725 | cellular homeostasis | 13 | 2.515 | 1.579 | 0.976 | 0.807 | null | null |
| GO:0055082 | intracellular chemical homeostasis | 13 | 2.423 | 1.279 | 0.973 | 0.866 | null | null |
| GO:0043085 | positive regulation of catalytic activity | 13 | 1.279 | 0.087 | 0.926 | 0.889 | null | null |
| GO:0051248 | negative regulation of protein metabolic process | 13 | 2.114 | 0.625 | 0.870 | 0.729 | null | null |
| GO:0022603 | regulation of anatomical structure morphogenesis | 13 | 2.577 | 1.826 | 0.895 | 0.751 | null | null |
| GO:0051960 | regulation of nervous system development | 13 | 2.352 | 1.085 | 0.867 | 0.703 | null | null |
| GO:0003012 | muscle system process | 12 | 2.117 | 0.630 | 0.935 | 0.208 | -6.909 | 2.965 |
| GO:0007005 | mitochondrion organization | 12 | 2.362 | 1.109 | 0.955 | 0.478 | -4.524 | 5.951 |
| GO:0007267 | cell-cell signaling | 12 | 2.841 | 3.357 | 0.928 | 0.098 | -2.677 | 7.055 |
| GO:0008283 | cell population proliferation | 12 | 2.212 | 0.785 | 0.991 | 0.017 | -3.517 | -7.503 |
| GO:0009791 | post-embryonic development | 12 | 1.041 | 0.048 | 0.931 | 0.197 | -7.400 | 1.461 |
| GO:0030036 | actin cytoskeleton organization | 12 | 2.744 | 2.679 | 0.945 | 0.348 | -4.360 | 5.708 |
| GO:0045934 | negative regulation of nucleobase-containing compound metabolic process | 12 | 2.838 | 3.328 | 0.858 | 0.887 | null | null |
| GO:0031344 | regulation of cell projection organization | 12 | 2.430 | 1.298 | 0.908 | 0.687 | 1.496 | -1.874 |
| GO:0043687 | post-translational protein modification | 12 | 2.820 | 3.192 | 0.910 | 0.836 | null | null |
| GO:0040008 | regulation of growth | 12 | 2.185 | 0.736 | 0.955 | 0.080 | -4.874 | -6.187 |
| GO:0009057 | macromolecule catabolic process | 12 | 2.834 | 3.299 | 0.911 | 0.810 | null | null |
| GO:0015031 | protein transport | 12 | 2.851 | 3.430 | 0.918 | 0.707 | null | null |
| GO:0006873 | intracellular monoatomic ion homeostasis | 12 | 2.384 | 1.167 | 0.972 | 0.967 | null | null |
| GO:0030162 | regulation of proteolysis | 12 | 2.045 | 0.533 | 0.905 | 0.717 | null | null |
| GO:0042692 | muscle cell differentiation | 12 | 2.446 | 1.347 | 0.903 | 0.876 | null | null |
| GO:0006357 | regulation of transcription by RNA polymerase II | 12 | 3.387 | 11.815 | 0.883 | 0.827 | null | null |
| GO:0072521 | purine-containing compound metabolic process | 12 | 2.528 | 1.628 | 0.906 | 0.516 | 1.270 | -6.412 |
| GO:0080134 | regulation of response to stress | 12 | 2.571 | 1.797 | 0.925 | 0.578 | 6.812 | 0.384 |
| GO:0120035 | regulation of plasma membrane bounded cell projection organization | 12 | 2.423 | 1.279 | 0.904 | 0.686 | 1.450 | -1.875 |
| GO:0007166 | cell surface receptor signaling pathway | 11 | 3.185 | 7.407 | 0.876 | 0.464 | 0.145 | 5.568 |
| GO:0007389 | pattern specification process | 11 | 2.813 | 3.144 | 0.898 | 0.381 | -6.410 | 0.295 |
| GO:0007626 | locomotory behavior | 11 | 2.124 | 0.639 | 0.935 | 0.209 | -6.173 | 2.858 |
| GO:1901701 | cellular response to oxygen-containing compound | 11 | 2.548 | 1.705 | 0.911 | 0.760 | null | null |
| GO:0014070 | response to organic cyclic compound | 11 | 2.212 | 0.785 | 0.929 | 0.603 | -0.757 | 6.545 |
| GO:0090304 | nucleic acid metabolic process | 11 | 3.215 | 7.949 | 0.888 | 0.831 | null | null |
| GO:0032787 | monocarboxylic acid metabolic process | 11 | 2.521 | 1.603 | 0.901 | 0.906 | null | null |
| GO:0055086 | nucleobase-containing small molecule metabolic process | 11 | 2.655 | 2.185 | 0.891 | 0.712 | null | null |
| GO:0030030 | cell projection organization | 11 | 3.024 | 5.120 | 0.954 | 0.522 | -4.770 | 5.765 |
| GO:0030855 | epithelial cell differentiation | 11 | 2.465 | 1.410 | 0.900 | 0.645 | -7.170 | -1.212 |
| GO:0051253 | negative regulation of RNA metabolic process | 11 | 2.804 | 3.081 | 0.859 | 0.937 | null | null |
| GO:0042176 | regulation of protein catabolic process | 11 | 2.061 | 0.552 | 0.898 | 0.830 | null | null |
| GO:0035295 | tube development | 11 | 2.903 | 3.870 | 0.896 | 0.394 | -6.469 | 0.229 |
| GO:0044057 | regulation of system process | 11 | 2.230 | 0.819 | 0.903 | 0.675 | 5.136 | -4.202 |
| GO:0045664 | regulation of neuron differentiation | 11 | 1.806 | 0.305 | 0.901 | 0.609 | 6.125 | -3.067 |
| GO:0030003 | intracellular monoatomic cation homeostasis | 11 | 2.384 | 1.167 | 0.972 | 0.953 | null | null |
| GO:0051336 | regulation of hydrolase activity | 11 | 1.342 | 0.102 | 0.927 | 0.900 | null | null |
| GO:0051129 | negative regulation of cellular component organization | 11 | 2.310 | 0.983 | 0.876 | 0.665 | 2.495 | -1.696 |
| GO:0055001 | muscle cell development | 11 | 2.362 | 1.109 | 0.900 | 0.546 | -7.358 | -1.047 |
| GO:0051146 | striated muscle cell differentiation | 11 | 2.340 | 1.056 | 0.904 | 0.939 | null | null |
| GO:0070925 | organelle assembly | 11 | 2.825 | 3.236 | 0.944 | 0.681 | -4.512 | 5.707 |
| GO:0120036 | plasma membrane bounded cell projection organization | 11 | 3.013 | 4.989 | 0.946 | 0.381 | -4.824 | 5.500 |
| GO:0010467 | gene expression | 11 | 3.196 | 7.605 | 0.892 | 0.750 | null | null |
| GO:0010720 | positive regulation of cell development | 11 | 2.100 | 0.606 | 0.852 | 0.847 | null | null |
| GO:0050767 | regulation of neurogenesis | 11 | 2.283 | 0.925 | 0.858 | 0.782 | null | null |
| GO:0000902 | cell morphogenesis | 10 | 2.814 | 3.149 | 0.910 | 0.567 | -6.239 | -0.921 |
| GO:0001501 | skeletal system development | 10 | 2.581 | 1.841 | 0.900 | 0.402 | -6.591 | 0.560 |
| GO:0006805 | xenobiotic metabolic process | 10 | 1.763 | 0.276 | 0.886 | 0.533 | 0.081 | 3.862 |
| GO:0006955 | immune response | 10 | 2.762 | 2.795 | 0.936 | 0.258 | -0.579 | 5.293 |
| GO:0007049 | cell cycle | 10 | 2.856 | 3.468 | 0.989 | 0.021 | -1.956 | -7.850 |
| GO:0009607 | response to biotic stimulus | 10 | 2.708 | 2.466 | 0.958 | 0.253 | 0.339 | 6.785 |
| GO:0071417 | cellular response to organonitrogen compound | 10 | 2.238 | 0.833 | 0.909 | 0.902 | null | null |
| GO:1901699 | cellular response to nitrogen compound | 10 | 2.322 | 1.012 | 0.916 | 0.921 | null | null |
| GO:0016070 | RNA metabolic process | 10 | 3.004 | 4.883 | 0.895 | 0.613 | 1.483 | -6.329 |
| GO:0016310 | phosphorylation | 10 | 3.066 | 5.634 | 0.908 | 0.821 | null | null |
| GO:1902679 | negative regulation of RNA biosynthetic process | 10 | 2.777 | 2.892 | 0.859 | 0.895 | null | null |
| GO:0009896 | positive regulation of catabolic process | 10 | 2.274 | 0.906 | 0.868 | 0.875 | null | null |
| GO:0033993 | response to lipid | 10 | 2.330 | 1.032 | 0.927 | 0.624 | -0.685 | 6.580 |
| GO:0098660 | inorganic ion transmembrane transport | 10 | 2.901 | 3.856 | 0.931 | 0.772 | null | null |
| GO:0098662 | inorganic cation transmembrane transport | 10 | 2.835 | 3.308 | 0.931 | 0.754 | null | null |
| GO:0035239 | tube morphogenesis | 10 | 2.784 | 2.940 | 0.884 | 0.562 | -6.336 | 0.000 |
| GO:0070647 | protein modification by small protein conjugation or removal | 10 | 2.812 | 3.139 | 0.909 | 0.833 | null | null |
| GO:0048584 | positive regulation of response to stimulus | 10 | 2.904 | 3.880 | 0.869 | 0.654 | 6.104 | -0.374 |
| GO:0048870 | cell motility | 10 | 2.886 | 3.720 | 0.989 | 0.021 | -4.539 | -6.284 |
| GO:0009891 | positive regulation of biosynthetic process | 10 | 2.946 | 4.277 | 0.855 | 0.832 | null | null |
| GO:0031328 | positive regulation of cellular biosynthetic process | 10 | 2.945 | 4.263 | 0.848 | 0.863 | null | null |
| GO:0045935 | positive regulation of nucleobase-containing compound metabolic process | 10 | 2.924 | 4.064 | 0.848 | 0.736 | null | null |
| GO:0051674 | localization of cell | 10 | 0.301 | 0.005 | 0.966 | 0.219 | 4.145 | 5.268 |
| GO:0051707 | response to other organism | 10 | 2.703 | 2.441 | 0.943 | 0.253 | 0.904 | 6.233 |
| GO:0043207 | response to external biotic stimulus | 10 | 2.703 | 2.441 | 0.943 | 0.973 | null | null |
| GO:0055002 | striated muscle cell development | 10 | 2.076 | 0.572 | 0.906 | 0.879 | null | null |
| GO:0072359 | circulatory system development | 10 | 3.004 | 4.883 | 0.888 | 0.465 | -6.665 | 0.357 |
| GO:0090407 | organophosphate biosynthetic process | 10 | 2.615 | 1.991 | 0.892 | 0.595 | 1.843 | -6.357 |
| GO:0006163 | purine nucleotide metabolic process | 10 | 2.490 | 1.492 | 0.870 | 0.941 | null | null |
| GO:0006753 | nucleoside phosphate metabolic process | 10 | 2.584 | 1.855 | 0.876 | 0.816 | null | null |
| GO:0009117 | nucleotide metabolic process | 10 | 2.569 | 1.792 | 0.875 | 0.951 | null | null |
| GO:0009150 | purine ribonucleotide metabolic process | 10 | 2.403 | 1.221 | 0.863 | 0.891 | null | null |
| GO:0009259 | ribonucleotide metabolic process | 10 | 2.436 | 1.318 | 0.867 | 0.969 | null | null |
| GO:0097435 | supramolecular fiber organization | 10 | 2.739 | 2.650 | 0.958 | 0.347 | -4.832 | 5.568 |
| GO:0031175 | neuron projection development | 10 | 2.810 | 3.120 | 0.831 | 0.872 | null | null |
| GO:1901137 | carbohydrate derivative biosynthetic process | 10 | 2.711 | 2.485 | 0.908 | 0.511 | 1.194 | -5.841 |
| GO:0019693 | ribose phosphate metabolic process | 10 | 2.453 | 1.371 | 0.894 | 0.787 | null | null |
| GO:0045596 | negative regulation of cell differentiation | 10 | 2.127 | 0.644 | 0.859 | 0.737 | null | null |
| GO:0002064 | epithelial cell development | 9 | 1.996 | 0.475 | 0.905 | 0.561 | -7.317 | -1.382 |
| GO:0002520 | immune system development | 9 | 1.633 | 0.203 | 0.905 | 0.579 | -5.638 | 0.647 |
| GO:0006952 | defense response | 9 | 2.680 | 2.315 | 0.947 | 0.684 | 0.715 | 6.688 |
| GO:0007417 | central nervous system development | 9 | 2.889 | 3.749 | 0.882 | 0.682 | -6.813 | 0.501 |
| GO:0007423 | sensory organ development | 9 | 2.865 | 3.546 | 0.911 | 0.559 | -6.633 | -0.947 |
| GO:0009792 | embryo development ending in birth or egg hatching | 9 | 2.723 | 2.558 | 0.895 | 0.370 | -6.450 | 0.414 |
| GO:0009991 | response to extracellular stimulus | 9 | 2.009 | 0.489 | 0.953 | 0.653 | 1.325 | 6.113 |
| GO:0034097 | response to cytokine | 9 | 2.447 | 1.351 | 0.925 | 0.763 | null | null |
| GO:0043434 | response to peptide hormone | 9 | 2.079 | 0.576 | 0.919 | 0.867 | null | null |
| GO:0071345 | cellular response to cytokine stimulus | 9 | 2.415 | 1.255 | 0.913 | 0.756 | null | null |
| GO:1901652 | response to peptide | 9 | 2.083 | 0.581 | 0.925 | 0.867 | null | null |
| GO:0010647 | positive regulation of cell communication | 9 | 2.775 | 2.882 | 0.872 | 0.678 | 5.738 | -0.471 |
| GO:0014706 | striated muscle tissue development | 9 | 2.045 | 0.533 | 0.926 | 0.569 | -7.068 | -1.798 |
| GO:0006631 | fatty acid metabolic process | 9 | 2.350 | 1.080 | 0.892 | 0.863 | null | null |
| GO:0048232 | male gamete generation | 9 | 2.064 | 0.557 | 0.909 | 0.926 | null | null |
| GO:0023056 | positive regulation of signaling | 9 | 2.775 | 2.882 | 0.878 | 0.676 | 5.832 | -0.531 |
| GO:0045892 | negative regulation of DNA-templated transcription | 9 | 2.776 | 2.887 | 0.859 | 0.952 | null | null |
| GO:0045732 | positive regulation of protein catabolic process | 9 | 1.881 | 0.363 | 0.863 | 0.796 | null | null |
| GO:0031400 | negative regulation of protein modification process | 9 | 1.681 | 0.228 | 0.875 | 0.659 | 3.960 | 0.022 |
| GO:0031667 | response to nutrient levels | 9 | 2.000 | 0.480 | 0.950 | 0.651 | 1.319 | 6.045 |
| GO:0032970 | regulation of actin filament-based process | 9 | 2.365 | 1.119 | 0.949 | 0.084 | -5.848 | -5.493 |
| GO:0030001 | metal ion transport | 9 | 2.816 | 3.168 | 0.939 | 0.916 | null | null |
| GO:0016042 | lipid catabolic process | 9 | 2.338 | 1.051 | 0.915 | 0.747 | null | null |
| GO:0044242 | cellular lipid catabolic process | 9 | 2.196 | 0.756 | 0.905 | 0.717 | null | null |
| GO:0044282 | small molecule catabolic process | 9 | 2.377 | 1.148 | 0.910 | 0.656 | 3.086 | -5.665 |
| GO:0006886 | intracellular protein transport | 9 | 2.626 | 2.044 | 0.920 | 0.857 | null | null |
| GO:0072657 | protein localization to membrane | 9 | 2.413 | 1.250 | 0.934 | 0.711 | null | null |
| GO:0048534 | hematopoietic or lymphoid organ development | 9 | 1.431 | 0.126 | 0.901 | 0.552 | -5.598 | 0.292 |
| GO:0048638 | regulation of developmental growth | 9 | 2.064 | 0.557 | 0.898 | 0.651 | 6.037 | -3.075 |
| GO:0006874 | intracellular calcium ion homeostasis | 9 | 2.064 | 0.557 | 0.973 | 0.878 | null | null |
| GO:0055074 | calcium ion homeostasis | 9 | 2.104 | 0.610 | 0.974 | 0.887 | null | null |
| GO:0010959 | regulation of metal ion transport | 9 | 1.886 | 0.368 | 0.915 | 0.742 | null | null |
| GO:0032880 | regulation of protein localization | 9 | 2.068 | 0.562 | 0.913 | 0.777 | null | null |
| GO:0043269 | regulation of monoatomic ion transport | 9 | 2.086 | 0.586 | 0.916 | 0.781 | null | null |
| GO:0051046 | regulation of secretion | 9 | 2.258 | 0.872 | 0.913 | 0.817 | null | null |
| GO:0051924 | regulation of calcium ion transport | 9 | 1.519 | 0.155 | 0.920 | 0.846 | null | null |
| GO:0051493 | regulation of cytoskeleton organization | 9 | 2.521 | 1.603 | 0.903 | 0.704 | null | null |
| GO:0010557 | positive regulation of macromolecule biosynthetic process | 9 | 2.938 | 4.195 | 0.848 | 0.954 | null | null |
| GO:0051254 | positive regulation of RNA metabolic process | 9 | 2.877 | 3.643 | 0.849 | 0.858 | null | null |
| GO:0007517 | muscle organ development | 9 | 2.307 | 0.978 | 0.913 | 0.846 | null | null |
| GO:0060322 | head development | 9 | 2.760 | 2.785 | 0.926 | 0.315 | -6.982 | -1.064 |
| GO:0048729 | tissue morphogenesis | 9 | 2.766 | 2.824 | 0.901 | 0.713 | null | null |
| GO:0060537 | muscle tissue development | 9 | 2.365 | 1.119 | 0.923 | 0.625 | -6.980 | -1.542 |
| GO:0000278 | mitotic cell cycle | 8 | 2.574 | 1.812 | 0.972 | 0.019 | -3.135 | -5.827 |
| GO:0001654 | eye development | 8 | 2.721 | 2.543 | 0.882 | 0.532 | -6.594 | 0.025 |
| GO:0001775 | cell activation | 8 | 2.212 | 0.785 | 0.937 | 0.213 | -6.596 | 2.738 |
| GO:0002252 | immune effector process | 8 | 1.968 | 0.446 | 0.980 | 0.630 | -4.991 | -4.388 |
| GO:0003002 | regionalization | 8 | 2.801 | 3.057 | 0.893 | 0.380 | -6.716 | 0.481 |
| GO:0003013 | circulatory system process | 8 | 2.394 | 1.196 | 0.931 | 0.635 | -6.852 | 2.834 |
| GO:0006979 | response to oxidative stress | 8 | 1.954 | 0.431 | 0.955 | 0.552 | 1.069 | 6.866 |
| GO:0007268 | chemical synaptic transmission | 8 | 2.602 | 1.933 | 0.927 | 0.320 | -2.730 | 7.209 |
| GO:0098916 | anterograde trans-synaptic signaling | 8 | 2.602 | 1.933 | 0.927 | 0.978 | null | null |
| GO:0099536 | synaptic signaling | 8 | 2.621 | 2.020 | 0.928 | 0.836 | null | null |
| GO:0099537 | trans-synaptic signaling | 8 | 2.606 | 1.952 | 0.927 | 0.970 | null | null |
| GO:0007420 | brain development | 8 | 2.744 | 2.684 | 0.876 | 0.649 | -6.829 | 0.285 |
| GO:0008015 | blood circulation | 8 | 2.384 | 1.167 | 0.930 | 0.592 | -6.888 | 2.837 |
| GO:0032870 | cellular response to hormone stimulus | 8 | 2.401 | 1.216 | 0.907 | 0.868 | null | null |
| GO:0010035 | response to inorganic substance | 8 | 2.097 | 0.601 | 0.936 | 0.583 | -0.731 | 6.694 |
| GO:0016477 | cell migration | 8 | 2.853 | 3.449 | 0.984 | 0.021 | -6.726 | -5.109 |
| GO:0018193 | peptidyl-amino acid modification | 8 | 1.973 | 0.450 | 0.927 | 0.641 | 0.442 | -6.793 |
| GO:0007283 | spermatogenesis | 8 | 2.037 | 0.523 | 0.888 | 0.920 | null | null |
| GO:0031032 | actomyosin structure organization | 8 | 2.225 | 0.809 | 0.951 | 0.825 | null | null |
| GO:0030097 | hemopoiesis | 8 | 2.684 | 2.335 | 0.902 | 0.604 | -7.278 | -0.874 |
| GO:0031331 | positive regulation of cellular catabolic process | 8 | 2.053 | 0.543 | 0.868 | 0.860 | null | null |
| GO:0006887 | exocytosis | 8 | 2.223 | 0.804 | 0.934 | 0.946 | null | null |
| GO:0045055 | regulated exocytosis | 8 | 1.944 | 0.421 | 0.938 | 0.886 | null | null |
| GO:0006820 | monoatomic anion transport | 8 | 2.255 | 0.867 | 0.948 | 0.727 | null | null |
| GO:0072522 | purine-containing compound biosynthetic process | 8 | 2.248 | 0.853 | 0.891 | 0.823 | null | null |
| GO:0016567 | protein ubiquitination | 8 | 2.762 | 2.795 | 0.910 | 0.947 | null | null |
| GO:0032446 | protein modification by small protein conjugation | 8 | 2.792 | 2.994 | 0.910 | 0.957 | null | null |
| GO:0010562 | positive regulation of phosphorus metabolic process | 8 | 2.049 | 0.538 | 0.865 | 0.932 | null | null |
| GO:0010563 | negative regulation of phosphorus metabolic process | 8 | 1.591 | 0.184 | 0.882 | 0.844 | null | null |
| GO:0045936 | negative regulation of phosphate metabolic process | 8 | 1.591 | 0.184 | 0.881 | 0.844 | null | null |
| GO:0045937 | positive regulation of phosphate metabolic process | 8 | 2.049 | 0.538 | 0.864 | 0.932 | null | null |
| GO:0043066 | negative regulation of apoptotic process | 8 | 2.677 | 2.296 | 0.896 | 0.896 | null | null |
| GO:0043069 | negative regulation of programmed cell death | 8 | 2.680 | 2.315 | 0.896 | 0.897 | null | null |
| GO:0043408 | regulation of MAPK cascade | 8 | 2.405 | 1.226 | 0.913 | 0.645 | 6.515 | 0.504 |
| GO:0043603 | amide metabolic process | 8 | 2.805 | 3.086 | 0.937 | 0.334 | 0.919 | -5.758 |
| GO:0044089 | positive regulation of cellular component biogenesis | 8 | 2.130 | 0.649 | 0.893 | 0.413 | 4.542 | -1.872 |
| GO:0008610 | lipid biosynthetic process | 8 | 2.629 | 2.059 | 0.912 | 0.816 | null | null |
| GO:0045321 | leukocyte activation | 8 | 2.079 | 0.576 | 0.917 | 0.648 | -5.580 | 2.535 |
| GO:0045862 | positive regulation of proteolysis | 8 | 1.820 | 0.315 | 0.867 | 0.680 | 4.132 | -0.588 |
| GO:0043549 | regulation of kinase activity | 8 | 0.954 | 0.039 | 0.894 | 0.896 | null | null |
| GO:0045859 | regulation of protein kinase activity | 8 | 0.954 | 0.039 | 0.881 | 0.838 | null | null |
| GO:0051338 | regulation of transferase activity | 8 | 1.279 | 0.087 | 0.927 | 0.889 | null | null |
| GO:0051345 | positive regulation of hydrolase activity | 8 | 0.903 | 0.034 | 0.930 | 0.830 | null | null |
| GO:1903530 | regulation of secretion by cell | 8 | 2.238 | 0.833 | 0.908 | 0.812 | null | null |
| GO:0045893 | positive regulation of DNA-templated transcription | 8 | 2.826 | 3.241 | 0.850 | 0.916 | null | null |
| GO:1902680 | positive regulation of RNA biosynthetic process | 8 | 2.826 | 3.241 | 0.850 | 0.931 | null | null |
| GO:0051640 | organelle localization | 8 | 2.520 | 1.599 | 0.953 | 0.395 | 4.564 | 4.499 |
| GO:0009617 | response to bacterium | 8 | 2.415 | 1.255 | 0.947 | 0.895 | null | null |
| GO:0051726 | regulation of cell cycle | 8 | 2.855 | 3.463 | 0.942 | 0.113 | -0.131 | 2.382 |
| GO:0090257 | regulation of muscle system process | 8 | 1.663 | 0.218 | 0.904 | 0.584 | 5.165 | -4.277 |
| GO:0010975 | regulation of neuron projection development | 8 | 2.283 | 0.925 | 0.907 | 0.948 | null | null |
| GO:0048812 | neuron projection morphogenesis | 8 | 2.715 | 2.509 | 0.825 | 0.844 | null | null |
| GO:0048858 | cell projection morphogenesis | 8 | 2.720 | 2.538 | 0.870 | 0.913 | null | null |
| GO:0120039 | plasma membrane bounded cell projection morphogenesis | 8 | 2.717 | 2.519 | 0.869 | 0.972 | null | null |
| GO:0009967 | positive regulation of signal transduction | 8 | 2.735 | 2.625 | 0.859 | 0.715 | null | null |
| GO:1902532 | negative regulation of intracellular signal transduction | 8 | 2.255 | 0.867 | 0.880 | 0.845 | null | null |
| GO:0090596 | sensory organ morphogenesis | 7 | 2.513 | 1.574 | 0.900 | 0.823 | null | null |
| GO:0002164 | larval development | 7 | 0.602 | 0.015 | 0.928 | 0.177 | -4.865 | 0.370 |
| GO:0050877 | nervous system process | 7 | 2.860 | 3.502 | 0.922 | 0.724 | null | null |
| GO:0006839 | mitochondrial transport | 7 | 2.057 | 0.547 | 0.955 | 0.382 | 5.184 | 4.061 |
| GO:0006936 | muscle contraction | 7 | 2.104 | 0.610 | 0.931 | 0.554 | -6.872 | 3.010 |
| GO:0007507 | heart development | 7 | 2.773 | 2.868 | 0.882 | 0.541 | -6.763 | 0.052 |
| GO:0008340 | determination of adult lifespan | 7 | 0.477 | 0.010 | 0.961 | 0.145 | -3.830 | 2.155 |
| GO:0009123 | nucleoside monophosphate metabolic process | 7 | 1.869 | 0.354 | 0.894 | 0.675 | 1.802 | -6.642 |
| GO:0009161 | ribonucleoside monophosphate metabolic process | 7 | 1.732 | 0.257 | 0.895 | 0.653 | 1.837 | -6.706 |
| GO:0009126 | purine nucleoside monophosphate metabolic process | 7 | 1.591 | 0.184 | 0.898 | 0.917 | null | null |
| GO:0009167 | purine ribonucleoside monophosphate metabolic process | 7 | 1.591 | 0.184 | 0.898 | 0.917 | null | null |
| GO:0009314 | response to radiation | 7 | 2.253 | 0.862 | 0.950 | 0.219 | 0.908 | 5.705 |
| GO:0016202 | regulation of striated muscle tissue development | 7 | 0.301 | 0.005 | 0.927 | 0.424 | 6.058 | -3.474 |
| GO:0016054 | organic acid catabolic process | 7 | 2.258 | 0.872 | 0.889 | 0.931 | null | null |
| GO:0046395 | carboxylic acid catabolic process | 7 | 2.258 | 0.872 | 0.889 | 0.840 | null | null |
| GO:0002009 | morphogenesis of an epithelium | 7 | 2.688 | 2.359 | 0.900 | 0.716 | null | null |
| GO:0010506 | regulation of autophagy | 7 | 1.996 | 0.475 | 0.911 | 0.818 | null | null |
| GO:0031347 | regulation of defense response | 7 | 2.348 | 1.075 | 0.926 | 0.084 | 6.847 | 0.291 |
| GO:0031401 | positive regulation of protein modification process | 7 | 2.134 | 0.654 | 0.860 | 0.824 | null | null |
| GO:0042326 | negative regulation of phosphorylation | 7 | 1.398 | 0.116 | 0.885 | 0.963 | null | null |
| GO:0042327 | positive regulation of phosphorylation | 7 | 2.021 | 0.504 | 0.865 | 0.988 | null | null |
| GO:0042391 | regulation of membrane potential | 7 | 2.427 | 1.289 | 0.940 | 0.079 | -1.380 | -2.267 |
| GO:0030163 | protein catabolic process | 7 | 2.732 | 2.606 | 0.898 | 0.901 | null | null |
| GO:0051603 | proteolysis involved in protein catabolic process | 7 | 2.719 | 2.529 | 0.898 | 0.779 | null | null |
| GO:0044283 | small molecule biosynthetic process | 7 | 2.520 | 1.599 | 0.910 | 0.684 | 2.047 | -5.338 |
| GO:0002274 | myeloid leukocyte activation | 7 | 1.398 | 0.116 | 0.927 | 0.807 | null | null |
| GO:0048511 | rhythmic process | 7 | 2.057 | 0.547 | 1.000 | 0.000 | -2.895 | -6.747 |
| GO:0048634 | regulation of muscle organ development | 7 | 0.845 | 0.029 | 0.925 | 0.488 | 6.047 | -3.372 |
| GO:0043270 | positive regulation of monoatomic ion transport | 7 | 1.255 | 0.082 | 0.890 | 0.804 | null | null |
| GO:0051928 | positive regulation of calcium ion transport | 7 | 0.903 | 0.034 | 0.894 | 0.752 | null | null |
| GO:0010638 | positive regulation of organelle organization | 7 | 2.265 | 0.886 | 0.864 | 0.787 | null | null |
| GO:0060538 | skeletal muscle organ development | 7 | 2.173 | 0.717 | 0.915 | 0.818 | null | null |
| GO:0060348 | bone development | 7 | 1.813 | 0.310 | 0.905 | 0.407 | -6.109 | 0.122 |
| GO:0048705 | skeletal system morphogenesis | 7 | 2.332 | 1.037 | 0.882 | 0.758 | null | null |
| GO:0006937 | regulation of muscle contraction | 7 | 1.591 | 0.184 | 0.900 | 0.767 | null | null |
| GO:0008016 | regulation of heart contraction | 7 | 1.959 | 0.436 | 0.898 | 0.824 | null | null |
| GO:1903522 | regulation of blood circulation | 7 | 2.057 | 0.547 | 0.896 | 0.896 | null | null |
| GO:0009165 | nucleotide biosynthetic process | 7 | 2.328 | 1.027 | 0.871 | 0.873 | null | null |
| GO:1901293 | nucleoside phosphate biosynthetic process | 7 | 2.330 | 1.032 | 0.873 | 0.874 | null | null |
| GO:0048667 | cell morphogenesis involved in neuron differentiation | 7 | 2.682 | 2.325 | 0.855 | 0.900 | null | null |
| GO:1901861 | regulation of muscle tissue development | 7 | 1.041 | 0.048 | 0.922 | 0.510 | 6.035 | -3.299 |
| GO:1902903 | regulation of supramolecular fiber organization | 7 | 2.310 | 0.983 | 0.910 | 0.665 | 1.473 | -1.971 |
| GO:0010721 | negative regulation of cell development | 7 | 1.919 | 0.397 | 0.861 | 0.877 | null | null |
| GO:0051962 | positive regulation of nervous system development | 7 | 2.061 | 0.552 | 0.836 | 0.906 | null | null |
| GO:0022402 | cell cycle process | 6 | 2.718 | 2.524 | 0.971 | 0.844 | null | null |
| GO:0001101 | response to acid chemical | 6 | 1.415 | 0.121 | 0.945 | 0.488 | -0.997 | 6.937 |
| GO:0048592 | eye morphogenesis | 6 | 2.373 | 1.138 | 0.879 | 0.878 | null | null |
| GO:0001745 | compound eye morphogenesis | 6 | 0.301 | 0.005 | 0.920 | 0.287 | -5.184 | -0.468 |
| GO:0002065 | columnar/cuboidal epithelial cell differentiation | 6 | 1.643 | 0.208 | 0.915 | 0.729 | null | null |
| GO:0002066 | columnar/cuboidal epithelial cell development | 6 | 1.398 | 0.116 | 0.915 | 0.694 | -7.452 | -1.721 |
| GO:0002119 | nematode larval development | 6 | 0.301 | 0.005 | 0.933 | 0.813 | null | null |
| GO:0002446 | neutrophil mediated immunity | 6 | 0.845 | 0.029 | 0.979 | 0.483 | -5.175 | -4.487 |
| GO:0002444 | myeloid leukocyte mediated immunity | 6 | 0.845 | 0.029 | 0.980 | 0.735 | null | null |
| GO:0043312 | neutrophil degranulation | 6 | 0.477 | 0.046 | 0.855 | 1.029 | null | null |
| GO:0006091 | generation of precursor metabolites and energy | 6 | 2.365 | 1.119 | 0.940 | 0.242 | 3.058 | -6.144 |
| GO:0006468 | protein phosphorylation | 6 | 1.415 | 0.121 | 0.918 | 0.554 | 0.569 | -6.895 |
| GO:0006518 | peptide metabolic process | 6 | 2.655 | 2.185 | 0.924 | 0.440 | 0.895 | -6.614 |
| GO:0006520 | amino acid metabolic process | 6 | 2.394 | 1.196 | 0.944 | 0.259 | 1.491 | -7.066 |
| GO:0006575 | cellular modified amino acid metabolic process | 6 | 2.061 | 0.552 | 0.928 | 0.366 | 0.964 | -6.828 |
| GO:0006790 | sulfur compound metabolic process | 6 | 2.332 | 1.037 | 0.940 | 0.240 | 3.112 | -6.037 |
| GO:0006897 | endocytosis | 6 | 2.545 | 1.695 | 0.934 | 0.671 | 4.594 | 4.018 |
| GO:0007017 | microtubule-based process | 6 | 2.797 | 3.032 | 0.990 | 0.021 | -2.082 | -7.648 |
| GO:0007444 | imaginal disc development | 6 | 2.270 | 0.768 | 0.924 | 0.453 | -6.347 | -1.343 |
| GO:0007623 | circadian rhythm | 6 | 1.863 | 0.349 | 0.998 | 0.000 | -2.252 | -7.840 |
| GO:0030534 | adult behavior | 6 | 1.146 | 0.063 | 0.946 | 0.701 | null | null |
| GO:0003015 | heart process | 6 | 2.117 | 0.630 | 0.934 | 0.919 | null | null |
| GO:0060047 | heart contraction | 6 | 2.104 | 0.610 | 0.934 | 0.916 | null | null |
| GO:0008284 | positive regulation of cell population proliferation | 6 | 2.336 | 1.046 | 0.899 | 0.435 | 4.962 | -1.584 |
| GO:0008344 | adult locomotory behavior | 6 | 0.477 | 0.010 | 0.952 | 0.609 | -5.654 | 3.489 |
| GO:0009141 | nucleoside triphosphate metabolic process | 6 | 2.053 | 0.543 | 0.890 | 0.742 | null | null |
| GO:0009144 | purine nucleoside triphosphate metabolic process | 6 | 1.978 | 0.455 | 0.889 | 0.968 | null | null |
| GO:0009199 | ribonucleoside triphosphate metabolic process | 6 | 2.004 | 0.484 | 0.889 | 0.713 | null | null |
| GO:0009205 | purine ribonucleoside triphosphate metabolic process | 6 | 1.959 | 0.436 | 0.889 | 0.971 | null | null |
| GO:0046034 | ATP metabolic process | 6 | 1.881 | 0.363 | 0.876 | 0.948 | null | null |
| GO:0009611 | response to wounding | 6 | 2.220 | 0.799 | 0.952 | 0.594 | 0.896 | 6.839 |
| GO:0019221 | cytokine-mediated signaling pathway | 6 | 2.389 | 1.182 | 0.863 | 0.963 | null | null |
| GO:0010038 | response to metal ion | 6 | 2.000 | 0.480 | 0.935 | 0.567 | -0.810 | 6.736 |
| GO:0010256 | endomembrane system organization | 6 | 2.453 | 1.371 | 0.961 | 0.306 | -4.843 | 5.976 |
| GO:0010817 | regulation of hormone levels | 6 | 2.173 | 0.717 | 0.943 | 0.532 | -1.489 | -2.465 |
| GO:0010883 | regulation of lipid storage | 6 | 1.146 | 0.063 | 0.932 | 0.625 | -1.325 | -3.790 |
| GO:0010927 | cellular component assembly involved in morphogenesis | 6 | 2.107 | 0.615 | 0.886 | 0.549 | -5.436 | 1.797 |
| GO:0015711 | organic anion transport | 6 | 2.583 | 1.850 | 0.944 | 0.470 | 4.861 | 4.398 |
| GO:0048641 | regulation of skeletal muscle tissue development | 6 | 1.000 | 0.044 | 0.918 | 0.863 | null | null |
| GO:0006635 | fatty acid beta-oxidation | 6 | 1.672 | 0.223 | 0.891 | 0.946 | null | null |
| GO:0009062 | fatty acid catabolic process | 6 | 1.845 | 0.334 | 0.887 | 0.900 | null | null |
| GO:0019395 | fatty acid oxidation | 6 | 1.724 | 0.252 | 0.905 | 0.966 | null | null |
| GO:0072329 | monocarboxylic acid catabolic process | 6 | 1.886 | 0.368 | 0.897 | 0.909 | null | null |
| GO:0021700 | developmental maturation | 6 | 2.057 | 0.547 | 0.940 | 0.243 | -7.055 | -2.159 |
| GO:0030707 | follicle cell of egg chamber development | 6 | 1.230 | 0.137 | 0.870 | 0.901 | null | null |
| GO:0048608 | reproductive structure development | 6 | 1.431 | 0.126 | 0.891 | 0.739 | null | null |
| GO:0022604 | regulation of cell morphogenesis | 6 | 2.025 | 0.509 | 0.905 | 0.644 | 6.075 | -3.191 |
| GO:0030155 | regulation of cell adhesion | 6 | 2.364 | 1.114 | 0.949 | 0.084 | -6.258 | -5.323 |
| GO:0060562 | epithelial tube morphogenesis | 6 | 2.403 | 1.221 | 0.884 | 0.850 | null | null |
| GO:0000122 | negative regulation of transcription by RNA polymerase II | 6 | 2.584 | 1.855 | 0.865 | 0.896 | null | null |
| GO:0009895 | negative regulation of catabolic process | 6 | 1.924 | 0.402 | 0.883 | 0.804 | null | null |
| GO:0007584 | response to nutrient | 6 | 1.041 | 0.048 | 0.937 | 0.816 | null | null |
| GO:0032886 | regulation of microtubule-based process | 6 | 2.114 | 0.625 | 0.952 | 0.078 | -6.455 | -5.560 |
| GO:0002263 | cell activation involved in immune response | 6 | 1.477 | 0.140 | 0.896 | 0.895 | null | null |
| GO:0002275 | myeloid cell activation involved in immune response | 6 | 1.176 | 0.068 | 0.900 | 0.944 | null | null |
| GO:0002366 | leukocyte activation involved in immune response | 6 | 1.462 | 0.136 | 0.895 | 0.892 | null | null |
| GO:0002443 | leukocyte mediated immunity | 6 | 1.716 | 0.247 | 0.977 | 0.939 | null | null |
| GO:0043299 | leukocyte degranulation | 6 | 1.929 | 0.417 | 0.909 | 0.885 | null | null |
| GO:0098656 | monoatomic anion transmembrane transport | 6 | 2.233 | 0.823 | 0.938 | 0.723 | null | null |
| GO:0034440 | lipid oxidation | 6 | 1.778 | 0.286 | 0.930 | 0.641 | 2.672 | -6.624 |
| GO:0006351 | DNA-templated transcription | 6 | 2.322 | 1.012 | 0.895 | 0.840 | null | null |
| GO:0006366 | transcription by RNA polymerase II | 6 | 2.025 | 0.509 | 0.902 | 0.914 | null | null |
| GO:0032774 | RNA biosynthetic process | 6 | 2.336 | 1.046 | 0.895 | 0.844 | null | null |
| GO:0034976 | response to endoplasmic reticulum stress | 6 | 2.068 | 0.562 | 0.943 | 0.570 | 0.902 | 6.714 |
| GO:0040012 | regulation of locomotion | 6 | 2.509 | 1.560 | 0.951 | 0.088 | -5.379 | -5.743 |
| GO:0042060 | wound healing | 6 | 2.083 | 0.581 | 0.954 | 0.572 | 0.998 | 6.831 |
| GO:0001933 | negative regulation of protein phosphorylation | 6 | 1.362 | 0.107 | 0.872 | 0.957 | null | null |
| GO:0001934 | positive regulation of protein phosphorylation | 6 | 2.009 | 0.489 | 0.851 | 0.986 | null | null |
| GO:0043068 | positive regulation of programmed cell death | 6 | 2.238 | 0.833 | 0.898 | 0.783 | null | null |
| GO:0043062 | extracellular structure organization | 6 | 2.334 | 1.041 | 0.962 | 0.295 | -5.027 | 5.784 |
| GO:0006511 | ubiquitin-dependent protein catabolic process | 6 | 2.624 | 2.034 | 0.901 | 0.938 | null | null |
| GO:0019941 | modification-dependent protein catabolic process | 6 | 2.634 | 2.083 | 0.900 | 0.971 | null | null |
| GO:0043632 | modification-dependent macromolecule catabolic process | 6 | 2.642 | 2.122 | 0.913 | 0.876 | null | null |
| GO:0030258 | lipid modification | 6 | 2.193 | 0.751 | 0.924 | 0.716 | null | null |
| GO:0046486 | glycerolipid metabolic process | 6 | 2.453 | 1.371 | 0.919 | 0.773 | null | null |
| GO:0045165 | cell fate commitment | 6 | 2.255 | 0.867 | 0.917 | 0.436 | -7.584 | -1.021 |
| GO:0002283 | neutrophil activation involved in immune response | 6 | 0.845 | 0.029 | 0.906 | 0.950 | null | null |
| GO:0036230 | granulocyte activation | 6 | 1.114 | 0.058 | 0.930 | 0.901 | null | null |
| GO:0042119 | neutrophil activation | 6 | 1.079 | 0.053 | 0.930 | 0.758 | null | null |
| GO:1903050 | regulation of proteolysis involved in protein catabolic process | 6 | 1.934 | 0.412 | 0.902 | 0.925 | null | null |
| GO:0046677 | response to antibiotic | 6 | 0.699 | 0.019 | 0.952 | 0.411 | -1.502 | 6.831 |
| GO:0033365 | protein localization to organelle | 6 | 2.581 | 1.841 | 0.933 | 0.748 | null | null |
| GO:0048193 | Golgi vesicle transport | 6 | 2.314 | 0.993 | 0.938 | 0.411 | 4.464 | 4.112 |
| GO:0048749 | compound eye development | 6 | 0.477 | 0.010 | 0.926 | 0.554 | -7.850 | 0.697 |
| GO:0050776 | regulation of immune response | 6 | 2.489 | 1.487 | 0.901 | 0.495 | 6.733 | 0.093 |
| GO:0002683 | negative regulation of immune system process | 6 | 2.140 | 0.664 | 0.896 | 0.769 | null | null |
| GO:0002791 | regulation of peptide secretion | 6 | 1.431 | 0.126 | 0.923 | 0.820 | null | null |
| GO:0050708 | regulation of protein secretion | 6 | 1.505 | 0.150 | 0.917 | 0.931 | null | null |
| GO:0051223 | regulation of protein transport | 6 | 1.681 | 0.228 | 0.916 | 0.872 | null | null |
| GO:0060627 | regulation of vesicle-mediated transport | 6 | 2.286 | 0.930 | 0.909 | 0.823 | null | null |
| GO:0070201 | regulation of establishment of protein localization | 6 | 1.771 | 0.281 | 0.917 | 0.888 | null | null |
| GO:0010639 | negative regulation of organelle organization | 6 | 2.083 | 0.581 | 0.876 | 0.751 | null | null |
| GO:0032956 | regulation of actin cytoskeleton organization | 6 | 2.358 | 1.100 | 0.900 | 0.819 | null | null |
| GO:0070507 | regulation of microtubule cytoskeleton organization | 6 | 1.964 | 0.441 | 0.911 | 0.729 | null | null |
| GO:0045944 | positive regulation of transcription by RNA polymerase II | 6 | 2.656 | 2.189 | 0.856 | 0.865 | null | null |
| GO:0051259 | protein complex oligomerization | 6 | 2.083 | 0.581 | 0.957 | 0.631 | -4.603 | 5.336 |
| GO:0051276 | chromosome organization | 6 | 2.418 | 1.264 | 0.954 | 0.486 | -4.409 | 5.870 |
| GO:0042330 | taxis | 6 | 2.403 | 1.221 | 0.948 | 0.726 | null | null |
| GO:0042742 | defense response to bacterium | 6 | 2.064 | 0.557 | 0.942 | 0.814 | null | null |
| GO:0098542 | defense response to other organism | 6 | 2.558 | 1.744 | 0.936 | 0.933 | null | null |
| GO:0007519 | skeletal muscle tissue development | 6 | 2.121 | 0.635 | 0.907 | 0.917 | null | null |
| GO:0061458 | reproductive system development | 6 | 1.431 | 0.126 | 0.922 | 0.216 | -6.055 | 1.000 |
| GO:0071407 | cellular response to organic cyclic compound | 6 | 2.057 | 0.547 | 0.917 | 0.577 | -0.705 | 6.420 |
| GO:0090087 | regulation of peptide transport | 6 | 1.431 | 0.126 | 0.926 | 0.667 | 0.107 | -0.966 |
| GO:0006942 | regulation of striated muscle contraction | 6 | 1.230 | 0.078 | 0.906 | 0.909 | null | null |
| GO:0006164 | purine nucleotide biosynthetic process | 6 | 2.223 | 0.804 | 0.866 | 0.964 | null | null |
| GO:0009152 | purine ribonucleotide biosynthetic process | 6 | 2.155 | 0.688 | 0.861 | 0.947 | null | null |
| GO:0009260 | ribonucleotide biosynthetic process | 6 | 2.207 | 0.775 | 0.863 | 0.974 | null | null |
| GO:0046390 | ribose phosphate biosynthetic process | 6 | 2.220 | 0.799 | 0.887 | 0.916 | null | null |
| GO:0098657 | import into cell | 6 | 2.707 | 2.461 | 0.947 | 0.467 | 4.588 | 4.227 |
| GO:0031346 | positive regulation of cell projection organization | 6 | 2.033 | 0.518 | 0.870 | 0.891 | null | null |
| GO:1902533 | positive regulation of intracellular signal transduction | 6 | 2.533 | 1.647 | 0.864 | 0.889 | null | null |
| GO:1905952 | regulation of lipid localization | 6 | 1.505 | 0.150 | 0.927 | 0.678 | 0.161 | -0.913 |
| GO:1990542 | mitochondrial transmembrane transport | 6 | 1.949 | 0.426 | 0.945 | 0.573 | 4.376 | 4.523 |
| GO:1990778 | protein localization to cell periphery | 6 | 2.100 | 0.606 | 0.940 | 0.558 | 4.495 | 4.631 |
| GO:0050769 | positive regulation of neurogenesis | 6 | 1.978 | 0.455 | 0.828 | 0.888 | null | null |
| GO:1903706 | regulation of hemopoiesis | 6 | 1.919 | 0.397 | 0.860 | 0.807 | null | null |
| GO:0000209 | protein polyubiquitination | 5 | 2.250 | 0.857 | 0.921 | 0.694 | 0.576 | -6.704 |
| GO:0001503 | ossification | 5 | 1.934 | 0.412 | 0.947 | 0.199 | -6.482 | 2.942 |
| GO:0035282 | segmentation | 5 | 2.104 | 0.610 | 0.910 | 0.780 | null | null |
| GO:0005975 | carbohydrate metabolic process | 5 | 2.515 | 1.579 | 0.942 | 0.269 | 1.652 | -6.979 |
| GO:0071466 | cellular response to xenobiotic stimulus | 5 | 1.934 | 0.412 | 0.927 | 0.943 | null | null |
| GO:0007155 | cell adhesion | 5 | 2.893 | 3.783 | 0.989 | 0.021 | -4.033 | -7.270 |
| GO:0007167 | enzyme-linked receptor protein signaling pathway | 5 | 2.634 | 2.083 | 0.892 | 0.323 | 0.039 | 5.820 |
| GO:0007346 | regulation of mitotic cell cycle | 5 | 2.655 | 2.185 | 0.941 | 0.106 | -3.494 | -6.046 |
| GO:0001944 | vasculature development | 5 | 2.711 | 2.485 | 0.892 | 0.793 | null | null |
| GO:0007622 | rhythmic behavior | 5 | 1.146 | 0.063 | 0.946 | 0.701 | null | null |
| GO:0009266 | response to temperature stimulus | 5 | 1.778 | 0.286 | 0.955 | 0.734 | null | null |
| GO:0009416 | response to light stimulus | 5 | 2.167 | 0.707 | 0.950 | 0.803 | null | null |
| GO:0009636 | response to toxic substance | 5 | 2.025 | 0.509 | 0.937 | 0.571 | -0.744 | 6.783 |
| GO:0032868 | response to insulin | 5 | 1.799 | 0.300 | 0.923 | 0.934 | null | null |
| GO:0070848 | response to growth factor | 5 | 2.286 | 0.930 | 0.950 | 0.787 | null | null |
| GO:0071363 | cellular response to growth factor stimulus | 5 | 2.283 | 0.925 | 0.949 | 0.786 | null | null |
| GO:0071375 | cellular response to peptide hormone stimulus | 5 | 1.996 | 0.475 | 0.910 | 0.974 | null | null |
| GO:1901653 | cellular response to peptide | 5 | 1.996 | 0.475 | 0.911 | 0.974 | null | null |
| GO:0010608 | post-transcriptional regulation of gene expression | 5 | 2.556 | 1.739 | 0.910 | 0.346 | 2.847 | 0.741 |
| GO:0015849 | organic acid transport | 5 | 2.476 | 1.444 | 0.945 | 0.596 | 4.924 | 4.388 |
| GO:0016050 | vesicle organization | 5 | 2.307 | 0.978 | 0.955 | 0.471 | -4.435 | 5.956 |
| GO:0019098 | reproductive behavior | 5 | 0.477 | 0.010 | 0.927 | 0.609 | -5.969 | 3.564 |
| GO:0016053 | organic acid biosynthetic process | 5 | 2.267 | 0.891 | 0.897 | 0.861 | null | null |
| GO:0046394 | carboxylic acid biosynthetic process | 5 | 2.262 | 0.882 | 0.896 | 0.842 | null | null |
| GO:0007548 | sex differentiation | 5 | 1.580 | 0.179 | 0.911 | 0.765 | null | null |
| GO:0009994 | oocyte differentiation | 5 | 1.613 | 0.194 | 0.876 | 0.927 | null | null |
| GO:0051321 | meiotic cell cycle | 5 | 2.029 | 0.513 | 0.937 | 0.851 | null | null |
| GO:0000226 | microtubule cytoskeleton organization | 5 | 2.636 | 2.093 | 0.948 | 0.745 | null | null |
| GO:0007015 | actin filament organization | 5 | 2.467 | 1.414 | 0.948 | 0.883 | null | null |
| GO:0030198 | extracellular matrix organization | 5 | 2.334 | 1.041 | 0.961 | 0.295 | -5.115 | 5.723 |
| GO:0030334 | regulation of cell migration | 5 | 2.447 | 1.351 | 0.939 | 0.086 | -6.092 | -5.060 |
| GO:2000145 | regulation of cell motility | 5 | 2.479 | 1.453 | 0.939 | 0.960 | null | null |
| GO:0031330 | negative regulation of cellular catabolic process | 5 | 1.820 | 0.315 | 0.879 | 0.785 | null | null |
| GO:0080135 | regulation of cellular response to stress | 5 | 2.086 | 0.586 | 0.927 | 0.816 | null | null |
| GO:0031647 | regulation of protein stability | 5 | 1.623 | 0.199 | 0.949 | 0.467 | -1.509 | -2.724 |
| GO:0023061 | signal release | 5 | 2.025 | 0.509 | 0.890 | 0.903 | null | null |
| GO:0006816 | calcium ion transport | 5 | 2.326 | 1.022 | 0.946 | 0.743 | null | null |
| GO:0070588 | calcium ion transmembrane transport | 5 | 2.299 | 0.959 | 0.935 | 0.777 | null | null |
| GO:0034599 | cellular response to oxidative stress | 5 | 1.653 | 0.213 | 0.923 | 0.518 | -0.328 | 6.790 |
| GO:0035220 | wing disc development | 5 | 2.270 | 0.077 | 0.937 | 0.352 | -6.349 | -2.162 |
| GO:0001568 | blood vessel development | 5 | 2.636 | 2.093 | 0.894 | 0.914 | null | null |
| GO:0048514 | blood vessel morphogenesis | 5 | 2.573 | 1.807 | 0.883 | 0.829 | null | null |
| GO:0035966 | response to topologically incorrect protein | 5 | 1.763 | 0.276 | 0.928 | 0.533 | -0.453 | 6.851 |
| GO:0035967 | cellular response to topologically incorrect protein | 5 | 1.690 | 0.233 | 0.917 | 0.578 | -0.377 | 6.749 |
| GO:0006986 | response to unfolded protein | 5 | 1.699 | 0.237 | 0.927 | 0.971 | null | null |
| GO:0030968 | endoplasmic reticulum unfolded protein response | 5 | 1.568 | 0.174 | 0.876 | 0.947 | null | null |
| GO:0034620 | cellular response to unfolded protein | 5 | 1.613 | 0.194 | 0.918 | 0.960 | null | null |
| GO:0042063 | gliogenesis | 5 | 2.104 | 0.610 | 0.886 | 0.649 | -7.079 | 0.408 |
| GO:0010498 | proteasomal protein catabolic process | 5 | 2.403 | 1.221 | 0.906 | 0.879 | null | null |
| GO:0043161 | proteasome-mediated ubiquitin-dependent protein catabolic process | 5 | 2.305 | 0.974 | 0.908 | 0.855 | null | null |
| GO:0061136 | regulation of proteasomal protein catabolic process | 5 | 1.914 | 0.392 | 0.895 | 0.964 | null | null |
| GO:1903052 | positive regulation of proteolysis involved in protein catabolic process | 5 | 1.771 | 0.281 | 0.867 | 0.936 | null | null |
| GO:2000058 | regulation of ubiquitin-dependent protein catabolic process | 5 | 1.799 | 0.300 | 0.898 | 0.900 | null | null |
| GO:0045995 | regulation of embryonic development | 5 | 1.362 | 0.107 | 0.890 | 0.548 | 5.671 | -3.544 |
| GO:0090150 | establishment of protein localization to membrane | 5 | 2.137 | 0.659 | 0.936 | 0.870 | null | null |
| GO:0048285 | organelle fission | 5 | 2.233 | 0.823 | 0.956 | 0.461 | -4.320 | 5.923 |
| GO:0048469 | cell maturation | 5 | 1.898 | 0.378 | 0.915 | 0.479 | -7.612 | -1.226 |
| GO:0071695 | anatomical structure maturation | 5 | 1.973 | 0.450 | 0.935 | 0.943 | null | null |
| GO:0045927 | positive regulation of growth | 5 | 1.556 | 0.170 | 0.911 | 0.843 | null | null |
| GO:0048639 | positive regulation of developmental growth | 5 | 1.431 | 0.126 | 0.874 | 0.822 | null | null |
| GO:0048871 | multicellular organismal-level homeostasis | 5 | 2.415 | 1.255 | 0.920 | 0.785 | null | null |
| GO:0002697 | regulation of immune effector process | 5 | 1.792 | 0.295 | 0.935 | 0.707 | null | null |
| GO:0043086 | negative regulation of catalytic activity | 5 | 1.230 | 0.078 | 0.927 | 0.882 | null | null |
| GO:0044092 | negative regulation of molecular function | 5 | 1.301 | 0.092 | 0.928 | 0.893 | null | null |
| GO:0071900 | regulation of protein serine/threonine kinase activity | 5 | 0.602 | 0.015 | 0.888 | 0.941 | null | null |
| GO:0050803 | regulation of synapse structure or activity | 5 | 1.792 | 0.295 | 0.947 | 0.485 | -1.621 | -2.465 |
| GO:0050804 | modulation of chemical synaptic transmission | 5 | 2.258 | 0.872 | 0.929 | 0.081 | 6.470 | 1.253 |
| GO:0050807 | regulation of synapse organization | 5 | 1.748 | 0.266 | 0.908 | 0.579 | 0.036 | -2.148 |
| GO:0002793 | positive regulation of peptide secretion | 5 | 1.176 | 0.068 | 0.892 | 0.954 | null | null |
| GO:0030100 | regulation of endocytosis | 5 | 1.763 | 0.276 | 0.884 | 0.720 | null | null |
| GO:0034762 | regulation of transmembrane transport | 5 | 1.973 | 0.450 | 0.914 | 0.758 | null | null |
| GO:0034765 | regulation of monoatomic ion transmembrane transport | 5 | 1.919 | 0.397 | 0.909 | 0.918 | null | null |
| GO:0050714 | positive regulation of protein secretion | 5 | 1.146 | 0.063 | 0.887 | 0.912 | null | null |
| GO:0051047 | positive regulation of secretion | 5 | 1.602 | 0.189 | 0.884 | 0.850 | null | null |
| GO:0051051 | negative regulation of transport | 5 | 1.778 | 0.286 | 0.887 | 0.723 | null | null |
| GO:0051222 | positive regulation of protein transport | 5 | 1.380 | 0.111 | 0.883 | 0.909 | null | null |
| GO:1903169 | regulation of calcium ion transmembrane transport | 5 | 1.230 | 0.078 | 0.918 | 0.853 | null | null |
| GO:1903532 | positive regulation of secretion by cell | 5 | 1.568 | 0.174 | 0.880 | 0.844 | null | null |
| GO:1904062 | regulation of monoatomic cation transmembrane transport | 5 | 1.699 | 0.237 | 0.913 | 0.930 | null | null |
| GO:1904951 | positive regulation of establishment of protein localization | 5 | 1.531 | 0.160 | 0.886 | 0.936 | null | null |
| GO:0051494 | negative regulation of cytoskeleton organization | 5 | 1.898 | 0.378 | 0.878 | 0.853 | null | null |
| GO:0110053 | regulation of actin filament organization | 5 | 2.210 | 0.780 | 0.902 | 0.949 | null | null |
| GO:0051260 | protein homooligomerization | 5 | 2.000 | 0.480 | 0.957 | 0.639 | -4.537 | 5.307 |
| GO:0051301 | cell division | 5 | 2.483 | 1.468 | 0.990 | 0.019 | -5.239 | -6.112 |
| GO:0006935 | chemotaxis | 5 | 2.401 | 1.216 | 0.921 | 0.975 | null | null |
| GO:0014902 | myotube differentiation | 5 | 1.940 | 0.417 | 0.912 | 0.851 | null | null |
| GO:0030239 | myofibril assembly | 5 | 2.041 | 0.528 | 0.857 | 0.872 | null | null |
| GO:0031960 | response to corticosteroid | 5 | 1.204 | 0.073 | 0.933 | 0.811 | null | null |
| GO:0048545 | response to steroid hormone | 5 | 1.771 | 0.281 | 0.926 | 0.868 | null | null |
| GO:0051384 | response to glucocorticoid | 5 | 1.204 | 0.073 | 0.933 | 0.774 | null | null |
| GO:0071496 | cellular response to external stimulus | 5 | 1.820 | 0.315 | 0.955 | 0.623 | 1.442 | 5.987 |
| GO:0090066 | regulation of anatomical structure size | 5 | 2.480 | 1.458 | 0.939 | 0.576 | -1.342 | -2.172 |
| GO:0006644 | phospholipid metabolic process | 5 | 2.476 | 1.444 | 0.894 | 0.791 | null | null |
| GO:0099177 | regulation of trans-synaptic signaling | 5 | 2.258 | 0.872 | 0.930 | 0.494 | 6.444 | 1.234 |
| GO:0031345 | negative regulation of cell projection organization | 5 | 1.771 | 0.281 | 0.882 | 0.838 | null | null |
| GO:0060491 | regulation of cell projection assembly | 5 | 1.898 | 0.378 | 0.910 | 0.863 | null | null |
| GO:0120032 | regulation of plasma membrane bounded cell projection assembly | 5 | 1.886 | 0.368 | 0.909 | 0.861 | null | null |
| GO:0007409 | axonogenesis | 5 | 2.651 | 2.165 | 0.827 | 0.955 | null | null |
| GO:0007411 | axon guidance | 5 | 2.502 | 1.536 | 0.833 | 0.913 | null | null |
| GO:0061564 | axon development | 5 | 2.691 | 2.374 | 0.835 | 0.905 | null | null |
| GO:0097485 | neuron projection guidance | 5 | 2.502 | 1.536 | 0.833 | 0.913 | null | null |
| GO:1905039 | carboxylic acid transmembrane transport | 5 | 2.378 | 1.153 | 0.932 | 0.649 | 4.838 | 4.453 |
| GO:0046942 | carboxylic acid transport | 5 | 2.476 | 1.444 | 0.942 | 0.960 | null | null |
| GO:1903825 | organic acid transmembrane transport | 5 | 2.378 | 1.153 | 0.932 | 0.936 | null | null |
| GO:1905954 | positive regulation of lipid localization | 5 | 0.954 | 0.039 | 0.904 | 0.600 | 4.005 | -2.009 |
| GO:0010884 | positive regulation of lipid storage | 5 | 0.778 | 0.024 | 0.907 | 0.802 | null | null |
| GO:0045666 | positive regulation of neuron differentiation | 5 | 1.380 | 0.111 | 0.871 | 0.721 | null | null |
| GO:0050768 | negative regulation of neurogenesis | 5 | 1.763 | 0.276 | 0.839 | 0.878 | null | null |
| GO:0051961 | negative regulation of nervous system development | 5 | 1.785 | 0.291 | 0.848 | 0.851 | null | null |
| GO:2000027 | regulation of animal organ morphogenesis | 5 | 1.415 | 0.121 | 0.915 | 0.655 | 6.084 | -3.242 |
| GO:2000241 | regulation of reproductive process | 5 | 2.121 | 0.635 | 0.956 | 0.078 | -1.638 | 1.531 |
| GO:0007059 | chromosome segregation | 4 | 2.255 | 0.867 | 0.972 | 0.799 | null | null |
| GO:1903047 | mitotic cell cycle process | 4 | 2.480 | 1.458 | 0.970 | 0.788 | null | null |
| GO:0001817 | regulation of cytokine production | 4 | 2.104 | 0.610 | 0.871 | 0.653 | 4.486 | -1.364 |
| GO:0001819 | positive regulation of cytokine production | 4 | 1.748 | 0.266 | 0.842 | 0.596 | 4.586 | -1.583 |
| GO:0002165 | instar larval or pupal development | 4 | 3.128 | 1.421 | 0.895 | 1.166 | null | null |
| GO:0007560 | imaginal disc morphogenesis | 4 | 1.892 | 0.249 | 0.890 | 1.489 | null | null |
| GO:0009886 | post-embryonic animal morphogenesis | 4 | 0.602 | 0.015 | 0.921 | 0.863 | null | null |
| GO:0040024 | dauer larval development | 4 | 0.301 | 0.005 | 0.933 | 0.889 | null | null |
| GO:0048707 | instar larval or pupal morphogenesis | 4 | 0.602 | 0.154 | 0.905 | 1.422 | null | null |
| GO:0009798 | axis specification | 4 | 1.886 | 0.368 | 0.914 | 0.737 | null | null |
| GO:0007600 | sensory perception | 4 | 2.655 | 2.185 | 0.925 | 0.785 | null | null |
| GO:0043604 | amide biosynthetic process | 4 | 2.649 | 2.156 | 0.898 | 0.880 | null | null |
| GO:0006836 | neurotransmitter transport | 4 | 2.041 | 0.528 | 0.955 | 0.380 | 5.162 | 3.934 |
| GO:0006915 | apoptotic process | 4 | 2.496 | 1.511 | 0.985 | 0.019 | -2.673 | -7.760 |
| GO:0012501 | programmed cell death | 4 | 2.568 | 1.787 | 0.985 | 0.962 | null | null |
| GO:0097190 | apoptotic signaling pathway | 4 | 1.996 | 0.475 | 0.904 | 0.828 | null | null |
| GO:0097193 | intrinsic apoptotic signaling pathway | 4 | 1.716 | 0.247 | 0.907 | 0.775 | null | null |
| GO:0006941 | striated muscle contraction | 4 | 1.845 | 0.334 | 0.934 | 0.933 | null | null |
| GO:0043500 | muscle adaptation | 4 | 1.643 | 7.557 | 0.866 | 1.316 | null | null |
| GO:0007162 | negative regulation of cell adhesion | 4 | 2.061 | 0.552 | 0.906 | 0.448 | 5.999 | -1.245 |
| GO:0007163 | establishment or maintenance of cell polarity | 4 | 2.164 | 0.702 | 0.991 | 0.017 | -4.245 | -7.080 |
| GO:0007169 | transmembrane receptor protein tyrosine kinase signaling pathway | 4 | 2.493 | 1.502 | 0.896 | 0.597 | 0.070 | 5.854 |
| GO:0007204 | positive regulation of cytosolic calcium ion concentration | 4 | 2.017 | 0.499 | 0.945 | 0.512 | -1.616 | -2.574 |
| GO:0010564 | regulation of cell cycle process | 4 | 2.483 | 1.468 | 0.944 | 0.807 | null | null |
| GO:0003007 | heart morphogenesis | 4 | 2.382 | 1.163 | 0.879 | 0.717 | null | null |
| GO:0007552 | metamorphosis | 4 | 3.128 | 6.506 | 0.889 | 0.492 | -6.567 | 0.180 |
| GO:0007617 | mating behavior | 4 | 0.477 | 0.010 | 0.925 | 0.609 | -4.561 | 2.786 |
| GO:0045475 | locomotor rhythm | 4 | 0.602 | 0.015 | 0.950 | 0.909 | null | null |
| GO:0048512 | circadian behavior | 4 | 1.146 | 0.063 | 0.944 | 0.795 | null | null |
| GO:0007611 | learning or memory | 4 | 1.362 | 0.107 | 0.934 | 0.732 | null | null |
| GO:0008219 | cell death | 4 | 2.568 | 1.787 | 0.990 | 0.019 | -4.516 | -7.019 |
| GO:0008285 | negative regulation of cell population proliferation | 4 | 2.061 | 0.552 | 0.912 | 0.805 | null | null |
| GO:0007629 | flight behavior | 4 | 0.477 | 0.010 | 0.952 | 0.797 | null | null |
| GO:0009612 | response to mechanical stimulus | 4 | 1.748 | 0.266 | 0.944 | 0.730 | null | null |
| GO:0071214 | cellular response to abiotic stimulus | 4 | 1.898 | 0.378 | 0.943 | 0.754 | null | null |
| GO:0048598 | embryonic morphogenesis | 4 | 2.868 | 3.570 | 0.880 | 0.730 | null | null |
| GO:0032869 | cellular response to insulin stimulus | 4 | 1.708 | 0.242 | 0.915 | 0.916 | null | null |
| GO:0010830 | regulation of myotube differentiation | 4 | 1.230 | 0.078 | 0.907 | 0.532 | 6.153 | -2.995 |
| GO:0051153 | regulation of striated muscle cell differentiation | 4 | 1.322 | 0.097 | 0.906 | 0.957 | null | null |
| GO:0002790 | peptide secretion | 4 | 1.204 | 0.073 | 0.951 | 0.836 | null | null |
| GO:0015931 | nucleobase-containing compound transport | 4 | 2.155 | 0.688 | 0.948 | 0.546 | 4.821 | 4.552 |
| GO:0016358 | dendrite development | 4 | 1.505 | 0.150 | 0.873 | 0.594 | -6.014 | 1.933 |
| GO:0072330 | monocarboxylic acid biosynthetic process | 4 | 2.000 | 0.480 | 0.901 | 0.929 | null | null |
| GO:1901605 | alpha-amino acid metabolic process | 4 | 2.236 | 0.828 | 0.897 | 0.835 | null | null |
| GO:0019882 | antigen processing and presentation | 4 | 1.591 | 0.184 | 0.982 | 0.573 | -2.218 | 2.243 |
| GO:0022411 | cellular component disassembly | 4 | 2.173 | 0.717 | 0.963 | 0.283 | -5.005 | 5.954 |
| GO:0007618 | mating | 4 | 0.477 | 0.426 | 0.954 | 0.834 | null | null |
| GO:0008406 | gonad development | 4 | 1.362 | 0.107 | 0.885 | 0.986 | null | null |
| GO:0045137 | development of primary sexual characteristics | 4 | 1.380 | 0.111 | 0.895 | 0.731 | null | null |
| GO:0048515 | spermatid differentiation | 4 | 1.613 | 0.194 | 0.881 | 0.770 | null | null |
| GO:1903046 | meiotic cell cycle process | 4 | 1.914 | 0.392 | 0.936 | 0.827 | null | null |
| GO:0022900 | electron transport chain | 4 | 1.929 | 0.407 | 0.940 | 0.214 | 3.425 | -5.928 |
| GO:0015980 | energy derivation by oxidation of organic compounds | 4 | 2.233 | 0.823 | 0.936 | 0.862 | null | null |
| GO:0030278 | regulation of ossification | 4 | 1.431 | 0.126 | 0.917 | 0.553 | 5.240 | -4.356 |
| GO:0045088 | regulation of innate immune response | 4 | 2.076 | 0.572 | 0.907 | 0.814 | null | null |
| GO:0031668 | cellular response to extracellular stimulus | 4 | 1.785 | 0.291 | 0.939 | 0.980 | null | null |
| GO:0031669 | cellular response to nutrient levels | 4 | 1.771 | 0.281 | 0.939 | 0.944 | null | null |
| GO:0042594 | response to starvation | 4 | 1.839 | 0.329 | 0.943 | 0.958 | null | null |
| GO:0032101 | regulation of response to external stimulus | 4 | 2.587 | 1.865 | 0.924 | 0.581 | 6.786 | 0.280 |
| GO:0032469 | endoplasmic reticulum calcium ion homeostasis | 4 | 0.778 | 0.024 | 0.979 | 0.529 | -3.678 | -4.923 |
| GO:0032535 | regulation of cellular component size | 4 | 2.378 | 1.153 | 0.903 | 0.560 | -3.894 | 4.503 |
| GO:0033044 | regulation of chromosome organization | 4 | 1.763 | 0.276 | 0.917 | 0.581 | 1.486 | -2.164 |
| GO:0097553 | calcium ion transmembrane import into cytosol | 4 | 1.839 | 0.329 | 0.941 | 0.876 | null | null |
| GO:0034248 | regulation of amide metabolic process | 4 | 2.441 | 1.332 | 0.917 | 0.352 | 3.304 | 0.857 |
| GO:0035914 | skeletal muscle cell differentiation | 4 | 1.079 | 0.053 | 0.913 | 0.696 | -7.233 | -1.793 |
| GO:0040029 | epigenetic regulation of gene expression | 4 | 1.813 | 0.310 | 0.890 | 0.588 | -3.197 | 4.498 |
| GO:0043009 | chordate embryonic development | 4 | 2.721 | 2.543 | 0.894 | 0.696 | -6.404 | 0.395 |
| GO:1902115 | regulation of organelle assembly | 4 | 1.875 | 0.358 | 0.911 | 0.752 | null | null |
| GO:0006650 | glycerophospholipid metabolic process | 4 | 2.360 | 1.104 | 0.895 | 0.899 | null | null |
| GO:0045017 | glycerolipid biosynthetic process | 4 | 2.188 | 0.741 | 0.909 | 0.715 | null | null |
| GO:0044272 | sulfur compound biosynthetic process | 4 | 1.987 | 0.465 | 0.927 | 0.423 | 2.782 | -5.498 |
| GO:0045807 | positive regulation of endocytosis | 4 | 1.114 | 0.058 | 0.863 | 0.621 | 2.723 | -1.963 |
| GO:0034764 | positive regulation of transmembrane transport | 4 | 1.362 | 0.107 | 0.884 | 0.738 | null | null |
| GO:0032434 | regulation of proteasomal ubiquitin-dependent protein catabolic process | 4 | 1.771 | 0.281 | 0.898 | 0.968 | null | null |
| GO:1901800 | positive regulation of proteasomal protein catabolic process | 4 | 1.763 | 0.276 | 0.862 | 0.978 | null | null |
| GO:2000060 | positive regulation of ubiquitin-dependent protein catabolic process | 4 | 1.681 | 0.228 | 0.865 | 0.960 | null | null |
| GO:0046700 | heterocycle catabolic process | 4 | 2.407 | 1.230 | 0.904 | 0.649 | 2.777 | -5.986 |
| GO:0072659 | protein localization to plasma membrane | 4 | 2.053 | 0.543 | 0.939 | 0.853 | null | null |
| GO:0048002 | antigen processing and presentation of peptide antigen | 4 | 1.491 | 0.145 | 0.978 | 0.560 | -2.265 | 2.399 |
| GO:0001558 | regulation of cell growth | 4 | 2.053 | 0.543 | 0.904 | 0.937 | null | null |
| GO:0045926 | negative regulation of growth | 4 | 1.771 | 0.281 | 0.913 | 0.881 | null | null |
| GO:0048732 | gland development | 4 | 2.422 | 1.274 | 0.920 | 0.483 | -6.493 | -1.158 |
| GO:0048872 | homeostasis of number of cells | 4 | 2.188 | 0.741 | 0.923 | 0.737 | null | null |
| GO:0051480 | regulation of cytosolic calcium ion concentration | 4 | 1.623 | 0.199 | 0.975 | 0.888 | null | null |
| GO:0002694 | regulation of leukocyte activation | 4 | 2.204 | 0.770 | 0.884 | 0.782 | null | null |
| GO:0006469 | negative regulation of protein kinase activity | 4 | 0.778 | 0.024 | 0.857 | 0.971 | null | null |
| GO:0022898 | regulation of transmembrane transporter activity | 4 | 0.778 | 0.024 | 0.897 | 0.987 | null | null |
| GO:0032409 | regulation of transporter activity | 4 | 0.778 | 0.024 | 0.934 | 0.811 | null | null |
| GO:0032412 | regulation of monoatomic ion transmembrane transporter activity | 4 | 0.699 | 0.019 | 0.898 | 0.798 | null | null |
| GO:0033673 | negative regulation of kinase activity | 4 | 0.778 | 0.024 | 0.868 | 0.971 | null | null |
| GO:0033674 | positive regulation of kinase activity | 4 | 0.602 | 0.015 | 0.869 | 0.941 | null | null |
| GO:0045860 | positive regulation of protein kinase activity | 4 | 0.602 | 0.015 | 0.859 | 0.941 | null | null |
| GO:0051347 | positive regulation of transferase activity | 4 | 0.903 | 0.034 | 0.929 | 0.888 | null | null |
| GO:0051348 | negative regulation of transferase activity | 4 | 1.000 | 0.044 | 0.928 | 0.937 | null | null |
| GO:0052547 | regulation of peptidase activity | 4 | 0.602 | 0.015 | 0.896 | 0.783 | null | null |
| GO:1901019 | regulation of calcium ion transmembrane transporter activity | 4 | 0.477 | 0.010 | 0.900 | 0.936 | null | null |
| GO:0008582 | regulation of synaptic assembly at neuromuscular junction | 4 | 0.477 | 0.010 | 0.883 | 0.766 | null | null |
| GO:0051963 | regulation of synapse assembly | 4 | 1.462 | 0.136 | 0.908 | 0.927 | null | null |
| GO:1904396 | regulation of neuromuscular junction development | 4 | 0.778 | 0.024 | 0.923 | 0.815 | null | null |
| GO:0050829 | defense response to Gram-negative bacterium | 4 | 1.362 | 0.107 | 0.950 | 0.686 | 1.501 | 6.555 |
| GO:0050865 | regulation of cell activation | 4 | 2.223 | 0.804 | 0.900 | 0.674 | 5.099 | -4.161 |
| GO:0050890 | cognition | 4 | 1.380 | 0.111 | 0.943 | 0.475 | -6.940 | 3.314 |
| GO:0034767 | positive regulation of monoatomic ion transmembrane transport | 4 | 1.146 | 0.063 | 0.886 | 0.958 | null | null |
| GO:0051282 | regulation of sequestering of calcium ion | 4 | 1.748 | 0.266 | 0.920 | 0.718 | null | null |
| GO:0060341 | regulation of cellular localization | 4 | 2.170 | 0.712 | 0.913 | 0.798 | null | null |
| GO:1904064 | positive regulation of cation transmembrane transport | 4 | 1.114 | 0.058 | 0.886 | 0.919 | null | null |
| GO:1904427 | positive regulation of calcium ion transmembrane transport | 4 | 0.602 | 0.015 | 0.895 | 0.843 | null | null |
| GO:0031110 | regulation of microtubule polymerization or depolymerization | 4 | 1.708 | 0.242 | 0.915 | 0.881 | null | null |
| GO:0051495 | positive regulation of cytoskeleton organization | 4 | 1.869 | 0.354 | 0.873 | 0.735 | null | null |
| GO:1902904 | negative regulation of supramolecular fiber organization | 4 | 1.875 | 0.358 | 0.880 | 0.849 | null | null |
| GO:0051147 | regulation of muscle cell differentiation | 4 | 1.398 | 0.116 | 0.908 | 0.553 | 6.058 | -3.128 |
| GO:0051235 | maintenance of location | 4 | 1.851 | 0.339 | 0.960 | 0.325 | 4.294 | 4.805 |
| GO:0006417 | regulation of translation | 4 | 2.430 | 1.298 | 0.891 | 0.790 | null | null |
| GO:0051262 | protein tetramerization | 4 | 1.519 | 0.155 | 0.962 | 0.867 | null | null |
| GO:0051289 | protein homotetramerization | 4 | 1.279 | 0.087 | 0.963 | 0.826 | null | null |
| GO:0051302 | regulation of cell division | 4 | 1.748 | 0.266 | 0.955 | 0.071 | -2.408 | -5.493 |
| GO:0051651 | maintenance of location in cell | 4 | 1.613 | 0.194 | 0.946 | 0.305 | 4.340 | 3.910 |
| GO:0014904 | myotube cell development | 4 | 1.839 | 0.329 | 0.911 | 0.954 | null | null |
| GO:0045214 | sarcomere organization | 4 | 1.756 | 0.271 | 0.865 | 0.921 | null | null |
| GO:0048741 | skeletal muscle fiber development | 4 | 1.833 | 0.325 | 0.895 | 0.859 | null | null |
| GO:0055088 | lipid homeostasis | 4 | 1.929 | 0.407 | 0.976 | 0.690 | -3.714 | -4.740 |
| GO:0060349 | bone morphogenesis | 4 | 1.623 | 0.199 | 0.897 | 0.654 | -6.025 | -0.162 |
| GO:0061448 | connective tissue development | 4 | 2.246 | 0.848 | 0.925 | 0.603 | -6.996 | -1.643 |
| GO:0070296 | sarcoplasmic reticulum calcium ion transport | 4 | 0.845 | 0.029 | 0.959 | 0.282 | 4.060 | 4.435 |
| GO:0071229 | cellular response to acid chemical | 4 | 1.322 | 0.097 | 0.934 | 0.477 | -1.025 | 6.358 |
| GO:0043200 | response to amino acid | 4 | 1.415 | 0.121 | 0.933 | 0.982 | null | null |
| GO:0071396 | cellular response to lipid | 4 | 2.173 | 0.717 | 0.916 | 0.642 | -0.644 | 6.428 |
| GO:0014819 | regulation of skeletal muscle contraction | 4 | 0.477 | 0.010 | 0.917 | 0.787 | null | null |
| GO:0043502 | regulation of muscle adaptation | 4 | 0.699 | 0.019 | 0.899 | 0.824 | null | null |
| GO:0104004 | cellular response to environmental stimulus | 4 | 1.898 | 0.378 | 0.956 | 0.198 | 0.458 | 5.284 |
| GO:0010977 | negative regulation of neuron projection development | 4 | 1.748 | 0.266 | 0.882 | 0.834 | null | null |
| GO:0009100 | glycoprotein metabolic process | 4 | 2.464 | 1.405 | 0.911 | 0.789 | null | null |
| GO:0009101 | glycoprotein biosynthetic process | 4 | 2.413 | 1.250 | 0.887 | 0.778 | null | null |
| GO:1901136 | carbohydrate derivative catabolic process | 4 | 2.185 | 0.736 | 0.914 | 0.730 | null | null |
| GO:1901361 | organic cyclic compound catabolic process | 4 | 2.462 | 1.400 | 0.912 | 0.660 | 1.615 | -5.576 |
| GO:1902105 | regulation of leukocyte differentiation | 4 | 1.748 | 0.266 | 0.861 | 0.700 | 5.731 | -3.259 |
| GO:1902905 | positive regulation of supramolecular fiber organization | 4 | 1.833 | 0.325 | 0.875 | 0.590 | 3.166 | -2.092 |
| GO:1903311 | regulation of mRNA metabolic process | 4 | 2.276 | 0.911 | 0.915 | 0.336 | 2.988 | 0.511 |
| GO:1903779 | regulation of cardiac conduction | 4 | 0.477 | 0.010 | 0.923 | 0.620 | 5.157 | -4.380 |
| GO:0003333 | amino acid transmembrane transport | 4 | 2.188 | 0.741 | 0.934 | 0.942 | null | null |
| GO:0006865 | amino acid transport | 4 | 2.217 | 0.794 | 0.944 | 0.898 | null | null |
| GO:0045665 | negative regulation of neuron differentiation | 4 | 1.000 | 0.044 | 0.884 | 0.794 | null | null |
| GO:0000280 | nuclear division | 3 | 2.179 | 0.727 | 0.954 | 0.454 | -4.542 | 6.042 |
| GO:0001708 | cell fate specification | 3 | 2.029 | 0.513 | 0.921 | 0.409 | -7.607 | -1.355 |
| GO:0007472 | wing disc morphogenesis | 3 | 0.602 | 0.077 | 0.900 | 1.336 | null | null |
| GO:0002244 | hematopoietic progenitor cell differentiation | 3 | 2.000 | 0.480 | 0.915 | 0.492 | -7.574 | -1.135 |
| GO:0002700 | regulation of production of molecular mediator of immune response | 3 | 1.415 | 0.121 | 0.909 | 0.649 | 4.126 | 0.687 |
| GO:0002699 | positive regulation of immune effector process | 3 | 1.591 | 0.184 | 0.900 | 0.892 | null | null |
| GO:0002920 | regulation of humoral immune response | 3 | 1.204 | 0.073 | 0.924 | 0.620 | 6.817 | -0.072 |
| GO:0009880 | embryonic pattern specification | 3 | 1.863 | 0.349 | 0.910 | 0.733 | null | null |
| GO:0003205 | cardiac chamber development | 3 | 1.643 | 0.208 | 0.908 | 0.591 | -7.244 | 0.122 |
| GO:0005996 | monosaccharide metabolic process | 3 | 1.929 | 0.407 | 0.924 | 0.581 | 1.423 | -7.239 |
| GO:0006022 | aminoglycan metabolic process | 3 | 1.903 | 0.383 | 0.925 | 0.678 | 0.324 | -6.309 |
| GO:0006403 | RNA localization | 3 | 1.996 | 0.475 | 0.954 | 0.338 | 5.288 | 3.944 |
| GO:0006470 | protein dephosphorylation | 3 | 0.699 | 0.019 | 0.929 | 0.466 | 0.214 | -7.085 |
| GO:0006412 | translation | 3 | 2.543 | 1.686 | 0.886 | 0.853 | null | null |
| GO:0043043 | peptide biosynthetic process | 3 | 2.550 | 1.715 | 0.890 | 0.921 | null | null |
| GO:0006638 | neutral lipid metabolic process | 3 | 1.799 | 0.300 | 0.930 | 0.645 | 2.612 | -6.662 |
| GO:0006890 | retrograde vesicle-mediated transport, Golgi to endoplasmic reticulum | 3 | 1.544 | 0.165 | 0.947 | 0.529 | 4.927 | 3.673 |
| GO:0006898 | receptor-mediated endocytosis | 3 | 1.940 | 0.417 | 0.943 | 0.756 | null | null |
| GO:0006900 | vesicle budding from membrane | 3 | 1.602 | 0.189 | 0.914 | 0.601 | 2.671 | 4.412 |
| GO:0070059 | intrinsic apoptotic signaling pathway in response to endoplasmic reticulum stress | 3 | 1.041 | 0.048 | 0.910 | 0.781 | null | null |
| GO:0014888 | striated muscle adaptation | 3 | 1.643 | 0.630 | 0.897 | 0.991 | null | null |
| GO:0090075 | relaxation of muscle | 3 | 0.602 | 0.015 | 0.948 | 0.723 | null | null |
| GO:0007006 | mitochondrial membrane organization | 3 | 1.716 | 0.247 | 0.954 | 0.404 | -4.174 | 5.770 |
| GO:0022407 | regulation of cell-cell adhesion | 3 | 2.204 | 0.770 | 0.941 | 0.884 | null | null |
| GO:0045785 | positive regulation of cell adhesion | 3 | 2.045 | 0.533 | 0.900 | 0.852 | null | null |
| GO:1903037 | regulation of leukocyte cell-cell adhesion | 3 | 2.041 | 0.528 | 0.942 | 0.852 | null | null |
| GO:0007274 | neuromuscular synaptic transmission | 3 | 1.342 | 0.102 | 0.945 | 0.714 | null | null |
| GO:0045787 | positive regulation of cell cycle | 3 | 2.064 | 0.557 | 0.904 | 0.720 | null | null |
| GO:0030900 | forebrain development | 3 | 2.190 | 0.746 | 0.891 | 0.818 | null | null |
| GO:0007422 | peripheral nervous system development | 3 | 1.863 | 0.349 | 0.907 | 0.500 | -7.001 | 0.836 |
| GO:0007525 | somatic muscle development | 3 | 0.903 | 0.034 | 0.943 | 0.618 | -7.000 | -2.762 |
| GO:0008037 | cell recognition | 3 | 1.924 | 0.402 | 0.992 | 0.016 | -3.023 | -7.657 |
| GO:0006754 | ATP biosynthetic process | 3 | 1.447 | 0.131 | 0.878 | 0.970 | null | null |
| GO:0009142 | nucleoside triphosphate biosynthetic process | 3 | 1.740 | 0.262 | 0.885 | 0.920 | null | null |
| GO:0009145 | purine nucleoside triphosphate biosynthetic process | 3 | 1.602 | 0.189 | 0.888 | 0.946 | null | null |
| GO:0009201 | ribonucleoside triphosphate biosynthetic process | 3 | 1.690 | 0.233 | 0.886 | 0.962 | null | null |
| GO:0009206 | purine ribonucleoside triphosphate biosynthetic process | 3 | 1.602 | 0.189 | 0.888 | 0.894 | null | null |
| GO:0009124 | nucleoside monophosphate biosynthetic process | 3 | 1.716 | 0.247 | 0.886 | 0.940 | null | null |
| GO:0009127 | purine nucleoside monophosphate biosynthetic process | 3 | 1.447 | 0.131 | 0.892 | 0.972 | null | null |
| GO:0009156 | ribonucleoside monophosphate biosynthetic process | 3 | 1.633 | 0.203 | 0.888 | 0.952 | null | null |
| GO:0009168 | purine ribonucleoside monophosphate biosynthetic process | 3 | 1.447 | 0.131 | 0.892 | 0.972 | null | null |
| GO:0001666 | response to hypoxia | 3 | 1.914 | 0.392 | 0.944 | 0.757 | null | null |
| GO:0009581 | detection of external stimulus | 3 | 1.964 | 0.441 | 0.951 | 0.785 | null | null |
| GO:0009582 | detection of abiotic stimulus | 3 | 1.964 | 0.441 | 0.951 | 0.766 | null | null |
| GO:0036293 | response to decreased oxygen levels | 3 | 1.919 | 0.397 | 0.953 | 0.982 | null | null |
| GO:0070482 | response to oxygen levels | 3 | 1.949 | 0.426 | 0.953 | 0.763 | null | null |
| GO:0071478 | cellular response to radiation | 3 | 1.826 | 0.320 | 0.943 | 0.982 | null | null |
| GO:0009409 | response to cold | 3 | 1.230 | 0.078 | 0.951 | 0.654 | 1.494 | 6.711 |
| GO:0048568 | embryonic organ development | 3 | 2.809 | 3.115 | 0.883 | 0.751 | null | null |
| GO:0070555 | response to interleukin-1 | 3 | 1.602 | 0.189 | 0.936 | 0.797 | null | null |
| GO:0048738 | cardiac muscle tissue development | 3 | 2.037 | 0.523 | 0.890 | 0.847 | null | null |
| GO:0045844 | positive regulation of striated muscle tissue development | 3 | 0.301 | 0.005 | 0.880 | 0.863 | null | null |
| GO:0048636 | positive regulation of muscle organ development | 3 | 0.301 | 0.005 | 0.883 | 0.819 | null | null |
| GO:0048643 | positive regulation of skeletal muscle tissue development | 3 | 0.699 | 0.019 | 0.891 | 0.937 | null | null |
| GO:1901863 | positive regulation of muscle tissue development | 3 | 0.778 | 0.024 | 0.890 | 0.950 | null | null |
| GO:0016311 | dephosphorylation | 3 | 2.297 | 0.954 | 0.925 | 0.541 | 2.881 | -6.323 |
| GO:0018209 | peptidyl-serine modification | 3 | 0.602 | 0.015 | 0.944 | 0.455 | -0.299 | -7.089 |
| GO:0019216 | regulation of lipid metabolic process | 3 | 1.940 | 0.417 | 0.926 | 0.302 | 3.534 | 0.749 |
| GO:0021675 | nerve development | 3 | 1.903 | 0.383 | 0.907 | 0.505 | -6.897 | 0.848 |
| GO:0007293 | germarium-derived egg chamber formation | 3 | 1.279 | 0.141 | 0.869 | 0.903 | null | null |
| GO:0048599 | oocyte development | 3 | 1.602 | 0.189 | 0.876 | 0.925 | null | null |
| GO:0006119 | oxidative phosphorylation | 3 | 1.845 | 0.334 | 0.940 | 0.900 | null | null |
| GO:0022904 | respiratory electron transport chain | 3 | 1.898 | 0.378 | 0.940 | 0.802 | null | null |
| GO:0045333 | cellular respiration | 3 | 2.100 | 0.606 | 0.937 | 0.897 | null | null |
| GO:0030203 | glycosaminoglycan metabolic process | 3 | 1.792 | 0.295 | 0.922 | 0.660 | 0.238 | -6.325 |
| GO:0006026 | aminoglycan catabolic process | 3 | 1.342 | 0.102 | 0.915 | 0.872 | null | null |
| GO:0006027 | glycosaminoglycan catabolic process | 3 | 1.230 | 0.078 | 0.916 | 0.854 | null | null |
| GO:0030322 | stabilization of membrane potential | 3 | 1.362 | 0.107 | 0.950 | 0.441 | -1.354 | -2.586 |
| GO:0030335 | positive regulation of cell migration | 3 | 2.149 | 0.678 | 0.898 | 0.897 | null | null |
| GO:0040017 | positive regulation of locomotion | 3 | 2.199 | 0.761 | 0.903 | 0.892 | null | null |
| GO:2000147 | positive regulation of cell motility | 3 | 2.173 | 0.717 | 0.897 | 0.975 | null | null |
| GO:0031146 | SCF-dependent proteasomal ubiquitin-dependent protein catabolic process | 3 | 1.653 | 0.213 | 0.921 | 0.529 | 0.649 | -6.921 |
| GO:0010507 | negative regulation of autophagy | 3 | 1.362 | 0.107 | 0.890 | 0.872 | null | null |
| GO:0016241 | regulation of macroautophagy | 3 | 1.447 | 0.131 | 0.920 | 0.723 | null | null |
| GO:0050727 | regulation of inflammatory response | 3 | 2.004 | 0.484 | 0.931 | 0.854 | null | null |
| GO:0017148 | negative regulation of translation | 3 | 1.748 | 0.266 | 0.872 | 0.840 | null | null |
| GO:0031670 | cellular response to nutrient | 3 | 0.602 | 0.015 | 0.931 | 0.747 | null | null |
| GO:0032006 | regulation of TOR signaling | 3 | 1.898 | 0.378 | 0.922 | 0.652 | 6.608 | 0.540 |
| GO:0032470 | positive regulation of endoplasmic reticulum calcium ion concentration | 3 | 0.301 | 0.005 | 0.981 | 0.565 | -3.789 | -4.995 |
| GO:0032872 | regulation of stress-activated MAPK cascade | 3 | 0.903 | 0.034 | 0.933 | 0.630 | 6.680 | 0.578 |
| GO:0007269 | neurotransmitter secretion | 3 | 1.934 | 0.412 | 0.884 | 0.884 | null | null |
| GO:0009306 | protein secretion | 3 | 1.681 | 0.228 | 0.932 | 0.835 | null | null |
| GO:0099643 | signal release from synapse | 3 | 1.934 | 0.412 | 0.891 | 0.957 | null | null |
| GO:0070509 | calcium ion import | 3 | 1.544 | 0.165 | 0.953 | 0.821 | null | null |
| GO:1903515 | calcium ion transport from cytosol to endoplasmic reticulum | 3 | 1.544 | 0.116 | 0.938 | 0.795 | null | null |
| GO:0033539 | fatty acid beta-oxidation using acyl-CoA dehydrogenase | 3 | 1.000 | 0.044 | 0.904 | 0.714 | null | null |
| GO:0000302 | response to reactive oxygen species | 3 | 1.544 | 0.165 | 0.931 | 0.864 | null | null |
| GO:0042542 | response to hydrogen peroxide | 3 | 1.176 | 0.068 | 0.933 | 0.807 | null | null |
| GO:0001525 | angiogenesis | 3 | 2.470 | 1.424 | 0.884 | 0.916 | null | null |
| GO:0036335 | intestinal stem cell homeostasis | 3 | 0.301 | 0.005 | 0.948 | 0.467 | -4.220 | -3.472 |
| GO:0006325 | chromatin organization | 3 | 2.684 | 2.335 | 0.954 | 0.753 | null | null |
| GO:0050730 | regulation of peptidyl-tyrosine phosphorylation | 3 | 1.602 | 0.189 | 0.898 | 0.846 | null | null |
| GO:0042752 | regulation of circadian rhythm | 3 | 1.643 | 0.208 | 0.960 | 0.069 | -4.115 | -6.553 |
| GO:0043065 | positive regulation of apoptotic process | 3 | 2.230 | 0.819 | 0.898 | 0.782 | null | null |
| GO:0043112 | receptor metabolic process | 3 | 1.230 | 0.078 | 0.953 | 0.253 | -0.475 | -6.491 |
| GO:0043279 | response to alkaloid | 3 | 0.699 | 0.019 | 0.944 | 0.411 | -1.400 | 6.946 |
| GO:0032271 | regulation of protein polymerization | 3 | 2.149 | 0.678 | 0.908 | 0.800 | null | null |
| GO:0043254 | regulation of protein-containing complex assembly | 3 | 2.207 | 0.775 | 0.907 | 0.814 | null | null |
| GO:0006639 | acylglycerol metabolic process | 3 | 1.792 | 0.295 | 0.927 | 0.789 | null | null |
| GO:0006641 | triglyceride metabolic process | 3 | 1.613 | 0.194 | 0.930 | 0.959 | null | null |
| GO:0008654 | phospholipid biosynthetic process | 3 | 2.217 | 0.794 | 0.888 | 0.781 | null | null |
| GO:0046474 | glycerophospholipid biosynthetic process | 3 | 2.140 | 0.664 | 0.888 | 0.853 | null | null |
| GO:0044706 | multi-multicellular organism process | 3 | 0.477 | 0.010 | 0.961 | 0.145 | -7.240 | 3.831 |
| GO:0045598 | regulation of fat cell differentiation | 3 | 1.000 | 0.044 | 0.915 | 0.506 | 6.206 | -3.036 |
| GO:0045600 | positive regulation of fat cell differentiation | 3 | 0.954 | 0.039 | 0.882 | 0.601 | 5.717 | -2.575 |
| GO:0050764 | regulation of phagocytosis | 3 | 0.903 | 0.034 | 0.901 | 0.761 | null | null |
| GO:0032436 | positive regulation of proteasomal ubiquitin-dependent protein catabolic process | 3 | 1.672 | 0.223 | 0.865 | 0.961 | null | null |
| GO:0045861 | negative regulation of proteolysis | 3 | 1.362 | 0.107 | 0.881 | 0.826 | null | null |
| GO:0046626 | regulation of insulin receptor signaling pathway | 3 | 1.204 | 0.073 | 0.934 | 0.392 | 6.609 | 0.293 |
| GO:0046627 | negative regulation of insulin receptor signaling pathway | 3 | 1.114 | 0.058 | 0.906 | 0.385 | 6.266 | 0.068 |
| GO:1900077 | negative regulation of cellular response to insulin stimulus | 3 | 1.114 | 0.058 | 0.911 | 0.952 | null | null |
| GO:0046683 | response to organophosphorus | 3 | 1.146 | 0.063 | 0.943 | 0.457 | -1.197 | 6.879 |
| GO:0019439 | aromatic compound catabolic process | 3 | 2.436 | 1.318 | 0.903 | 0.730 | null | null |
| GO:0034655 | nucleobase-containing compound catabolic process | 3 | 2.346 | 1.071 | 0.896 | 0.713 | null | null |
| GO:0044270 | cellular nitrogen compound catabolic process | 3 | 2.410 | 1.240 | 0.901 | 0.725 | null | null |
| GO:0006605 | protein targeting | 3 | 2.173 | 0.717 | 0.937 | 0.749 | null | null |
| GO:0002478 | antigen processing and presentation of exogenous peptide antigen | 3 | 1.398 | 0.116 | 0.978 | 0.946 | null | null |
| GO:0019884 | antigen processing and presentation of exogenous antigen | 3 | 1.398 | 0.116 | 0.978 | 0.946 | null | null |
| GO:0042551 | neuron maturation | 3 | 1.114 | 0.058 | 0.900 | 0.826 | null | null |
| GO:0030308 | negative regulation of cell growth | 3 | 1.724 | 0.252 | 0.880 | 0.873 | null | null |
| GO:0048813 | dendrite morphogenesis | 3 | 1.255 | 0.082 | 0.872 | 0.558 | -5.968 | 1.788 |
| GO:0048863 | stem cell differentiation | 3 | 2.367 | 1.124 | 0.915 | 0.451 | -7.494 | -0.979 |
| GO:0050673 | epithelial cell proliferation | 3 | 1.591 | 0.184 | 0.991 | 0.014 | -3.374 | -7.157 |
| GO:0050684 | regulation of mRNA processing | 3 | 1.973 | 0.450 | 0.920 | 0.310 | 3.136 | 0.487 |
| GO:0002684 | positive regulation of immune system process | 3 | 2.525 | 1.618 | 0.880 | 0.851 | null | null |
| GO:0050863 | regulation of T cell activation | 3 | 2.093 | 0.596 | 0.887 | 0.958 | null | null |
| GO:0051249 | regulation of lymphocyte activation | 3 | 2.158 | 0.693 | 0.885 | 0.973 | null | null |
| GO:0010952 | positive regulation of peptidase activity | 3 | 0.301 | 0.005 | 0.875 | 0.729 | null | null |
| GO:0032411 | positive regulation of transporter activity | 3 | 0.699 | 0.019 | 0.872 | 0.987 | null | null |
| GO:0032414 | positive regulation of ion transmembrane transporter activity | 3 | 0.602 | 0.015 | 0.867 | 0.958 | null | null |
| GO:0032781 | positive regulation of ATP-dependent activity | 3 | 0.301 | 0.005 | 0.938 | 1.024 | null | null |
| GO:0043405 | regulation of MAP kinase activity | 3 | 0.602 | 0.015 | 0.888 | 0.977 | null | null |
| GO:0043462 | regulation of ATP-dependent activity | 3 | 0.301 | 0.005 | 0.940 | 0.729 | null | null |
| GO:0071902 | positive regulation of protein serine/threonine kinase activity | 3 | 0.477 | 0.010 | 0.862 | 0.977 | null | null |
| GO:1901021 | positive regulation of calcium ion transmembrane transporter activity | 3 | 0.301 | 0.005 | 0.875 | 0.953 | null | null |
| GO:1901894 | regulation of ATPase-coupled calcium transmembrane transporter activity | 3 | 0.301 | 0.012 | 0.898 | 1.074 | null | null |
| GO:1901896 | positive regulation of ATPase-coupled calcium transmembrane transporter activity | 3 | 0.301 | 0.008 | 0.871 | 0.924 | null | null |
| GO:0050878 | regulation of body fluid levels | 3 | 2.029 | 0.513 | 0.945 | 0.513 | -1.378 | -2.473 |
| GO:0043271 | negative regulation of monoatomic ion transport | 3 | 1.230 | 0.078 | 0.894 | 0.800 | null | null |
| GO:0046883 | regulation of hormone secretion | 3 | 1.602 | 0.189 | 0.890 | 0.850 | null | null |
| GO:0090279 | regulation of calcium ion import | 3 | 0.699 | 0.019 | 0.930 | 0.781 | null | null |
| GO:0008064 | regulation of actin polymerization or depolymerization | 3 | 2.090 | 0.591 | 0.861 | 0.922 | null | null |
| GO:0030832 | regulation of actin filament length | 3 | 2.100 | 0.606 | 0.862 | 0.924 | null | null |
| GO:0031113 | regulation of microtubule polymerization | 3 | 1.204 | 0.073 | 0.917 | 0.797 | null | null |
| GO:0034249 | negative regulation of amide metabolic process | 3 | 1.748 | 0.266 | 0.892 | 0.830 | null | null |
| GO:0051606 | detection of stimulus | 3 | 2.442 | 1.337 | 0.961 | 0.232 | 0.496 | 5.593 |
| GO:0051656 | establishment of organelle localization | 3 | 2.384 | 1.167 | 0.946 | 0.406 | 4.791 | 3.897 |
| GO:0051646 | mitochondrion localization | 3 | 1.431 | 0.126 | 0.958 | 0.732 | null | null |
| GO:0051648 | vesicle localization | 3 | 2.117 | 0.630 | 0.952 | 0.855 | null | null |
| GO:0051650 | establishment of vesicle localization | 3 | 2.100 | 0.606 | 0.934 | 0.852 | null | null |
| GO:0051896 | regulation of phosphatidylinositol 3-kinase/protein kinase B signal transduction | 3 | 1.505 | 0.150 | 0.928 | 0.417 | 6.616 | 0.686 |
| GO:0051926 | negative regulation of calcium ion transport | 3 | 0.477 | 0.010 | 0.906 | 0.536 | 2.942 | -1.316 |
| GO:0055090 | acylglycerol homeostasis | 3 | 1.279 | 0.087 | 0.978 | 0.592 | -3.576 | -4.700 |
| GO:0042632 | cholesterol homeostasis | 3 | 1.613 | 0.194 | 0.977 | 0.825 | null | null |
| GO:0055092 | sterol homeostasis | 3 | 1.613 | 0.194 | 0.977 | 0.878 | null | null |
| GO:0070328 | triglyceride homeostasis | 3 | 1.114 | 0.058 | 0.979 | 0.756 | null | null |
| GO:0061640 | cytoskeleton-dependent cytokinesis | 3 | 1.968 | 0.446 | 0.971 | 0.689 | -1.688 | 0.005 |
| GO:0000910 | cytokinesis | 3 | 1.978 | 0.455 | 0.973 | 0.770 | null | null |
| GO:0032506 | cytokinetic process | 3 | 1.462 | 0.136 | 0.974 | 0.893 | null | null |
| GO:0033206 | meiotic cytokinesis | 3 | 0.699 | 0.019 | 0.947 | 0.769 | null | null |
| GO:0070302 | regulation of stress-activated protein kinase signaling cascade | 3 | 0.903 | 0.034 | 0.934 | 0.640 | 6.670 | 0.686 |
| GO:0070828 | heterochromatin organization | 3 | 1.732 | 0.257 | 0.963 | 0.655 | -4.360 | 5.240 |
| GO:0071230 | cellular response to amino acid stimulus | 3 | 1.322 | 0.097 | 0.921 | 0.982 | null | null |
| GO:0071236 | cellular response to antibiotic | 3 | 0.477 | 0.010 | 0.946 | 0.388 | -1.504 | 6.635 |
| GO:0072488 | ammonium transmembrane transport | 3 | 1.176 | 0.068 | 0.949 | 0.429 | 4.559 | 4.858 |
| GO:0090092 | regulation of transmembrane receptor protein serine/threonine kinase signaling pathway | 3 | 2.114 | 0.625 | 0.921 | 0.478 | 6.562 | 0.623 |
| GO:0014724 | regulation of twitch skeletal muscle contraction | 3 | 0.477 | 0.036 | 0.909 | 1.077 | null | null |
| GO:0031446 | regulation of fast-twitch skeletal muscle fiber contraction | 3 | 0.477 | 0.010 | 0.917 | 0.787 | null | null |
| GO:0031448 | positive regulation of fast-twitch skeletal muscle fiber contraction | 3 | 0.477 | 0.010 | 0.887 | 1.026 | null | null |
| GO:0045933 | positive regulation of muscle contraction | 3 | 0.602 | 0.015 | 0.886 | 0.808 | null | null |
| GO:0045989 | positive regulation of striated muscle contraction | 3 | 0.845 | 0.034 | 0.878 | 0.937 | null | null |
| GO:0055117 | regulation of cardiac muscle contraction | 3 | 1.176 | 0.068 | 0.905 | 0.900 | null | null |
| GO:0086004 | regulation of cardiac muscle cell contraction | 3 | 0.477 | 0.010 | 0.906 | 0.787 | null | null |
| GO:0097164 | ammonium ion metabolic process | 3 | 1.146 | 0.063 | 0.957 | 0.209 | -0.411 | -5.446 |
| GO:0097305 | response to alcohol | 3 | 1.342 | 0.102 | 0.937 | 0.540 | -1.142 | 6.698 |
| GO:0099003 | vesicle-mediated transport in synapse | 3 | 2.107 | 0.615 | 0.941 | 0.601 | 4.375 | 4.146 |
| GO:0099504 | synaptic vesicle cycle | 3 | 2.107 | 0.615 | 0.934 | 0.671 | 4.478 | 4.057 |
| GO:0110020 | regulation of actomyosin structure organization | 3 | 1.477 | 0.140 | 0.917 | 0.577 | 1.242 | -1.916 |
| GO:0010976 | positive regulation of neuron projection development | 3 | 1.531 | 0.160 | 0.882 | 0.794 | null | null |
| GO:0050773 | regulation of dendrite development | 3 | 1.431 | 0.126 | 0.878 | 0.777 | null | null |
| GO:0051489 | regulation of filopodium assembly | 3 | 1.322 | 0.097 | 0.918 | 0.886 | null | null |
| GO:0120034 | positive regulation of plasma membrane bounded cell projection assembly | 3 | 1.544 | 0.165 | 0.878 | 0.925 | null | null |
| GO:0030031 | cell projection assembly | 3 | 2.591 | 1.884 | 0.948 | 0.809 | null | null |
| GO:0120031 | plasma membrane bounded cell projection assembly | 3 | 2.568 | 1.787 | 0.948 | 0.803 | null | null |
| GO:1900076 | regulation of cellular response to insulin stimulus | 3 | 1.255 | 0.082 | 0.940 | 0.372 | 6.909 | 0.679 |
| GO:1901654 | response to ketone | 3 | 1.342 | 0.102 | 0.937 | 0.479 | -1.161 | 6.639 |
| GO:0045637 | regulation of myeloid cell differentiation | 3 | 1.663 | 0.218 | 0.863 | 0.913 | null | null |
| GO:1902275 | regulation of chromatin organization | 3 | 1.255 | 0.082 | 0.928 | 0.518 | 1.336 | -2.111 |
| GO:0043409 | negative regulation of MAPK cascade | 3 | 1.672 | 0.223 | 0.893 | 0.728 | null | null |
| GO:0043410 | positive regulation of MAPK cascade | 3 | 2.188 | 0.741 | 0.873 | 0.806 | null | null |
| GO:1903115 | regulation of actin filament-based movement | 3 | 0.602 | 0.015 | 0.955 | 0.053 | -0.886 | 0.078 |
| GO:1903429 | regulation of cell maturation | 3 | 0.699 | 0.019 | 0.916 | 0.503 | 6.213 | -2.954 |
| GO:1903431 | positive regulation of cell maturation | 3 | 1.230 | 0.081 | 0.871 | 0.550 | 5.691 | -2.600 |
| GO:1904888 | cranial skeletal system development | 3 | 2.364 | 1.114 | 0.933 | 0.277 | -6.492 | -1.592 |
| GO:0015807 | L-amino acid transport | 3 | 1.929 | 0.407 | 0.947 | 0.838 | null | null |
| GO:1902475 | L-alpha-amino acid transmembrane transport | 3 | 1.924 | 0.402 | 0.937 | 0.884 | null | null |
| GO:1905879 | regulation of oogenesis | 3 | 0.954 | 0.039 | 0.889 | 0.500 | 5.692 | -3.478 |
| GO:1990845 | adaptive thermogenesis | 3 | 0.845 | 0.029 | 0.970 | 0.119 | 4.620 | -5.448 |
| GO:2001014 | regulation of skeletal muscle cell differentiation | 3 | 0.699 | 0.019 | 0.919 | 0.472 | 6.225 | -2.910 |
| GO:0000041 | transition metal ion transport | 2 | 1.903 | 0.383 | 0.951 | 0.659 | 5.093 | 3.808 |
| GO:0000165 | MAPK cascade | 2 | 1.940 | 0.417 | 0.906 | 0.681 | 0.668 | 5.908 |
| GO:0045448 | mitotic cell cycle, embryonic | 2 | 1.908 | 0.388 | 0.891 | 0.805 | null | null |
| GO:0098813 | nuclear chromosome segregation | 2 | 2.045 | 0.533 | 0.973 | 0.702 | null | null |
| GO:0000070 | mitotic sister chromatid segregation | 2 | 1.806 | 0.305 | 0.943 | 0.888 | null | null |
| GO:0000266 | mitochondrial fission | 2 | 1.176 | 0.068 | 0.961 | 0.779 | null | null |
| GO:0000819 | sister chromatid segregation | 2 | 1.820 | 0.315 | 0.945 | 0.901 | null | null |
| GO:0140014 | mitotic nuclear division | 2 | 1.908 | 0.388 | 0.942 | 0.909 | null | null |
| GO:0001649 | osteoblast differentiation | 2 | 1.322 | 0.097 | 0.919 | 0.342 | -7.918 | -0.364 |
| GO:0042471 | ear morphogenesis | 2 | 2.013 | 0.494 | 0.886 | 0.926 | null | null |
| GO:0042472 | inner ear morphogenesis | 2 | 2.009 | 0.489 | 0.886 | 0.925 | null | null |
| GO:0043010 | camera-type eye development | 2 | 2.649 | 2.156 | 0.884 | 0.953 | null | null |
| GO:0043583 | ear development | 2 | 2.290 | 0.940 | 0.918 | 0.773 | null | null |
| GO:0048839 | inner ear development | 2 | 2.286 | 0.930 | 0.917 | 0.772 | null | null |
| GO:0071599 | otic vesicle development | 2 | 1.763 | 0.276 | 0.896 | 0.875 | null | null |
| GO:0001709 | cell fate determination | 2 | 1.041 | 0.048 | 0.935 | 0.729 | null | null |
| GO:0001751 | compound eye photoreceptor cell differentiation | 2 | 0.301 | 0.005 | 0.904 | 1.080 | null | null |
| GO:0042051 | compound eye photoreceptor development | 2 | 1.041 | 0.021 | 0.892 | 1.005 | null | null |
| GO:0001763 | morphogenesis of a branching structure | 2 | 1.462 | 0.136 | 0.920 | 0.385 | -7.679 | -0.165 |
| GO:0001889 | liver development | 2 | 2.212 | 0.785 | 0.900 | 0.454 | -6.832 | 0.012 |
| GO:0001890 | placenta development | 2 | 2.270 | 0.768 | 0.924 | 0.453 | -6.269 | -1.292 |
| GO:0001885 | endothelial cell development | 2 | 1.431 | 0.126 | 0.914 | 0.904 | null | null |
| GO:0045446 | endothelial cell differentiation | 2 | 1.708 | 0.242 | 0.913 | 0.739 | null | null |
| GO:0007476 | imaginal disc-derived wing morphogenesis | 2 | 0.602 | 0.014 | 0.909 | 0.861 | null | null |
| GO:0035120 | post-embryonic appendage morphogenesis | 2 | 1.176 | 0.158 | 0.902 | 1.425 | null | null |
| GO:0002521 | leukocyte differentiation | 2 | 2.068 | 0.562 | 0.914 | 0.698 | -7.529 | -1.104 |
| GO:0002702 | positive regulation of production of molecular mediator of immune response | 2 | 1.380 | 0.111 | 0.873 | 0.859 | null | null |
| GO:0009952 | anterior/posterior pattern specification | 2 | 2.403 | 1.221 | 0.903 | 0.849 | null | null |
| GO:0009953 | dorsal/ventral pattern formation | 2 | 2.190 | 0.746 | 0.908 | 0.799 | null | null |
| GO:0003151 | outflow tract morphogenesis | 2 | 0.778 | 0.024 | 0.913 | 0.408 | -7.530 | 0.094 |
| GO:0003158 | endothelium development | 2 | 1.820 | 0.315 | 0.930 | 0.535 | -7.073 | -1.952 |
| GO:0003231 | cardiac ventricle development | 2 | 1.431 | 0.126 | 0.911 | 0.562 | -7.405 | 0.223 |
| GO:0003206 | cardiac chamber morphogenesis | 2 | 1.431 | 0.126 | 0.901 | 0.923 | null | null |
| GO:0003254 | regulation of membrane depolarization | 2 | 1.230 | 0.078 | 0.945 | 0.613 | -1.508 | -2.644 |
| GO:0016051 | carbohydrate biosynthetic process | 2 | 1.851 | 0.339 | 0.936 | 0.730 | null | null |
| GO:0019318 | hexose metabolic process | 2 | 1.857 | 0.344 | 0.926 | 0.731 | null | null |
| GO:0006109 | regulation of carbohydrate metabolic process | 2 | 1.580 | 0.179 | 0.932 | 0.276 | 3.685 | 0.941 |
| GO:0006497 | protein lipidation | 2 | 1.672 | 0.223 | 0.911 | 0.592 | 0.673 | -6.540 |
| GO:0006501 | C-terminal protein lipidation | 2 | 1.079 | 0.054 | 0.920 | 0.887 | null | null |
| GO:0042158 | lipoprotein biosynthetic process | 2 | 1.690 | 0.233 | 0.915 | 0.924 | null | null |
| GO:0006600 | creatine metabolic process | 2 | 0.477 | 0.010 | 0.927 | 0.654 | 0.339 | -7.382 |
| GO:0006826 | iron ion transport | 2 | 1.653 | 0.213 | 0.953 | 0.617 | 5.182 | 3.755 |
| GO:0034755 | iron ion transmembrane transport | 2 | 1.146 | 0.063 | 0.949 | 0.820 | null | null |
| GO:0048250 | iron import into the mitochondrion | 2 | 0.477 | 0.010 | 0.955 | 0.720 | null | null |
| GO:0006855 | xenobiotic transmembrane transport | 2 | 0.602 | 0.015 | 0.959 | 0.265 | 5.462 | 3.552 |
| GO:0006885 | regulation of pH | 2 | 1.740 | 0.262 | 0.939 | 0.659 | -3.671 | -4.807 |
| GO:0006888 | endoplasmic reticulum to Golgi vesicle-mediated transport | 2 | 2.000 | 0.480 | 0.935 | 0.777 | null | null |
| GO:0006906 | vesicle fusion | 2 | 1.964 | 0.441 | 0.907 | 0.783 | null | null |
| GO:0090174 | organelle membrane fusion | 2 | 1.973 | 0.450 | 0.953 | 0.957 | null | null |
| GO:0006913 | nucleocytoplasmic transport | 2 | 2.111 | 0.620 | 0.938 | 0.671 | 4.951 | 4.017 |
| GO:0006914 | autophagy | 2 | 2.204 | 0.770 | 0.919 | 0.688 | 3.039 | -5.833 |
| GO:0000045 | autophagosome assembly | 2 | 1.771 | 0.281 | 0.893 | 0.899 | null | null |
| GO:0000422 | autophagy of mitochondrion | 2 | 1.279 | 0.087 | 0.904 | 0.811 | null | null |
| GO:0016236 | macroautophagy | 2 | 2.021 | 0.504 | 0.922 | 0.951 | null | null |
| GO:0044804 | nucleophagy | 2 | 1.415 | 0.121 | 0.932 | 0.834 | null | null |
| GO:1905037 | autophagosome organization | 2 | 1.778 | 0.286 | 0.896 | 0.901 | null | null |
| GO:0008637 | apoptotic mitochondrial changes | 2 | 1.415 | 0.121 | 0.958 | 0.725 | null | null |
| GO:0014889 | muscle atrophy | 2 | 1.643 | 0.317 | 0.903 | 0.928 | null | null |
| GO:0043501 | skeletal muscle adaptation | 2 | 1.643 | 0.630 | 0.897 | 0.994 | null | null |
| GO:0006954 | inflammatory response | 2 | 2.290 | 0.940 | 0.950 | 0.606 | 0.868 | 6.790 |
| GO:0006959 | humoral immune response | 2 | 1.839 | 0.329 | 0.947 | 0.609 | -0.977 | 4.835 |
| GO:0006984 | ER-nucleus signaling pathway | 2 | 1.079 | 0.053 | 0.923 | 0.215 | 1.410 | 7.119 |
| GO:0007029 | endoplasmic reticulum organization | 2 | 1.785 | 0.291 | 0.959 | 0.410 | -4.503 | 6.141 |
| GO:0007009 | plasma membrane organization | 2 | 1.929 | 0.407 | 0.959 | 0.746 | null | null |
| GO:0007033 | vacuole organization | 2 | 2.037 | 0.523 | 0.958 | 0.438 | -4.393 | 6.083 |
| GO:0007051 | spindle organization | 2 | 2.025 | 0.509 | 0.940 | 0.698 | -4.230 | 5.619 |
| GO:0001578 | microtubule bundle formation | 2 | 1.940 | 0.417 | 0.956 | 0.704 | null | null |
| GO:0002695 | negative regulation of leukocyte activation | 2 | 1.934 | 0.412 | 0.857 | 0.985 | null | null |
| GO:0022408 | negative regulation of cell-cell adhesion | 2 | 1.991 | 0.470 | 0.907 | 0.911 | null | null |
| GO:0050868 | negative regulation of T cell activation | 2 | 1.851 | 0.339 | 0.852 | 0.936 | null | null |
| GO:0051250 | negative regulation of lymphocyte activation | 2 | 1.881 | 0.363 | 0.859 | 0.968 | null | null |
| GO:1903038 | negative regulation of leukocyte cell-cell adhesion | 2 | 1.851 | 0.339 | 0.909 | 0.938 | null | null |
| GO:0007186 | G protein-coupled receptor signaling pathway | 2 | 3.084 | 5.876 | 0.880 | 0.445 | 0.146 | 5.676 |
| GO:0007187 | G protein-coupled receptor signaling pathway, coupled to cyclic nucleotide second messenger | 2 | 1.799 | 0.300 | 0.911 | 0.531 | 0.400 | 7.181 |
| GO:0007188 | adenylate cyclase-modulating G protein-coupled receptor signaling pathway | 2 | 2.476 | 1.444 | 0.897 | 0.308 | 0.210 | 5.982 |
| GO:0007193 | adenylate cyclase-inhibiting G protein-coupled receptor signaling pathway | 2 | 1.987 | 0.465 | 0.907 | 0.555 | 0.340 | 7.097 |
| GO:0007265 | Ras protein signal transduction | 2 | 1.813 | 0.310 | 0.909 | 0.663 | 0.768 | 5.871 |
| GO:0198738 | cell-cell signaling by wnt | 2 | 2.253 | 0.862 | 0.934 | 0.754 | null | null |
| GO:0007272 | ensheathment of neurons | 2 | 1.813 | 0.310 | 0.902 | 0.493 | -6.887 | 0.945 |
| GO:0045786 | negative regulation of cell cycle | 2 | 2.121 | 0.635 | 0.910 | 0.731 | null | null |
| GO:0007349 | cellularization | 2 | 2.246 | 0.536 | 0.903 | 0.594 | -5.951 | 0.040 |
| GO:0007350 | blastoderm segmentation | 2 | 1.204 | 0.073 | 0.920 | 0.659 | -7.504 | 1.113 |
| GO:0000578 | embryonic axis specification | 2 | 1.505 | 0.150 | 0.916 | 0.821 | null | null |
| GO:0016319 | mushroom body development | 2 | 1.851 | 0.300 | 0.900 | 0.739 | null | null |
| GO:0021536 | diencephalon development | 2 | 1.934 | 0.412 | 0.897 | 0.764 | null | null |
| GO:0048562 | embryonic organ morphogenesis | 2 | 2.648 | 2.151 | 0.874 | 0.736 | null | null |
| GO:0007549 | sex-chromosome dosage compensation | 2 | 1.146 | 0.067 | 0.903 | 0.504 | -3.126 | 4.402 |
| GO:0007619 | courtship behavior | 2 | 0.301 | 0.005 | 0.928 | 0.964 | null | null |
| GO:0008049 | male courtship behavior | 2 | 0.301 | 0.005 | 0.928 | 0.964 | null | null |
| GO:0060179 | male mating behavior | 2 | 0.301 | 0.005 | 0.928 | 0.964 | null | null |
| GO:0007612 | learning | 2 | 1.079 | 0.053 | 0.937 | 0.945 | null | null |
| GO:0007613 | memory | 2 | 1.146 | 0.063 | 0.936 | 0.956 | null | null |
| GO:0008306 | associative learning | 2 | 0.301 | 0.005 | 0.946 | 0.810 | null | null |
| GO:0030537 | larval behavior | 2 | 1.255 | 0.082 | 0.945 | 0.717 | null | null |
| GO:0007635 | chemosensory behavior | 2 | 1.000 | 0.044 | 0.899 | 0.681 | -2.461 | 4.384 |
| GO:0008038 | neuron recognition | 2 | 1.602 | 0.189 | 0.894 | 0.606 | -7.312 | 0.494 |
| GO:0050678 | regulation of epithelial cell proliferation | 2 | 1.613 | 0.194 | 0.954 | 0.727 | null | null |
| GO:0008544 | epidermis development | 2 | 2.100 | 0.606 | 0.927 | 0.578 | -6.979 | -1.770 |
| GO:0033865 | nucleoside bisphosphate metabolic process | 2 | 1.892 | 0.373 | 0.893 | 0.717 | null | null |
| GO:0033866 | nucleoside bisphosphate biosynthetic process | 2 | 1.602 | 0.189 | 0.889 | 0.939 | null | null |
| GO:0033875 | ribonucleoside bisphosphate metabolic process | 2 | 1.892 | 0.373 | 0.892 | 0.996 | null | null |
| GO:0034030 | ribonucleoside bisphosphate biosynthetic process | 2 | 1.602 | 0.189 | 0.889 | 0.998 | null | null |
| GO:0034032 | purine nucleoside bisphosphate metabolic process | 2 | 1.892 | 0.373 | 0.886 | 0.717 | null | null |
| GO:0034033 | purine nucleoside bisphosphate biosynthetic process | 2 | 1.602 | 0.189 | 0.882 | 0.939 | null | null |
| GO:0010212 | response to ionizing radiation | 2 | 1.519 | 0.155 | 0.956 | 0.825 | null | null |
| GO:0050906 | detection of stimulus involved in sensory perception | 2 | 2.314 | 0.993 | 0.894 | 0.848 | null | null |
| GO:0051602 | response to electrical stimulus | 2 | 1.748 | 0.262 | 0.955 | 0.728 | null | null |
| GO:0071479 | cellular response to ionizing radiation | 2 | 0.903 | 0.034 | 0.952 | 0.819 | null | null |
| GO:0009408 | response to heat | 2 | 1.544 | 0.165 | 0.948 | 0.861 | null | null |
| GO:0009411 | response to UV | 2 | 1.322 | 0.097 | 0.958 | 0.667 | 1.885 | 6.262 |
| GO:0009649 | entrainment of circadian clock | 2 | 1.255 | 0.082 | 0.925 | 0.545 | 1.866 | 6.017 |
| GO:0009743 | response to carbohydrate | 2 | 1.342 | 0.102 | 0.937 | 0.540 | -1.116 | 6.755 |
| GO:0009749 | response to glucose | 2 | 1.322 | 0.097 | 0.936 | 0.539 | -1.102 | 6.819 |
| GO:0009746 | response to hexose | 2 | 1.342 | 0.102 | 0.936 | 0.995 | null | null |
| GO:0034284 | response to monosaccharide | 2 | 1.342 | 0.102 | 0.936 | 0.999 | null | null |
| GO:0009914 | hormone transport | 2 | 1.279 | 0.087 | 0.914 | 0.433 | 3.843 | 3.407 |
| GO:0010001 | glial cell differentiation | 2 | 2.053 | 0.543 | 0.886 | 0.640 | -7.125 | 0.423 |
| GO:0008347 | glial cell migration | 2 | 0.954 | 0.039 | 0.905 | 0.775 | null | null |
| GO:0014037 | Schwann cell differentiation | 2 | 1.398 | 0.116 | 0.900 | 0.846 | null | null |
| GO:0021782 | glial cell development | 2 | 1.833 | 0.325 | 0.888 | 0.926 | null | null |
| GO:0071347 | cellular response to interleukin-1 | 2 | 1.544 | 0.165 | 0.927 | 0.787 | null | null |
| GO:0051592 | response to calcium ion | 2 | 1.690 | 0.233 | 0.939 | 0.892 | null | null |
| GO:0071241 | cellular response to inorganic substance | 2 | 1.716 | 0.247 | 0.928 | 0.896 | null | null |
| GO:0071248 | cellular response to metal ion | 2 | 1.708 | 0.242 | 0.928 | 0.895 | null | null |
| GO:0010226 | response to lithium ion | 2 | 0.477 | 0.010 | 0.952 | 0.697 | -1.610 | 6.936 |
| GO:0010324 | membrane invagination | 2 | 1.699 | 0.237 | 0.961 | 0.612 | -4.676 | 5.236 |
| GO:0010332 | response to gamma radiation | 2 | 0.477 | 0.010 | 0.964 | 0.699 | -2.203 | 7.358 |
| GO:0010591 | regulation of lamellipodium assembly | 2 | 0.903 | 0.034 | 0.925 | 0.694 | 1.754 | -2.149 |
| GO:0010631 | epithelial cell migration | 2 | 1.663 | 0.218 | 0.937 | 0.667 | -7.375 | 2.721 |
| GO:0007297 | follicle cell of egg chamber migration | 2 | 1.041 | 0.102 | 0.868 | 0.955 | null | null |
| GO:0007298 | border follicle cell migration | 2 | 1.041 | 0.102 | 0.868 | 0.926 | null | null |
| GO:0090132 | epithelium migration | 2 | 1.699 | 0.237 | 0.947 | 0.974 | null | null |
| GO:0010660 | regulation of muscle cell apoptotic process | 2 | 0.477 | 0.010 | 0.961 | 0.505 | -2.251 | -6.910 |
| GO:0010662 | regulation of striated muscle cell apoptotic process | 2 | 0.477 | 0.010 | 0.961 | 0.505 | -2.274 | -6.786 |
| GO:0010831 | positive regulation of myotube differentiation | 2 | 0.699 | 0.019 | 0.884 | 0.883 | null | null |
| GO:0048742 | regulation of skeletal muscle fiber development | 2 | 1.114 | 0.058 | 0.906 | 0.949 | null | null |
| GO:0051148 | negative regulation of muscle cell differentiation | 2 | 0.477 | 0.010 | 0.893 | 0.823 | null | null |
| GO:0051149 | positive regulation of muscle cell differentiation | 2 | 1.079 | 0.053 | 0.876 | 0.918 | null | null |
| GO:0051154 | negative regulation of striated muscle cell differentiation | 2 | 0.699 | 0.023 | 0.887 | 0.891 | null | null |
| GO:0051155 | positive regulation of striated muscle cell differentiation | 2 | 1.079 | 0.053 | 0.876 | 0.943 | null | null |
| GO:0010906 | regulation of glucose metabolic process | 2 | 1.230 | 0.078 | 0.936 | 0.254 | 3.820 | 1.104 |
| GO:0055007 | cardiac muscle cell differentiation | 2 | 1.756 | 0.271 | 0.877 | 0.933 | null | null |
| GO:0014812 | muscle cell migration | 2 | 1.114 | 0.058 | 0.989 | 0.586 | -6.751 | -5.145 |
| GO:0014854 | response to inactivity | 2 | 3.786 | 29.597 | 0.942 | 0.659 | 0.156 | 6.346 |
| GO:0014870 | response to muscle inactivity | 2 | 3.786 | 0.121 | 0.969 | 0.175 | 1.811 | 6.900 |
| GO:0014896 | muscle hypertrophy | 2 | 0.301 | 0.005 | 0.952 | 0.670 | -6.919 | 3.968 |
| GO:0014897 | striated muscle hypertrophy | 2 | 0.301 | 0.005 | 0.952 | 0.670 | -6.888 | 3.971 |
| GO:0015695 | organic cation transport | 2 | 1.462 | 0.136 | 0.956 | 0.327 | 5.137 | 4.384 |
| GO:0015698 | inorganic anion transport | 2 | 2.279 | 0.916 | 0.952 | 0.407 | 5.095 | 4.150 |
| GO:0016071 | mRNA metabolic process | 2 | 2.605 | 1.947 | 0.905 | 0.670 | 1.448 | -6.423 |
| GO:0045843 | negative regulation of striated muscle tissue development | 2 | 0.301 | 0.005 | 0.883 | 1.000 | null | null |
| GO:0048633 | positive regulation of skeletal muscle tissue growth | 2 | 0.602 | 0.033 | 0.881 | 1.005 | null | null |
| GO:0048635 | negative regulation of muscle organ development | 2 | 0.602 | 0.015 | 0.878 | 0.867 | null | null |
| GO:1901862 | negative regulation of muscle tissue development | 2 | 0.301 | 0.005 | 0.903 | 0.768 | null | null |
| GO:0001667 | ameboidal-type cell migration | 2 | 2.378 | 1.153 | 0.985 | 0.809 | null | null |
| GO:0016485 | protein processing | 2 | 2.100 | 0.606 | 0.910 | 0.455 | 0.915 | -6.374 |
| GO:0016579 | protein deubiquitination | 2 | 1.176 | 0.068 | 0.938 | 0.523 | 0.001 | -6.943 |
| GO:0017038 | protein import | 2 | 0.477 | 0.010 | 0.951 | 0.374 | 4.514 | 4.951 |
| GO:0018212 | peptidyl-tyrosine modification | 2 | 0.477 | 0.010 | 0.946 | 0.595 | -0.399 | -7.102 |
| GO:0018279 | protein N-linked glycosylation via asparagine | 2 | 1.000 | 0.044 | 0.913 | 0.549 | 0.316 | -6.503 |
| GO:0018410 | C-terminal protein amino acid modification | 2 | 0.477 | 0.010 | 0.946 | 0.440 | -0.353 | -7.180 |
| GO:0019722 | calcium-mediated signaling | 2 | 2.111 | 0.620 | 0.903 | 0.479 | 0.583 | 5.989 |
| GO:0007264 | small GTPase-mediated signal transduction | 2 | 2.346 | 1.071 | 0.897 | 0.748 | null | null |
| GO:0006633 | fatty acid biosynthetic process | 2 | 1.940 | 0.417 | 0.892 | 0.916 | null | null |
| GO:0009063 | amino acid catabolic process | 2 | 2.009 | 0.489 | 0.886 | 0.934 | null | null |
| GO:0043648 | dicarboxylic acid metabolic process | 2 | 1.732 | 0.257 | 0.917 | 0.733 | null | null |
| GO:1901606 | alpha-amino acid catabolic process | 2 | 1.949 | 0.426 | 0.887 | 0.922 | null | null |
| GO:0021545 | cranial nerve development | 2 | 1.792 | 0.295 | 0.907 | 0.491 | -7.062 | 0.880 |
| GO:0021602 | cranial nerve morphogenesis | 2 | 1.398 | 0.116 | 0.907 | 0.881 | null | null |
| GO:0021783 | preganglionic parasympathetic fiber development | 2 | 0.954 | 0.039 | 0.919 | 0.401 | -7.199 | 1.258 |
| GO:0021561 | facial nerve development | 2 | 0.778 | 0.024 | 0.921 | 0.956 | null | null |
| GO:0021610 | facial nerve morphogenesis | 2 | 0.602 | 0.015 | 0.917 | 0.927 | null | null |
| GO:0048486 | parasympathetic nervous system development | 2 | 1.000 | 0.044 | 0.920 | 0.761 | null | null |
| GO:0022406 | membrane docking | 2 | 1.813 | 0.310 | 0.992 | 0.015 | -3.756 | -7.317 |
| GO:0001893 | maternal placenta development | 2 | 1.699 | 0.208 | 0.881 | 1.589 | null | null |
| GO:0007286 | spermatid development | 2 | 1.613 | 0.194 | 0.877 | 0.853 | null | null |
| GO:0007308 | oocyte construction | 2 | 1.204 | 0.073 | 0.883 | 0.866 | null | null |
| GO:0007309 | oocyte axis specification | 2 | 1.204 | 0.073 | 0.874 | 0.857 | null | null |
| GO:0007530 | sex determination | 2 | 1.204 | 0.073 | 0.917 | 0.703 | null | null |
| GO:0007565 | female pregnancy | 2 | 0.477 | 1.831 | 0.897 | 1.954 | null | null |
| GO:0008584 | male gonad development | 2 | 0.954 | 0.039 | 0.894 | 0.918 | null | null |
| GO:0030706 | germarium-derived oocyte differentiation | 2 | 1.279 | 0.141 | 0.869 | 0.971 | null | null |
| GO:0046546 | development of primary male sexual characteristics | 2 | 1.041 | 0.048 | 0.901 | 0.961 | null | null |
| GO:0046661 | male sex differentiation | 2 | 1.114 | 0.058 | 0.900 | 0.886 | null | null |
| GO:0046697 | decidualization | 2 | 1.869 | 0.269 | 0.872 | 1.625 | null | null |
| GO:0060135 | maternal process involved in female pregnancy | 2 | 2.009 | 0.421 | 0.907 | 1.691 | null | null |
| GO:0061982 | meiosis I cell cycle process | 2 | 1.785 | 0.291 | 0.938 | 0.959 | null | null |
| GO:0140013 | meiotic nuclear division | 2 | 1.857 | 0.344 | 0.906 | 0.935 | null | null |
| GO:0008360 | regulation of cell shape | 2 | 1.954 | 0.431 | 0.892 | 0.733 | null | null |
| GO:0022617 | extracellular matrix disassembly | 2 | 0.301 | 0.005 | 0.974 | 0.623 | -5.573 | 5.794 |
| GO:0006122 | mitochondrial electron transport, ubiquinol to cytochrome c | 2 | 1.041 | 0.048 | 0.949 | 0.830 | null | null |
| GO:0042773 | ATP synthesis coupled electron transport | 2 | 1.806 | 0.305 | 0.941 | 0.977 | null | null |
| GO:0042775 | mitochondrial ATP synthesis coupled electron transport | 2 | 1.785 | 0.291 | 0.941 | 0.960 | null | null |
| GO:0030010 | establishment of cell polarity | 2 | 1.845 | 0.334 | 0.991 | 0.015 | -5.501 | -6.399 |
| GO:0090162 | establishment of epithelial cell polarity | 2 | 1.255 | 0.082 | 0.992 | 0.769 | null | null |
| GO:0007018 | microtubule-based movement | 2 | 2.425 | 1.284 | 0.988 | 0.825 | null | null |
| GO:0030048 | actin filament-based movement | 2 | 1.724 | 0.252 | 0.986 | 0.725 | null | null |
| GO:0030866 | cortical actin cytoskeleton organization | 2 | 1.681 | 0.228 | 0.956 | 0.717 | null | null |
| GO:0044837 | actomyosin contractile ring organization | 2 | 0.954 | 0.039 | 0.948 | 0.822 | null | null |
| GO:0030111 | regulation of Wnt signaling pathway | 2 | 2.316 | 0.998 | 0.917 | 0.518 | 6.514 | 0.565 |
| GO:0006023 | aminoglycan biosynthetic process | 2 | 1.633 | 0.203 | 0.905 | 0.922 | null | null |
| GO:0006024 | glycosaminoglycan biosynthetic process | 2 | 1.477 | 0.140 | 0.908 | 0.895 | null | null |
| GO:0030206 | chondroitin sulfate biosynthetic process | 2 | 1.176 | 0.068 | 0.907 | 0.993 | null | null |
| GO:0030207 | chondroitin sulfate catabolic process | 2 | 0.845 | 0.092 | 0.899 | 0.973 | null | null |
| GO:1903510 | mucopolysaccharide metabolic process | 2 | 1.653 | 0.213 | 0.924 | 0.926 | null | null |
| GO:0010594 | regulation of endothelial cell migration | 2 | 1.415 | 0.121 | 0.908 | 0.757 | null | null |
| GO:0010632 | regulation of epithelial cell migration | 2 | 1.477 | 0.140 | 0.906 | 0.767 | null | null |
| GO:0030336 | negative regulation of cell migration | 2 | 1.672 | 0.223 | 0.914 | 0.801 | null | null |
| GO:0040013 | negative regulation of locomotion | 2 | 1.908 | 0.388 | 0.914 | 0.831 | null | null |
| GO:2000146 | negative regulation of cell motility | 2 | 1.690 | 0.233 | 0.914 | 0.909 | null | null |
| GO:0016331 | morphogenesis of embryonic epithelium | 2 | 1.869 | 0.354 | 0.894 | 0.738 | null | null |
| GO:0072175 | epithelial tube formation | 2 | 1.839 | 0.329 | 0.895 | 0.733 | null | null |
| GO:0030865 | cortical cytoskeleton organization | 2 | 1.826 | 0.320 | 0.958 | 0.596 | -4.277 | 6.110 |
| GO:0031047 | regulatory ncRNA-mediated gene silencing | 2 | 1.851 | 0.339 | 0.888 | 0.654 | 3.946 | 0.105 |
| GO:0031098 | stress-activated protein kinase signaling cascade | 2 | 0.301 | 0.005 | 0.926 | 0.365 | 1.986 | 6.807 |
| GO:0031099 | regeneration | 2 | 2.299 | 0.959 | 0.934 | 0.272 | -7.250 | -1.526 |
| GO:0045019 | negative regulation of nitric oxide biosynthetic process | 2 | 2.243 | 0.744 | 0.876 | 0.719 | null | null |
| GO:0045428 | regulation of nitric oxide biosynthetic process | 2 | 0.602 | 0.015 | 0.937 | 1.286 | null | null |
| GO:1904406 | negative regulation of nitric oxide metabolic process | 2 | 1.653 | 0.645 | 0.882 | 1.777 | null | null |
| GO:0016242 | negative regulation of macroautophagy | 2 | 0.903 | 0.034 | 0.899 | 0.799 | null | null |
| GO:0042177 | negative regulation of protein catabolic process | 2 | 1.380 | 0.111 | 0.878 | 0.875 | null | null |
| GO:0031349 | positive regulation of defense response | 2 | 1.908 | 0.388 | 0.892 | 0.837 | null | null |
| GO:0050778 | positive regulation of immune response | 2 | 2.350 | 1.080 | 0.862 | 0.855 | null | null |
| GO:1903320 | regulation of protein modification by small protein conjugation or removal | 2 | 1.643 | 0.208 | 0.909 | 0.747 | null | null |
| GO:1903321 | negative regulation of protein modification by small protein conjugation or removal | 2 | 1.146 | 0.063 | 0.887 | 0.910 | null | null |
| GO:0031623 | receptor internalization | 2 | 1.519 | 0.155 | 0.948 | 0.685 | 4.853 | 3.669 |
| GO:0009267 | cellular response to starvation | 2 | 1.740 | 0.262 | 0.931 | 0.985 | null | null |
| GO:0033273 | response to vitamin | 2 | 0.954 | 0.039 | 0.938 | 0.914 | null | null |
| GO:0032465 | regulation of cytokinesis | 2 | 1.477 | 0.140 | 0.954 | 0.625 | -2.890 | -6.977 |
| GO:0032471 | negative regulation of endoplasmic reticulum calcium ion concentration | 2 | 0.301 | 0.005 | 0.981 | 0.838 | null | null |
| GO:0002576 | platelet degranulation | 2 | 2.083 | 0.554 | 0.929 | 1.020 | null | null |
| GO:0035010 | encapsulation of foreign target | 2 | 1.462 | 0.147 | 0.978 | 0.898 | null | null |
| GO:0033292 | T-tubule organization | 2 | 0.903 | 0.034 | 0.903 | 0.680 | -5.201 | 2.345 |
| GO:0033500 | carbohydrate homeostasis | 2 | 1.748 | 0.266 | 0.977 | 0.660 | -3.850 | -4.898 |
| GO:0034330 | cell junction organization | 2 | 2.531 | 1.642 | 0.960 | 0.313 | -4.897 | 5.856 |
| GO:0034333 | adherens junction assembly | 2 | 0.477 | 0.010 | 0.969 | 0.580 | -5.490 | 5.546 |
| GO:0034504 | protein localization to nucleus | 2 | 1.863 | 0.349 | 0.943 | 0.523 | 4.739 | 3.762 |
| GO:0006974 | DNA damage response | 2 | 2.652 | 2.170 | 0.934 | 0.710 | null | null |
| GO:0035051 | cardiocyte differentiation | 2 | 1.881 | 0.363 | 0.889 | 0.626 | -7.129 | -0.094 |
| GO:0035148 | tube formation | 2 | 1.886 | 0.368 | 0.904 | 0.685 | -5.854 | 0.001 |
| GO:0035270 | endocrine system development | 2 | 2.086 | 0.586 | 0.911 | 0.347 | -6.487 | 0.862 |
| GO:0035283 | central nervous system segmentation | 2 | 0.845 | 0.029 | 0.919 | 0.622 | -7.378 | 1.194 |
| GO:0035284 | brain segmentation | 2 | 0.845 | 0.029 | 0.913 | 0.576 | -7.413 | 0.850 |
| GO:0036498 | IRE1-mediated unfolded protein response | 2 | 0.778 | 0.024 | 0.893 | 0.817 | null | null |
| GO:0051697 | protein delipidation | 2 | 3.009 | 4.941 | 0.905 | 0.896 | null | null |
| GO:0040007 | growth | 2 | 2.502 | 1.536 | 1.000 | 0.000 | -4.519 | -6.672 |
| GO:0040018 | positive regulation of multicellular organism growth | 2 | 0.301 | 0.005 | 0.880 | 0.424 | 5.471 | -2.894 |
| GO:0042157 | lipoprotein metabolic process | 2 | 1.869 | 0.354 | 0.934 | 0.427 | 0.380 | -6.717 |
| GO:0050731 | positive regulation of peptidyl-tyrosine phosphorylation | 2 | 1.531 | 0.160 | 0.864 | 0.833 | null | null |
| GO:0042398 | cellular modified amino acid biosynthetic process | 2 | 1.477 | 0.140 | 0.922 | 0.314 | 0.637 | -6.141 |
| GO:0042219 | cellular modified amino acid catabolic process | 2 | 1.415 | 0.121 | 0.922 | 0.778 | null | null |
| GO:0042461 | photoreceptor cell development | 2 | 1.940 | 0.417 | 0.884 | 0.661 | -7.190 | 0.396 |
| GO:0001754 | eye photoreceptor cell differentiation | 2 | 1.875 | 0.358 | 0.869 | 0.934 | null | null |
| GO:0042462 | eye photoreceptor cell development | 2 | 1.716 | 0.247 | 0.871 | 0.904 | null | null |
| GO:0042475 | odontogenesis of dentin-containing tooth | 2 | 1.000 | 0.044 | 0.929 | 0.425 | -5.741 | -1.620 |
| GO:0042476 | odontogenesis | 2 | 1.447 | 0.131 | 0.923 | 0.384 | -5.847 | -1.348 |
| GO:0042552 | myelination | 2 | 1.813 | 0.310 | 0.901 | 0.493 | -6.812 | 0.959 |
| GO:0008366 | axon ensheathment | 2 | 1.813 | 0.310 | 0.901 | 0.997 | null | null |
| GO:0042593 | glucose homeostasis | 2 | 1.740 | 0.262 | 0.977 | 0.659 | -3.836 | -4.837 |
| GO:0042908 | xenobiotic transport | 2 | 1.079 | 0.053 | 0.963 | 0.298 | 5.352 | 3.626 |
| GO:1904748 | regulation of apoptotic process involved in development | 2 | 1.892 | 0.410 | 0.899 | 0.720 | null | null |
| GO:0048701 | embryonic cranial skeleton morphogenesis | 2 | 2.217 | 0.794 | 0.881 | 0.840 | null | null |
| GO:0048704 | embryonic skeletal system morphogenesis | 2 | 2.258 | 0.872 | 0.880 | 0.963 | null | null |
| GO:0048706 | embryonic skeletal system development | 2 | 2.294 | 0.949 | 0.897 | 0.858 | null | null |
| GO:0043094 | cellular metabolic compound salvage | 2 | 1.568 | 0.174 | 0.950 | 0.142 | 3.679 | -5.783 |
| GO:0043101 | purine-containing compound salvage | 2 | 1.301 | 0.092 | 0.910 | 0.652 | 0.784 | -6.055 |
| GO:0043122 | regulation of canonical NF-kappaB signal transduction | 2 | 1.914 | 0.392 | 0.922 | 0.654 | 6.590 | 0.471 |
| GO:0043244 | regulation of protein-containing complex disassembly | 2 | 1.914 | 0.392 | 0.918 | 0.602 | 1.557 | -2.111 |
| GO:0051056 | regulation of small GTPase mediated signal transduction | 2 | 2.248 | 0.853 | 0.916 | 0.710 | null | null |
| GO:0043484 | regulation of RNA splicing | 2 | 2.097 | 0.601 | 0.918 | 0.320 | 3.301 | 0.628 |
| GO:0043523 | regulation of neuron apoptotic process | 2 | 1.771 | 0.281 | 0.949 | 0.691 | -2.278 | -6.660 |
| GO:0043524 | negative regulation of neuron apoptotic process | 2 | 1.431 | 0.126 | 0.920 | 0.635 | 3.550 | -2.376 |
| GO:0043588 | skin development | 2 | 1.643 | 0.208 | 0.933 | 0.390 | -6.277 | -1.799 |
| GO:0031116 | positive regulation of microtubule polymerization | 2 | 1.146 | 0.063 | 0.883 | 0.898 | null | null |
| GO:0031334 | positive regulation of protein-containing complex assembly | 2 | 1.672 | 0.223 | 0.877 | 0.869 | null | null |
| GO:0032273 | positive regulation of protein polymerization | 2 | 1.633 | 0.203 | 0.878 | 0.862 | null | null |
| GO:0019432 | triglyceride biosynthetic process | 2 | 1.301 | 0.092 | 0.923 | 0.904 | null | null |
| GO:0046460 | neutral lipid biosynthetic process | 2 | 1.301 | 0.092 | 0.924 | 0.904 | null | null |
| GO:0046463 | acylglycerol biosynthetic process | 2 | 1.301 | 0.092 | 0.923 | 0.999 | null | null |
| GO:0046470 | phosphatidylcholine metabolic process | 2 | 1.447 | 0.131 | 0.906 | 0.733 | null | null |
| GO:0046503 | glycerolipid catabolic process | 2 | 1.613 | 0.194 | 0.914 | 0.810 | null | null |
| GO:0044273 | sulfur compound catabolic process | 2 | 1.279 | 0.087 | 0.932 | 0.728 | null | null |
| GO:0030098 | lymphocyte differentiation | 2 | 1.799 | 0.300 | 0.884 | 0.918 | null | null |
| GO:0030217 | T cell differentiation | 2 | 1.653 | 0.213 | 0.887 | 0.958 | null | null |
| GO:0042110 | T cell activation | 2 | 1.771 | 0.281 | 0.921 | 0.872 | null | null |
| GO:0046649 | lymphocyte activation | 2 | 1.991 | 0.470 | 0.918 | 0.915 | null | null |
| GO:0045444 | fat cell differentiation | 2 | 1.176 | 0.068 | 0.934 | 0.195 | -7.565 | -2.168 |
| GO:0045778 | positive regulation of ossification | 2 | 0.699 | 0.019 | 0.896 | 0.469 | 4.803 | -2.868 |
| GO:0030500 | regulation of bone mineralization | 2 | 1.342 | 0.102 | 0.890 | 0.864 | null | null |
| GO:0050766 | positive regulation of phagocytosis | 2 | 0.699 | 0.019 | 0.873 | 0.735 | null | null |
| GO:0032435 | negative regulation of proteasomal ubiquitin-dependent protein catabolic process | 2 | 1.000 | 0.044 | 0.883 | 0.900 | null | null |
| GO:1901799 | negative regulation of proteasomal protein catabolic process | 2 | 1.079 | 0.053 | 0.882 | 0.982 | null | null |
| GO:1903051 | negative regulation of proteolysis involved in protein catabolic process | 2 | 1.114 | 0.058 | 0.886 | 0.788 | null | null |
| GO:2000059 | negative regulation of ubiquitin-dependent protein catabolic process | 2 | 1.041 | 0.048 | 0.882 | 0.969 | null | null |
| GO:0045931 | positive regulation of mitotic cell cycle | 2 | 1.415 | 0.121 | 0.916 | 0.616 | 4.399 | -2.129 |
| GO:0046328 | regulation of JNK cascade | 2 | 1.531 | 0.160 | 0.927 | 0.503 | 6.634 | 0.482 |
| GO:0046425 | regulation of receptor signaling pathway via JAK-STAT | 2 | 1.342 | 0.102 | 0.932 | 0.403 | 6.601 | 0.364 |
| GO:0046530 | photoreceptor cell differentiation | 2 | 2.053 | 0.543 | 0.886 | 0.682 | -7.100 | 0.479 |
| GO:0046621 | negative regulation of organ growth | 2 | 0.477 | 0.010 | 0.878 | 0.676 | 5.586 | -2.875 |
| GO:0006401 | RNA catabolic process | 2 | 2.009 | 0.489 | 0.899 | 0.881 | null | null |
| GO:0046777 | protein autophosphorylation | 2 | 0.699 | 0.019 | 0.928 | 0.466 | 0.163 | -7.039 |
| GO:0018105 | peptidyl-serine phosphorylation | 2 | 0.602 | 0.024 | 0.926 | 0.794 | null | null |
| GO:0018108 | peptidyl-tyrosine phosphorylation | 2 | 0.477 | 0.010 | 0.931 | 0.754 | null | null |
| GO:0006612 | protein targeting to membrane | 2 | 1.785 | 0.291 | 0.940 | 0.801 | null | null |
| GO:0016197 | endosomal transport | 2 | 2.276 | 0.911 | 0.931 | 0.704 | null | null |
| GO:0032456 | endocytic recycling | 2 | 1.785 | 0.291 | 0.937 | 0.801 | null | null |
| GO:0072594 | establishment of protein localization to organelle | 2 | 2.336 | 1.046 | 0.934 | 0.784 | null | null |
| GO:0002474 | antigen processing and presentation of peptide antigen via MHC class I | 2 | 1.041 | 0.048 | 0.980 | 0.887 | null | null |
| GO:0002495 | antigen processing and presentation of peptide antigen via MHC class II | 2 | 1.398 | 0.116 | 0.978 | 0.966 | null | null |
| GO:0002504 | antigen processing and presentation of peptide or polysaccharide antigen via MHC class II | 2 | 1.415 | 0.121 | 0.978 | 0.949 | null | null |
| GO:0019886 | antigen processing and presentation of exogenous peptide antigen via MHC class II | 2 | 1.362 | 0.107 | 0.978 | 0.992 | null | null |
| GO:0048194 | Golgi vesicle budding | 2 | 0.845 | 0.029 | 0.925 | 0.636 | 2.647 | 4.378 |
| GO:0048284 | organelle fusion | 2 | 2.064 | 0.557 | 0.958 | 0.441 | -4.298 | 6.016 |
| GO:0016322 | neuron remodeling | 2 | 0.602 | 0.015 | 0.910 | 0.746 | null | null |
| GO:0048483 | autonomic nervous system development | 2 | 1.740 | 0.262 | 0.910 | 0.484 | -6.981 | 0.973 |
| GO:0048538 | thymus development | 2 | 1.342 | 0.102 | 0.902 | 0.912 | null | null |
| GO:0048589 | developmental growth | 2 | 2.502 | 1.536 | 0.934 | 0.277 | -6.647 | -1.657 |
| GO:0040014 | regulation of multicellular organism growth | 2 | 0.778 | 0.024 | 0.898 | 0.721 | null | null |
| GO:0046620 | regulation of organ growth | 2 | 1.176 | 0.068 | 0.891 | 0.781 | null | null |
| GO:0048631 | regulation of skeletal muscle tissue growth | 2 | 1.146 | 0.128 | 0.909 | 0.823 | null | null |
| GO:0048640 | negative regulation of developmental growth | 2 | 1.681 | 0.228 | 0.872 | 0.971 | null | null |
| GO:0048736 | appendage development | 2 | 2.083 | 0.581 | 0.915 | 0.256 | -6.040 | 0.569 |
| GO:0048737 | imaginal disc-derived appendage development | 2 | 1.908 | 0.388 | 0.915 | 0.244 | -6.040 | 0.764 |
| GO:0035107 | appendage morphogenesis | 2 | 1.845 | 0.334 | 0.906 | 0.909 | null | null |
| GO:0035114 | imaginal disc-derived appendage morphogenesis | 2 | 1.342 | 0.104 | 0.915 | 0.824 | null | null |
| GO:0048754 | branching morphogenesis of an epithelial tube | 2 | 1.301 | 0.092 | 0.907 | 0.594 | -5.850 | -0.497 |
| GO:0061138 | morphogenesis of a branching epithelium | 2 | 1.362 | 0.107 | 0.911 | 0.953 | null | null |
| GO:0060249 | anatomical structure homeostasis | 2 | 1.949 | 0.426 | 0.927 | 0.834 | null | null |
| GO:0050655 | dermatan sulfate proteoglycan metabolic process | 2 | 0.699 | 0.019 | 0.934 | 0.513 | -0.315 | -6.726 |
| GO:0030166 | proteoglycan biosynthetic process | 2 | 1.763 | 0.276 | 0.900 | 0.909 | null | null |
| GO:0030204 | chondroitin sulfate metabolic process | 2 | 1.255 | 0.082 | 0.915 | 0.956 | null | null |
| GO:0030205 | dermatan sulfate metabolic process | 2 | 0.602 | 0.015 | 0.906 | 1.090 | null | null |
| GO:0030208 | dermatan sulfate biosynthetic process | 2 | 1.146 | 0.116 | 0.883 | 1.257 | null | null |
| GO:0050650 | chondroitin sulfate proteoglycan biosynthetic process | 2 | 1.362 | 0.107 | 0.907 | 0.859 | null | null |
| GO:0050651 | dermatan sulfate proteoglycan biosynthetic process | 2 | 1.362 | 0.107 | 0.907 | 0.845 | null | null |
| GO:0050654 | chondroitin sulfate proteoglycan metabolic process | 2 | 1.431 | 0.126 | 0.924 | 0.750 | null | null |
| GO:0050658 | RNA transport | 2 | 1.934 | 0.412 | 0.945 | 0.517 | 4.784 | 4.597 |
| GO:0050657 | nucleic acid transport | 2 | 1.934 | 0.412 | 0.950 | 0.900 | null | null |
| GO:0051236 | establishment of RNA localization | 2 | 1.934 | 0.412 | 0.952 | 0.969 | null | null |
| GO:0048134 | germ-line cyst formation | 2 | 1.146 | 0.073 | 0.885 | 0.711 | null | null |
| GO:0048024 | regulation of mRNA splicing, via spliceosome | 2 | 1.903 | 0.383 | 0.920 | 0.849 | null | null |
| GO:0002696 | positive regulation of leukocyte activation | 2 | 2.021 | 0.504 | 0.850 | 0.988 | null | null |
| GO:0022409 | positive regulation of cell-cell adhesion | 2 | 1.959 | 0.436 | 0.901 | 0.950 | null | null |
| GO:0050866 | negative regulation of cell activation | 2 | 1.934 | 0.412 | 0.866 | 0.924 | null | null |
| GO:0050867 | positive regulation of cell activation | 2 | 2.021 | 0.504 | 0.858 | 0.942 | null | null |
| GO:0050870 | positive regulation of T cell activation | 2 | 1.919 | 0.397 | 0.846 | 0.921 | null | null |
| GO:0051251 | positive regulation of lymphocyte activation | 2 | 1.991 | 0.470 | 0.851 | 0.967 | null | null |
| GO:1903039 | positive regulation of leukocyte cell-cell adhesion | 2 | 1.919 | 0.397 | 0.901 | 0.979 | null | null |
| GO:0010469 | regulation of signaling receptor activity | 2 | 0.477 | 0.010 | 0.911 | 0.762 | null | null |
| GO:0032147 | activation of protein kinase activity | 2 | 0.477 | 0.023 | 0.854 | 1.026 | null | null |
| GO:0043087 | regulation of GTPase activity | 2 | 1.114 | 0.058 | 0.929 | 0.892 | null | null |
| GO:0043406 | positive regulation of MAP kinase activity | 2 | 0.477 | 0.010 | 0.840 | 0.977 | null | null |
| GO:0043547 | positive regulation of GTPase activity | 2 | 0.778 | 0.024 | 0.931 | 0.875 | null | null |
| GO:0051098 | regulation of binding | 2 | 0.477 | 0.010 | 0.938 | 0.762 | null | null |
| GO:0051099 | positive regulation of binding | 2 | 0.477 | 0.010 | 0.936 | 0.762 | null | null |
| GO:0051346 | negative regulation of hydrolase activity | 2 | 0.845 | 0.029 | 0.931 | 0.913 | null | null |
| GO:0052548 | regulation of endopeptidase activity | 2 | 0.477 | 0.010 | 0.898 | 0.921 | null | null |
| GO:0071901 | negative regulation of protein serine/threonine kinase activity | 2 | 0.301 | 0.005 | 0.870 | 0.883 | null | null |
| GO:0050795 | regulation of behavior | 2 | 1.491 | 0.145 | 0.916 | 0.561 | 5.213 | -4.311 |
| GO:0048167 | regulation of synaptic plasticity | 2 | 1.748 | 0.266 | 0.924 | 0.881 | null | null |
| GO:0048168 | regulation of neuronal synaptic plasticity | 2 | 1.322 | 0.097 | 0.930 | 0.805 | null | null |
| GO:0050808 | synapse organization | 2 | 2.279 | 0.916 | 0.959 | 0.291 | -5.115 | 5.584 |
| GO:0007416 | synapse assembly | 2 | 1.799 | 0.300 | 0.875 | 0.770 | null | null |
| GO:0034329 | cell junction assembly | 2 | 2.288 | 0.935 | 0.955 | 0.863 | null | null |
| GO:0034332 | adherens junction organization | 2 | 1.792 | 0.295 | 0.963 | 0.769 | null | null |
| GO:0045216 | cell-cell junction organization | 2 | 2.196 | 0.756 | 0.960 | 0.844 | null | null |
| GO:0050821 | protein stabilization | 2 | 1.431 | 0.126 | 0.950 | 0.448 | -1.581 | -2.336 |
| GO:0031648 | protein destabilization | 2 | 0.477 | 0.010 | 0.959 | 0.780 | null | null |
| GO:0050954 | sensory perception of mechanical stimulus | 2 | 1.881 | 0.363 | 0.937 | 0.527 | -6.914 | 3.097 |
| GO:0007601 | visual perception | 2 | 2.253 | 0.862 | 0.932 | 0.721 | null | null |
| GO:0050953 | sensory perception of light stimulus | 2 | 2.260 | 0.877 | 0.931 | 0.788 | null | null |
| GO:0050982 | detection of mechanical stimulus | 2 | 1.447 | 0.131 | 0.947 | 0.684 | 1.606 | 6.069 |
| GO:0032386 | regulation of intracellular transport | 2 | 1.643 | 0.208 | 0.918 | 0.865 | null | null |
| GO:0050796 | regulation of insulin secretion | 2 | 1.279 | 0.087 | 0.893 | 0.972 | null | null |
| GO:0090276 | regulation of peptide hormone secretion | 2 | 1.431 | 0.126 | 0.892 | 0.999 | null | null |
| GO:0090280 | positive regulation of calcium ion import | 2 | 0.602 | 0.015 | 0.900 | 0.904 | null | null |
| GO:0031111 | negative regulation of microtubule polymerization or depolymerization | 2 | 1.041 | 0.048 | 0.895 | 0.809 | null | null |
| GO:0031112 | positive regulation of microtubule polymerization or depolymerization | 2 | 1.176 | 0.068 | 0.887 | 0.827 | null | null |
| GO:0051169 | nuclear transport | 2 | 2.111 | 0.620 | 0.938 | 0.671 | 4.418 | 4.312 |
| GO:0051403 | stress-activated MAPK cascade | 2 | 0.301 | 0.005 | 0.926 | 0.365 | 1.996 | 6.741 |
| GO:0051451 | myoblast migration | 2 | 0.778 | 0.024 | 0.989 | 0.542 | -6.685 | -5.137 |
| GO:0051560 | mitochondrial calcium ion homeostasis | 2 | 1.041 | 0.048 | 0.978 | 0.647 | -3.736 | -4.893 |
| GO:0051561 | positive regulation of mitochondrial calcium ion concentration | 2 | 0.602 | 0.015 | 0.980 | 0.601 | -3.573 | -4.852 |
| GO:0051604 | protein maturation | 2 | 2.531 | 1.642 | 0.902 | 0.519 | 1.078 | -6.342 |
| GO:0032507 | maintenance of protein location in cell | 2 | 1.380 | 0.111 | 0.946 | 0.869 | null | null |
| GO:0045185 | maintenance of protein location | 2 | 1.462 | 0.136 | 0.945 | 0.882 | null | null |
| GO:0051657 | maintenance of organelle location | 2 | 0.778 | 0.024 | 0.950 | 0.778 | null | null |
| GO:0051659 | maintenance of mitochondrion location | 2 | 0.301 | 0.005 | 0.956 | 0.700 | null | null |
| GO:0006903 | vesicle targeting | 2 | 1.301 | 0.092 | 0.940 | 0.711 | null | null |
| GO:0048489 | synaptic vesicle transport | 2 | 1.398 | 0.116 | 0.942 | 1.002 | null | null |
| GO:0097479 | synaptic vesicle localization | 2 | 1.415 | 0.121 | 0.958 | 0.852 | null | null |
| GO:0097480 | establishment of synaptic vesicle localization | 2 | 1.462 | 0.131 | 0.942 | 0.858 | null | null |
| GO:0140056 | organelle localization by membrane tethering | 2 | 1.813 | 0.310 | 0.948 | 0.796 | null | null |
| GO:0002237 | response to molecule of bacterial origin | 2 | 1.982 | 0.460 | 0.921 | 0.818 | null | null |
| GO:0019730 | antimicrobial humoral response | 2 | 1.531 | 0.160 | 0.931 | 0.714 | null | null |
| GO:0051881 | regulation of mitochondrial membrane potential | 2 | 0.699 | 0.019 | 0.956 | 0.558 | -1.217 | -2.499 |
| GO:0051897 | positive regulation of phosphatidylinositol 3-kinase/protein kinase B signal transduction | 2 | 1.079 | 0.053 | 0.899 | 0.463 | 6.246 | -0.239 |
| GO:0055081 | monoatomic anion homeostasis | 2 | 1.415 | 0.121 | 0.977 | 0.610 | -3.624 | -4.752 |
| GO:0055123 | digestive system development | 2 | 2.176 | 0.722 | 0.909 | 0.356 | -6.487 | 0.764 |
| GO:0051216 | cartilage development | 2 | 2.210 | 0.780 | 0.888 | 0.832 | null | null |
| GO:0060350 | endochondral bone morphogenesis | 2 | 1.342 | 0.102 | 0.903 | 0.878 | null | null |
| GO:0060351 | cartilage development involved in endochondral bone morphogenesis | 2 | 1.176 | 0.068 | 0.900 | 0.852 | null | null |
| GO:0060485 | mesenchyme development | 2 | 2.389 | 1.182 | 0.912 | 0.630 | -6.776 | -1.303 |
| GO:0060541 | respiratory system development | 2 | 1.301 | 0.092 | 0.924 | 0.209 | -6.647 | 1.292 |
| GO:0060586 | multicellular organismal-level iron ion homeostasis | 2 | 0.699 | 0.019 | 0.939 | 0.520 | -4.242 | -3.067 |
| GO:0060612 | adipose tissue development | 2 | 0.903 | 0.034 | 0.935 | 0.427 | -6.854 | -2.279 |
| GO:0060688 | regulation of morphogenesis of a branching structure | 2 | 0.699 | 0.019 | 0.904 | 0.472 | 5.677 | -3.640 |
| GO:0060788 | ectodermal placode formation | 2 | 1.491 | 0.145 | 0.931 | 0.388 | -5.527 | -1.297 |
| GO:0071697 | ectodermal placode morphogenesis | 2 | 1.556 | 0.170 | 0.932 | 0.954 | null | null |
| GO:0060828 | regulation of canonical Wnt signaling pathway | 2 | 2.158 | 0.693 | 0.919 | 0.523 | 6.542 | 0.673 |
| GO:0030178 | negative regulation of Wnt signaling pathway | 2 | 2.033 | 0.518 | 0.887 | 0.891 | null | null |
| GO:0090090 | negative regulation of canonical Wnt signaling pathway | 2 | 1.857 | 0.344 | 0.891 | 0.857 | null | null |
| GO:0061008 | hepaticobiliary system development | 2 | 2.223 | 0.804 | 0.908 | 0.360 | -6.388 | 0.694 |
| GO:0061025 | membrane fusion | 2 | 2.057 | 0.547 | 0.958 | 0.658 | -4.220 | 5.419 |
| GO:0061053 | somite development | 2 | 2.176 | 0.722 | 0.897 | 0.594 | -6.648 | -0.242 |
| GO:0061383 | trabecula morphogenesis | 2 | 1.079 | 0.053 | 0.939 | 0.352 | -5.447 | -1.690 |
| GO:0061384 | heart trabecula morphogenesis | 2 | 1.079 | 0.053 | 0.939 | 0.352 | -5.692 | -1.961 |
| GO:0000281 | mitotic cytokinesis | 2 | 1.756 | 0.271 | 0.972 | 0.948 | null | null |
| GO:1902410 | mitotic cytokinetic process | 2 | 1.000 | 0.044 | 0.975 | 0.816 | null | null |
| GO:0061919 | process utilizing autophagic mechanism | 2 | 2.204 | 0.770 | 0.991 | 0.017 | -2.320 | -4.779 |
| GO:0062012 | regulation of small molecule metabolic process | 2 | 1.778 | 0.286 | 0.930 | 0.265 | 2.669 | 0.860 |
| GO:0070085 | glycosylation | 2 | 2.369 | 1.129 | 0.957 | 0.178 | 3.395 | -5.363 |
| GO:0070167 | regulation of biomineral tissue development | 2 | 1.342 | 0.102 | 0.890 | 0.546 | 5.644 | -3.558 |
| GO:0070252 | actin-mediated cell contraction | 2 | 1.447 | 0.131 | 0.987 | 0.678 | -2.025 | 2.935 |
| GO:0070646 | protein modification by small protein removal | 2 | 1.491 | 0.145 | 0.933 | 0.565 | 0.168 | -6.859 |
| GO:0032496 | response to lipopolysaccharide | 2 | 1.973 | 0.450 | 0.915 | 0.876 | null | null |
| GO:0014074 | response to purine-containing compound | 2 | 1.114 | 0.058 | 0.937 | 0.760 | null | null |
| GO:0032355 | response to estradiol | 2 | 1.415 | 0.121 | 0.933 | 0.807 | null | null |
| GO:0071383 | cellular response to steroid hormone stimulus | 2 | 1.613 | 0.194 | 0.918 | 0.870 | null | null |
| GO:0071384 | cellular response to corticosteroid stimulus | 2 | 1.146 | 0.063 | 0.925 | 0.989 | null | null |
| GO:0071385 | cellular response to glucocorticoid stimulus | 2 | 1.146 | 0.063 | 0.925 | 0.989 | null | null |
| GO:0071480 | cellular response to gamma radiation | 2 | 0.477 | 0.010 | 0.956 | 0.557 | -0.477 | 4.390 |
| GO:0071696 | ectodermal placode development | 2 | 1.643 | 0.208 | 0.943 | 0.227 | -6.666 | -2.339 |
| GO:0072347 | response to anesthetic | 2 | 2.507 | 1.554 | 0.930 | 0.660 | -0.573 | 6.577 |
| GO:0072348 | sulfur compound transport | 2 | 1.568 | 0.174 | 0.959 | 0.335 | 4.234 | 4.645 |
| GO:0090076 | relaxation of skeletal muscle | 2 | 0.301 | 0.005 | 0.952 | 0.670 | -6.946 | 3.951 |
| GO:0090130 | tissue migration | 2 | 1.740 | 0.262 | 0.950 | 0.191 | -7.274 | 2.960 |
| GO:0002027 | regulation of heart rate | 2 | 1.505 | 0.150 | 0.893 | 0.868 | null | null |
| GO:0010611 | regulation of cardiac muscle hypertrophy | 2 | 0.477 | 0.010 | 0.901 | 0.976 | null | null |
| GO:0010614 | negative regulation of cardiac muscle hypertrophy | 2 | 0.477 | 0.074 | 0.860 | 1.277 | null | null |
| GO:0014733 | regulation of skeletal muscle adaptation | 2 | 0.301 | 0.005 | 0.906 | 0.925 | null | null |
| GO:0014741 | negative regulation of muscle hypertrophy | 2 | 0.903 | 0.040 | 0.882 | 0.867 | null | null |
| GO:0014743 | regulation of muscle hypertrophy | 2 | 0.778 | 0.024 | 0.914 | 0.836 | null | null |
| GO:0014874 | response to stimulus involved in regulation of muscle adaptation | 2 | 0.699 | 14.801 | 0.771 | 2.115 | null | null |
| GO:0014877 | response to muscle inactivity involved in regulation of muscle adaptation | 2 | 0.699 | 0.208 | 0.849 | 1.161 | null | null |
| GO:0014883 | transition between fast and slow fiber | 2 | 0.301 | 0.005 | 0.906 | 0.925 | null | null |
| GO:0014894 | response to denervation involved in regulation of muscle adaptation | 2 | 0.699 | 0.019 | 0.873 | 0.824 | null | null |
| GO:0045932 | negative regulation of muscle contraction | 2 | 0.699 | 0.019 | 0.888 | 0.824 | null | null |
| GO:0045988 | negative regulation of striated muscle contraction | 2 | 0.845 | 0.044 | 0.880 | 0.953 | null | null |
| GO:0098909 | regulation of cardiac muscle cell action potential involved in regulation of contraction | 2 | 0.477 | 1.261 | 0.850 | 1.298 | null | null |
| GO:0090287 | regulation of cellular response to growth factor stimulus | 2 | 2.225 | 0.809 | 0.930 | 0.463 | 6.862 | 0.159 |
| GO:0090559 | regulation of membrane permeability | 2 | 1.279 | 0.087 | 0.952 | 0.433 | -1.505 | -2.178 |
| GO:0097066 | response to thyroid hormone | 2 | 0.699 | 0.019 | 0.942 | 0.432 | -1.436 | 6.403 |
| GO:0098609 | cell-cell adhesion | 2 | 2.681 | 2.320 | 0.990 | 0.020 | -1.619 | 1.037 |
| GO:0098754 | detoxification | 2 | 1.982 | 0.460 | 0.937 | 0.565 | -0.870 | 6.450 |
| GO:0097237 | cellular response to toxic substance | 2 | 1.914 | 0.392 | 0.927 | 0.962 | null | null |
| GO:0098900 | regulation of action potential | 2 | 0.954 | 0.039 | 0.951 | 0.584 | -1.233 | -2.380 |
| GO:0098901 | regulation of cardiac muscle cell action potential | 2 | 0.477 | 0.010 | 0.955 | 0.534 | -1.203 | -2.244 |
| GO:0099024 | plasma membrane invagination | 2 | 1.690 | 0.233 | 0.961 | 0.611 | -4.116 | 5.400 |
| GO:0036090 | cleavage furrow ingression | 2 | 0.903 | 0.208 | 0.944 | 0.985 | null | null |
| GO:1990386 | mitotic cleavage furrow ingression | 2 | 0.301 | 0.005 | 0.957 | 0.754 | null | null |
| GO:0048814 | regulation of dendrite morphogenesis | 2 | 1.255 | 0.082 | 0.881 | 0.748 | null | null |
| GO:0051491 | positive regulation of filopodium assembly | 2 | 1.041 | 0.048 | 0.888 | 0.715 | null | null |
| GO:1902743 | regulation of lamellipodium organization | 2 | 1.000 | 0.044 | 0.927 | 0.709 | null | null |
| GO:0044782 | cilium organization | 2 | 2.568 | 1.787 | 0.946 | 0.803 | null | null |
| GO:0060271 | cilium assembly | 2 | 2.537 | 1.661 | 0.941 | 0.960 | null | null |
| GO:0006029 | proteoglycan metabolic process | 2 | 1.833 | 0.325 | 0.920 | 0.832 | null | null |
| GO:0006486 | protein glycosylation | 2 | 2.332 | 1.037 | 0.884 | 0.940 | null | null |
| GO:0006487 | protein N-linked glycosylation | 2 | 1.708 | 0.242 | 0.899 | 0.808 | null | null |
| GO:0043413 | macromolecule glycosylation | 2 | 2.332 | 1.037 | 0.931 | 0.970 | null | null |
| GO:1901264 | carbohydrate derivative transport | 2 | 1.792 | 0.295 | 0.953 | 0.499 | 4.476 | 4.577 |
| GO:1901655 | cellular response to ketone | 2 | 1.114 | 0.058 | 0.932 | 0.519 | -1.120 | 6.532 |
| GO:1901657 | glycosyl compound metabolic process | 2 | 1.748 | 0.266 | 0.938 | 0.653 | 0.202 | -5.571 |
| GO:1901879 | regulation of protein depolymerization | 2 | 1.806 | 0.305 | 0.917 | 0.587 | 1.457 | -2.100 |
| GO:0030834 | regulation of actin filament depolymerization | 2 | 1.681 | 0.228 | 0.870 | 0.940 | null | null |
| GO:0030835 | negative regulation of actin filament depolymerization | 2 | 1.633 | 0.203 | 0.840 | 0.921 | null | null |
| GO:0043242 | negative regulation of protein-containing complex disassembly | 2 | 1.771 | 0.281 | 0.884 | 0.946 | null | null |
| GO:1901880 | negative regulation of protein depolymerization | 2 | 1.724 | 0.252 | 0.885 | 0.962 | null | null |
| GO:0002761 | regulation of myeloid leukocyte differentiation | 2 | 1.462 | 0.136 | 0.867 | 0.898 | null | null |
| GO:0002762 | negative regulation of myeloid leukocyte differentiation | 2 | 1.000 | 0.044 | 0.850 | 0.948 | null | null |
| GO:0045580 | regulation of T cell differentiation | 2 | 1.362 | 0.107 | 0.866 | 0.862 | null | null |
| GO:0045582 | positive regulation of T cell differentiation | 2 | 0.903 | 0.034 | 0.842 | 0.913 | null | null |
| GO:0045619 | regulation of lymphocyte differentiation | 2 | 1.477 | 0.140 | 0.864 | 0.882 | null | null |
| GO:0045621 | positive regulation of lymphocyte differentiation | 2 | 1.114 | 0.058 | 0.841 | 0.947 | null | null |
| GO:0045638 | negative regulation of myeloid cell differentiation | 2 | 1.146 | 0.063 | 0.849 | 0.828 | null | null |
| GO:0045670 | regulation of osteoclast differentiation | 2 | 0.778 | 0.024 | 0.882 | 0.773 | null | null |
| GO:0045671 | negative regulation of osteoclast differentiation | 2 | 0.602 | 0.015 | 0.860 | 0.883 | null | null |
| GO:1902106 | negative regulation of leukocyte differentiation | 2 | 1.301 | 0.092 | 0.842 | 0.852 | null | null |
| GO:1902107 | positive regulation of leukocyte differentiation | 2 | 1.301 | 0.092 | 0.839 | 0.852 | null | null |
| GO:1903707 | negative regulation of hemopoiesis | 2 | 1.301 | 0.092 | 0.843 | 0.852 | null | null |
| GO:1903708 | positive regulation of hemopoiesis | 2 | 1.362 | 0.107 | 0.838 | 0.862 | null | null |
| GO:1902116 | negative regulation of organelle assembly | 2 | 1.000 | 0.044 | 0.895 | 0.628 | 2.566 | -1.827 |
| GO:0017015 | regulation of transforming growth factor beta receptor signaling pathway | 2 | 1.431 | 0.126 | 0.930 | 0.811 | null | null |
| GO:0070372 | regulation of ERK1 and ERK2 cascade | 2 | 2.037 | 0.523 | 0.919 | 0.856 | null | null |
| GO:0090101 | negative regulation of transmembrane receptor protein serine/threonine kinase signaling pathway | 2 | 1.820 | 0.315 | 0.892 | 0.754 | null | null |
| GO:0032231 | regulation of actin filament bundle assembly | 2 | 1.380 | 0.111 | 0.913 | 0.730 | null | null |
| GO:0032233 | positive regulation of actin filament bundle assembly | 2 | 1.301 | 0.092 | 0.878 | 0.955 | null | null |
| GO:0051492 | regulation of stress fiber assembly | 2 | 1.204 | 0.073 | 0.916 | 0.706 | null | null |
| GO:0051496 | positive regulation of stress fiber assembly | 2 | 1.079 | 0.053 | 0.883 | 0.920 | null | null |
| GO:1903008 | organelle disassembly | 2 | 1.792 | 0.295 | 0.959 | 0.411 | -4.469 | 6.182 |
| GO:1903010 | regulation of bone development | 2 | 0.954 | 0.039 | 0.898 | 0.500 | 5.620 | -3.607 |
| GO:0014041 | regulation of neuron maturation | 2 | 0.301 | 0.005 | 0.920 | 1.002 | null | null |
| GO:0014042 | positive regulation of neuron maturation | 2 | 0.301 | 0.005 | 0.892 | 1.002 | null | null |
| GO:1904799 | regulation of neuron remodeling | 2 | 0.301 | 0.005 | 0.920 | 1.002 | null | null |
| GO:1904801 | positive regulation of neuron remodeling | 2 | 0.301 | 0.005 | 0.892 | 1.002 | null | null |
| GO:1903844 | regulation of cellular response to transforming growth factor beta stimulus | 2 | 1.431 | 0.126 | 0.941 | 0.386 | 6.940 | 0.274 |
| GO:1904892 | regulation of receptor signaling pathway via STAT | 2 | 1.342 | 0.102 | 0.932 | 0.403 | 6.594 | 0.800 |
| GO:0006835 | dicarboxylic acid transport | 2 | 1.748 | 0.266 | 0.950 | 0.804 | null | null |
| GO:0015800 | acidic amino acid transport | 2 | 1.431 | 0.126 | 0.952 | 0.750 | null | null |
| GO:1905330 | regulation of morphogenesis of an epithelium | 2 | 1.431 | 0.126 | 0.915 | 0.657 | 6.139 | -3.206 |
| GO:2000242 | negative regulation of reproductive process | 2 | 1.380 | 0.111 | 0.925 | 0.817 | null | null |
| GO:2000243 | positive regulation of reproductive process | 2 | 1.929 | 0.407 | 0.911 | 0.753 | null | null |
| GO:1905881 | positive regulation of oogenesis | 2 | 0.301 | 0.005 | 0.874 | 0.595 | 5.475 | -2.932 |
| GO:0060281 | regulation of oocyte development | 2 | 0.602 | 0.015 | 0.895 | 0.837 | null | null |
| GO:0060282 | positive regulation of oocyte development | 2 | 0.477 | 0.010 | 0.870 | 0.869 | null | null |
| GO:1990089 | response to nerve growth factor | 2 | 1.041 | 0.048 | 0.960 | 0.607 | -0.108 | 4.448 |
| GO:1990090 | cellular response to nerve growth factor stimulus | 2 | 1.041 | 0.048 | 0.960 | 0.160 | 0.066 | 4.441 |
| GO:0045765 | regulation of angiogenesis | 2 | 1.996 | 0.475 | 0.874 | 0.900 | null | null |
| GO:0045766 | positive regulation of angiogenesis | 2 | 1.580 | 0.179 | 0.849 | 0.752 | null | null |
| GO:1901342 | regulation of vasculature development | 2 | 2.025 | 0.509 | 0.876 | 0.735 | null | null |
| GO:1904018 | positive regulation of vasculature development | 2 | 1.580 | 0.179 | 0.851 | 0.831 | null | null |
| GO:2000136 | regulation of cell proliferation involved in heart morphogenesis | 2 | 0.477 | 0.010 | 0.963 | 0.569 | 0.137 | 0.091 |
| GO:2000377 | regulation of reactive oxygen species metabolic process | 2 | 1.114 | 0.058 | 0.936 | 0.227 | 2.761 | 1.289 |
| GO:2000378 | negative regulation of reactive oxygen species metabolic process | 2 | 0.699 | 0.019 | 0.911 | 0.206 | 4.212 | 0.098 |
| GO:1903426 | regulation of reactive oxygen species biosynthetic process | 2 | 0.778 | 0.024 | 0.937 | 0.883 | null | null |
| GO:1903427 | negative regulation of reactive oxygen species biosynthetic process | 2 | 0.602 | 0.015 | 0.912 | 0.857 | null | null |
| GO:2000736 | regulation of stem cell differentiation | 2 | 1.613 | 0.194 | 0.905 | 0.581 | 6.102 | -3.088 |
| GO:2000826 | regulation of heart morphogenesis | 2 | 0.477 | 0.010 | 0.929 | 0.447 | 6.152 | -3.315 |
|  |  |  |  |  |  |  |  |  |

**Table S6:** GO terms associated with the genes differentially expressed between natural-origin offspring and hatchery-origin offspring and reduced according to REVIGO. This table complements the terms highlighted in Figure 4 and lists all GO terms from each k.means cluster where each row indicates the HEX colors used to delineate each cluster, Count represents the number distinct DEGs that contain a particular GO term, and the GO term description itself.

| **Group** | **Count** | **Go term** |
| --- | --- | --- |
| #1B9E77 | 60 | anatomical structure development |
| #1B9E77 | 47 | cellular developmental process |
| #1B9E77 | 46 | cell differentiation |
| #1B9E77 | 40 | animal organ development |
| #1B9E77 | 35 | anatomical structure morphogenesis |
| #1B9E77 | 27 | tissue development |
| #1B9E77 | 19 | animal organ morphogenesis |
| #1B9E77 | 17 | anatomical structure formation involved in morphogenesis |
| #1B9E77 | 17 | epithelium development |
| #1B9E77 | 17 | muscle structure development |
| #1B9E77 | 16 | cellular anatomical entity morphogenesis |
| #1B9E77 | 11 | epithelial cell differentiation |
| #1B9E77 | 11 | muscle cell development |
| #1B9E77 | 10 | cell morphogenesis |
| #1B9E77 | 9 | epithelial cell development |
| #1B9E77 | 9 | sensory organ development |
| #1B9E77 | 9 | striated muscle tissue development |
| #1B9E77 | 9 | head development |
| #1B9E77 | 9 | muscle tissue development |
| #1B9E77 | 8 | hemopoiesis |
| #1B9E77 | 6 | columnar/cuboidal epithelial cell development |
| #1B9E77 | 6 | imaginal disc development |
| #1B9E77 | 6 | developmental maturation |
| #1B9E77 | 6 | cell fate commitment |
| #1B9E77 | 5 | wing disc development |
| #1B9E77 | 5 | cell maturation |
| #1B9E77 | 4 | skeletal muscle cell differentiation |
| #1B9E77 | 4 | gland development |
| #1B9E77 | 4 | connective tissue development |
| #1B9E77 | 3 | cell fate specification |
| #1B9E77 | 3 | hematopoietic progenitor cell differentiation |
| #1B9E77 | 3 | somatic muscle development |
| #1B9E77 | 3 | stem cell differentiation |
| #1B9E77 | 3 | cranial skeletal system development |
| #1B9E77 | 2 | placenta development |
| #1B9E77 | 2 | leukocyte differentiation |
| #1B9E77 | 2 | endothelium development |
| #1B9E77 | 2 | epidermis development |
| #1B9E77 | 2 | regeneration |
| #1B9E77 | 2 | odontogenesis of dentin-containing tooth |
| #1B9E77 | 2 | odontogenesis |
| #1B9E77 | 2 | skin development |
| #1B9E77 | 2 | fat cell differentiation |
| #1B9E77 | 2 | developmental growth |
| #1B9E77 | 2 | mesenchyme development |
| #1B9E77 | 2 | adipose tissue development |
| #1B9E77 | 2 | ectodermal placode formation |
| #1B9E77 | 2 | trabecula morphogenesis |
| #1B9E77 | 2 | heart trabecula morphogenesis |
| #1B9E77 | 2 | ectodermal placode development |
| #D95F02 | 56 | multicellular organism development |
| #D95F02 | 49 | system development |
| #D95F02 | 26 | nervous system development |
| #D95F02 | 16 | embryo development |
| #D95F02 | 14 | neuron development |
| #D95F02 | 12 | post-embryonic development |
| #D95F02 | 11 | pattern specification process |
| #D95F02 | 11 | tube development |
| #D95F02 | 10 | skeletal system development |
| #D95F02 | 10 | tube morphogenesis |
| #D95F02 | 10 | circulatory system development |
| #D95F02 | 9 | immune system development |
| #D95F02 | 9 | central nervous system development |
| #D95F02 | 9 | embryo development ending in birth or egg hatching |
| #D95F02 | 9 | hematopoietic or lymphoid organ development |
| #D95F02 | 8 | eye development |
| #D95F02 | 8 | regionalization |
| #D95F02 | 8 | brain development |
| #D95F02 | 7 | larval development |
| #D95F02 | 7 | heart development |
| #D95F02 | 7 | bone development |
| #D95F02 | 6 | compound eye morphogenesis |
| #D95F02 | 6 | compound eye development |
| #D95F02 | 6 | reproductive system development |
| #D95F02 | 5 | gliogenesis |
| #D95F02 | 4 | metamorphosis |
| #D95F02 | 4 | chordate embryonic development |
| #D95F02 | 4 | bone morphogenesis |
| #D95F02 | 3 | cardiac chamber development |
| #D95F02 | 3 | peripheral nervous system development |
| #D95F02 | 3 | nerve development |
| #D95F02 | 2 | osteoblast differentiation |
| #D95F02 | 2 | morphogenesis of a branching structure |
| #D95F02 | 2 | liver development |
| #D95F02 | 2 | outflow tract morphogenesis |
| #D95F02 | 2 | cardiac ventricle development |
| #D95F02 | 2 | ensheathment of neurons |
| #D95F02 | 2 | cellularization |
| #D95F02 | 2 | blastoderm segmentation |
| #D95F02 | 2 | neuron recognition |
| #D95F02 | 2 | glial cell differentiation |
| #D95F02 | 2 | cranial nerve development |
| #D95F02 | 2 | preganglionic parasympathetic fiber development |
| #D95F02 | 2 | cardiocyte differentiation |
| #D95F02 | 2 | tube formation |
| #D95F02 | 2 | endocrine system development |
| #D95F02 | 2 | central nervous system segmentation |
| #D95F02 | 2 | brain segmentation |
| #D95F02 | 2 | photoreceptor cell development |
| #D95F02 | 2 | myelination |
| #D95F02 | 2 | photoreceptor cell differentiation |
| #D95F02 | 2 | autonomic nervous system development |
| #D95F02 | 2 | appendage development |
| #D95F02 | 2 | imaginal disc-derived appendage development |
| #D95F02 | 2 | branching morphogenesis of an epithelial tube |
| #D95F02 | 2 | digestive system development |
| #D95F02 | 2 | respiratory system development |
| #D95F02 | 2 | hepaticobiliary system development |
| #D95F02 | 2 | somite development |
| #7570B3 | 22 | system process |
| #7570B3 | 18 | behavior |
| #7570B3 | 12 | muscle system process |
| #7570B3 | 11 | locomotory behavior |
| #7570B3 | 8 | cell activation |
| #7570B3 | 8 | circulatory system process |
| #7570B3 | 8 | blood circulation |
| #7570B3 | 8 | leukocyte activation |
| #7570B3 | 7 | muscle contraction |
| #7570B3 | 6 | adult locomotory behavior |
| #7570B3 | 6 | cellular component assembly involved in morphogenesis |
| #7570B3 | 5 | ossification |
| #7570B3 | 5 | reproductive behavior |
| #7570B3 | 4 | mating behavior |
| #7570B3 | 4 | dendrite development |
| #7570B3 | 4 | cognition |
| #7570B3 | 3 | multi-multicellular organism process |
| #7570B3 | 3 | dendrite morphogenesis |
| #7570B3 | 2 | epithelial cell migration |
| #7570B3 | 2 | muscle hypertrophy |
| #7570B3 | 2 | striated muscle hypertrophy |
| #7570B3 | 2 | T-tubule organization |
| #7570B3 | 2 | sensory perception of mechanical stimulus |
| #7570B3 | 2 | relaxation of skeletal muscle |
| #7570B3 | 2 | tissue migration |
| #E7298A | 40 | organelle organization |
| #E7298A | 32 | cellular component biogenesis |
| #E7298A | 16 | cytoskeleton organization |
| #E7298A | 15 | protein-containing complex organization |
| #E7298A | 15 | membrane organization |
| #E7298A | 14 | protein-containing complex assembly |
| #E7298A | 12 | mitochondrion organization |
| #E7298A | 12 | actin cytoskeleton organization |
| #E7298A | 11 | cell projection organization |
| #E7298A | 11 | organelle assembly |
| #E7298A | 11 | plasma membrane bounded cell projection organization |
| #E7298A | 10 | supramolecular fiber organization |
| #E7298A | 6 | endomembrane system organization |
| #E7298A | 6 | extracellular structure organization |
| #E7298A | 6 | protein complex oligomerization |
| #E7298A | 6 | chromosome organization |
| #E7298A | 5 | vesicle organization |
| #E7298A | 5 | extracellular matrix organization |
| #E7298A | 5 | organelle fission |
| #E7298A | 5 | protein homooligomerization |
| #E7298A | 4 | cellular component disassembly |
| #E7298A | 3 | nuclear division |
| #E7298A | 3 | mitochondrial membrane organization |
| #E7298A | 3 | heterochromatin organization |
| #E7298A | 2 | endoplasmic reticulum organization |
| #E7298A | 2 | vacuole organization |
| #E7298A | 2 | spindle organization |
| #E7298A | 2 | membrane invagination |
| #E7298A | 2 | extracellular matrix disassembly |
| #E7298A | 2 | cortical cytoskeleton organization |
| #E7298A | 2 | cell junction organization |
| #E7298A | 2 | adherens junction assembly |
| #E7298A | 2 | organelle fusion |
| #E7298A | 2 | synapse organization |
| #E7298A | 2 | membrane fusion |
| #E7298A | 2 | plasma membrane invagination |
| #E7298A | 2 | organelle disassembly |
| #66A61E | 45 | response to chemical |
| #66A61E | 40 | cellular response to stimulus |
| #66A61E | 37 | response to stress |
| #66A61E | 31 | response to organic substance |
| #66A61E | 29 | response to external stimulus |
| #66A61E | 26 | signal transduction |
| #66A61E | 22 | response to endogenous stimulus |
| #66A61E | 20 | response to abiotic stimulus |
| #66A61E | 19 | response to oxygen-containing compound |
| #66A61E | 18 | response to nitrogen compound |
| #66A61E | 17 | intracellular signal transduction |
| #66A61E | 15 | cellular response to stress |
| #66A61E | 11 | cell surface receptor signaling pathway |
| #66A61E | 11 | response to organic cyclic compound |
| #66A61E | 10 | immune response |
| #66A61E | 10 | response to biotic stimulus |
| #66A61E | 10 | response to lipid |
| #66A61E | 10 | response to other organism |
| #66A61E | 9 | defense response |
| #66A61E | 9 | response to extracellular stimulus |
| #66A61E | 9 | response to nutrient levels |
| #66A61E | 8 | response to oxidative stress |
| #66A61E | 8 | response to inorganic substance |
| #66A61E | 7 | response to radiation |
| #66A61E | 6 | response to acid chemical |
| #66A61E | 6 | response to wounding |
| #66A61E | 6 | response to metal ion |
| #66A61E | 6 | response to endoplasmic reticulum stress |
| #66A61E | 6 | wound healing |
| #66A61E | 6 | response to antibiotic |
| #66A61E | 6 | cellular response to organic cyclic compound |
| #66A61E | 5 | enzyme-linked receptor protein signaling pathway |
| #66A61E | 5 | response to toxic substance |
| #66A61E | 5 | cellular response to oxidative stress |
| #66A61E | 5 | response to topologically incorrect protein |
| #66A61E | 5 | cellular response to topologically incorrect protein |
| #66A61E | 5 | cellular response to external stimulus |
| #66A61E | 4 | transmembrane receptor protein tyrosine kinase signaling pathway |
| #66A61E | 4 | defense response to Gram-negative bacterium |
| #66A61E | 4 | cellular response to acid chemical |
| #66A61E | 4 | cellular response to lipid |
| #66A61E | 4 | cellular response to environmental stimulus |
| #66A61E | 4 | response to xenobiotic stimulus |
| #66A61E | 3 | response to cold |
| #66A61E | 3 | response to alkaloid |
| #66A61E | 3 | response to organophosphorus |
| #66A61E | 3 | detection of stimulus |
| #66A61E | 3 | cellular response to antibiotic |
| #66A61E | 3 | response to alcohol |
| #66A61E | 3 | response to ketone |
| #66A61E | 2 | MAPK cascade |
| #66A61E | 2 | inflammatory response |
| #66A61E | 2 | ER-nucleus signaling pathway |
| #66A61E | 2 | G protein-coupled receptor signaling pathway |
| #66A61E | 2 | G protein-coupled receptor signaling pathway, coupled to cyclic nucleotide second messenger |
| #66A61E | 2 | adenylate cyclase-modulating G protein-coupled receptor signaling pathway |
| #66A61E | 2 | adenylate cyclase-inhibiting G protein-coupled receptor signaling pathway |
| #66A61E | 2 | Ras protein signal transduction |
| #66A61E | 2 | response to UV |
| #66A61E | 2 | entrainment of circadian clock |
| #66A61E | 2 | response to carbohydrate |
| #66A61E | 2 | response to glucose |
| #66A61E | 2 | response to lithium ion |
| #66A61E | 2 | response to inactivity |
| #66A61E | 2 | response to muscle inactivity |
| #66A61E | 2 | calcium-mediated signaling |
| #66A61E | 2 | stress-activated protein kinase signaling cascade |
| #66A61E | 2 | detection of mechanical stimulus |
| #66A61E | 2 | stress-activated MAPK cascade |
| #66A61E | 2 | response to anesthetic |
| #66A61E | 2 | response to thyroid hormone |
| #66A61E | 2 | detoxification |
| #66A61E | 2 | cellular response to ketone |
| #E6AB02 | 49 | transport |
| #E6AB02 | 31 | cellular localization |
| #E6AB02 | 26 | macromolecule localization |
| #E6AB02 | 25 | organic substance transport |
| #E6AB02 | 23 | nitrogen compound transport |
| #E6AB02 | 21 | vesicle-mediated transport |
| #E6AB02 | 19 | monoatomic ion transport |
| #E6AB02 | 17 | transmembrane transport |
| #E6AB02 | 16 | monoatomic ion transmembrane transport |
| #E6AB02 | 16 | intracellular transport |
| #E6AB02 | 15 | secretion by cell |
| #E6AB02 | 15 | secretion |
| #E6AB02 | 13 | peptide transport |
| #E6AB02 | 13 | amide transport |
| #E6AB02 | 8 | organelle localization |
| #E6AB02 | 7 | mitochondrial transport |
| #E6AB02 | 6 | endocytosis |
| #E6AB02 | 6 | organic anion transport |
| #E6AB02 | 6 | Golgi vesicle transport |
| #E6AB02 | 6 | import into cell |
| #E6AB02 | 6 | mitochondrial transmembrane transport |
| #E6AB02 | 6 | protein localization to cell periphery |
| #E6AB02 | 5 | organic acid transport |
| #E6AB02 | 5 | carboxylic acid transmembrane transport |
| #E6AB02 | 4 | neurotransmitter transport |
| #E6AB02 | 4 | nucleobase-containing compound transport |
| #E6AB02 | 4 | maintenance of location |
| #E6AB02 | 4 | maintenance of location in cell |
| #E6AB02 | 4 | sarcoplasmic reticulum calcium ion transport |
| #E6AB02 | 3 | RNA localization |
| #E6AB02 | 3 | retrograde vesicle-mediated transport, Golgi to endoplasmic reticulum |
| #E6AB02 | 3 | establishment of organelle localization |
| #E6AB02 | 3 | vesicle-mediated transport in synapse |
| #E6AB02 | 3 | synaptic vesicle cycle |
| #E6AB02 | 2 | transition metal ion transport |
| #E6AB02 | 2 | iron ion transport |
| #E6AB02 | 2 | xenobiotic transmembrane transport |
| #E6AB02 | 2 | nucleocytoplasmic transport |
| #E6AB02 | 2 | organic cation transport |
| #E6AB02 | 2 | inorganic anion transport |
| #E6AB02 | 2 | protein import |
| #E6AB02 | 2 | receptor internalization |
| #E6AB02 | 2 | protein localization to nucleus |
| #E6AB02 | 2 | xenobiotic transport |
| #E6AB02 | 2 | RNA transport |
| #E6AB02 | 2 | nuclear transport |
| #E6AB02 | 2 | sulfur compound transport |
| #E6AB02 | 2 | carbohydrate derivative transport |
| #E6AB02 | 2 | ammonium transmembrane transport |
| #A6761D | 16 | negative regulation of response to stimulus |
| #A6761D | 15 | regulation of intracellular signal transduction |
| #A6761D | 14 | negative regulation of cell communication |
| #A6761D | 14 | negative regulation of signaling |
| #A6761D | 12 | regulation of response to stress |
| #A6761D | 10 | positive regulation of response to stimulus |
| #A6761D | 9 | positive regulation of cell communication |
| #A6761D | 9 | positive regulation of signaling |
| #A6761D | 8 | regulation of MAPK cascade |
| #A6761D | 7 | regulation of defense response |
| #A6761D | 6 | regulation of immune response |
| #A6761D | 5 | modulation of chemical synaptic transmission |
| #A6761D | 5 | regulation of trans-synaptic signaling |
| #A6761D | 4 | regulation of response to external stimulus |
| #A6761D | 3 | regulation of humoral immune response |
| #A6761D | 3 | regulation of TOR signaling |
| #A6761D | 3 | regulation of stress-activated MAPK cascade |
| #A6761D | 3 | regulation of insulin receptor signaling pathway |
| #A6761D | 3 | negative regulation of insulin receptor signaling pathway |
| #A6761D | 3 | regulation of phosphatidylinositol 3-kinase/protein kinase B signal transduction |
| #A6761D | 3 | regulation of stress-activated protein kinase signaling cascade |
| #A6761D | 3 | regulation of transmembrane receptor protein serine/threonine kinase signaling pathway |
| #A6761D | 3 | regulation of cellular response to insulin stimulus |
| #A6761D | 2 | regulation of Wnt signaling pathway |
| #A6761D | 2 | regulation of canonical NF-kappaB signal transduction |
| #A6761D | 2 | regulation of JNK cascade |
| #A6761D | 2 | regulation of receptor signaling pathway via JAK-STAT |
| #A6761D | 2 | positive regulation of phosphatidylinositol 3-kinase/protein kinase B signal transduction |
| #A6761D | 2 | regulation of canonical Wnt signaling pathway |
| #A6761D | 2 | regulation of cellular response to growth factor stimulus |
| #A6761D | 2 | regulation of cellular response to transforming growth factor beta stimulus |
| #A6761D | 2 | regulation of receptor signaling pathway via STAT |
| #666666 | 30 | regulation of multicellular organismal development |
| #666666 | 14 | negative regulation of developmental process |
| #666666 | 11 | regulation of system process |
| #666666 | 11 | regulation of neuron differentiation |
| #666666 | 9 | regulation of developmental growth |
| #666666 | 8 | regulation of muscle system process |
| #666666 | 7 | regulation of striated muscle tissue development |
| #666666 | 7 | regulation of muscle organ development |
| #666666 | 7 | regulation of muscle tissue development |
| #666666 | 6 | regulation of cell morphogenesis |
| #666666 | 5 | regulation of embryonic development |
| #666666 | 5 | regulation of animal organ morphogenesis |
| #666666 | 4 | regulation of myotube differentiation |
| #666666 | 4 | regulation of ossification |
| #666666 | 4 | regulation of cell activation |
| #666666 | 4 | regulation of muscle cell differentiation |
| #666666 | 4 | regulation of leukocyte differentiation |
| #666666 | 4 | regulation of cardiac conduction |
| #666666 | 3 | regulation of fat cell differentiation |
| #666666 | 3 | positive regulation of fat cell differentiation |
| #666666 | 3 | regulation of cell maturation |
| #666666 | 3 | positive regulation of cell maturation |
| #666666 | 3 | regulation of oogenesis |
| #666666 | 3 | regulation of skeletal muscle cell differentiation |
| #666666 | 2 | positive regulation of multicellular organism growth |
| #666666 | 2 | negative regulation of organ growth |
| #666666 | 2 | regulation of behavior |
| #666666 | 2 | regulation of morphogenesis of a branching structure |
| #666666 | 2 | regulation of biomineral tissue development |
| #666666 | 2 | regulation of bone development |
| #666666 | 2 | regulation of morphogenesis of an epithelium |
| #666666 | 2 | positive regulation of oogenesis |
| #666666 | 2 | regulation of stem cell differentiation |
| #666666 | 2 | regulation of heart morphogenesis |
| #B2DFEE | 73 | cellular metabolic process |
| #B2DFEE | 72 | organic substance metabolic process |
| #B2DFEE | 69 | primary metabolic process |
| #B2DFEE | 63 | nitrogen compound metabolic process |
| #B2DFEE | 55 | organonitrogen compound metabolic process |
| #B2DFEE | 51 | macromolecule metabolic process |
| #B2DFEE | 39 | protein metabolic process |
| #B2DFEE | 37 | biosynthetic process |
| #B2DFEE | 37 | organic substance biosynthetic process |
| #B2DFEE | 31 | cellular nitrogen compound metabolic process |
| #B2DFEE | 28 | protein modification process |
| #B2DFEE | 28 | macromolecule modification |
| #B2DFEE | 28 | small molecule metabolic process |
| #B2DFEE | 28 | organic cyclic compound metabolic process |
| #B2DFEE | 27 | heterocycle metabolic process |
| #B2DFEE | 26 | catabolic process |
| #B2DFEE | 25 | cellular aromatic compound metabolic process |
| #B2DFEE | 25 | cellular catabolic process |
| #B2DFEE | 22 | phosphorus metabolic process |
| #B2DFEE | 22 | organonitrogen compound biosynthetic process |
| #B2DFEE | 20 | cellular nitrogen compound biosynthetic process |
| #B2DFEE | 17 | heterocycle biosynthetic process |
| #B2DFEE | 17 | aromatic compound biosynthetic process |
| #B2DFEE | 17 | organic cyclic compound biosynthetic process |
| #B2DFEE | 16 | lipid metabolic process |
| #B2DFEE | 16 | nucleobase-containing compound biosynthetic process |
| #B2DFEE | 15 | proteolysis |
| #B2DFEE | 15 | organophosphate metabolic process |
| #B2DFEE | 15 | carboxylic acid metabolic process |
| #B2DFEE | 15 | cellular lipid metabolic process |
| #B2DFEE | 15 | carbohydrate derivative metabolic process |
| #B2DFEE | 12 | purine-containing compound metabolic process |
| #B2DFEE | 10 | RNA metabolic process |
| #B2DFEE | 10 | organophosphate biosynthetic process |
| #B2DFEE | 10 | carbohydrate derivative biosynthetic process |
| #B2DFEE | 9 | small molecule catabolic process |
| #B2DFEE | 8 | peptidyl-amino acid modification |
| #B2DFEE | 8 | amide metabolic process |
| #B2DFEE | 7 | nucleoside monophosphate metabolic process |
| #B2DFEE | 7 | ribonucleoside monophosphate metabolic process |
| #B2DFEE | 7 | small molecule biosynthetic process |
| #B2DFEE | 6 | generation of precursor metabolites and energy |
| #B2DFEE | 6 | protein phosphorylation |
| #B2DFEE | 6 | peptide metabolic process |
| #B2DFEE | 6 | amino acid metabolic process |
| #B2DFEE | 6 | cellular modified amino acid metabolic process |
| #B2DFEE | 6 | sulfur compound metabolic process |
| #B2DFEE | 6 | lipid oxidation |
| #B2DFEE | 5 | protein polyubiquitination |
| #B2DFEE | 5 | carbohydrate metabolic process |
| #B2DFEE | 4 | electron transport chain |
| #B2DFEE | 4 | sulfur compound biosynthetic process |
| #B2DFEE | 4 | heterocycle catabolic process |
| #B2DFEE | 4 | organic cyclic compound catabolic process |
| #B2DFEE | 3 | monosaccharide metabolic process |
| #B2DFEE | 3 | aminoglycan metabolic process |
| #B2DFEE | 3 | protein dephosphorylation |
| #B2DFEE | 3 | neutral lipid metabolic process |
| #B2DFEE | 3 | dephosphorylation |
| #B2DFEE | 3 | peptidyl-serine modification |
| #B2DFEE | 3 | glycosaminoglycan metabolic process |
| #B2DFEE | 3 | SCF-dependent proteasomal ubiquitin-dependent protein catabolic process |
| #B2DFEE | 3 | receptor metabolic process |
| #B2DFEE | 3 | ammonium ion metabolic process |
| #B2DFEE | 3 | adaptive thermogenesis |
| #B2DFEE | 2 | protein lipidation |
| #B2DFEE | 2 | creatine metabolic process |
| #B2DFEE | 2 | autophagy |
| #B2DFEE | 2 | mRNA metabolic process |
| #B2DFEE | 2 | protein processing |
| #B2DFEE | 2 | protein deubiquitination |
| #B2DFEE | 2 | peptidyl-tyrosine modification |
| #B2DFEE | 2 | protein N-linked glycosylation via asparagine |
| #B2DFEE | 2 | C-terminal protein amino acid modification |
| #B2DFEE | 2 | lipoprotein metabolic process |
| #B2DFEE | 2 | cellular modified amino acid biosynthetic process |
| #B2DFEE | 2 | cellular metabolic compound salvage |
| #B2DFEE | 2 | purine-containing compound salvage |
| #B2DFEE | 2 | protein autophosphorylation |
| #B2DFEE | 2 | dermatan sulfate proteoglycan metabolic process |
| #B2DFEE | 2 | protein maturation |
| #B2DFEE | 2 | glycosylation |
| #B2DFEE | 2 | protein modification by small protein removal |
| #B2DFEE |  | glycosyl compound metabolic process |
